# Development of mammalian cell logic gates controlled by unnatural amino acids

**DOI:** 10.1016/j.crmeth.2021.100073

**Published:** 2021-09-16

**Authors:** Emily M. Mills, Victoria L. Barlow, Arwyn T. Jones, Yu-Hsuan Tsai

**Affiliations:** 1 School of Chemistry, Cardiff University, Main Building, Park Place, Cardiff, Wales CF10 3AT, UK; 2 School of Pharmacy and Pharmaceutical Sciences, Cardiff University, Redwood Building, Cardiff, Wales CF10 3NB, UK; 3 Institute of Molecular Physiology, Shenzhen Bay Laboratory, Shenzhen, Guangdong 518132, China

**Keywords:** genetic code expansion, unnatural amino acid, non-canonical amino acid, quadruplet codon, pyrrolysyl tRNA, logic gate, synthetic biology

## Abstract

Mammalian cell logic gates hold great potential for wide-ranging applications. However, most of those currently available are controlled by drug(-like) molecules with inherent biological activities. To construct truly orthogonal circuits and artificial regulatory pathways, biologically inert molecules are ideal molecular switches. Here, we applied genetic code expansion and engineered logic gates controlled by two biologically inert unnatural amino acids. Genetic code expansion relies on orthogonal aminoacyl-tRNA synthetase/tRNA pairs for co-translational and site-specific unnatural amino acid incorporation conventionally in response to an amber (UAG) codon. By screening 11 quadruplet-decoding pyrrolysyl tRNA variants from the literature, we found that all variants decoding CUAG or AGGA tested here are functional in mammalian cells. Using a quadruplet-decoding orthogonal pair together with an amber-decoding pair, we constructed logic gates that can be successfully controlled by two different unnatural amino acids, expanding the scope of genetic code expansion and mammalian cell logic circuits.

## Highlights

• We evaluate the performance of 11 quadruplet-decoding tRNAs in HEK293
• Two tRNAs show high efficiency in decoding quadruplet codons
• We construct logic gates responding to two different unnatural amino acids
• These findings present an alternative approach to mammalian cell logic operations

## Motivation

Genetic code expansion employing an orthogonal aminoacyl-tRNA synthetase/tRNA pair for site-specific unnatural amino acid incorporation has a wide range of applications. However, a blank (orthogonal) codon is needed for each orthogonal tRNA. Although the amber stop codon is commonly used as the blank codon, the use of stop codons as the blank codons inevitably limits the number of possible different unnatural amino acids to be incorporated into proteins in a single cell. To overcome this limitation, we evaluated the capability of 11 Pyl tRNA variants for decoding quadruplet codons in mammalian cells. With efficient quadruplet-decoding orthogonal tRNA variants available, it is possible for simultaneous incorporation of more than three different unnatural amino acids in mammalian cells.

## Graphical abstract

**Figure:**
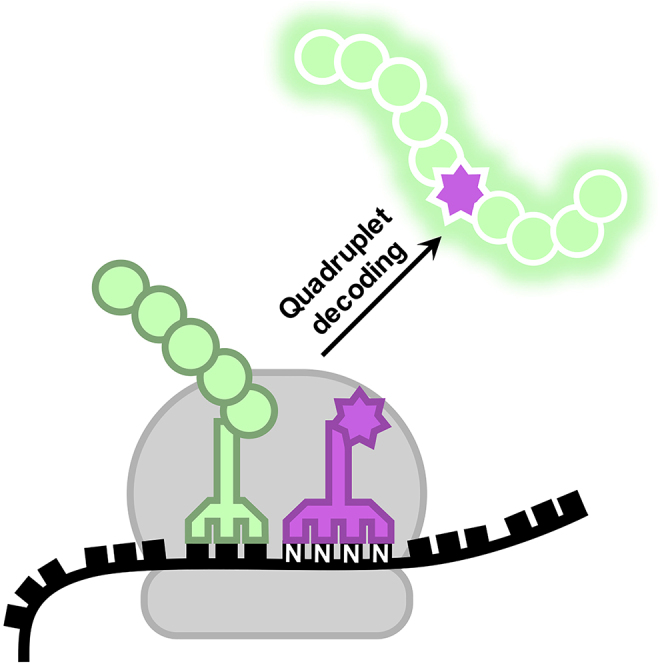

## Teaser

Logic gates enable regulation of protein function by small molecules, but these are often drug(-like) molecules with intrinsic biological activities. Mills et al. present an alternative approach through genetic code expansion, demonstrating that unnatural amino acids can act as biologically inert switches for effective mammalian cell logic operations.

## Introduction

Logic gates refers to integrated systems where an input controls a desired output. Employing logic gates in mammalian cells is a fast-developing area for intricate reversible cellular control, with significant biotechnological and biomedical applications (Cuthbertson and Nodwell, 2013; Fink et al., 2019; Nguyen et al., 2016; Ross et al., 2016; Wu et al., 2015; Zetsche et al., 2015). Some examples include novel sensors, diagnostics, as well as therapeutics (Brown et al., 2018; Kitada et al., 2018; Scheller and Fussenegger, 2019; Singh, 2014; Zhou et al., 2020). In particular, logic gates responding to small molecules can provide (spatio)temporal control by the user, permitting targeted manipulation of a designated phenotype. Specifically, biologically inert molecules that have no influence on endogenous cellular events are ideal molecular switches to control logic gates, therefore enabling the construction of truly orthogonal circuits and artificial regulatory pathways in cells.

**Figure 1:**
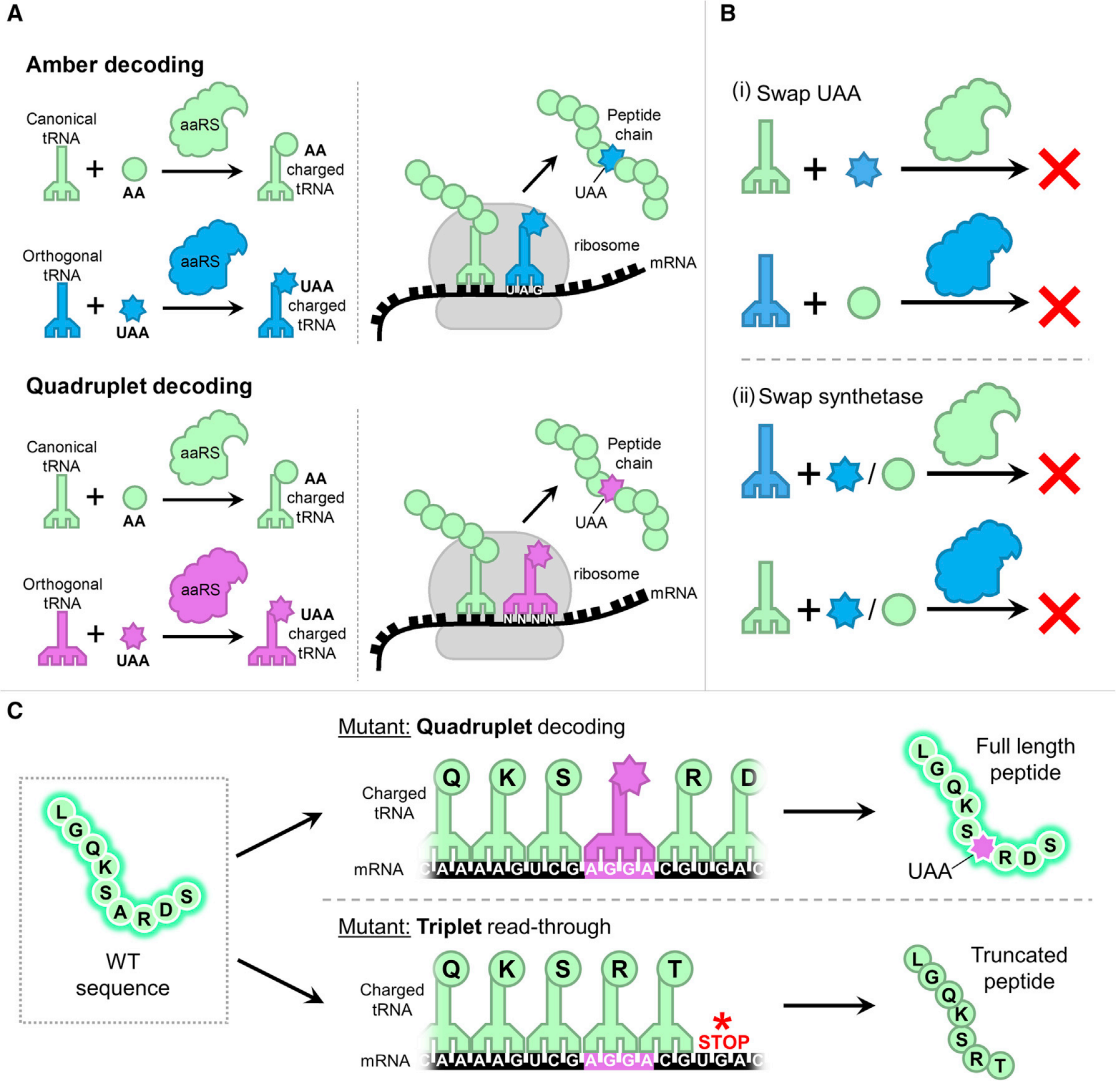
Principles of unnatural amino acid incorporation by genetic code expansion (A) Amber and quadruplet suppression. Charged tRNA molecules are generated by aminoacylation of tRNA molecules by their respective synthetases with the respective amino acid or unnatural amino acid. Charged tRNA molecules then enter the ribosome and interact with the complementary codon on the mRNA template before formation of the peptide bond with the brought-in amino acid. Abbreviations are as follows: AA, amino acid; UAA, unnatural amino acid. (B) Graphical representation of aaRS/tRNA pair orthogonality: (i) aaRS can only charge their paired tRNA with the respective amino acid, supplementation of a different amino acid results in no aminoacylation of tRNA; (ii) tRNA can only be aminoacylated by their respective aaRS, regardless of amino acid supplementation. (C) Principles imposed when using quadruplet decoding. Alongside the introduction of the quadruplet codon to the gene, a further point mutation is introduced downstream to the codon to ensure that, if the first three bases of the quadruplet are translated by the canonical triplet tRNA, an in-frame stop codon is generated downstream to the incorporation site enabling premature truncation. Moreover, if the quadruplet is correctly decoded, this point mutation remains silent and does not alter the overall amino acid sequence of the protein.

We hypothesized that this can be realized by using biologically inert unnatural (non-canonical) amino acids, which are artificial synthetic molecules and do not produce observable phenotypes or toxicities *in vitro* or *in vivo* (Chen et al., 2017; Han et al., 2017; Krogager et al., 2018; Liu et al., 2017; Suzuki et al., 2018). More importantly, such unnatural amino acids can be site-specifically incorporated into proteins in mammalian cells by repurposing the cellular translational machinery through the technique of genetic code expansion (Figure 1). This technique has wide-ranging applications in protein research (Chin, 2017; de la Torre and Chin, 2021; Dumas et al., 2015; Huang and Liu, 2018; Kato, 2019; Neumann-Staubitz and Neumann, 2016; Nodling et al., 2019), including the proposed use of unnatural amino acids as logic gate inputs. To incorporate an unnatural amino acid into a protein during translation, an orthogonal aminoacyl-tRNA synthetase (aaRS)/tRNA pair that selectively decodes a blank codon is required. The amber stop codon (UAG) is often used as the blank codon because it does not encode an amino acid and is the rarest codon in many organisms (Ivanov et al., 2001). The use of the amber codon to encode an unnatural amino acid is known as amber suppression (Figure 1A). To date, various amber suppressor orthogonal pairs have been developed (Chin, 2017; Dumas et al., 2015; Huang and Liu, 2018; Neumann-Staubitz and Neumann, 2016; Nodling et al., 2019). Within prokaryotic and eukaryotic systems, pyrrolysyl (Pyl)-tRNA synthetase (PylRS) and its cognate tRNA from the archaeal *Methanosarcina* species is arguably one of the most widely utilized and developed orthogonal pairs for unnatural amino acid incorporation. Nevertheless, various *Escherichia coli* aaRS enzymes (e.g., *Ec*LeuRS, *Ec*TyrRS, *Ec*TrpRS) and their cognate tRNAs have also been exploited and specifically engineered to function as amber suppressor orthogonal pairs in mammalian cells. It is possible to employ several orthogonal pairs for simultaneous incorporation of multiple unnatural amino acids in a single mammalian cell (Meineke et al., 2018, 2020; Xiao et al., 2013; Zheng et al., 2017, 2018). However, such applications are often thwarted as these orthogonal pairs were originally engineered for amber decoding. To attain site-specific incorporation of multiple unnatural amino acids within a single cell, orthogonal pairs need to decode unique blank codons and have no crossover interactions (Figure 1B). An emerging strategy to generate additional blank codons is to use quadruplet (four-base) codons. Employing quadruplet codons can greatly expand the genetic code, providing the possibility for simultaneous incorporation of three or more distinct unnatural amino acids within a single cell (Dunkelmann et al., 2020).

Indeed, quadruplet decoding has been well-explored in *E. coli* (de la Torre and Chin, 2021; Moore et al., 2000; Neumann et al., 2010; O'Connor, 2002; Wang et al., 2014; Wang et al., 2016; Willis and Chin, 2018), including a recent example demonstrating simultaneous incorporation of three different unnatural amino acids (Dunkelmann et al., 2020). However, quadruplet decoding in mammalian cells is still largely unexplored and remains in its infancy (Anderson et al., 2004; Chen et al., 2018; Niu et al., 2013; Taki et al., 2006).

Here, we recognized the potential applications of genetic code expansion in the development of orthogonal logic circuits and engineered novel AND and OR logic gates that can be controlled by two distinct unnatural amino acids. As this proposed system design required two mutually orthogonal aaRS/tRNA pairs to respond to the logic inputs, we set out to identify a quadruplet-decoding aaRS/tRNA pair that is orthogonal to an amber-decoding aaRS/tRNA pair in mammalian cells. Specifically, we investigated the quadruplet-decoding efficiency of 11 Pyl tRNA_XXXX_ variants previously tested in *E. coli* and found that all selected variants decoding AGGA (Niu et al., 2013; Wang et al., 2014) or CUAG (Wang et al., 2014) were functional in human embryonic kidney (HEK293) cells, whereas none of the tested variants were able to decode UAGN codons efficiently. We then confirmed the orthogonality of an amber-decoding pair to an AGGA-decoding PylRS/tRNA pair, enabling incorporation of two different unnatural amino acids in the same mammalian cell. After a series of system optimizations, we constructed AND and OR logic gates by using a split GFP reporter assay. To the best of our knowledge, this is the first example exploiting genetic code expansion in mammalian cell logic gates. This study not only expands the applications of genetic code expansion but also provides an alternative approach to engineer mammalian cell logic circuits.

## Results

### Quadruplet-decoding analysis

**Figure 2:**
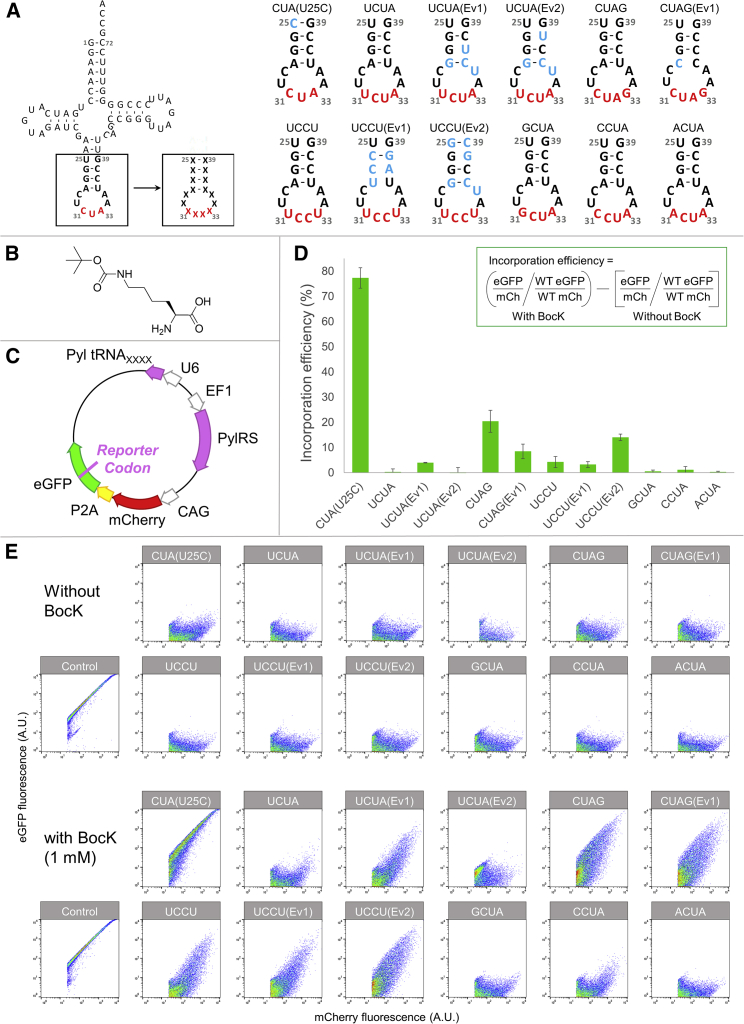
Pyl tRNA variants for quadruplet decoding in mammalian cells (A) Nucleotide sequences of tRNA variants tested; X = A, C, G, or U; red denotes the anticodon, blue denotes mutations from the wild-type Pyl tRNA sequence in *M. mazei*, black denotes bases retained from the wild-type sequence. (B) Chemical structure of BocK. (C) Schematic representation of reporters used to analyze PylRS/tRNA_XXXX_-mediated BocK incorporation into eGFP. The reporter codon encodes the 150^th^ amino acid residue of eGFP. Lack of BocK incorporation results in termination of translation and no eGFP fluorescence. (D) BocK incorporation efficiency of each tRNA_XXXX_ variant. HEK293 cells were transiently transfected with a reporter vector and incubated in the presence or absence of 1 mM BocK. Cells underwent flow-cytometry analysis and the equation displayed was used to calculate incorporation efficiency (Bartoschek et al., 2021). Means and standard deviations calculated from three biological replicates are shown. (E) Representative flow cytometry results used for calculating BocK incorporation efficiency. Events were gated for HEK293 cells and to exclude doublets. Cells were then gated to include only transfected (i.e., mCherry-positive) cells. Fluorescence intensities are given in arbitrary units (a.u.). The geometric mean of fluorescence intensity was used to calculate the incorporation efficiency. See also Figure S1 for fluorescence imaging and immunoblotting results of a single fluorescence eGFP(150XXXX) reporter.

Pyl tRNA variants that can decode UAGN (N = A/U/C/G), CUAG, or AGGA codons have been engineered for unnatural amino acid incorporation in *E. coli* (Chen et al., 2018; Niu et al., 2013; Wang et al., 2014, 2016). However, the translation machineries in *E. coli* and mammalian cells are different (Melnikov et al., 2018), thus tRNA optimized in one system might not work well in the other. In light of this, we evaluated the performance of 11 quadruplet-decoding Pyl tRNA variants in mammalian cells (Figure 2A). These tRNA variants consisted of the simple replacement of the CUA anticodon to NCUA (for decoding UAGN), CUAG (for decoding CUAG), or UCCU (for decoding AGGA), as well as previously identified tRNA variants that were evolved in *E. coli* and carry additional mutations in the anticodon stem loop. Pyl tRNA_UCUA(Ev1)_, Pyl tRNA_CUAG(Ev1)_, and Pyl tRNA_UCCU(Ev1)_ were chosen for their high decoding efficiency in *E. coli* (Wang et al., 2014), whereas Pyl tRNA_UCUA(Ev2)_ (Chen et al., 2018) and Pyl tRNA_UCCU(Ev2)_ (Niu et al., 2013) have been used for unnatural amino acid incorporation in mammalian cells.

*Nε*-Boc-L-lysine (BocK) (Figure 2B) is a substrate of the wild-type PylRS (Yanagisawa et al., 2008). To perform a systematic comparison, we constructed dual-fluorescence reporters (Figure 2C) encoding the wild-type *Methanosarcina mazei* PylRS, a Pyl tRNA variant and an mCherry-P2A-eGFP(150XXXX) reporter, where the 150^th^ amino acid residue in eGFP corresponds to a quadruplet codon. P2A is a self-cleavage sequence, leaving only a Pro residue in the C-terminal fragment after cleavage (Kim et al., 2011). In this reporter, mCherry is produced constitutively and thus serves as the transfection control. On the other hand, green fluorescence will only be observed upon successful decoding of the quadruplet codon and production of full-length eGFP. If the first three bases of a quadruplet codon are decoded as a triplet codon, this will lead to a translational frameshift and premature termination (Figure 1C). Thus, quadruplet-decoding efficiency can be calculated from the ratio between the two fluorescence intensities in the presence and absence of BocK (Bartoschek et al., 2021; Gautier et al., 2010; Monk et al., 2017; Potts et al., 2020; Schmied et al., 2014).

Experimentally, HEK293 cells were transiently transfected with a reporter vector (Figure 2C) and cultured in the presence or absence of 1 mM BocK, followed by flow cytometry analysis. Interestingly, in contrast to the observations in *E. coli* where all variants can decode the corresponding quadruplet codon (Niu et al., 2013; Wang et al., 2014, 2016), only Pyl tRNA variants decoding CUAG or AGGA appear to be functional (Figure 2D). Particularly, Pyl tRNA_CUAG_ and Pyl tRNA_UCCU(Ev2)_ outperformed the other variants, and the functional Pyl tRNA variants remained orthogonal in mammalian cells, manifesting as a lack of detectable eGFP in the absence of BocK by flow cytometry, fluorescence microscopy, or immunoblotting (Figures 2E and S1). Interestingly, a higher production of eGFP was observed with Pyl tRNA_UCCU(Ev2)_ compared with Pyl tRNA_CUAG_ when using a single fluorescence eGFP(150XXXX) reporter (Figure S1), although these findings could be due to a higher translation frequency or better transfection efficiency of the vector. Nevertheless, we chose to use tRNA_UCCU(Ev2)_ for subsequent experiments.

### Identification of a second orthogonal pair

To utilize genetic code expansion to control logic gate inputs, two orthogonal aaRS/tRNA pairs that selectively incorporate different unnatural amino acids are required. Several *E. coli* aaRS enzymes (e.g., *Ec*TyrRS) and their cognate tRNA molecules have been engineered as orthogonal pairs for amber suppression in mammalian cells to incorporate various unnatural amino acids (Italia et al., 2018; Nodling et al., 2019). As PylRS/tRNA can be used as an orthogonal pair in *E. coli*, it should remain orthogonal to engineered *Ec*TyrRS/tRNA_CUA_ pairs. To verify this, an *Ec*TyrRS variant (referred to hereafter as TyrRS∗) containing Y37V, D182S, F183M, and G265R mutations was chosen. TyrRS∗ was reported to be able to incorporate a range of unnatural amino acids, including *O*-methyl-L-tyrosine (OMeY) and 4-azido-L-phenylalanine (AzF), into proteins in mammalian cells (Chatterjee et al., 2013).

**Figure 3:**
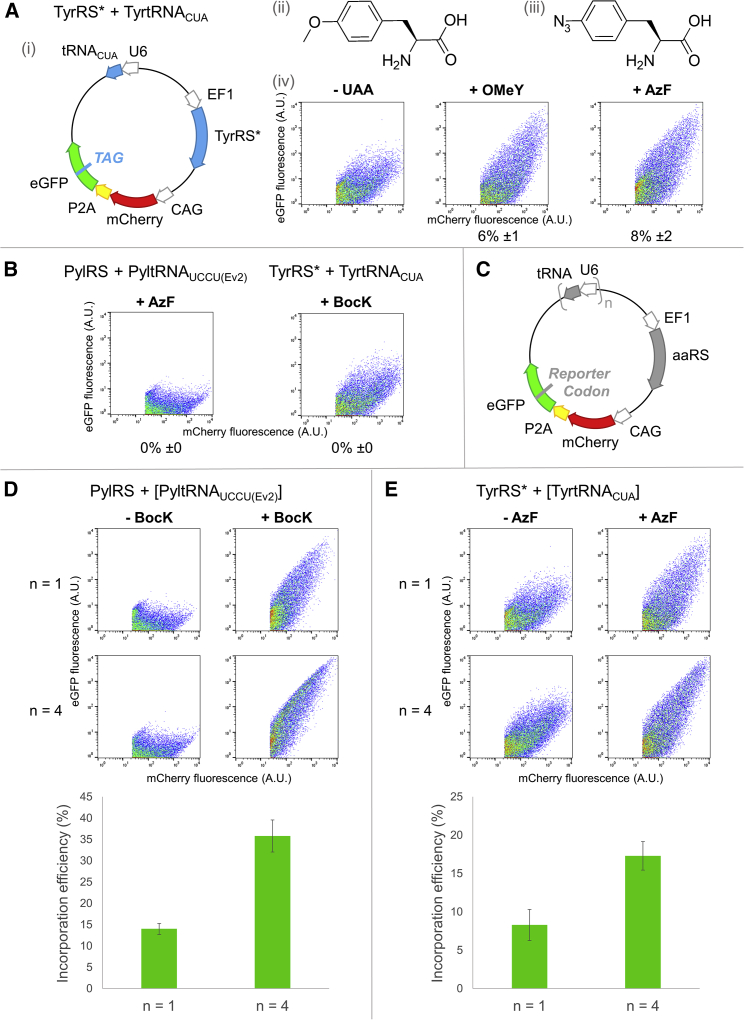
Confirmation of TyrRS∗/tRNA_CUA_ as the second orthogonal pair (A) (i) The reporter to test efficiency of TyrRS∗/tRNA_CUA_. (ii) Chemical structure of OMeY. (iii) Chemical structure of AzF. (iv) OMeY or AzF incorporation efficiency by TyrRS∗/tRNA_CUA_ in HEK293 cells by using flow cytometry. Higher eGFP fluorescence was detected with AzF supplementation. (B) Orthogonality of tRNA/aaRS pairs. HEK293 cells transfected with PylRS/tRNA_UCCU(Ev2)_ or TyrRS∗/tRNA_CUA_ reporter vector and incubated for 24 h in the presence of AzF or BocK respectively. Fluorescent eGFP was only detected when the aaRS/tRNA pairs were in the presence of their cognate unnatural amino acid depicting mutual orthogonality of the pairs. (C) Reporters for testing the impact of tRNA copy number on unnatural amino acid incorporation efficiency. (D) Comparison of Pyl tRNA_UCCU(Ev2)_ n = 1 and n = 4. (E) Comparison of Tyr tRNA_CUA_ n = 1 and n = 4. Flow cytometry was used to analyze BocK or AzF incorporation into the 150^th^ residue of eGFP. Unnatural amino acids were supplemented into the cell growth medium to a final concentration of 1 mM. Means and standard deviations calculated from three biological replicates are shown. For incorporation efficiency calculation, see Figure 2.

For systematic comparison, a reporter vector following the same design as the reporter vector for Pyl tRNA variants was generated, in which PylRS was replaced by TyrRS∗ and Pyl tRNA by Tyr tRNA_CUA_ (Figure 3A). Although TyrRS∗ was shown to incorporate both OMeY and AzF efficiently (Chatterjee et al., 2013), we observed higher incorporation efficiency of AzF than OMeY by TyrRS∗ (Figure 3A) and the observable difference seemed to be more prominent in fluorescence microscopy analysis (Figure S2A). Further, AzF incorporation by TyrRS∗ was greater compared with an *Ec*TyrRS variant (Takimoto et al., 2009) evolved specifically for AzF incorporation (Figure S2B). We thus continued with AzF and TyrRS∗/tRNA_CUA_ to confirm the orthogonality with PylRS/tRNA_UCCU(Ev2)_, and showed that the two pairs specifically recognized their respective unnatural amino acids (Figures 3B and S2C). We then transfected HEK293 cells with two vectors, one carrying only the TyrRS∗/tRNA_CUA_ machinery, and the other carrying the PylRS/tRNA_UCCU(Ev2)_ pair alongside a double-incorporation reporter eGFP(40TAG,150AGGA) that contains a TAG codon for the 40^th^ amino acid residue and an AGGA codon for the 150^th^ residue in the *eGFP* gene. For 24 h, cells were maintained under four distinct conditions: (1) no unnatural amino acid, (2) BocK only, (3) AzF only, and (4) both BocK and AzF. Fluorescence was only detected when both unnatural amino acids were present (Figure S3), confirming the orthogonality of the two pairs as well as demonstrating their use for double incorporation into the same protein.

### Optimization of unnatural amino acid incorporation

To use unnatural amino acids as logic gate inputs, their incorporation efficiency would directly affect the logic gate responses and so a higher incorporation efficiency is preferable. We first attempted to improve the incorporation efficiency by overexpression of an engineered eukaryotic release factor 1 (eRF1). With an E55D point mutation, this engineered eRF1 was shown to improve amber suppression by Pyl tRNA_CUA(U25C)_ (Li et al., 2020; Schmied et al., 2014). We therefore tested the incorporation efficiency of both aaRS/tRNA pairs here in the presence and absence of this engineered eRF1; however, no significant improvement was observed in either case (Figure S2D).

It has also been reported that increasing the copy number of orthogonal tRNA can improve the unnatural amino acid incorporation efficiency in mammalian cells (Chatterjee et al., 2013; Schmied et al., 2014). Thus, we constructed vectors carrying four copies of tRNA (n = 1 or n = 4; Figure 3C), and both PylRS/tRNA_UCCU(Ev2)_ and TyrRS∗/tRNA_CUA_ pairs demonstrated a significant increase in eGFP production (p < 0.005) (Figures 3D and 3E). Moreover, by increasing the tRNA and reporter copy numbers, a marked increase in eGFP production was also observed in the double-incorporation experiment, as demonstrated by fluorescence microscopy and immunoblotting (Figure S3).

### Construction of logic gates controlled by two unnatural amino acids

**Figure 4:**
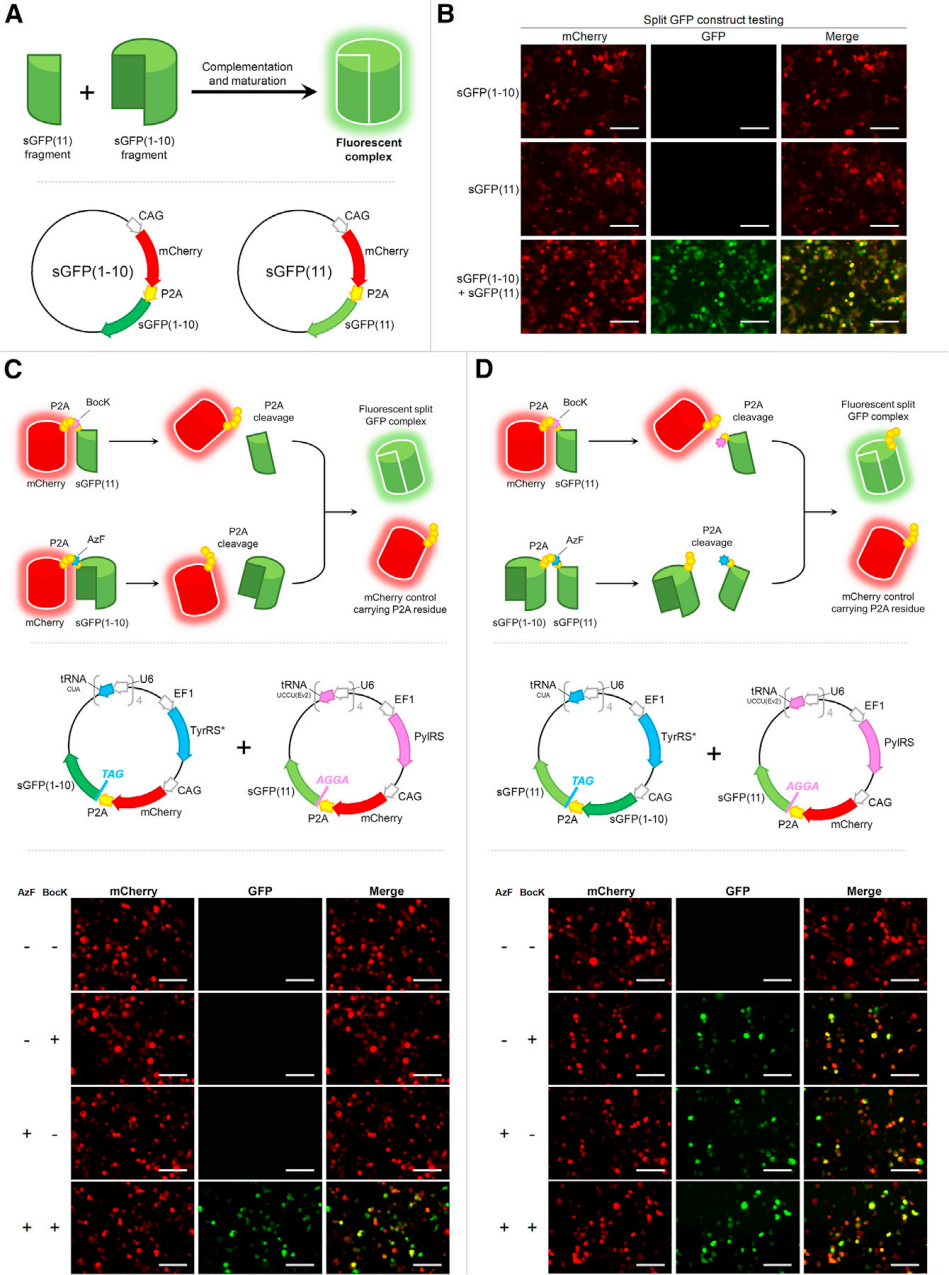
Exploiting genetic code expansion for AND and OR mammalian cell logic gates (A) Principles of how split GFP works and vector designs used for testing the core design for logic operations. (B) Fluorescent microscope imaging of HEK293 transfected with either or both vectors depicted in (A), GFP fluorescence was only detected when both fragments were present; mCherry expression was equal across all conditions. (C and D) Shown are (C) AND logic gate and (D) OR logic gate controlled by BocK and AzF. Graphical depiction of principle approach is depicted at the top panel. Vector designs are shown in the middle. Fluorescence imaging results are shown at the bottom panel. HEK293 cells were transfected with the two vectors and incubated under four different conditions: no unnatural amino acid, BocK only, AzF only, or BocK and AzF. mCherry fluorescence indicated transfection efficiency and was at similar intensity across all four conditions. GFP fluorescence represents the logic operation output. All images are 223 μm × 167 μm, scale bars denote 50 μm. All unnatural amino acids used were supplemented into the cell growth medium to obtain a final concentration of 1 mM. See also Figure S4.

For proof of concept, we then applied PylRS/tRNA_UCCU(Ev2)_ and TyrRS∗/tRNA_CUA_ pairs to construct logic gates controlled by two unnatural amino acids, BocK and AzF. The logic gates were designed by using a split GFP system, which requires the concurrent presence of two complementary polypeptides sGFP(1–10) and sGFP(11) to form a fluorescent complex (Cabantous et al., 2005; Kamiyama et al., 2016). When produced individually, the GFP chromophore is unable to assemble, and no fluorescence can be detected (Figures 4A and 4B). In short, the designed logic gates have two inputs (i.e., BocK and AzF) and one output (i.e., GFP fluorescence). To test them, we created a two-vector system based on the dual-fluorescence reporters (Figures 2 and 3) whereby there is an N-terminal mCherry leading to the sGFP fragments via a P2A sequence (Figures 4A and 4B). These vectors would hence allow for control of sGFP fragment production via selected introduction of TAG or AGGA codon upstream of the sGFP sequences.

To generate a logic gate that performs AND operation, we inserted TAG and AGGA codons into the reporter genes before the sequences encoding sGFP(1–10) and sGFP(11), respectively (Figure 4C). In this case, production of sGFP(1–10) and sGFP(11) became strictly dependent on the presence of AzF and BocK concurrently, and only in the presence of both AzF and BocK can the fluorescent complex form.

HEK293 cells were transiently transfected with two vectors (Figure 4C). One vector encoded the TyrRS∗/tRNA_CUA_ pair and mCherry-P2A-TAG-sGFP(1–10), whereas the other vector encodes PylRS/tRNA_UCCU(Ev2)_ and mCherry-P2A-AGGA-sGFP(11). As expected, cells with green fluorescence were only observed in the presence of both AzF and BocK, and the level of GFP fluorescence indicates a promising efficiency of the operation.

Using a similar concept, we constructed an OR logic gate (Figure 4D). In this case, sGFP(1–10) was constantly produced, but sGFP(11) was only generated in the presence of either AzF, BocK, or both. Thus, green fluorescence is observed when at least one unnatural amino acid is present, and again the GFP fluorescence observed indicates a promising operational efficiency.

Finally, to confirm the specificity of BocK/AzF dependence and continuation of orthogonality, the vectors used for logic testing were individually transfected into HEK293 cells and incubated in the presence or absence of their respective unnatural amino acids. Upon imaging, no green fluorescence was detected regardless of unnatural amino acid supplementation for AND logic vectors (Figure S4). Indeed, green fluorescence was only detected with the OR logic vector containing TyrRS∗/tRNA_CUA_ and in the presence of AzF.

## Discussion

Here, we have demonstrated the applications of genetic code expansion for developing novel mammalian cell logic gates. We quantified the quadruplet-decoding efficiency of 11 Pyl tRNA variants in HEK293 cells. Unlike most other aaRS enzymes, PylRS does not recognize the anticodon stem loop on the Pyl tRNA during aminoacylation (Suzuki et al., 2017; Tharp et al., 2018), so alteration of the anticodon is theoretically tolerated by the synthetase. Nevertheless, whereas change of the anticodon from CUA to CUAG or UCCU was tolerated, Pyl tRNA variants with the anticodon NCUA did not seem to be functional in mammalian cells (Figures 2 and S1). In addition, although variation in the nucleic acid sequence of the anticodon stem loop can significantly affect the incorporation efficiency, we found that tRNA sequences optimized in *E. coli* (Niu et al., 2013; Wang et al., 2014, 2016) might not necessarily work well in mammalian cells. For example, Pyl tRNA_CUAG_ outperformed Pyl tRNA_CUAG(Ev1)_.

Successful incorporation of an unnatural amino acid involves four steps: (1) cellular uptake of the designated unnatural amino acid; (2) aminoacylation of the orthogonal tRNA by the orthogonal synthetase; (3) formation of a ternary complex containing aminoacylated tRNA, eEF1A (or EF-Tu in bacteria), and GTP; and (4) interaction of the ternary complex with the ribosome, followed by peptide bond formation. The high incorporation efficiency of BocK by Pyl tRNA_CUA(U25C)_ indicates that BocK can be readily taken up by mammalian cells. Additionally, all tested Pyl tRNA variants should be able to be aminoacylated by PylRS given that all variants were reported to be functional in *E. coli*. Thus, the differences in the prokaryotic and eukaryotic translational machinery (Melnikov et al., 2018) are likely the cause of the observed discrepancy in decoding efficiency, although it remains elusive whether formation of the ternary complex or interaction of the ternary complex with the ribosome is the main cause.

Intriguingly, negligible eGFP production was observed with Pyl tRNA_UCUA_ and Pyl tRNA_UCUA(Ev2)_. These two variants were reported to be functional in mammalian cells (Chen et al., 2018), although under different experimental conditions. Thus, the discrepancy might result from the choice of the cell line (HEK293 versus HEK293T), BocK concentration (1 mM versus 5 mM), unnatural amino acid incorporation site (40^th^ versus 150^th^ residue in eGFP), or a combination of these factors.

To construct double-input logic gates, another orthogonal pair, in addition to PylRS/tRNA_UCCU(Ev2)_, is required. We chose the *E. coli*-derived TyrRS∗/tRNA_CUA_ as it had been optimized for incorporating a range of unnatural amino acids in mammalian cells (Chatterjee et al., 2013). Given that PylRS/tRNA can be used as an orthogonal pair in *E. coli*, it should remain orthogonal to TyrRS∗/tRNA_CUA_ in mammalian cells. Indeed, each pair specifically recognized their respective unnatural amino acids (Figure 3B). Furthermore, they can function orthogonally in the same cell, as demonstrated in the double-incorporation experiment (Figure S3). It is noteworthy that PylRS and TyrRS∗ can mediate incorporation of different unnatural amino acids, including cyclopropene lysine and propargyl tyrosine (Nodling et al., 2019) for bioorthogonal labeling, so the two orthogonal pairs can further be used for dual modification of proteins.

To use the two pairs in controlling logic gate inputs, higher incorporation efficiency of the two employed unnatural amino acids is ideal. Co-expression of an engineered eRF1 was previously shown to increase the unnatural amino acid incorporation efficiency by PylRS/tRNA_CUA(U25C)_ in mammalian cells (Schmied et al., 2014). However, co-expression of the engineered eRF1 did not appear to improve AzF incorporation by TyrRS∗/tRNA_CUA_ or BocK incorporation by PylRS/tRNA_UCCU(Ev2)_ (Figure S2D). This discrepancy could be due to the difference in the employed orthogonal pairs. In addition, the beneficial effect was most prominent with genes containing multiple UAG codons in the original study (Schmied et al., 2014), whereas we only used single UAG or AGGA codons in our study. Nevertheless, improvement in AzF and BocK incorporation was observed when increasing the tRNA copy number from one to four (Figures 3D and 3E), a strategy that has been shown to work previously (Chatterjee et al., 2013; Schmied et al., 2014)

Finally, we employed the two orthogonal aaRS/tRNA pairs to construct logic gates and demonstrated selective AND and OR logic operations dependent on AzF and BocK supplementation. These designs demonstrate that unnatural amino acids can be used as molecular switches in synthetic biology logic gates. The use of biologically inert unnatural amino acids (Chen et al., 2017; Han et al., 2017; Krogager et al., 2018; Liu et al., 2017; Suzuki et al., 2018) is ideal for constructing orthogonal, artificial pathways for synthetic biological applications with no identifiable side effects or non-specific activities. Moreover, the wide substrate scope of PylRS and TyrRS∗ (Nodling et al., 2019) enables fine-tuning the logic gate response for different applications.

To the best of our knowledge, this is the first example of unnatural amino acid-based logic gates in mammalian cells. Further exploration of quadruplet-decoding orthogonal tRNA in eukaryotic cells, as well as the underlying mechanism for their different performance in bacterial and eukaryotic systems, will provide new opportunities and applications for genetic code expansion. This will facilitate not only our understanding of protein structure and function but also the discovery and development of new diagnostics and therapeutics to target human diseases.

### Significance

Mammalian cell logic gates remain heavily limited by the current approaches that involve the use of drug(-like) molecules that carry undesirable biological activities. In light of this, here we developed an alternative approach to mammalian cell logic gates via the simple process of genetic code expansion. By repurposing the cell translational machinery, we demonstrated the use of biologically inert unnatural amino acids as input switch molecules for effective and efficient mammalian cell logic operations, enabling selective and intrinsic control. The results shown here simultaneously expand the scope of genetic code expansion, while providing an exciting approach to mammalian cell logic gates.

### Limitations of the study

Although this study expands the scope of mammalian cell logic circuits, it is important to note that this investigation employed human embryonic kidney (HEK293) cells and the tRNA variants might perform differently in other cell lines. Additionally, as with all genetic code expansion applications, amber and quadruplet suppression might not be limited to the gene of interest and can potentially incorporate unnatural amino acids into other applicable codons in the host genome.

## STAR★Methods

### Key resources table

**Table:** 

REAGENT or RESOURCE	SOURCE	IDENTIFIER
**Antibodies**		

α-eGFP	ThermoFisher	#MA1-952; RRID:AB_889471
α-mouse	ThermoFisher	#32430; RRID:AB_1185566
α-GAPDH	ThermoFisher	#MA5-15738; RRID:AB_10977387
α-FLAG	Sigma	#F3165; RRID:AB_259529

**Bacterial and virus strains**		

*Escherichia coli* OneShot Stbl3	ThermoFisher	Cat#C737303

**Chemicals, peptides, and recombinant proteins**		

BocK	Fluorochem	Cat#078520
AzF	Bachem	Cat#4020250
OMeY	Alfa Aesar	H63096

**Deposited data**		

Biological triplicates of flow cytometry results (∗.fcs) available in depository	This paper	https://doi.org/10.17035/d.2021.0135747960

**Experimental models: Cell lines**		

HEK293	ECACC General Collection	Cat#85120602; RRID:CVCL_0045

**Oligonucleotides**		

See Table S1 in the supplemental information	Merck Life Sciences	VC00021

**Recombinant DNA**		

See vector cloning section below and the supplemental information for vector sequences STAR methods	This paper	n/a
Vector 58: Pyl tRNA_CUA(U25C)_ PylRS mCherry-P2A-eGFP(150TAG)	This paper	Addgene Plasmid #174526
Vector 62: Pyl tRNA_CUAG_ PylRS mCherry-P2A-eGFP(150CTAG)	This paper	Addgene Plasmid #174527
Vector 66: Pyl tRNA_UCCU(Ev2)_ PylRS mCherry-P2A-eGFP(150AGGA)	This paper	Addgene Plasmid #174528
Vector 73: 4x(Pyl tRNA_UCCU(Ev2)_) PylRS mCherry-P2A-eGFP(150AGGA)	This paper	Addgene Plasmid #174529

**Software and algorithms**		

FlowJo	BD Biosciences	RRID:SCR_008520
ZEN	Zeiss	RRID:SCR_013672
Image Lab	Bio-Rad	RRID:SCR_014210

**Other**		

Dulbecco’s Modified Eagle Medium with GlutaMAX supplement	Fisher Scientific	#11574516
Fetal bovine serum	Fisher Scientific	#11573397
Trypsin-EDTA (0.25%)	Fisher Scientific	#11560626
Dulbecco’s Phosphate Buffered Saline	Sigma	#D1408
Lipofectamine 2000	Life Technologies	#10696343
Opti-MEM	Fisher Scientific	#11058021

### Resource availability

#### Lead contact

Further information and requests for resources and reagents should be directed to and will be fulfilled by the lead contact, Yu-Hsuan Tsai (Tsai.Y-H@outlook.com).

#### Materials availability

Full DNA sequences for each vector used in the main manuscript can be found in the Supporting Information. Plasmids generated in this study have been deposited to Addgene: 174526 for vector 58 – Pyl tRNA_CUA(U25C)_ PylRS mCherry-P2A-eGFP(150TAG); 174527 for vector 62 – Pyl tRNA_CUAG_ PylRS mCherry-P2A-eGFP(150CTAG); 174528 for vector 66 – Pyl tRNA_UCCU(Ev2)_ PylRS mCherry-P2A-eGFP(150AGGA); 174529 for vector 73 – 4x(Pyl tRNA_UCCU(Ev2)_) PylRS mCherry-P2A-eGFP(150AGGA).

### Experimental model and subject details

HEK293 cells were obtained from ECACC (ECACC General Collection, #85120602) and routinely tested for mycoplasma infection. Cells were maintained in T75 flasks at 37°C in a 5% CO_2_ high-humidity atmosphere in Dulbecco’s Modified Eagle Medium (DMEM) with GlutaMAX supplement (Fisher Scientific, #11574516) supplemented with 10% (v/v) fetal bovine serum (FBS; Fisher Scientific, #11573397). Cells were maintained at a sub-confluent monolayer and split at 80-85% confluency. For splitting, cells were washed with Dulbecco’s phosphate buffered saline (DPBS; Sigma, #D1408), detached in 1 mL of 0.25% trypsin-EDTA (Fisher Scientific, #11560626) and 200 μL of the 1000 μL trypsin cell suspension was re-suspended in 12 mL fresh DMEM containing 10% (v/v) FBS in a new T75 flask.

### Method details

#### Vector cloning

All Vector stocks were generated and maintained via transformation of chemically competent One Shot™ Stbl3™ *E. coli* cells from ThermoFisher (ThermoFisher, #C737303) and isolated from 5 mL cultures using QIAprep Spin Miniprep Kit (Qiagen, #27106) following the manufacturer’s protocol. Where appropriate, restriction digests were conducted using FastDigest restriction enzymes (Thermo Scientific) following manufacturer’s protocol. PCR was conducted in a thermocycler using PrimeSTAR Max (Takara, #R045A) following the manufacturer’s protocol. Restriction digests and PCR products were electrophoresed on 1% agarose gels in TAE buffer (40 mM Tris pH 7.6, 20 mM acetic acid, 1 mM EDTA) and visualised using SYBRSafe DNA stain (Invitrogen, #S33102). Desired bands were excised from the gel and extracted using QIAquick Gel Extraction Kit (Qiagen, #28704) following the manufacturer’s protocol. T4 ligations were performed using T4 DNA Ligase (ThermoFisher, #EL0014) and Gibson assembly via NEBuilder HiFi DNA Assembly (New England Biolabs, #E5520) following respective manufacturers protocols. All constructs were confirmed via Sanger sequencing. A list of primers used in this study is provided in Table S1.

##### Vector 1: Pyl tRNA_CUA(U25C)_

This Vector was purchased from GeneArt (ThermoFisher). The full DNA sequence can be found in the supplemental information.

##### Vector 2: 4xPyl tRNA_CUA(U25C)_-PylRS-eGFP(150TAG)

This Vector is pEF1α-FLAGPylRS-CAG-eGFP(150TAG)-4xU6-PylTU25C (Ref: 10.1038/s41598-018-28178-3).

##### Vector 3: Pyl tRNA_CUA(U25C)_-PylRS-eGFP(150TAG)

**Vector 2** was cut with the restriction enzyme Eco147i to generate the vector. Insert generated via PCR of **Vector 2** using **Primer 1** and **Primer 2**. The vector and insert were combined using Gibson Assembly.

##### Vector 4: pCAG-eGFP

This Vector is pCAG-eGFP (Ref: Perry, A. C. F. *et al.* Mammalian transgenesis by intracytoplasmic sperm injection. *Science* **284**, 1180–1183 (1999).)

##### Vector 5: eGFP(150TAGA)

Cloned by PCR mutagenesis of **Vector 4** using **Primer 3** and **Primer 4**. Template **Vector 4** removed from reaction mixture via DpnI restriction enzyme digest.

##### Vector 6: eGFP(150CTAG)

Cloned by PCR mutagenesis of **Vector 4** using **Primer 3** and **Primer 5**. Template **Vector 4** removed from reaction mixture via DpnI restriction enzyme digest.

##### Vector 7: eGFP(150AGGA)

Cloned by PCR mutagenesis of **Vector 4** using **Primer 3** and **Primer 6**. Template **Vector 4** removed from reaction mixture via DpnI restriction enzyme digest.

##### Vector 8: eGFP(150TAGT)

Cloned by PCR mutagenesis of **Vector 4** using **Primer 3** and **Primer 7**. Template **Vector 4** removed from reaction mixture via DpnI restriction enzyme digest.

##### Vector 9: eGFP(150TAGC)

Cloned by PCR mutagenesis of **Vector 4** using **Primer 3** and **Primer 8**. Template **Vector 4** removed from reaction mixture via DpnI restriction enzyme digest.

##### Vector 10: eGFP(150TAGG)

Cloned by PCR mutagenesis of **Vector 4** using **Primer 3** and **Primer 9**. Template **Vector 4** removed from reaction mixture via DpnI restriction enzyme digest.

##### Vector 11: Pyl tRNA_CUA(U25C)_-PylRS-eGFP(150TAGA)

Vector generated via digestion of **Vector 3** with the restriction enzymes BglII and XbaI. Insert generated via digestion of **Vector 5** with BglII and XbaI. The vector and insert fragments were assembled by T4 ligation.

##### Vector 12: Pyl tRNA_CUA(U25C)_-PylRS-eGFP(150CTAG)

Vector generated via digestion of **Vector 3** with restriction enzymes BglII and XbaI. Insert generated via digestion of **Vector 6** with BglII and XbaI. The vector and insert fragments were assembled by T4 ligation.

##### Vector 13: Pyl tRNA_CUA(U25C)_-PylRS-eGFP(150AGGA)

Vector generated via digestion of **Vector 3** with restriction enzymes BglII and XbaI. Insert generated via digestion of **Vector 7** with BglII and XbaI. The vector and insert fragments were assembled by T4 ligation.

##### Vector 14: Pyl tRNA_CUA(U25C)_-PylRS-eGFP(150TAGT)

Vector generated via digestion of **Vector 3** with the restriction enzymes BglII and XbaI. Insert generated via digestion of **Vector 8** with BglII and XbaI. The vector and insert fragments were assembled by T4 ligation.

##### Vector 15: Pyl tRNA_CUA(U25C)_-PylRS-eGFP(150TAGC)

Vector generated via digestion of **Vector 3** with the restriction enzymes BglII and XbaI. Insert generated via digestion of **Vector 9** with BglII and XbaI. The vector and insert fragments were assembled by T4 ligation.

##### Vector 16: Pyl tRNA_CUA(U25C)_-PylRS-eGFP(150TAGT)

Vector generated via digestion of **Vector 3** with the restriction enzymes BglII and XbaI. Insert generated via digestion of **Vector 10** with BglII and XbaI. The vector and insert fragments were assembled by T4 ligation.

##### Vector 17: Pyl tRNA_UCUA_

Cloned by PCR mutagenesis of **Vector 1** using **Primer 10** and **Primer 11**. The template **Vector 1** was removed from the reaction mixture via DpnI restriction enzyme digest.

##### Vector 18: Pyl tRNA_UCUA(Ev1)_

Cloned by PCR mutagenesis of **Vector 1** using **Primer 10** and **Primer 12**. The template **Vector 1** was removed from the reaction mixture via DpnI restriction enzyme digest.

##### Vector 19: Pyl tRNA_UCUA(Ev2)_

Cloned by PCR mutagenesis of **Vector 1** using **Primer 10** and **Primer 13**. The template **Vector 1** was removed from the reaction mixture via DpnI restriction enzyme digest.

##### Vector 20: Pyl tRNA_CUAG_

Cloned by PCR mutagenesis of **Vector 1** using **Primer 10** and **Primer 14**. The template **Vector 1** was removed from the reaction mixture via DpnI restriction enzyme digest.

##### Vector 21: Pyl tRNA_CUAG(Ev1)_

Cloned by PCR mutagenesis of **Vector 1** using **Primer 10** and **Primer 15**. The template **Vector 1** was removed from the reaction mixture via DpnI restriction enzyme digest.

##### Vector 22: Pyl tRNA_UCCU_

Cloned by PCR mutagenesis of **Vector 1** using **Primer 10** and **Primer 16**. The template **Vector 1** was removed from the reaction mixture via DpnI restriction enzyme digest.

##### Vector 23: Pyl tRNA_UCCU(Ev1)_

Cloned by PCR mutagenesis of **Vector 1** using **Primer 10** and **Primer 17**. The template **Vector 1** was removed from the reaction mixture via DpnI restriction enzyme digest.

##### Vector 24: Pyl tRNA_UCCU(Ev2)_

Cloned by PCR mutagenesis of **Vector 1** using **Primer 10** and **Primer 18**. The template **Vector 1** was removed from the reaction mixture via DpnI restriction enzyme digest.

##### Vector 25: Pyl tRNA_ACUA_

Cloned by PCR mutagenesis of **Vector 1** using **Primer 10** and **Primer 19**. The template **Vector 1** was removed from the reaction mixture via DpnI restriction enzyme digest.

##### Vector 26: Pyl tRNA_GCUA_

Cloned by PCR mutagenesis of **Vector 1** using **Primer 10** and **Primer 20**. The template **Vector 1** was removed from the reaction mixture via DpnI restriction enzyme digest.

##### Vector 27: Pyl tRNA_CCUA_

Cloned by PCR mutagenesis of **Vector 1** using **Primer 10** and **Primer 21**. The template **Vector 1** was removed from the reaction mixture via DpnI restriction enzyme digest.

##### Vector 28: Pyl tRNA_UCUA_-PylRS-eGFP(150TAGA)

Vector generated by digesting **Vector 11** with the restriction enzymes Eco147i and AgeI. Insert generated from restriction digestion of **Vector 17** using Eco147i and AgeI. The vector and insert fragments were assembled by T4 ligation.

##### Vector 29: Pyl tRNA_UCUA(Ev1)_-PylRS-eGFP(150TAGA)

Vector generated by digesting **Vector 11** with the restriction enzymes Eco147i and AgeI. Insert generated from restriction digestion of **Vector 18** using Eco147i and AgeI. The vector and insert fragments were assembled by T4 ligation.

##### Vector 30: Pyl tRNA_UCUA(Ev2)_-PylRS-eGFP(150TAGA)

Vector generated by digesting **Vector 11** with the restriction enzymes Eco147i and AgeI. Insert generated from restriction digestion of **Vector 19** using Eco147i and AgeI. The vector and insert fragments were assembled by T4 ligation.

##### Vector 31: Pyl tRNA_CUAG_-PylRS-eGFP(150CTAG)

Vector generated by digesting **Vector 12** with the restriction enzymes Eco147i and AgeI. Insert generated from restriction digestion of **Vector 20** using Eco147i and AgeI. The vector and insert fragments were assembled by T4 ligation.

##### Vector 32: Pyl tRNA_CUAG(Ev1)_-PylRS-eGFP(150CTAG)

Vector generated by digesting **Vector 12** with the restriction enzymes Eco147i and AgeI. Insert generated from restriction digestion of **Vector 21** using Eco147i and AgeI. The vector and insert fragments were assembled by T4 ligation.

##### Vector 33: Pyl tRNA_UCCU_-PylRS-eGFP(150AGGA)

Vector generated by digesting **Vector 13** with the restriction enzymes Eco147i and AgeI. Insert generated from restriction digestion of **Vector 22** using Eco147i and AgeI. The vector and insert fragments were assembled by T4 ligation.

##### Vector 34: Pyl tRNA_UCCU(Ev1)_-PylRS-eGFP(150AGGA)

Vector generated by digestion of **Vector 13** with restriction enzymes Eco147i and AgeI. Insert generated from restriction digestion of **Vector 23** using Eco147i and AgeI. The vector and insert fragments were assembled by T4 ligation.

##### Vector 35: Pyl tRNA_UCCU(Ev2)_-PylRS-eGFP(150AGGA)

Vector generated by digesting **Vector 13** with restriction enzymes Eco147i and AgeI. Insert generated from restriction digestion of **Vector 24** using Eco147i and AgeI. The vector and insert fragments were assembled by T4 ligation.

##### Vector 36: Pyl tRNA_ACUA_-PylRS-eGFP(150TAGT)

Vector generated by digesting **Vector 14** with restriction enzymes Eco147i and AgeI. Insert generated from restriction digestion of **Vector 25** using Eco147i and AgeI. The vector and insert fragments were assembled by T4 ligation.

##### Vector 37: Pyl tRNA_GCUA_-PylRS-eGFP(150TAGC)

Vector generated by digesting **Vector 15** with restriction enzymes Eco147i and AgeI. Insert generated from restriction digestion of **Vector 26** using Eco147i and AgeI. The vector and insert fragments were assembled by T4 ligation.

##### Vector 38: Pyl tRNA_CCUA_-PylRS-eGFP(150TAGG)

Vector generated by digesting **Vector 16** with restriction enzymes Eco147i and AgeI. Insert generated from restriction digestion of **Vector 27** using Eco147i and AgeI. The vector and insert fragments were assembled by T4 ligation.

##### Vector 39: (U6-Tyr tRNA_CUA_)x2(H1-Tyr tRNA_CUA_)x2 TyrRS∗-eGFP(40TAG)

This Vector was purchased from Addgene (#50831).

##### Vector 40: Tyr tRNA_CUA_-PylRS-eGFP(150TAG)

Vector generated by digesting **Vector 3** with restriction enzymes Eco147i and AgeI. Insert generated via PCR of **Vector 39** using **Primer 22** and **Primer 23**. The vector and insert were combined using Gibson assembly.

##### Vector 41: Tyr tRNA_CUA_-TyrRS∗-eGFP(150TAG)

Vector generated by digesting **Vector 40** with restriction enzymes NheI and BamHI. Insert generated via PCR of **Vector 39** using **Primer 24** and **Primer 25**. The vector and insert were combined using Gibson assembly.

##### Vector 42: (Pyl tRNA_UCCU (Ev2)_)x4-PylRS-eGFP(150AGGA)

Vector generated by digesting **Vector 35** with Eco147i restriction enzyme. Thermosensitive Alkaline Phosphotase (TAP) was included in reaction mixture to remove 5’ and 3’ phosphate groups and prevent re-ligation. The three inserts were generated via PCR of **Vector 35** using three distinctive pairs of primers: **Primer 26** and **Primer 27**; **Primer 28** and **Primer 29**; and **Primer 30** and **Primer 31**. The vector and inserts were combined using Gibson assembly.

##### Vector 43: (Tyr tRNA_CUA_)x4-TyrRS∗-eGFP(150TAG)

Vector generated by digesting **Vector 41** with Eco147i restriction enzyme. Thermosensitive Alkaline Phosphotase (TAP) was included in reaction mixture to remove 5’ and 3’ phosphate groups and prevent re-ligation. The three inserts were generated via PCR of **Vector 41** using three distinctive pairs of primers: **Primer 32** and **Primer 33**; **Primer 34** and **Primer 35**; and **Primer 36** and **Primer 37**. The vector and inserts were combined using Gibson assembly.

##### Vector 44: eRF1(E55D)

This was a kind gift from Jason Chin (Schmied et al., 2014).

##### Vector 45: eGFP(40TAG,150AGGA)

Vector generated by PCR of **Vector 7** using **Primer 38** and **Primer 39**. Insert generated by PCR of **Vector 7** using **Primer 40** and **Primer 41**. The vector and insert were combined using Gibson assembly.

##### Vector 46: Pyl tRNA_UCCU(Ev2)_-PylRS-eGFP(40TAG,150AGGA)

Vector generated by digesting **Vector 35** using restriction enzymes BglII and EcoRI. Insert generated by restriction digest of **Vector 45** using BglII and EcoRI. Both the vector and insert isolated via TAE agarose gel electrophoresis and gel extraction. The vector and insert fragments were assembled by T4 ligation.

##### Vector 47: Tyr tRNA_CUA_-TyrRS∗

Vector generated by digesting **Vector 41** with dual cutter restriction enzyme EcoRI and isolating larger fragment. The DNA was re-ligated via T4 ligation.

##### Vector 48: (Pyl tRNA_UCCU(Ev2)_)x4-PylRS-eGFP(40TAG,150AGGA)

Vector generated by digesting **Vector 42** using restriction enzymes BglII and XbaI. Insert generated by restriction digest of **Vector 45** using BglII and XbaI. The vector and insert fragments were assembled by T4 ligation.

##### Vector 49: (Tyr tRNA_CUA_)x4-TyrRS∗-eGFP(40TAG,150AGGA)

Vector generated by digesting **Vector 43** using restriction enzymes BglII and XbaI. Insert generated by restriction digest of **Vector 45** using BglII and XbaI. The vector and insert fragments were assembled by T4 ligation.

##### Vector 50: mCherry

This was constructed from pCAG-mCherry.

##### Vector 51: mCherry-P2A-sGFP(1-10)

Vector generated by digesting **Vector 50** with restriction enzymes BsrGI and BglII. Insert composed a gene strings fragment encoding C-terminal residues of mCherry, P2A linker and sfGFP(1-10) and was ordered from GeneArt (ThermoFisher). Vector and insert combined via Gibson assembly.

##### Vector 52: sGFP(11)

This Vector was purchased from GeneArt (ThermoFisher).

##### Vector 53: mCherry-P2A-sGFP(11)

Vector generated by digesting **Vector 51** with MluI and BglII. Insert generated by digestion of **Vector 52** with MluI and BglII. Vector and insert were assembled via T4 ligation.

##### Vector 54: mCherry-P2A-TAG-sGFP(1-10)

Vector generated by digesting **Vector 51** with PshAI and BglII. Insert generated by PCR of **Vector 51** with **Primer 42** and **Primer 43**. The vector and insert were combined using Gibson assembly.

##### Vector 55: (Tyr tRNA_CUA_)x4-TyrRS∗-mCherry-P2A-TAG-sGFP(1-10)

Vector generated by restriction digestion of **Vector 43** using BglII and XbaI. Insert generated by restriction digest of **Vector 54** with BglII and XbaI. The vector and insert were combined using T4 ligation.

##### Vector 56: (Pyl tRNA_UCCU(Ev2)_)x4-PylRS-mCherry-P2A-AGGA-sGFP(11)

Vector generated by restriction digest of **Vector 42** with XbaI and BglII. Insert 1 generated by restriction digest of **Vector 51** with PshAI and XbaI. Insert 2 generated by PCR of **Vector 53** using **Primer 44** and **Primer 45**. Insert 2 subjected to restriction digestion with PshAI and BglII to generate sticky ends. The three fragments were combined by T4 ligation.

##### Vector 57: (Tyr tRNA_CUA_)x4-TyrRS∗-sGFP(1-10)-P2A-TAG-sGFP(11)

Vector generated by digesting **Vector 43** with XbaI and BglII. Insert 1 generated by PCR of **Vector 53** with **Primer 46** and **Primer 47**. Insert 2 generated by PCR of **Vector 51** with **Primer 48** and **Primer 49**. The three fragments were combined via Gibson Assembly.

##### Vector 58: Pyl tRNA_CUA(U25C)_-PylRS-mCherry-P2A-eGFP(150TAG)

**Vector 3** digested with NheI and BamHI to generate the vector. Insert 1 generated by PCR of **Vector 3** with **Primer 50** and **Primer 51**. Insert 2 generated by PCR of **Vector 51** with **Primer 52** and **Primer 53**. The three fragments were combined via Gibson assembly.

##### Vector 59: Pyl tRNA_UCUA_-PylRS-mCherry-P2A-eGFP(150TAGA)

**Vector 28** digested with EcoRI and BglII to generate vector. Insert 1 generated by PCR of **Vector 28** with **Primer 50** and **Primer 51.** Insert 2 generated by PCR of **Vector 51** with **Primer 52** and **Primer 53**. The three fragments were combined via Gibson assembly.

##### Vector 60: Pyl tRNA_UCUA(Ev1)_-PylRS-mCherry-P2A-eGFP(150TAGA)

**Vector 29** digested with EcoRI and BglII to generate vector. Insert 1 generated by PCR of **Vector 29** with **Primer 50** and **Primer 51.** Insert 2 generated by PCR of **Vector 51** with **Primer 52** and **Primer 53**. The three fragments were combined via Gibson assembly.

##### Vector 61: Pyl tRNA_UCUA(Ev2)_-PylRS-mCherry-P2A-eGFP(150TAGA)

**Vector 30** digested with EcoRI and BglII to generate vector. Insert 1 generated by PCR of **Vector 30** with **Primer 50** and **Primer 51.** Insert 2 generated by PCR of **Vector 51** with **Primer 52** and **Primer 53**. The three fragments were combined via Gibson assembly.

##### Vector 62: Pyl tRNA_CUAG_-PylRS-mCherry-P2A-eGFP(150CTAG)

**Vector 31** digested with EcoRI and BglII to generate vector. Insert 1 generated by PCR of **Vector 31** with **Primer 50** and **Primer 51.** Insert 2 generated by PCR of **Vector 51** with **Primer 52** and **Primer 53**. The three fragments were combined via Gibson assembly.

##### Vector 63: Pyl tRNA_CUAG(Ev1)_-PylRS-mCherry-P2A-eGFP(150CTAG)

**Vector 32** digested with EcoRI and BglII to generate vector. Insert 1 generated by PCR of **Vector 32** with **Primer 50** and **Primer 51.** Insert 2 generated by PCR of **Vector 51** with **Primer 52** and **Primer 53**. The three fragments were combined via Gibson assembly.

##### Vector 64: Pyl tRNA_UCCU_-PylRS-mCherry-P2A-eGFP(150AGGA)

**Vector 33** digested with EcoRI and BglII to generate vector. Insert 1 generated by PCR of **Vector 33** with **Primer 50** and **Primer 51.** Insert 2 generated by PCR of **Vector 51** with **Primer 52** and **Primer 53**. The three fragments were combined via Gibson assembly.

##### Vector 65: Pyl tRNA_UCCU(Ev1)_-PylRS-mCherry-P2A-eGFP(150AGGA)

**Vector 34** digested with EcoRI and BglII to generate vector. Insert 1 generated by PCR of **Vector 34** with **Primer 50** and **Primer 51.** Insert 2 generated by PCR of **Vector 51** with **Primer 52** and **Primer 53**. The three fragments were combined via Gibson assembly.

##### Vector 66: Pyl tRNA_UCCU(Ev2)_-PylRS-mCherry-P2A-eGFP(150AGGA)

**Vector 35** digested with EcoRI and BglII to generate vector. Insert 1 generated by PCR of **Vector 35** with **Primer 50** and **Primer 51.** Insert 2 generated by PCR of **Vector 51** with **Primer 52** and **Primer 53**. The three fragments were combined via Gibson assembly.

##### Vector 67: Pyl tRNA_ACUA_-PylRS-mCherry-P2A-eGFP(150TAGT)

**Vector 36** digested with EcoRI and BglII to generate vector. Insert 1 generated by PCR of **Vector 36** with **Primer 50** and **Primer 51.** Insert 2 generated by PCR of **Vector 51** with **Primer 52** and **Primer 53**. The three fragments were combined via Gibson assembly.

##### Vector 68: Pyl tRNA_CCUA_-PylRS-mCherry-P2A-eGFP(150TAGG)

**Vector 38** digested with EcoRI and BglII to generate vector. Insert 1 generated by PCR of **Vector 38** with **Primer 50** and **Primer 51.** Insert 2 generated by PCR of **Vector 51** with **Primer 52** and **Primer 53**. The three fragments were combined via Gibson assembly.

##### Vector 69: Pyl tRNA_GCUA_-PylRS-mCherry-P2A-eGFP(150TAGC)

**Vector 37** digested with EcoRI and BglII to generate vector. Insert 1 generated by PCR of **Vector 37** with **Primer 50** and **Primer 51.** Insert 2 generated by PCR of **Vector 51** with **Primer 52** and **Primer 53**. The three fragments were combined via Gibson assembly.

##### Vector 70: Pyl tRNA_CUA_-PylRS-mCherry-P2A-eGFP

**Vector 3** digested with EcoRI and BglII to generate the vector. Insert 1 generated by PCR of **Vector 51** with **Primer 52** and **Primer 53**. Insert 2 generated by PCR of **Vector 4** with **Primer 50** and **Primer 51**. The three fragments were combined via Gibson Assembly.

##### Vector 71: Tyr tRNA_CUA_-TyrRS∗-mCherry-P2A-eGFP(150TAG)

**Vector 41** digested with EcoRI and BglII to generate vector. Insert 1 generated by PCR of **Vector 51** with **Primer 52** and **Primer 53**. Insert 2 generated by PCR of **Vector 41** with **Primer 50** and **Primer 51**. The three fragments were combined via Gibson Assembly.

##### Vector 72: Tyr tRNA_CUA_-EAziRS-mCherry-P2A-eGFP(150TAG)

**Vector 71** digested with BamHI and HindIII to generate vector. Insert 1 generated by PCR of **Vector 71** with **Primer 54** and **Primer 55**. Insert 2 generated by PCR of **Vector 71** with **Primer 56** and **Primer 57**. Insert 3 generated by PCR of **Vector 71** with **Primer 58** and **Primer 59**. The three fragments were combined via Gibson Assembly.

##### Vector 73: (Pyl tRNA_UCCU (Ev2)_)x4-PylRS-mCherry-P2A-eGFP(150AGGA)

**Vector 42** digested with EcoRI and BglII to generate vector. Insert 1 generated by PCR of **Vector 51** with **Primer 52** and **Primer 53**. Insert 2 generated by PCR of **Vector 42** with **Primer 50** and **Primer 51**. The three fragments were combined via Gibson Assembly.

##### Vector 74: (Tyr tRNA_CUA_)x4-TyrRS∗-mCherry-P2A-eGFP(150TAG)

**Vector 43** digested with EcoRI and BglII to generate vector. Insert 1 generated by PCR of **Vector 51** with **Primer 52** and **Primer 53**. Insert 2 generated by PCR of **Vector 43** with **Primer 50** and **Primer 51**. The three fragments were combined via Gibson Assembly.

#### Transient transfection

HEK293 cells were plated at a 1x10^6^ cells per well of a 24 well plate (Corning, #10380932) in DMEM (Fisher Scientific, #11574516) supplemented with 10% (v/v) FBS (Fisher Scientific, #11573397) and maintained at 37°C in a high-humidity 5% CO_2_ atmosphere for 24 h or until 90% confluent. For transfection, per well, 1.5 μL Lipofectamine 2000 (Life Technologies, #10696343) was suspended in 50 μL Opti-MEM (Fisher Scientific, #11058021), and incubated for 10 min at room temperature. 500 ng of Vector was diluted in 50 μL Opti-MEM and mixed with the lipofectamine/Opti-MEM solution to a final volume of 100 μL and incubated for 30 minutes at room temperature. Media in each well was exchanged for fresh DMEM/10% FBS and, when appropriate, supplemented with 100 mM stock of BocK (Fluorochem, #078520) and/or AzF (Bachem, #4020250) or OMeY (Alfa Aesar, #H63096) to final working concentrations of 1 mM. Vector-lipofectamine solution was then added drop-wise to the well, and the plate incubated at 37°C in a high-humidity 5% CO_2_ atmosphere for 48 h.

#### Imaging

Cells were imaged using Zeiss AxioCam MRm microscope camera and ZEN (version 2.3) computer imaging programme. All images were taken at 200X magnification, and representative regions of the entire well were captured. GFP fluorescence was detected with Zeiss FSet 38 green fluorescence filter (excitation 470/40 and emission 525/50), with a constant exposure of 550 ms per image. mCherry fluorescence was detected with Zeiss FSet 45 (excitation 560/40 and emission 630/75), with a constant exposure of 550 ms per image. To analyse dual-fluorescence reporters, GFP and mCherry fluorescent images were composited and the resulting images annotated as ‘Merge’.

#### Flow cytometry

After transfection, media was aspirated from wells and cells washed with 100 μL DPBS. Then, 50 μL of trypsin was added to each well and the plate incubated at 37°C, 5% CO_2_ for 5 minutes. The plate was sharply tapped to ensure cellular detachment and cells were resuspended in 450 μL DPBS and filtered through a CellTrics® 50 μM filter (Sysmex #04-0042-2317) into a round-bottom flow cytometry tube. Flow cytometry was performed using an S3e cell sorter (Bio-Rad). For each condition, acquisition was allowed to continue until at least 10,000 mCherry-positive cells had been analysed (see quantification and statistical analysis). Each experiment was performed in triplicate.

#### Immunoblotting

Immediately following microscopical analysis the media was removed from wells and cells washed twice with 1x PBS. 50 μL of RIPA Buffer (Sigma-Aldrich, #R0278) containing 1% v/v protease inhibitor cocktail (Sigma-Aldrich, #P8340) was added dropwise to the centre of the well and plate incubated on ice for 10 minutes. Cells were then scraped from the surface of the well, and all contents transferred to a 1.5 mL Eppendorf tube. Lysates were pelleted (20,000 *g*, 10 min, 4 °C) and supernatant (45 μL) added to 15 μL NuPAGE™ LDS Sample Buffer (Invitrogen, #NP0007). Samples were heated (95 °C, 5 min) and loaded onto a Novex™ WedgeWell™ 4-20% Tris-Glycine 1.0 mm Mini Protein Gel (Invitrogen, #XP04205BOX) for electrophoresis. The gel was transferred to a nitrocellulose membrane using a Trans-Blot Turbo Transfer System (Bio-Rad), stained with Ponceau S to confirm protein transfer and imaged on a ChemiDoc XRS + (Bio-Rad). Once imaged, membranes were blocked with PBST (0.05% [v/v] Tween 20 in PBS) containing 5% (w/v) milk powder at 16 °C for 1 h, and then incubated (4 °C, overnight) with primary mouse anti-eGFP antibody (ThermoFisher, #MA1-952, 1:500 [v/v] dilution). The membrane was then washed three times (10 mL PBST, 5 min per wash). All subsequent washing steps used this procedure. Membranes were incubated in secondary anti-mouse antibody (ThermoFisher, #32430, 1:1000 [v/v] dilution) for 1 h at 16°C, and then washed. The signal was developed by addition of Clarity Max™ Western ECL Substrate (Bio-Rad, #1705062). After imaging on a ChemiDoc XRS + system, the membrane was washed 5 times (10 mL PBST, 5 min per wash) and then incubated (4 °C, overnight) with either mouse anti-GAPDH antibody (ThermoFisher, #MA5-15738, 1:500 [v/v] dilution) or mouse anti-FLAG (Sigma-Aldrich, #F3165, 1:500 [v/v] dilution) before being incubated with the secondary anti-mouse antibody and processed again for imaging as described above.

### Quantification and statistical analysis

Flow cytometry data was analysed with FlowJo (version 10.7.2, BD Biosciences). Events were gated to ensure only mCherry-positive, single cells were used for quantification purposes. This was achieved by gating for single cells, excluding debris (FSC-A vs SSC-A) and gating to exclude doublets (FSC-A vs FSC-H and SSC-A vs SSC-H). Cells were gated for transfected cells by excluding mCherry negative cells (mCh-A vs FSC-A). Finally, mCherry fluorescence was plotted against eGFP fluorescence and the mean fluorescence intensities were taken to calculate the incorporation efficiency using the formula (Bartoschek et al., 2021):

$$Incoporation\;efficiency=\left(\frac{eGFP}{mCh}/\frac{WT\;eGFP}{WT\;mCh}\right)-\left[\frac{eGFP}{mCh}/\frac{WT\;eGFP}{WT\;mCh}\right]$$

Where ( ) and [ ] indicate samples incubated with and without the designated unnatural amino acid, respectively. Each condition was performed in triplicate (i.e. transient transfection of cells of different passages and split at different time). Data of each replicate containing over 10,000 mCherry positive cells were used to calculate the incorporation efficiency. Average incorporation efficiency was calculated from the three replicates for each condition and are provided in bar charts in Figures 2D, 3D, and 3E. The standard deviation from the means were also calculated and are represented as error bars on Figures 2D, 3D, and 3E and after ± symbols in Figures 3A and 3B. In the results, when directly comparing incorporation efficiencies between two vectors, an independent two-tailed t-test assuming equal variances was used. Incorporation efficiency, average incorporation efficiency, standard deviation from the mean and t-test calculations were performed in Microsoft Excel.

## Data Availability

• All FACS data have been deposited to the Cardiff University data catalogue and are publicly available as of the date of publication. DOIs are listed in the key resources table. Microscopy data reported in this paper will be shared by the lead contact upon request. • This paper does not report original code. • Any additional information required to reanalyse the data reported in this paper is available from the lead contact upon request.

## Supplemental information

Document S1. Supplemental experimental procedures and Figures S1–S4

Document S2. Article plus supplemental information

## Footnotes

Supplemental information can be found online at https://doi.org/10.1016/j.crmeth.2021.100073.

## References

[bib1] Anderson J.C., Wu N., Santoro S.W., Lakshman V., King D.S., Schultz P.G. (2004). An expanded genetic code with a functional quadruplet codon. Proc. Natl. Acad. Sci. U S A.

[bib2] Bartoschek M.D., Ugur E., Nguyen T.A., Rodschinka G., Wierer M., Lang K., Bultmann S. (2021). Identification of permissive amber suppression sites for efficient non-canonical amino acid incorporation in mammalian cells. Nucleic Acids Res..

[bib3] Brown W., Liu J.H., Deiters A. (2018). Genetic code expansion in animals. ACS Chem. Biol..

[bib4] Cabantous S., Terwilliger T.C., Waldo G.S. (2005). Protein tagging and detection with engineered self-assembling fragments of green fluorescent protein. Nat. Biotechnol..

[bib5] Chatterjee A., Xiao H., Bollong M., Ai H.W., Schultz P.G. (2013). Efficient viral delivery system for unnatural amino acid mutagenesis in mammalian cells. Proc. Natl. Acad. Sci. U S A.

[bib6] Chen Y., Wan Y., Wang N., Yuan Z., Niu W., Li Q., Guo J. (2018). Controlling the replication of a genomically recoded HIV-1 with a functional quadruplet codon in mammalian cells. ACS Synth. Biol..

[bib7] Chen Y.T., Ma J., Lu W.Q., Tian M.L., Thauvin M., Yuan C.G., Volovitch M., Wang Q., Holst J., Liu M.Y. (2017). Heritable expansion of the genetic code in mouse and zebrafish. Cell Res..

[bib8] Chin J.W. (2017). Expanding and reprogramming the genetic code. Nature.

[bib9] Cuthbertson L., Nodwell J.R. (2013). The TetR family of regulators. Microbiol. Mol. Biol. Rev..

[bib10] de la Torre D., Chin J.W. (2021). Reprogramming the genetic code. Nat. Rev. Genet..

[bib11] Dumas A., Lercher L., Spicer C.D., Davis B.G. (2015). Designing logical codon reassignment - expanding the chemistry in biology. Chem. Sci..

[bib12] Dunkelmann D.L., Willis J.C.W., Beattie A.T., Chin J.W. (2020). Engineered triply orthogonal pyrrolysyl-tRNA synthetase/tRNA pairs enable the genetic encoding of three distinct non-canonical amino acids. Nat. Chem..

[bib13] Fink T., Lonzaric J., Praznik A., Plaper T., Merljak E., Leben K., Jerala N., Lebar T., Strmsek Z., Lapenta F. (2019). Design of fast proteolysis-based signaling and logic circuits in mammalian cells. Nat. Chem. Biol..

[bib14] Gautier A., Nguyen D.P., Lusic H., An W.A., Deiters A., Chin J.W. (2010). Genetically encoded photocontrol of protein localization in mammalian cells. J. Am. Chem. Soc..

[bib15] Han S., Yang A., Lee S., Lee H.W., Park C.B., Park H.S. (2017). Expanding the genetic code of *Mus musculus*. Nat. Commun..

[bib16] Huang Y., Liu T. (2018). Therapeutic applications of genetic code expansion. Synth. Syst. Biotechnol..

[bib17] Italia J.S., Latour C., Wrobel C.J.J., Chatterjee A. (2018). Resurrecting the bacterial tyrosyl-tRNA synthetase/tRNA pair for expanding the genetic code of both *E. coli* and Eukaryotes. Cell Chem Biol..

[bib18] Ivanov V., Beniaminov A., Mikheyev A., Minyat E. (2001). A mechanism for stop codon recognition by the ribosome: a bioinformatic approach. RNA.

[bib19] Kamiyama D., Sekine S., Barsi-Rhyne B., Hu J., Chen B., Gilbert L.A., Ishikawa H., Leonetti M.D., Marshall W.F., Weissman J.S. (2016). Versatile protein tagging in cells with split fluorescent protein. Nat. Commun..

[bib20] Kato Y. (2019). Translational control using an expanded genetic code. Int. J. Mol. Sci..

[bib21] Kim J.H., Lee S.R., Li L.H., Park H.J., Park J.H., Lee K.Y., Kim M.K., Shin B.A., Choi S.Y. (2011). High cleavage efficiency of a 2A peptide derived from porcine teschovirus-1 in human cell lines, zebrafish and mice. Plos One.

[bib22] Kitada T., DiAndreth B., Teague B., Weiss R. (2018). Programming gene and engineered-cell therapies with synthetic biology. Science.

[bib23] Krogager T.P., Ernst R.J., Elliott T.S., Calo L., Beranek V., Ciabatti E., Spillantini M.G., Tripodi M., Hastings M.H., Chin J.W. (2018). Labeling and identifying cell-specific proteomes in the mouse brain. Nat. Biotechnol..

[bib24] Li Y., Wang S., Chen Y., Li M., Dong X., Hang H.C., Peng T. (2020). Site-specific chemical fatty-acylation for gain-of-function analysis of protein S-palmitoylation in live cells. Chem. Commun..

[bib25] Liu J., Hemphill J., Samanta S., Tsang M., Deiters A. (2017). Genetic code expansion in zebrafish embryos and its application to optical control of cell signaling. J. Am. Chem. Soc..

[bib26] Meineke B., Heimgartner J., Eirich J., Landreh M., Elsasser S.J. (2020). Site-specific incorporation of two ncAAs for two-color bioorthogonal labeling and crosslinking of proteins on live mammalian cells. Cell Rep..

[bib27] Meineke B., Heimgartner J., Lafranchi L., Elsasser S.J. (2018). Methanomethylophilus alvus Mx1201 provides basis for mutual orthogonal pyrrolysyl tRNA/aminoacyl-tRNA synthetase pairs in mammalian cells. ACS Chem. Biol..

[bib28] Melnikov S., Manakongtreecheep K., Soll D. (2018). Revising the structural diversity of ribosomal proteins across the three domains of life. Mol. Biol. Evol..

[bib29] Monk J.W., Leonard S.P., Brown C.W., Hammerling M.J., Mortensen C., Gutierrez A.E., Shin N.Y., Watkins E., Mishler D.M., Barrick J.E. (2017). Rapid and inexpensive evaluation of nonstandard amino acid incorporation in *Escherichia coli*. ACS Synth. Biol..

[bib30] Moore B., Persson B.C., Nelson C.C., Gesteland R.F., Atkins J.F. (2000). Quadruplet codons: implications for code expansion and the specification of translation step size. J. Mol. Biol..

[bib31] Neumann-Staubitz P., Neumann H. (2016). The use of unnatural amino acids to study and engineer protein function. Curr. Opin. Struct. Biol..

[bib32] Neumann H., Wang K.H., Davis L., Garcia-Alai M., Chin J.W. (2010). Encoding multiple unnatural amino acids via evolution of a quadruplet-decoding ribosome. Nature.

[bib33] Nguyen D.P., Miyaoka Y., Gilbert L.A., Mayerl S.J., Lee B.H., Weissman J.S., Conklin B.R., Wells J.A. (2016). Ligand-binding domains of nuclear receptors facilitate tight control of split CRISPR activity. Nat. Commun..

[bib34] Niu W., Schultz P.G., Guo J. (2013). An expanded genetic code in mammalian cells with a functional quadruplet codon. ACS Chem. Biol..

[bib35] Nodling A.R., Spear L.A., Williams T.L., Luk L.Y.P., Tsai Y.-H. (2019). Using genetically incorporated unnatural amino acids to control protein functions in mammalian cells. Essays Biochem..

[bib36] O'Connor M. (2002). Insertions in the anticodon loop of tRNA1Gln(sufG) and tRNA(Lys) promote quadruplet decoding of CAAA. Nucleic Acids Res..

[bib37] Potts K.A., Stieglitz J.T., Lei M., Van Deventer J.A. (2020). Reporter system architecture affects measurements of noncanonical amino acid incorporation efficiency and fidelity. Mol. Syst. Des. Eng..

[bib38] Ross B., Mehta S., Zhang J. (2016). Molecular tools for acute spatiotemporal manipulation of signal transduction. Curr. Opin. Chem. Biol..

[bib39] Scheller L., Fussenegger M. (2019). From synthetic biology to human therapy: engineered mammalian cells. Curr. Opin. Biotechnol..

[bib40] Schmied W.H., Elsasser S.J., Uttamapinant C., Chin J.W. (2014). Efficient multisite unnatural amino acid incorporation in mammalian cells via optimized pyrrolysyl tRNA synthetase/tRNA expression and engineered eRF1. J. Am. Chem. Soc..

[bib41] Singh V. (2014). Recent advances and opportunities in synthetic logic gates engineering in living cells. Syst. Synth. Biol..

[bib42] Suzuki T., Asami M., Patel S.G., Luk L.Y.P., Tsai Y.-H., Perry A.C.F. (2018). Switchable genome editing via genetic code expansion. Sci. Rep..

[bib43] Suzuki T., Miller C., Guo L.T., Ho J.M.L., Bryson D.I., Wang Y.S., Liu D.R., Soll D. (2017). Crystal structures reveal an elusive functional domain of pyrrolysyl-tRNA synthetase. Nat. Chem. Biol..

[bib44] Taki M., Matsushita J., Sisido M. (2006). Expanding the genetic code in a mammalian cell line by the introduction of four-base codon/anticodon pairs. ChemBioChem.

[bib45] Takimoto J.K., Adams K.L., Xiang Z., Wang L. (2009). Improving orthogonal tRNA-synthetase recognition for efficient unnatural amino acid incorporation and application in mammalian cells. Mol. Biosyst..

[bib46] Tharp J.M., Ehnbom A., Liu W.R. (2018). tRNA(Pyl): structure, function, and applications. RNA Biol..

[bib47] Wang K., Sachdeva A., Cox D.J., Wilf N.M., Lang K., Wallace S., Mehl R.A., Chin J.W. (2014). Optimized orthogonal translation of unnatural amino acids enables spontaneous protein double-labelling and FRET. Nat. Chem..

[bib48] Wang N., Shang X., Cerny R., Niu W., Guo J. (2016). Systematic evolution and study of UAGN decoding tRNAs in a genomically recoded bacteria. Sci. Rep..

[bib49] Willis J.C.W., Chin J.W. (2018). Mutually orthogonal pyrrolysyl-tRNA synthetase/tRNA pairs. Nat. Chem..

[bib50] Wu C.Y., Roybal K.T., Puchner E.M., Onuffer J., Lim W.A. (2015). Remote control of therapeutic T cells through a small molecule-gated chimeric receptor. Science.

[bib51] Xiao H., Chatterjee A., Choi S.H., Bajjuri K.M., Sinha S.C., Schultz P.G. (2013). Genetic incorporation of multiple unnatural amino acids into proteins in mammalian cells. Angew. Chem. Int. Ed..

[bib52] Yanagisawa T., Ishii R., Fukunaga R., Kobayashi T., Sakamoto K., Yokoyama S. (2008). Multistep engineering of pyrrolysyl-tRNA synthetase to genetically encode N(epsilon)-(o-azidobenzyloxycarbonyl) lysine for site-specific protein modification. Chem. Biol..

[bib53] Zetsche B., Volz S.E., Zhang F. (2015). A split-Cas9 architecture for inducible genome editing and transcription modulation. Nat. Biotechnol..

[bib54] Zheng Y., Addy P.S., Mukherjee R., Chatterjee A. (2017). Defining the current scope and limitations of dual noncanonical amino acid mutagenesis in mammalian cells. Chem. Sci..

[bib55] Zheng Y., Mukherjee R., Chin M.A., Igo P., Gilgenast M.J., Chatterjee A. (2018). Expanding the scope of single- and double-noncanonical amino acid mutagenesis in mammalian cells using orthogonal polyspecific leucyl-tRNA synthetases. Biochemistry.

[bib56] Zhou W., Smidlehner T., Jerala R. (2020). Synthetic biology principles for the design of protein with novel structures and functions. FEBS Lett..

