# Supplementary material for: Development of mammalian cell logic gates controlled by unnatural amino acids
Source: Cell Rep Methods. 2021 Sep 16;1(6):100073. doi: 10.1016/j.crmeth.2021.100073 (PMC9017196; doi:10.1016/j.crmeth.2021.100073)
Supplement: Document S2. Article plus supplemental information [file mmc2.pdf]

Cell Reports
Methods

Article

Development of mammalian cell logic gates controlled by unnatural amino acids

Graphical abstract

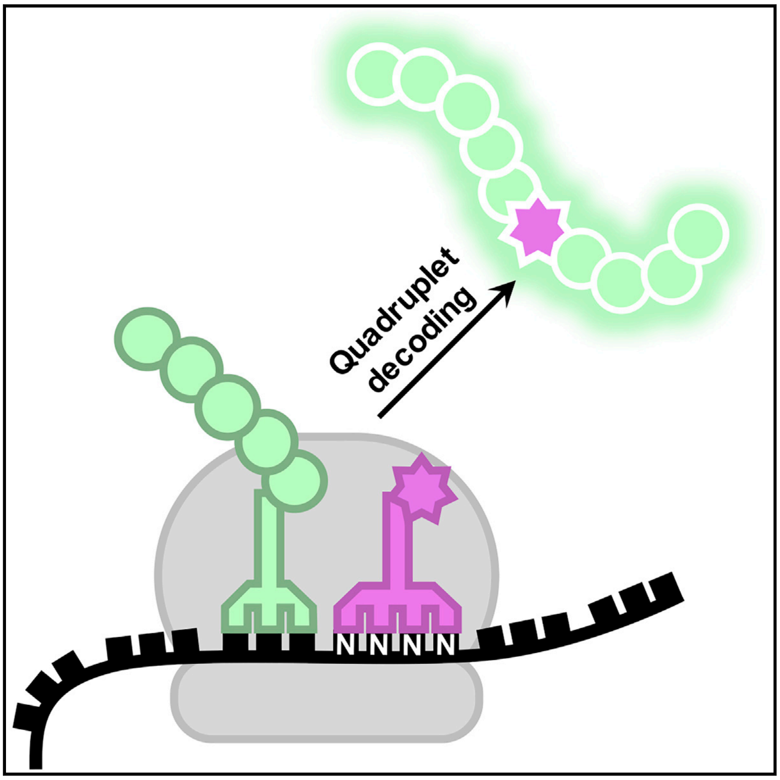

Highlights

- We evaluate the performance of 11 quadruplet-decoding tRNAs in HEK293
- Two tRNAs show high efficiency in decoding quadruplet codons
- We construct logic gates responding to two different unnatural amino acids
- These findings present an alternative approach to mammalian cell logic operations

Authors

Emily M. Mills, Victoria L. Barlow, Arwyn T. Jones, Yu-Hsuan Tsai

Correspondence

tsai.y-h@outlook.com

In brief

Logic gates enable regulation of protein function by small molecules, but these are often drug(-like) molecules with intrinsic biological activities. Mills et al. present an alternative approach through genetic code expansion, demonstrating that unnatural amino acids can act as biologically inert switches for effective mammalian cell logic operations.

Mills et al., 2021, Cell Reports Methods *1*, 100073
October 25, 2021 © 2021 The Author(s).
https://doi.org/10.1016/j.crmeth.2021.100073

CellPress

Cell Reports Methods

CellPress
OPEN ACCESS

Article

Development of mammalian cell logic gates controlled by unnatural amino acids

Emily M. Mills,[1,4] Victoria L. Barlow,[1,4] Arwyn T. Jones,[2] and Yu-Hsuan Tsai[1,3,5,*]
[1]School of Chemistry, Cardiff University, Main Building, Park Place, Cardiff, Wales CF10 3AT, UK
[2]School of Pharmacy and Pharmaceutical Sciences, Cardiff University, Redwood Building, Cardiff, Wales CF10 3NB, UK
[3]Institute of Molecular Physiology, Shenzhen Bay Laboratory, Shenzhen, Guangdong 518132, China
[4]These authors contributed equally
[5]Lead contact
*Correspondence: tsai.y-h@outlook.com
https://doi.org/10.1016/j.crmeth.2021.100073

MOTIVATION Genetic code expansion employing an orthogonal aminoacyl-tRNA synthetase/tRNA pair for site-specific unnatural amino acid incorporation has a wide range of applications. However, a blank (orthogonal) codon is needed for each orthogonal tRNA. Although the amber stop codon is commonly used as the blank codon, the use of stop codons as the blank codons inevitably limits the number of possible different unnatural amino acids to be incorporated into proteins in a single cell. To overcome this limitation, we evaluated the capability of 11 Pyl tRNA variants for decoding quadruplet codons in mammalian cells. With efficient quadruplet-decoding orthogonal tRNA variants available, it is possible for simultaneous incorporation of more than three different unnatural amino acids in mammalian cells.

SUMMARY

Mammalian cell logic gates hold great potential for wide-ranging applications. However, most of those currently available are controlled by drug(-like) molecules with inherent biological activities. To construct truly orthogonal circuits and artificial regulatory pathways, biologically inert molecules are ideal molecular switches. Here, we applied genetic code expansion and engineered logic gates controlled by two biologically inert unnatural amino acids. Genetic code expansion relies on orthogonal aminoacyl-tRNA synthetase/tRNA pairs for co-translational and site-specific unnatural amino acid incorporation conventionally in response to an amber (UAG) codon. By screening 11 quadruplet-decoding pyrrolysyl tRNA variants from the literature, we found that all variants decoding CUAG or AGGA tested here are functional in mammalian cells. Using a quadruplet-decoding orthogonal pair together with an amber-decoding pair, we constructed logic gates that can be successfully controlled by two different unnatural amino acids, expanding the scope of genetic code expansion and mammalian cell logic circuits.

INTRODUCTION

Logic gates refers to integrated systems where an input controls a desired output. Employing logic gates in mammalian cells is a fast-developing area for intricate reversible cellular control, with significant biotechnological and biomedical applications (Cuthbertson and Nodwell, 2013; Fink et al., 2019; Nguyen et al., 2016; Ross et al., 2016; Wu et al., 2015; Zetsche et al., 2015). Some examples include novel sensors, diagnostics, as well as therapeutics (Brown et al., 2018; Kitada et al., 2018; Scheller and Fussenegger, 2019; Singh, 2014; Zhou et al., 2020). In particular, logic gates responding to small molecules can provide (spatio)temporal control by the user, permitting targeted manipulation of a designated phenotype. Specifically, biologically inert molecules that have no influence on endogenous cellular events are ideal molecular switches to control logic gates, therefore enabling the construction of truly orthogonal circuits and artificial regulatory pathways in cells.

We hypothesized that this can be realized by using biologically inert unnatural (non-canonical) amino acids, which are artificial synthetic molecules and do not produce observable phenotypes or toxicities *in vitro* or *in vivo* (Chen et al., 2017; Han et al., 2017; Krogager et al., 2018; Liu et al., 2017; Suzuki et al., 2018). More importantly, such unnatural amino acids can be site-specifically incorporated into proteins in mammalian cells by repurposing the cellular translational machinery through the technique of genetic code expansion (Figure 1). This technique has wide-ranging applications in protein research (Chin, 2017; de la Torre and Chin, 2021; Dumas et al., 2015; Huang and Liu, 2018; Kato, 2019; Neumann-Staubitz and Neumann, 2016; Nodling et al.,

This is an open access article under the CC BY license (http://creativecommons.org/licenses/by/4.0/).

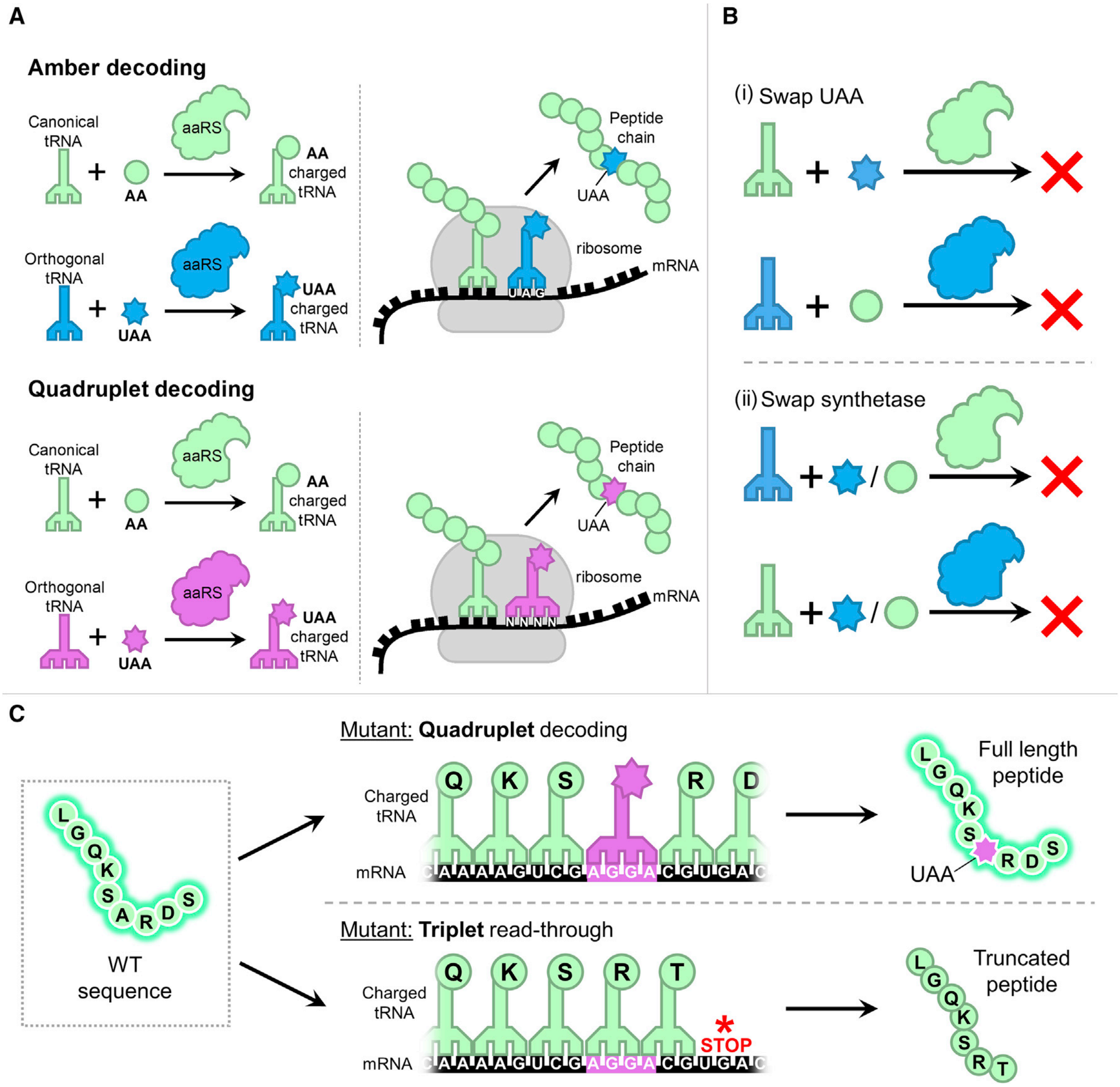

Figure 1. Principles of unnatural amino acid incorporation by genetic code expansion

(A) Amber and quadruplet suppression. Charged tRNA molecules are generated by aminoacylation of tRNA molecules by their respective synthetases with the respective amino acid or unnatural amino acid. Charged tRNA molecules then enter the ribosome and interact with the complementary codon on the mRNA template before formation of the peptide bond with the brought-in amino acid. Abbreviations are as follows: AA, amino acid; UAA, unnatural amino acid.

(B) Graphical representation of aaRS/tRNA pair orthogonality: (i) aaRS can only charge their paired tRNA with the respective amino acid, supplementation of a different amino acid results in no aminoacylation of tRNA; (ii) tRNA can only be aminoacylated by their respective aaRS, regardless of amino acid supplementation.

(C) Principles imposed when using quadruplet decoding. Alongside the introduction of the quadruplet codon to the gene, a further point mutation is introduced downstream to the codon to ensure that, if the first three bases of the quadruplet are translated by the canonical triplet tRNA, an in-frame stop codon is generated downstream to the incorporation site enabling premature truncation. Moreover, if the quadruplet is correctly decoded, this point mutation remains silent and does not alter the overall amino acid sequence of the protein.

2019), including the proposed use of unnatural amino acids as logic gate inputs. To incorporate an unnatural amino acid into a protein during translation, an orthogonal aminoacyl-tRNA synthetase (aaRS)/tRNA pair that selectively decodes a blank codon is required. The amber stop codon (UAG) is often used as the blank codon because it does not encode an amino acid and is the rarest codon in many organisms (Ivanov et al., 2001). The use of the amber codon to encode an unnatural amino acid is known as amber suppression (Figure 1A). To date, various amber suppressor orthogonal pairs have been developed (Chin, 2017; Dumas et al., 2015; Huang and Liu, 2018; Neumann-Staubitz and Neumann, 2016; Nodling et al., 2019). Within prokaryotic and eukaryotic systems, pyrrolysyl (Pyl)-tRNA synthetase (PylRS) and its cognate tRNA from the archaeal *Methanosarcina* species is arguably one of the most widely utilized and developed orthogonal pairs for unnatural amino acid

incorporation. Nevertheless, various *Escherichia coli* aaRS enzymes (e.g., *Ec*LeuRS, *Ec*TyrRS, *Ec*TrpRS) and their cognate tRNAs have also been exploited and specifically engineered to function as amber suppressor orthogonal pairs in mammalian cells. It is possible to employ several orthogonal pairs for simultaneous incorporation of multiple unnatural amino acids in a single mammalian cell (Meineke et al., 2018, 2020; Xiao et al., 2013; Zheng et al., 2017, 2018). However, such applications are often thwarted as these orthogonal pairs were originally engineered for amber decoding. To attain site-specific incorporation of multiple unnatural amino acids within a single cell, orthogonal pairs need to decode unique blank codons and have no crossover interactions (Figure 1B). An emerging strategy to generate additional blank codons is to use quadruplet (four-base) codons. Employing quadruplet codons can greatly expand the genetic code, providing the possibility for simultaneous incorporation of three or more distinct unnatural amino acids within a single cell (Dunkelmann et al., 2020).

Indeed, quadruplet decoding has been well-explored in *E. coli* (de la Torre and Chin, 2021; Moore et al., 2000; Neumann et al., 2010; O'Connor, 2002; Wang et al., 2014; Wang et al., 2016; Willis and Chin, 2018), including a recent example demonstrating simultaneous incorporation of three different unnatural amino acids (Dunkelmann et al., 2020). However, quadruplet decoding in mammalian cells is still largely unexplored and remains in its infancy (Anderson et al., 2004; Chen et al., 2018; Niu et al., 2013; Taki et al., 2006).

Here, we recognized the potential applications of genetic code expansion in the development of orthogonal logic circuits and engineered novel AND and OR logic gates that can be controlled by two distinct unnatural amino acids. As this proposed system design required two mutually orthogonal aaRS/tRNA pairs to respond to the logic inputs, we set out to identify a quadruplet-decoding aaRS/tRNA pair that is orthogonal to an amber-decoding aaRS/tRNA pair in mammalian cells. Specifically, we investigated the quadruplet-decoding efficiency of 11 Pyl $tRNA_{XXXX}$ variants previously tested in *E. coli* and found that all selected variants decoding AGGA (Niu et al., 2013; Wang et al., 2014) or CUAG (Wang et al., 2014) were functional in human embryonic kidney (HEK293) cells, whereas none of the tested variants were able to decode UAGN codons efficiently. We then confirmed the orthogonality of an amber-decoding pair to an AGGA-decoding PylRS/tRNA pair, enabling incorporation of two different unnatural amino acids in the same mammalian cell. After a series of system optimizations, we constructed AND and OR logic gates by using a split GFP reporter assay. To the best of our knowledge, this is the first example exploiting genetic code expansion in mammalian cell logic gates. This study not only expands the applications of genetic code expansion but also provides an alternative approach to engineer mammalian cell logic circuits.

RESULTS

Quadruplet-decoding analysis

Pyl tRNA variants that can decode UAGN (N = A/U/C/G), CUAG, or AGGA codons have been engineered for unnatural amino acid incorporation in *E. coli* (Chen et al., 2018; Niu et al., 2013; Wang et al., 2014, 2016). However, the translation machineries in *E. coli* and mammalian cells are different (Melnikov et al., 2018), thus tRNA optimized in one system might not work well in the other. In light of this, we evaluated the performance of 11 quadruplet-decoding Pyl tRNA variants in mammalian cells (Figure 2A). These tRNA variants consisted of the simple replacement of the CUA anticodon to NCUA (for decoding UAGN), CUAG (for decoding CUAG), or UCCU (for decoding AGGA), as well as previously identified tRNA variants that were evolved in *E. coli* and carry additional mutations in the anticodon stem loop. Pyl $tRNA_{UCUA(Ev1)}$, Pyl $tRNA_{CUAG(Ev1)}$, and Pyl $tRNA_{UCCU(Ev1)}$ were chosen for their high decoding efficiency in *E. coli* (Wang et al., 2014), whereas Pyl $tRNA_{UCUA(Ev2)}$ (Chen et al., 2018) and Pyl $tRNA_{UCCU(Ev2)}$ (Niu et al., 2013) have been used for unnatural amino acid incorporation in mammalian cells.

$N\varepsilon$-Boc-L-lysine (BocK) (Figure 2B) is a substrate of the wild-type PylRS (Yanagisawa et al., 2008). To perform a systematic comparison, we constructed dual-fluorescence reporters (Figure 2C) encoding the wild-type *Methanosarcina mazei* PylRS, a Pyl tRNA variant and an mCherry-P2A-eGFP(150XXXX) reporter, where the 150th amino acid residue in eGFP corresponds to a quadruplet codon. P2A is a self-cleavage sequence, leaving only a Pro residue in the C-terminal fragment after cleavage (Kim et al., 2011). In this reporter, mCherry is produced constitutively and thus serves as the transfection control. On the other hand, green fluorescence will only be observed upon successful decoding of the quadruplet codon and production of full-length eGFP. If the first three bases of a quadruplet codon are decoded as a triplet codon, this will lead to a translational frameshift and premature termination (Figure 1C). Thus, quadruplet-decoding efficiency can be calculated from the ratio between the two fluorescence intensities in the presence and absence of BocK (Bartoschek et al., 2021; Gautier et al., 2010; Monk et al., 2017; Potts et al., 2020; Schmied et al., 2014).

Experimentally, HEK293 cells were transiently transfected with a reporter vector (Figure 2C) and cultured in the presence or absence of 1 mM BocK, followed by flow cytometry analysis. Interestingly, in contrast to the observations in *E. coli* where all variants can decode the corresponding quadruplet codon (Niu et al., 2013; Wang et al., 2014, 2016), only Pyl tRNA variants decoding CUAG or AGGA appear to be functional (Figure 2D). Particularly, Pyl $tRNA_{CUAG}$ and Pyl $tRNA_{UCCU(Ev2)}$ outperformed the other variants, and the functional Pyl tRNA variants remained orthogonal in mammalian cells, manifesting as a lack of detectable eGFP in the absence of BocK by flow cytometry, fluorescence microscopy, or immunoblotting (Figures 2E and S1). Interestingly, a higher production of eGFP was observed with Pyl $tRNA_{UCCU(Ev2)}$ compared with Pyl $tRNA_{CUAG}$ when using a single fluorescence eGFP(150XXXX) reporter (Figure S1), although these findings could be due to a higher translation frequency or better transfection efficiency of the vector. Nevertheless, we chose to use $tRNA_{UCCU(Ev2)}$ for subsequent experiments.

Identification of a second orthogonal pair

To utilize genetic code expansion to control logic gate inputs, two orthogonal aaRS/tRNA pairs that selectively incorporate different unnatural amino acids are required. Several *E. coli* aaRS enzymes (e.g., *Ec*TyrRS) and their cognate tRNA

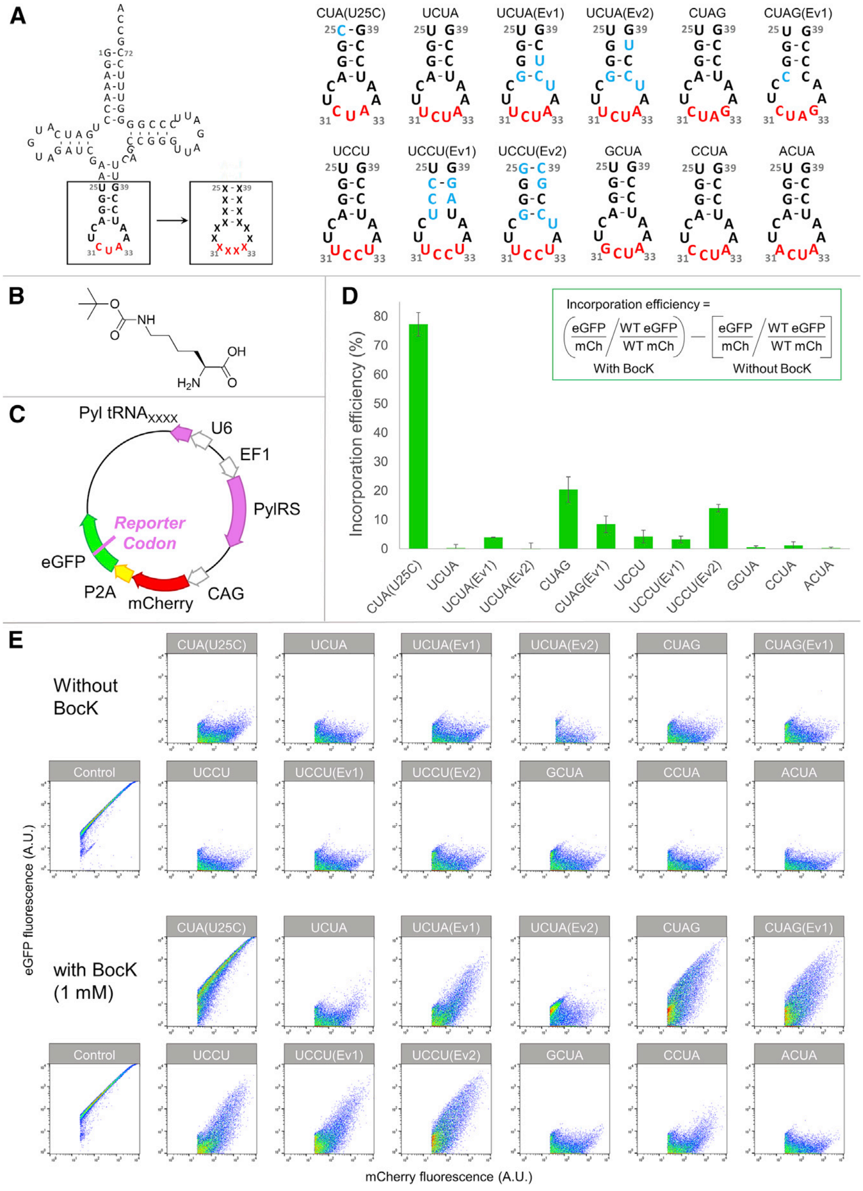

Figure 2. Pyl tRNA variants for quadruplet decoding in mammalian cells

(A) Nucleotide sequences of tRNA variants tested; X = A, C, G, or U; red denotes the anticodon, blue denotes mutations from the wild-type Pyl tRNA sequence in *M. mazei*, black denotes bases retained from the wild-type sequence.

(B) Chemical structure of BocK.

(C) Schematic representation of reporters used to analyze PylRS/tRNA$_{XXXX}$-mediated BocK incorporation into eGFP. The reporter codon encodes the 150th amino acid residue of eGFP. Lack of BocK incorporation results in termination of translation and no eGFP fluorescence.

(D) BocK incorporation efficiency of each tRNA$_{XXXX}$ variant. HEK293 cells were transiently transfected with a reporter vector and incubated in the presence or absence of 1 mM BocK. Cells underwent flow-cytometry analysis and the equation displayed was used to calculate incorporation efficiency (Bartoschek et al., 2021). Means and standard deviations calculated from three biological replicates are shown.

(E) Representative flow cytometry results used for calculating BocK incorporation efficiency. Events were gated for HEK293 cells and to exclude doublets. Cells were then gated to include only transfected (i.e., mCherry-positive) cells. Fluorescence intensities are given in arbitrary units (a.u.). The geometric mean of fluorescence intensity was used to calculate the incorporation efficiency. See also Figure S1 for fluorescence imaging and immunoblotting results of a single fluorescence eGFP(150XXXX) reporter.

molecules have been engineered as orthogonal pairs for amber suppression in mammalian cells to incorporate various unnatural amino acids (Italia et al., 2018; Nodling et al., 2019). As PylRS/tRNA can be used as an orthogonal pair in *E. coli*, it should remain orthogonal to engineered *Ec*TyrRS/tRNA$_{CUA}$ pairs. To verify this, an *Ec*TyrRS variant (referred to hereafter as TyrRS*) containing Y37V, D182S, F183M, and G265R mutations was chosen. TyrRS* was reported to be able to incorporate a range of unnatural amino acids, including *O*-methyl-L-tyrosine (OMeY) and 4-azido-L-phenylalanine (AzF), into proteins in mammalian cells (Chatterjee et al., 2013).

For systematic comparison, a reporter vector following the same design as the reporter vector for Pyl tRNA variants was generated, in which PylRS was replaced by TyrRS* and Pyl tRNA by Tyr tRNA$_{CUA}$ (Figure 3A). Although TyrRS* was shown to incorporate both OMeY and AzF efficiently (Chatterjee et al., 2013), we observed higher incorporation efficiency of AzF than OMeY by TyrRS* (Figure 3A) and the observable difference seemed to be more prominent in fluorescence microscopy analysis (Figure S2A). Further, AzF incorporation by TyrRS* was greater compared with an *Ec*TyrRS variant (Takimoto et al., 2009) evolved specifically for AzF incorporation (Figure S2B). We thus continued with AzF and TyrRS*/tRNA$_{CUA}$ to confirm the orthogonality with PylRS/tRNA$_{UCCU(Ev2)}$, and showed that the two pairs specifically recognized their respective unnatural amino acids (Figures 3B and S2C). We then transfected HEK293 cells with two vectors, one carrying only the TyrRS*/tRNA$_{CUA}$ machinery, and the other carrying the PylRS/tRNA$_{UCCU(Ev2)}$ pair alongside a double-incorporation reporter eGFP(40TAG,150AGGA) that contains a TAG codon for the 40th amino acid residue and an AGGA codon for the 150th residue in the *eGFP* gene. For 24 h, cells were maintained under four distinct conditions: (1) no unnatural amino acid, (2) BocK only, (3) AzF only, and (4) both BocK and AzF. Fluorescence was only detected when both unnatural amino acids were present (Figure S3), confirming the orthogonality of the

CellPress
OPEN ACCESS

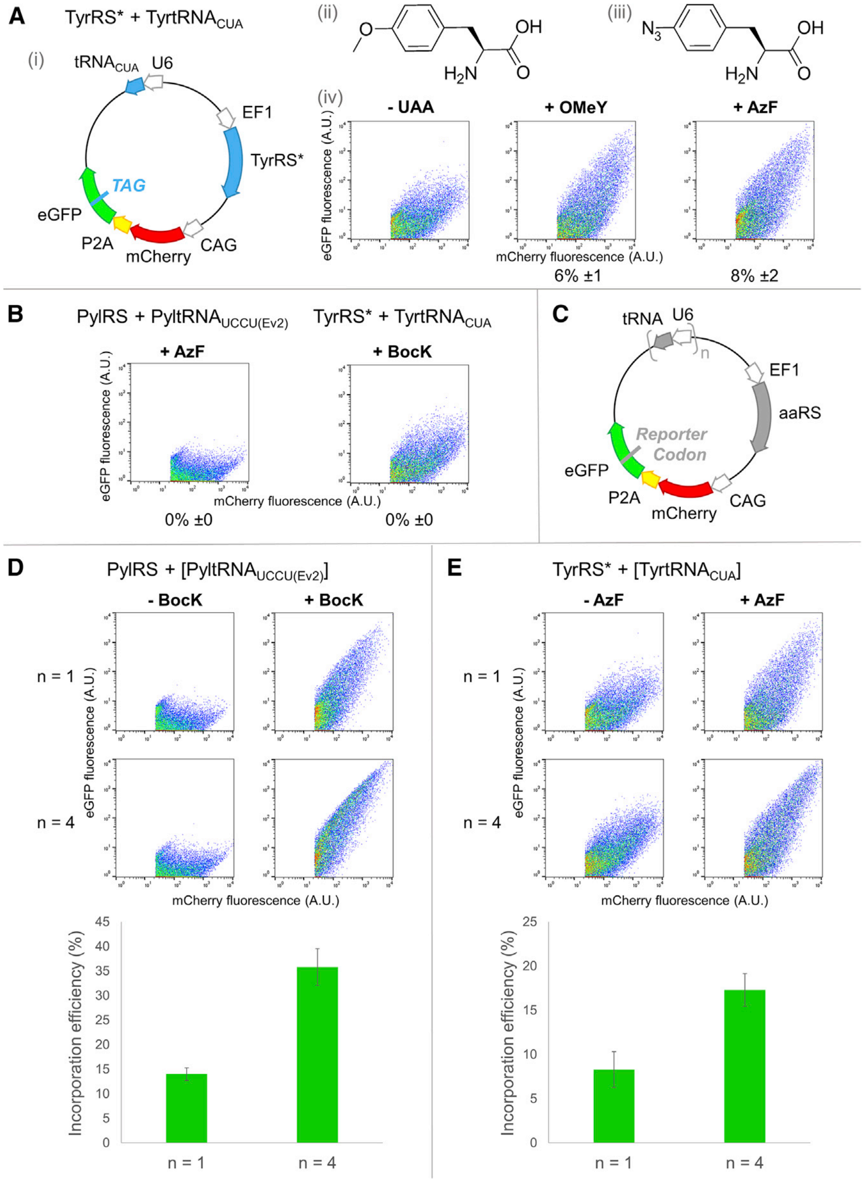

Figure 3. Confirmation of TyrRS*/$tRNA_{CUA}$ as the second orthogonal pair

(A) (i) The reporter to test efficiency of TyrRS*/$tRNA_{CUA}$. (ii) Chemical structure of OMeY. (iii) Chemical structure of AzF. (iv) OMeY or AzF incorporation efficiency by TyrRS*/$tRNA_{CUA}$ in HEK293 cells by using flow cytometry. Higher eGFP fluorescence was detected with AzF supplementation.

(B) Orthogonality of tRNA/aaRS pairs. HEK293 cells transfected with PylRS/$tRNA_{UCCU(Ev2)}$ or TyrRS*/$tRNA_{CUA}$ reporter vector and incubated for 24 h in the presence of AzF or BocK respectively. Fluorescent eGFP was only detected when the aaRS/tRNA pairs were in the presence of their cognate unnatural amino acid depicting mutual orthogonality of the pairs.

(C) Reporters for testing the impact of tRNA copy number on unnatural amino acid incorporation efficiency.

(D) Comparison of Pyl $tRNA_{UCCU(Ev2)}$ n = 1 and n = 4.

(E) Comparison of Tyr $tRNA_{CUA}$ n = 1 and n = 4. Flow cytometry was used to analyze BocK or AzF incorporation into the 150th residue of eGFP. Unnatural amino acids were supplemented into the cell growth medium to a final concentration of 1 mM. Means and standard deviations calculated from three biological replicates are shown. For incorporation efficiency calculation, see Figure 2.

two pairs as well as demonstrating their use for double incorporation into the same protein.

Optimization of unnatural amino acid incorporation

To use unnatural amino acids as logic gate inputs, their incorporation efficiency would directly affect the logic gate responses and so a higher incorporation efficiency is preferable. We first attempted to improve the incorporation efficiency by overexpression of an engineered eukaryotic release factor 1 (eRF1). With an E55D point mutation, this engineered eRF1 was shown to improve amber suppression by Pyl $tRNA_{CUA(U25C)}$ (Li et al., 2020; Schmied et al., 2014). We therefore tested the incorporation efficiency of both aaRS/tRNA pairs here in the presence and absence of this engineered eRF1; however, no significant improvement was observed in either case (Figure S2D).

It has also been reported that increasing the copy number of orthogonal tRNA can improve the unnatural amino acid incorporation efficiency in mammalian cells (Chatterjee et al., 2013; Schmied et al., 2014). Thus, we constructed vectors carrying four copies of tRNA (n = 1 or n = 4; Figure 3C), and both PylRS/$tRNA_{UCCU(Ev2)}$ and TyrRS*/$tRNA_{CUA}$ pairs demonstrated a significant increase in eGFP production ($p < 0.005$) (Figures 3D and 3E). Moreover, by increasing the tRNA and reporter copy numbers, a marked increase in eGFP production was also observed in the double-incorporation experiment, as demonstrated by fluorescence microscopy and immunoblotting (Figure S3).

Construction of logic gates controlled by two unnatural amino acids

For proof of concept, we then applied PylRS/$tRNA_{UCCU(Ev2)}$ and TyrRS*/$tRNA_{CUA}$ pairs to construct logic gates controlled by two unnatural amino acids, BocK and AzF. The logic gates were designed by using a split GFP system, which requires the concurrent presence of two complementary polypeptides sGFP(1–10) and sGFP(11) to form a fluorescent complex (Cabantous et al., 2005; Kamiyama et al., 2016). When produced individually, the GFP chromophore is unable to assemble, and no fluorescence can be detected (Figures 4A and 4B). In short, the designed logic gates

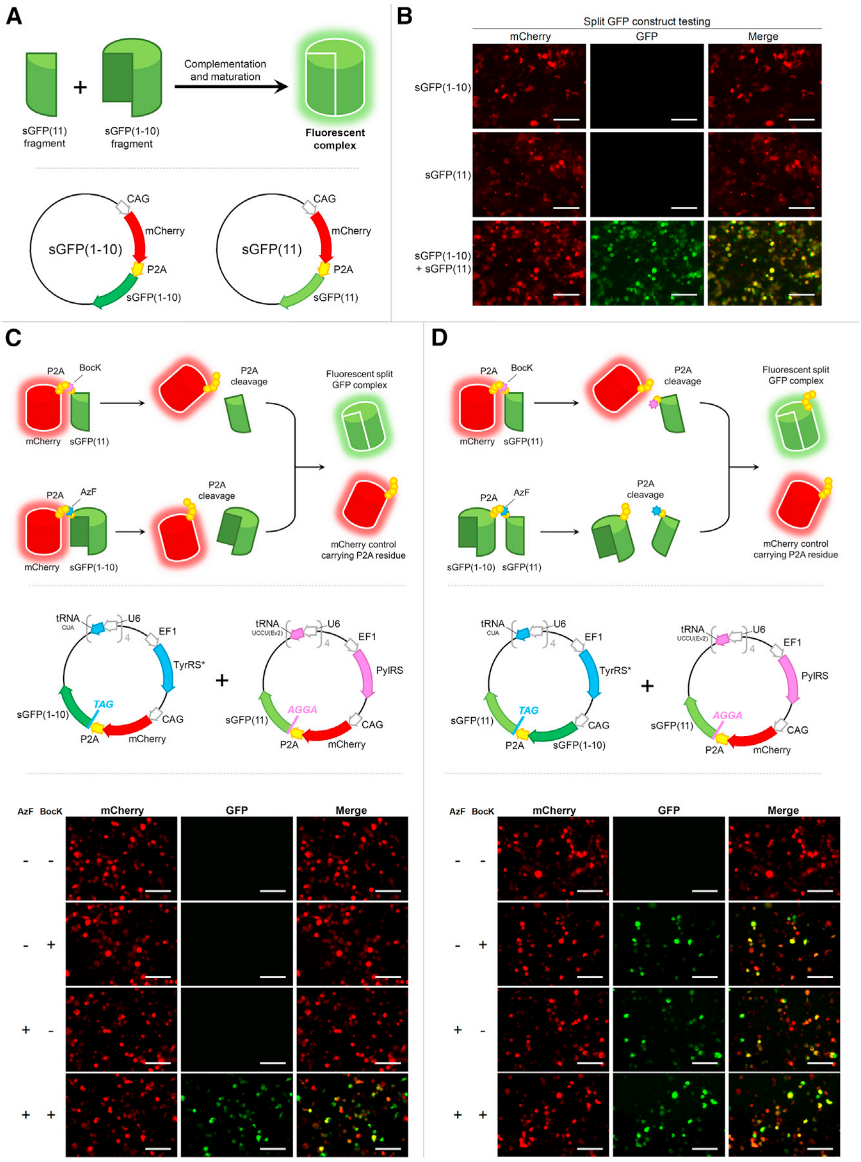

Figure 4. Exploiting genetic code expansion for AND and OR mammalian cell logic gates

(A) Principles of how split GFP works and vector designs used for testing the core design for logic operations.

(B) Fluorescent microscope imaging of HEK293 transfected with either or both vectors depicted in (A), GFP fluorescence was only detected when both fragments were present; mCherry expression was equal across all conditions.

(C and D) Shown are (C) AND logic gate and (D) OR logic gate controlled by BocK and AzF. Graphical depiction of principle approach is depicted at the top panel. Vector designs are shown in the middle. Fluorescence imaging results are shown at the bottom panel. HEK293 cells were transfected with the two vectors and incubated under four different conditions: no unnatural amino acid, BocK only, AzF only, or BocK and AzF. mCherry fluorescence indicated transfection efficiency and was at similar intensity across all four conditions. GFP fluorescence represents the logic operation output. All images are 223 μm × 167 μm, scale bars denote 50 μm. All unnatural amino acids used were supplemented into the cell growth medium to obtain a final concentration of 1 mM. See also Figure S4.

have two inputs (i.e., BocK and AzF) and one output (i.e., GFP fluorescence). To test them, we created a two-vector system based on the dual-fluorescence reporters (Figures 2 and 3) whereby there is an N-terminal mCherry leading to the sGFP fragments via a P2A sequence (Figures 4A and 4B). These vectors would hence allow for control of sGFP fragment production via selected introduction of TAG or AGGA codon upstream of the sGFP sequences.

To generate a logic gate that performs AND operation, we inserted TAG and AGGA codons into the reporter genes before the sequences encoding sGFP(1–10) and sGFP(11), respectively (Figure 4C). In this case, production of sGFP(1–10) and sGFP(11) became strictly dependent on the presence of AzF and BocK concurrently, and only in the presence of both AzF and BocK can the fluorescent complex form.

HEK293 cells were transiently transfected with two vectors (Figure 4C). One vector encoded the TyrRS*/tRNA$_{CUA}$ pair and mCherry-P2A-TAG-sGFP(1–10), whereas the other vector encodes PylRS/tRNA$_{UCCU(Ev2)}$ and mCherry-P2A-AGGA-sGFP(11). As expected, cells with green fluorescence were only observed in the presence of both AzF and BocK, and the level of GFP fluorescence indicates a promising efficiency of the operation.

Using a similar concept, we constructed an OR logic gate (Figure 4D). In this case, sGFP(1–10) was constantly produced, but sGFP(11) was only generated in the presence of either AzF, BocK, or both. Thus, green fluorescence is observed when at least one unnatural amino acid is present, and again the GFP fluorescence observed indicates a promising operational efficiency.

Finally, to confirm the specificity of BocK/AzF dependence and continuation of orthogonality, the vectors used for logic testing were individually transfected into HEK293 cells and incubated in the presence or absence of their respective unnatural amino acids. Upon imaging, no green fluorescence was detected regardless of unnatural amino acid supplementation for AND logic vectors (Figure S4). Indeed, green fluorescence was only detected with the OR logic vector containing TyrRS*/tRNA$_{CUA}$ and in the presence of AzF.

DISCUSSION

Here, we have demonstrated the applications of genetic code expansion for developing novel mammalian cell logic gates.

We quantified the quadruplet-decoding efficiency of 11 Pyl tRNA variants in HEK293 cells. Unlike most other aaRS enzymes, PylRS does not recognize the anticodon stem loop on the Pyl tRNA during aminoacylation (Suzuki et al., 2017; Tharp et al., 2018), so alteration of the anticodon is theoretically tolerated by the synthetase. Nevertheless, whereas change of the anticodon from CUA to CUAG or UCCU was tolerated, Pyl tRNA variants with the anticodon NCUA did not seem to be functional in mammalian cells (Figures 2 and S1). In addition, although variation in the nucleic acid sequence of the anticodon stem loop can significantly affect the incorporation efficiency, we found that tRNA sequences optimized in *E. coli* (Niu et al., 2013; Wang et al., 2014, 2016) might not necessarily work well in mammalian cells. For example, Pyl $tRNA_{CUAG}$ outperformed Pyl $tRNA_{CUAG(Ev1)}$.

Successful incorporation of an unnatural amino acid involves four steps: (1) cellular uptake of the designated unnatural amino acid; (2) aminoacylation of the orthogonal tRNA by the orthogonal synthetase; (3) formation of a ternary complex containing aminoacylated tRNA, eEF1A (or EF-Tu in bacteria), and GTP; and (4) interaction of the ternary complex with the ribosome, followed by peptide bond formation. The high incorporation efficiency of BocK by Pyl $tRNA_{CUA(U25C)}$ indicates that BocK can be readily taken up by mammalian cells. Additionally, all tested Pyl tRNA variants should be able to be aminoacylated by PylRS given that all variants were reported to be functional in *E. coli*. Thus, the differences in the prokaryotic and eukaryotic translational machinery (Melnikov et al., 2018) are likely the cause of the observed discrepancy in decoding efficiency, although it remains elusive whether formation of the ternary complex or interaction of the ternary complex with the ribosome is the main cause.

Intriguingly, negligible eGFP production was observed with Pyl $tRNA_{UCUA}$ and Pyl $tRNA_{UCUA(Ev2)}$. These two variants were reported to be functional in mammalian cells (Chen et al., 2018), although under different experimental conditions. Thus, the discrepancy might result from the choice of the cell line (HEK293 versus HEK293T), BocK concentration (1 mM versus 5 mM), unnatural amino acid incorporation site (40th versus 150th residue in eGFP), or a combination of these factors.

To construct double-input logic gates, another orthogonal pair, in addition to PylRS/$tRNA_{UCCU(Ev2)}$, is required. We chose the *E. coli*-derived TyrRS*/$tRNA_{CUA}$ as it had been optimized for incorporating a range of unnatural amino acids in mammalian cells (Chatterjee et al., 2013). Given that PylRS/tRNA can be used as an orthogonal pair in *E. coli*, it should remain orthogonal to TyrRS*/$tRNA_{CUA}$ in mammalian cells. Indeed, each pair specifically recognized their respective unnatural amino acids (Figure 3B). Furthermore, they can function orthogonally in the same cell, as demonstrated in the double-incorporation experiment (Figure S3). It is noteworthy that PylRS and TyrRS* can mediate incorporation of different unnatural amino acids, including cyclopropene lysine and propargyl tyrosine (Nodling et al., 2019) for bioorthogonal labeling, so the two orthogonal pairs can further be used for dual modification of proteins.

To use the two pairs in controlling logic gate inputs, higher incorporation efficiency of the two employed unnatural amino acids is ideal. Co-expression of an engineered eRF1 was previously shown to increase the unnatural amino acid incorporation efficiency by PylRS/$tRNA_{CUA(U25C)}$ in mammalian cells (Schmied et al., 2014). However, co-expression of the engineered eRF1 did not appear to improve AzF incorporation by TyrRS*/$tRNA_{CUA}$ or BocK incorporation by PylRS/$tRNA_{UCCU(Ev2)}$ (Figure S2D). This discrepancy could be due to the difference in the employed orthogonal pairs. In addition, the beneficial effect was most prominent with genes containing multiple UAG codons in the original study (Schmied et al., 2014), whereas we only used single UAG or AGGA codons in our study. Nevertheless, improvement in AzF and BocK incorporation was observed when increasing the tRNA copy number from one to four (Figures 3D and 3E), a strategy that has been shown to work previously (Chatterjee et al., 2013; Schmied et al., 2014)

Finally, we employed the two orthogonal aaRS/tRNA pairs to construct logic gates and demonstrated selective AND and OR logic operations dependent on AzF and BocK supplementation. These designs demonstrate that unnatural amino acids can be used as molecular switches in synthetic biology logic gates. The use of biologically inert unnatural amino acids (Chen et al., 2017; Han et al., 2017; Krogager et al., 2018; Liu et al., 2017; Suzuki et al., 2018) is ideal for constructing orthogonal, artificial pathways for synthetic biological applications with no identifiable side effects or non-specific activities. Moreover, the wide substrate scope of PylRS and TyrRS* (Nodling et al., 2019) enables fine-tuning the logic gate response for different applications.

To the best of our knowledge, this is the first example of unnatural amino acid-based logic gates in mammalian cells. Further exploration of quadruplet-decoding orthogonal tRNA in eukaryotic cells, as well as the underlying mechanism for their different performance in bacterial and eukaryotic systems, will provide new opportunities and applications for genetic code expansion. This will facilitate not only our understanding of protein structure and function but also the discovery and development of new diagnostics and therapeutics to target human diseases.

Significance

Mammalian cell logic gates remain heavily limited by the current approaches that involve the use of drug(-like) molecules that carry undesirable biological activities. In light of this, here we developed an alternative approach to mammalian cell logic gates via the simple process of genetic code expansion. By repurposing the cell translational machinery, we demonstrated the use of biologically inert unnatural amino acids as input switch molecules for effective and efficient mammalian cell logic operations, enabling selective and intrinsic control. The results shown here simultaneously expand the scope of genetic code expansion, while providing an exciting approach to mammalian cell logic gates.

Limitations of the study

Although this study expands the scope of mammalian cell logic circuits, it is important to note that this investigation employed human embryonic kidney (HEK293) cells and the tRNA variants might perform differently in other cell lines. Additionally, as with all genetic code expansion applications, amber and quadruplet suppression might not be limited to the gene of interest and

can potentially incorporate unnatural amino acids into other applicable codons in the host genome.

STAR★METHODS

Detailed methods are provided in the online version of this paper and include the following:

- KEY RESOURCES TABLE
- RESOURCE AVAILABILITY
  - Lead contact
  - Materials availability
  - Data and code availability
- EXPERIMENTAL MODEL AND SUBJECT DETAILS
- METHOD DETAILS
  - Vector cloning
  - Transient transfection
  - Imaging
  - Flow cytometry
  - Immunoblotting
- QUANTIFICATION AND STATISTICAL ANALYSIS

SUPPLEMENTAL INFORMATION

Supplemental information can be found online at https://doi.org/10.1016/j.crmeth.2021.100073.

ACKNOWLEDGMENTS

We are grateful for the financial support from BBSRC (BB/P009506/1), EPSRC (EP/T020687/1 and studentship to V.L.B.), and European Social Fund and Tenovus Cancer Care (Knowledge Economy Skills Scholarship to E.M.M.). We thank Prof. Dr. Anthony C.F. Perry for helpful discussions.

AUTHOR CONTRIBUTIONS

Conceptualization, E.M.M. and Y.H.T.; methodology, E.M.M., V.L.B., and Y.H.T.; validation, V.L.B.; formal analysis, E.M.M. and V.L.B.; investigation, E.M.M. and V.L.B.; writing, E.M.M., V.L.B., A.T.J., and Y.H.T.; supervision, A.T.J. and Y.H.T.; project administration, Y.H.T.; funding acquisition, A.T.J. and Y.H.T.

DECLARATION OF INTERESTS

The authors declare no competing interests.

Received: January 4, 2021
Revised: July 20, 2021
Accepted: August 13, 2021
Published: September 16, 2021

REFERENCES

Anderson, J.C., Wu, N., Santoro, S.W., Lakshman, V., King, D.S., and Schultz, P.G. (2004). An expanded genetic code with a functional quadruplet codon. Proc. Natl. Acad. Sci. U S A *101*, 7566–7571.

Bartoschek, M.D., Ugur, E., Nguyen, T.A., Rodschinka, G., Wierer, M., Lang, K., and Bultmann, S. (2021). Identification of permissive amber suppression sites for efficient non-canonical amino acid incorporation in mammalian cells. Nucleic Acids Res. *49*, e62.

Brown, W., Liu, J.H., and Deiters, A. (2018). Genetic code expansion in animals. ACS Chem. Biol. *13*, 2375–2386.

Cabantous, S., Terwilliger, T.C., and Waldo, G.S. (2005). Protein tagging and detection with engineered self-assembling fragments of green fluorescent protein. Nat. Biotechnol. *23*, 102–107.

Chatterjee, A., Xiao, H., Bollong, M., Ai, H.W., and Schultz, P.G. (2013). Efficient viral delivery system for unnatural amino acid mutagenesis in mammalian cells. Proc. Natl. Acad. Sci. U S A *110*, 11803–11808.

Chen, Y., Wan, Y., Wang, N., Yuan, Z., Niu, W., Li, Q., and Guo, J. (2018). Controlling the replication of a genomically recoded HIV-1 with a functional quadruplet codon in mammalian cells. ACS Synth. Biol. *7*, 1612–1617.

Chen, Y.T., Ma, J., Lu, W.Q., Tian, M.L., Thauvin, M., Yuan, C.G., Volovitch, M., Wang, Q., Holst, J., Liu, M.Y., et al. (2017). Heritable expansion of the genetic code in mouse and zebrafish. Cell Res. *27*, 294–297.

Chin, J.W. (2017). Expanding and reprogramming the genetic code. Nature *550*, 53–60.

Cuthbertson, L., and Nodwell, J.R. (2013). The TetR family of regulators. Microbiol. Mol. Biol. Rev. *77*, 440–475.

de la Torre, D., and Chin, J.W. (2021). Reprogramming the genetic code. Nat. Rev. Genet. *22*, 169–184.

Dumas, A., Lercher, L., Spicer, C.D., and Davis, B.G. (2015). Designing logical codon reassignment - expanding the chemistry in biology. Chem. Sci. *6*, 50–69.

Dunkelmann, D.L., Willis, J.C.W., Beattie, A.T., and Chin, J.W. (2020). Engineered triply orthogonal pyrrolysyl-tRNA synthetase/tRNA pairs enable the genetic encoding of three distinct non-canonical amino acids. Nat. Chem. *12*, 535–544.

Fink, T., Lonzaric, J., Praznik, A., Plaper, T., Merljak, E., Leben, K., Jerala, N., Lebar, T., Strmsek, Z., Lapenta, F., et al. (2019). Design of fast proteolysis-based signaling and logic circuits in mammalian cells. Nat. Chem. Biol. *15*, 115–122.

Gautier, A., Nguyen, D.P., Lusic, H., An, W.A., Deiters, A., and Chin, J.W. (2010). Genetically encoded photocontrol of protein localization in mammalian cells. J. Am. Chem. Soc. *132*, 4086–4088.

Han, S., Yang, A., Lee, S., Lee, H.W., Park, C.B., and Park, H.S. (2017). Expanding the genetic code of *Mus musculus*. Nat. Commun. *8*, 14568.

Huang, Y., and Liu, T. (2018). Therapeutic applications of genetic code expansion. Synth. Syst. Biotechnol. *3*, 150–158.

Italia, J.S., Latour, C., Wrobel, C.J.J., and Chatterjee, A. (2018). Resurrecting the bacterial tyrosyl-tRNA synthetase/tRNA pair for expanding the genetic code of both *E. coli* and Eukaryotes. Cell Chem Biol. *25*, 1304–1312.e5.

Ivanov, V., Beniaminov, A., Mikheyev, A., and Minyat, E. (2001). A mechanism for stop codon recognition by the ribosome: a bioinformatic approach. RNA *7*, 1683–1692.

Kamiyama, D., Sekine, S., Barsi-Rhyne, B., Hu, J., Chen, B., Gilbert, L.A., Ishikawa, H., Leonetti, M.D., Marshall, W.F., Weissman, J.S., et al. (2016). Versatile protein tagging in cells with split fluorescent protein. Nat. Commun. *7*, 11046.

Kato, Y. (2019). Translational control using an expanded genetic code. Int. J. Mol. Sci. *20*, 887.

Kim, J.H., Lee, S.R., Li, L.H., Park, H.J., Park, J.H., Lee, K.Y., Kim, M.K., Shin, B.A., and Choi, S.Y. (2011). High cleavage efficiency of a 2A peptide derived from porcine teschovirus-1 in human cell lines, zebrafish and mice. Plos One *6*, e18556.

Kitada, T., DiAndreth, B., Teague, B., and Weiss, R. (2018). Programming gene and engineered-cell therapies with synthetic biology. Science *359*, eaad1067.

Krogager, T.P., Ernst, R.J., Elliott, T.S., Calo, L., Beranek, V., Ciabatti, E., Spillantini, M.G., Tripodi, M., Hastings, M.H., and Chin, J.W. (2018). Labeling and identifying cell-specific proteomes in the mouse brain. Nat. Biotechnol. *36*, 156–159.

Li, Y., Wang, S., Chen, Y., Li, M., Dong, X., Hang, H.C., and Peng, T. (2020). Site-specific chemical fatty-acylation for gain-of-function analysis of protein S-palmitoylation in live cells. Chem. Commun. *56*, 13880–13883.

Liu, J., Hemphill, J., Samanta, S., Tsang, M., and Deiters, A. (2017). Genetic code expansion in zebrafish embryos and its application to optical control of cell signaling. J. Am. Chem. Soc. *139*, 9100–9103.

Meineke, B., Heimgartner, J., Eirich, J., Landreh, M., and Elsasser, S.J. (2020). Site-specific incorporation of two ncAAs for two-color bioorthogonal labeling and crosslinking of proteins on live mammalian cells. Cell Rep. *31*, 107811.

Meineke, B., Heimgartner, J., Lafranchi, L., and Elsasser, S.J. (2018). Methanomethylophilus alvus Mx1201 provides basis for mutual orthogonal pyrrolysyl tRNA/aminoacyl-tRNA synthetase pairs in mammalian cells. ACS Chem. Biol. *13*, 3087–3096.

Melnikov, S., Manakongtreecheep, K., and Soll, D. (2018). Revising the structural diversity of ribosomal proteins across the three domains of life. Mol. Biol. Evol. *35*, 1588–1598.

Monk, J.W., Leonard, S.P., Brown, C.W., Hammerling, M.J., Mortensen, C., Gutierrez, A.E., Shin, N.Y., Watkins, E., Mishler, D.M., and Barrick, J.E. (2017). Rapid and inexpensive evaluation of nonstandard amino acid incorporation in *Escherichia coli*. ACS Synth. Biol. *6*, 45–54.

Moore, B., Persson, B.C., Nelson, C.C., Gesteland, R.F., and Atkins, J.F. (2000). Quadruplet codons: implications for code expansion and the specification of translation step size. J. Mol. Biol. *298*, 195–209.

Neumann-Staubitz, P., and Neumann, H. (2016). The use of unnatural amino acids to study and engineer protein function. Curr. Opin. Struct. Biol. *38*, 119–128.

Neumann, H., Wang, K.H., Davis, L., Garcia-Alai, M., and Chin, J.W. (2010). Encoding multiple unnatural amino acids via evolution of a quadruplet-decoding ribosome. Nature *464*, 441–444.

Nguyen, D.P., Miyaoka, Y., Gilbert, L.A., Mayerl, S.J., Lee, B.H., Weissman, J.S., Conklin, B.R., and Wells, J.A. (2016). Ligand-binding domains of nuclear receptors facilitate tight control of split CRISPR activity. Nat. Commun. *7*, 12009.

Niu, W., Schultz, P.G., and Guo, J. (2013). An expanded genetic code in mammalian cells with a functional quadruplet codon. ACS Chem. Biol. *8*, 1640–1645.

Nodling, A.R., Spear, L.A., Williams, T.L., Luk, L.Y.P., and Tsai, Y.-H. (2019). Using genetically incorporated unnatural amino acids to control protein functions in mammalian cells. Essays Biochem. *63*, 237–266.

O'Connor, M. (2002). Insertions in the anticodon loop of tRNA1Gln(sufG) and tRNA(Lys) promote quadruplet decoding of CAAA. Nucleic Acids Res. *30*, 1985–1990.

Potts, K.A., Stieglitz, J.T., Lei, M., and Van Deventer, J.A. (2020). Reporter system architecture affects measurements of noncanonical amino acid incorporation efficiency and fidelity. Mol. Syst. Des. Eng. *5*, 573–588.

Ross, B., Mehta, S., and Zhang, J. (2016). Molecular tools for acute spatiotemporal manipulation of signal transduction. Curr. Opin. Chem. Biol. *34*, 135–142.

Scheller, L., and Fussenegger, M. (2019). From synthetic biology to human therapy: engineered mammalian cells. Curr. Opin. Biotechnol. *58*, 108–116.

Schmied, W.H., Elsasser, S.J., Uttamapinant, C., and Chin, J.W. (2014). Efficient multisite unnatural amino acid incorporation in mammalian cells via optimized pyrrolysyl tRNA synthetase/tRNA expression and engineered eRF1. J. Am. Chem. Soc. *136*, 15577–15583.

Singh, V. (2014). Recent advances and opportunities in synthetic logic gates engineering in living cells. Syst. Synth. Biol. *8*, 271–282.

Suzuki, T., Asami, M., Patel, S.G., Luk, L.Y.P., Tsai, Y.-H., and Perry, A.C.F. (2018). Switchable genome editing via genetic code expansion. Sci. Rep. *8*, 10051.

Suzuki, T., Miller, C., Guo, L.T., Ho, J.M.L., Bryson, D.I., Wang, Y.S., Liu, D.R., and Soll, D. (2017). Crystal structures reveal an elusive functional domain of pyrrolysyl-tRNA synthetase. Nat. Chem. Biol. *13*, 1261–1266.

Taki, M., Matsushita, J., and Sisido, M. (2006). Expanding the genetic code in a mammalian cell line by the introduction of four-base codon/anticodon pairs. ChemBioChem *7*, 425–428.

Takimoto, J.K., Adams, K.L., Xiang, Z., and Wang, L. (2009). Improving orthogonal tRNA-synthetase recognition for efficient unnatural amino acid incorporation and application in mammalian cells. Mol. Biosyst. *5*, 931–934.

Tharp, J.M., Ehnbom, A., and Liu, W.R. (2018). tRNA(Pyl): structure, function, and applications. RNA Biol. *15*, 441–452.

Wang, K., Sachdeva, A., Cox, D.J., Wilf, N.M., Lang, K., Wallace, S., Mehl, R.A., and Chin, J.W. (2014). Optimized orthogonal translation of unnatural amino acids enables spontaneous protein double-labelling and FRET. Nat. Chem. *6*, 393–403.

Wang, N., Shang, X., Cerny, R., Niu, W., and Guo, J. (2016). Systematic evolution and study of UAGN decoding tRNAs in a genomically recoded bacteria. Sci. Rep. *6*, 21898.

Willis, J.C.W., and Chin, J.W. (2018). Mutually orthogonal pyrrolysyl-tRNA synthetase/tRNA pairs. Nat. Chem. *10*, 831–837.

Wu, C.Y., Roybal, K.T., Puchner, E.M., Onuffer, J., and Lim, W.A. (2015). Remote control of therapeutic T cells through a small molecule-gated chimeric receptor. Science *350*, aab4077.

Xiao, H., Chatterjee, A., Choi, S.H., Bajjuri, K.M., Sinha, S.C., and Schultz, P.G. (2013). Genetic incorporation of multiple unnatural amino acids into proteins in mammalian cells. Angew. Chem. Int. Ed. *52*, 14080–14083.

Yanagisawa, T., Ishii, R., Fukunaga, R., Kobayashi, T., Sakamoto, K., and Yokoyama, S. (2008). Multistep engineering of pyrrolysyl-tRNA synthetase to genetically encode N(epsilon)-(o-azidobenzyloxycarbonyl) lysine for site-specific protein modification. Chem. Biol. *15*, 1187–1197.

Zetsche, B., Volz, S.E., and Zhang, F. (2015). A split-Cas9 architecture for inducible genome editing and transcription modulation. Nat. Biotechnol. *33*, 139–142.

Zheng, Y., Addy, P.S., Mukherjee, R., and Chatterjee, A. (2017). Defining the current scope and limitations of dual noncanonical amino acid mutagenesis in mammalian cells. Chem. Sci. *8*, 7211–7217.

Zheng, Y., Mukherjee, R., Chin, M.A., Igo, P., Gilgenast, M.J., and Chatterjee, A. (2018). Expanding the scope of single- and double-noncanonical amino acid mutagenesis in mammalian cells using orthogonal polyspecific leucyl-tRNA synthetases. Biochemistry *57*, 441–445.

Zhou, W., Smidlehner, T., and Jerala, R. (2020). Synthetic biology principles for the design of protein with novel structures and functions. FEBS Lett. *594*, 2199–2212.

STAR★METHODS

KEY RESOURCES TABLE

REAGENT or RESOURCE	SOURCE	IDENTIFIER
**Antibodies**		
α-eGFP	ThermoFisher	#MA1-952; RRID:AB_889471
α-mouse	ThermoFisher	#32430; RRID:AB_1185566
α-GAPDH	ThermoFisher	#MA5-15738; RRID:AB_10977387
α-FLAG	Sigma	#F3165; RRID:AB_259529
**Bacterial and virus strains**		
Escherichia coli OneShot Stbl3	ThermoFisher	Cat#C737303
**Chemicals, peptides, and recombinant proteins**		
BocK	Fluorochem	Cat#078520
AzF	Bachem	Cat#4020250
OMeY	Alfa Aesar	H63096
**Deposited data**		
Biological triplicates of flow cytometry results (*.fcs) available in depository	This paper	https://doi.org/10.17035/d.2021.0135747960
**Experimental models: Cell lines**		
HEK293	ECACC General Collection	Cat#85120602; RRID:CVCL_0045
**Oligonucleotides**		
See Table S1 in the supplemental information	Merck Life Sciences	VC00021
**Recombinant DNA**		
See vector cloning section below and the supplemental information for vector sequences STAR methods	This paper	n/a
Vector 58: Pyl $tRNA_{CUA(U25C)}$ PylRS mCherry-P2A-eGFP(150TAG)	This paper	Addgene Plasmid #174526
Vector 62: Pyl $tRNA_{CUAG}$ PylRS mCherry-P2A-eGFP(150CTAG)	This paper	Addgene Plasmid #174527
Vector 66: Pyl $tRNA_{UCCU(Ev2)}$ PylRS mCherry-P2A-eGFP(150AGGA)	This paper	Addgene Plasmid #174528
Vector 73: 4x(Pyl $tRNA_{UCCU(Ev2)}$) PylRS mCherry-P2A-eGFP(150AGGA)	This paper	Addgene Plasmid #174529
**Software and algorithms**		
FlowJo	BD Biosciences	RRID:SCR_008520
ZEN	Zeiss	RRID:SCR_013672
Image Lab	Bio-Rad	RRID:SCR_014210
**Other**		
Dulbecco's Modified Eagle Medium with GlutaMAX supplement	Fisher Scientific	#11574516
Fetal bovine serum	Fisher Scientific	#11573397
Trypsin-EDTA (0.25%)	Fisher Scientific	#11560626
Dulbecco's Phosphate Buffered Saline	Sigma	#D1408
Lipofectamine 2000	Life Technologies	#10696343
Opti-MEM	Fisher Scientific	#11058021

CellPress
OPEN ACCESS

RESOURCE AVAILABILITY

Lead contact

Further information and requests for resources and reagents should be directed to and will be fulfilled by the lead contact, Yu-Hsuan Tsai (Tsai.Y-H@outlook.com).

Materials availability

Full DNA sequences for each vector used in the main manuscript can be found in the Supporting Information. Plasmids generated in this study have been deposited to Addgene: 174526 for vector 58 – Pyl tRNA$_{CUA(U25C)}$ PylRS mCherry-P2A-eGFP(150TAG); 174527 for vector 62 – Pyl tRNA$_{CUAG}$ PylRS mCherry-P2A-eGFP(150CTAG); 174528 for vector 66 – Pyl tRNA$_{UCCU(Ev2)}$ PylRS mCherry-P2A-eGFP(150AGGA); 174529 for vector 73 – 4x(Pyl tRNA$_{UCCU(Ev2)}$) PylRS mCherry-P2A-eGFP(150AGGA).

Data and code availability

- All FACS data have been deposited to the Cardiff University data catalogue and are publicly available as of the date of publication. DOIs are listed in the key resources table. Microscopy data reported in this paper will be shared by the lead contact upon request.
- This paper does not report original code.
- Any additional information required to reanalyse the data reported in this paper is available from the lead contact upon request.

EXPERIMENTAL MODEL AND SUBJECT DETAILS

HEK293 cells were obtained from ECACC (ECACC General Collection, #85120602) and routinely tested for mycoplasma infection. Cells were maintained in T75 flasks at 37 °C in a 5% CO_2 high-humidity atmosphere in Dulbecco's Modified Eagle Medium (DMEM) with GlutaMAX supplement (Fisher Scientific, #11574516) supplemented with 10% (v/v) fetal bovine serum (FBS; Fisher Scientific, #11573397). Cells were maintained at a sub-confluent monolayer and split at 80-85% confluency. For splitting, cells were washed with Dulbecco's phosphate buffered saline (DPBS; Sigma, #D1408), detached in 1 mL of 0.25% trypsin-EDTA (Fisher Scientific, #11560626) and 200 μL of the 1000 μL trypsin cell suspension was re-suspended in 12 mL fresh DMEM containing 10% (v/v) FBS in a new T75 flask.

METHOD DETAILS

Vector cloning

All Vector stocks were generated and maintained via transformation of chemically competent One Shot^{TM} Stbl3^{TM} *E. coli* cells from ThermoFisher (ThermoFisher, #C737303) and isolated from 5 mL cultures using QIAprep Spin Miniprep Kit (Qiagen, #27106) following the manufacturer's protocol. Where appropriate, restriction digests were conducted using FastDigest restriction enzymes (Thermo Scientific) following manufacturer's protocol. PCR was conducted in a thermocycler using PrimeSTAR Max (Takara, #R045A) following the manufacturer's protocol. Restriction digests and PCR products were electrophoresed on 1% agarose gels in TAE buffer (40 mM Tris pH 7.6, 20 mM acetic acid, 1 mM EDTA) and visualised using SYBRSafe DNA stain (Invitrogen, #S33102). Desired bands were excised from the gel and extracted using QIAquick Gel Extraction Kit (Qiagen, #28704) following the manufacturer's protocol. T4 ligations were performed using T4 DNA Ligase (ThermoFisher, #EL0014) and Gibson assembly via NEBuilder HiFi DNA Assembly (New England Biolabs, #E5520) following respective manufacturers protocols. All constructs were confirmed via Sanger sequencing. A list of primers used in this study is provided in Table S1.

Vector 1: Pyl tRNA$_{CUA(U25C)}$

This Vector was purchased from GeneArt (ThermoFisher). The full DNA sequence can be found in the supplemental information.

Vector 2: 4xPyl tRNA$_{CUA(U25C)}$-PylRS-eGFP(150TAG)

This Vector is pEF1α-FLAGPylRS-CAG-eGFP(150TAG)-4xU6-PylTU25C (Ref: 10.1038/s41598-018-28178-3).

Vector 3: Pyl tRNA$_{CUA(U25C)}$-PylRS-eGFP(150TAG)

Vector 2 was cut with the restriction enzyme Eco147i to generate the vector. Insert generated via PCR of **Vector 2** using **Primer 1** and **Primer 2**. The vector and insert were combined using Gibson Assembly.

Vector 4: pCAG-eGFP

This Vector is pCAG-eGFP (Ref: Perry, A. C. F. *et al.* Mammalian transgenesis by intracytoplasmic sperm injection. *Science* **284**, 1180–1183 (1999).)

Vector 5: eGFP(150TAGA)

Cloned by PCR mutagenesis of **Vector 4** using **Primer 3** and **Primer 4**. Template **Vector 4** removed from reaction mixture via DpnI restriction enzyme digest.

Vector 6: eGFP(150CTAG)
Cloned by PCR mutagenesis of **Vector 4** using **Primer 3** and **Primer 5**. Template **Vector 4** removed from reaction mixture via DpnI restriction enzyme digest.
Vector 7: eGFP(150AGGA)
Cloned by PCR mutagenesis of **Vector 4** using **Primer 3** and **Primer 6**. Template **Vector 4** removed from reaction mixture via DpnI restriction enzyme digest.
Vector 8: eGFP(150TAGT)
Cloned by PCR mutagenesis of **Vector 4** using **Primer 3** and **Primer 7**. Template **Vector 4** removed from reaction mixture via DpnI restriction enzyme digest.
Vector 9: eGFP(150TAGC)
Cloned by PCR mutagenesis of **Vector 4** using **Primer 3** and **Primer 8**. Template **Vector 4** removed from reaction mixture via DpnI restriction enzyme digest.
Vector 10: eGFP(150TAGG)
Cloned by PCR mutagenesis of **Vector 4** using **Primer 3** and **Primer 9**. Template **Vector 4** removed from reaction mixture via DpnI restriction enzyme digest.
Vector 11: Pyl tRNA$_{CUA(U25C)}$-PylRS-eGFP(150TAGA)
Vector generated via digestion of **Vector 3** with the restriction enzymes BglII and XbaI. Insert generated via digestion of **Vector 5** with BglII and XbaI. The vector and insert fragments were assembled by T4 ligation.
Vector 12: Pyl tRNA$_{CUA(U25C)}$-PylRS-eGFP(150CTAG)
Vector generated via digestion of **Vector 3** with restriction enzymes BglII and XbaI. Insert generated via digestion of **Vector 6** with BglII and XbaI. The vector and insert fragments were assembled by T4 ligation.
Vector 13: Pyl tRNA$_{CUA(U25C)}$-PylRS-eGFP(150AGGA)
Vector generated via digestion of **Vector 3** with restriction enzymes BglII and XbaI. Insert generated via digestion of **Vector 7** with BglII and XbaI. The vector and insert fragments were assembled by T4 ligation.
Vector 14: Pyl tRNA$_{CUA(U25C)}$-PylRS-eGFP(150TAGT)
Vector generated via digestion of **Vector 3** with the restriction enzymes BglII and XbaI. Insert generated via digestion of **Vector 8** with BglII and XbaI. The vector and insert fragments were assembled by T4 ligation.
Vector 15: Pyl tRNA$_{CUA(U25C)}$-PylRS-eGFP(150TAGC)
Vector generated via digestion of **Vector 3** with the restriction enzymes BglII and XbaI. Insert generated via digestion of **Vector 9** with BglII and XbaI. The vector and insert fragments were assembled by T4 ligation.
Vector 16: Pyl tRNA$_{CUA(U25C)}$-PylRS-eGFP(150TAGT)
Vector generated via digestion of **Vector 3** with the restriction enzymes BglII and XbaI. Insert generated via digestion of **Vector 10** with BglII and XbaI. The vector and insert fragments were assembled by T4 ligation.
Vector 17: Pyl tRNA$_{UCUA}$
Cloned by PCR mutagenesis of **Vector 1** using **Primer 10** and **Primer 11**. The template **Vector 1** was removed from the reaction mixture via DpnI restriction enzyme digest.
Vector 18: Pyl tRNA$_{UCUA(Ev1)}$
Cloned by PCR mutagenesis of **Vector 1** using **Primer 10** and **Primer 12**. The template **Vector 1** was removed from the reaction mixture via DpnI restriction enzyme digest.
Vector 19: Pyl tRNA$_{UCUA(Ev2)}$
Cloned by PCR mutagenesis of **Vector 1** using **Primer 10** and **Primer 13**. The template **Vector 1** was removed from the reaction mixture via DpnI restriction enzyme digest.
Vector 20: Pyl tRNA$_{CUAG}$
Cloned by PCR mutagenesis of **Vector 1** using **Primer 10** and **Primer 14**. The template **Vector 1** was removed from the reaction mixture via DpnI restriction enzyme digest.
Vector 21: Pyl tRNA$_{CUAG(Ev1)}$
Cloned by PCR mutagenesis of **Vector 1** using **Primer 10** and **Primer 15**. The template **Vector 1** was removed from the reaction mixture via DpnI restriction enzyme digest.
Vector 22: Pyl tRNA$_{UCCU}$
Cloned by PCR mutagenesis of **Vector 1** using **Primer 10** and **Primer 16**. The template **Vector 1** was removed from the reaction mixture via DpnI restriction enzyme digest.
Vector 23: Pyl tRNA$_{UCCU(Ev1)}$
Cloned by PCR mutagenesis of **Vector 1** using **Primer 10** and **Primer 17**. The template **Vector 1** was removed from the reaction mixture via DpnI restriction enzyme digest.
Vector 24: Pyl tRNA$_{UCCU(Ev2)}$
Cloned by PCR mutagenesis of **Vector 1** using **Primer 10** and **Primer 18**. The template **Vector 1** was removed from the reaction mixture via DpnI restriction enzyme digest.

Vector 25: Pyl tRNA$_{ACUA}$
Cloned by PCR mutagenesis of **Vector 1** using **Primer 10** and **Primer 19**. The template **Vector 1** was removed from the reaction mixture via DpnI restriction enzyme digest.
Vector 26: Pyl tRNA$_{GCUA}$
Cloned by PCR mutagenesis of **Vector 1** using **Primer 10** and **Primer 20**. The template **Vector 1** was removed from the reaction mixture via DpnI restriction enzyme digest.
Vector 27: Pyl tRNA$_{CCUA}$
Cloned by PCR mutagenesis of **Vector 1** using **Primer 10** and **Primer 21**. The template **Vector 1** was removed from the reaction mixture via DpnI restriction enzyme digest.
Vector 28: Pyl tRNA$_{UCUA}$-PylRS-eGFP(150TAGA)
Vector generated by digesting **Vector 11** with the restriction enzymes Eco147i and AgeI. Insert generated from restriction digestion of **Vector 17** using Eco147i and AgeI. The vector and insert fragments were assembled by T4 ligation.
Vector 29: Pyl tRNA$_{UCUA(Ev1)}$-PylRS-eGFP(150TAGA)
Vector generated by digesting **Vector 11** with the restriction enzymes Eco147i and AgeI. Insert generated from restriction digestion of **Vector 18** using Eco147i and AgeI. The vector and insert fragments were assembled by T4 ligation.
Vector 30: Pyl tRNA$_{UCUA(Ev2)}$-PylRS-eGFP(150TAGA)
Vector generated by digesting **Vector 11** with the restriction enzymes Eco147i and AgeI. Insert generated from restriction digestion of **Vector 19** using Eco147i and AgeI. The vector and insert fragments were assembled by T4 ligation.
Vector 31: Pyl tRNA$_{CUAG}$-PylRS-eGFP(150CTAG)
Vector generated by digesting **Vector 12** with the restriction enzymes Eco147i and AgeI. Insert generated from restriction digestion of **Vector 20** using Eco147i and AgeI. The vector and insert fragments were assembled by T4 ligation.
Vector 32: Pyl tRNA$_{CUAG(Ev1)}$-PylRS-eGFP(150CTAG)
Vector generated by digesting **Vector 12** with the restriction enzymes Eco147i and AgeI. Insert generated from restriction digestion of **Vector 21** using Eco147i and AgeI. The vector and insert fragments were assembled by T4 ligation.
Vector 33: Pyl tRNA$_{UCCU}$-PylRS-eGFP(150AGGA)
Vector generated by digesting **Vector 13** with the restriction enzymes Eco147i and AgeI. Insert generated from restriction digestion of **Vector 22** using Eco147i and AgeI. The vector and insert fragments were assembled by T4 ligation.
Vector 34: Pyl tRNA$_{UCCU(Ev1)}$-PylRS-eGFP(150AGGA)
Vector generated by digestion of **Vector 13** with restriction enzymes Eco147i and AgeI. Insert generated from restriction digestion of **Vector 23** using Eco147i and AgeI. The vector and insert fragments were assembled by T4 ligation.
Vector 35: Pyl tRNA$_{UCCU(Ev2)}$-PylRS-eGFP(150AGGA)
Vector generated by digesting **Vector 13** with restriction enzymes Eco147i and AgeI. Insert generated from restriction digestion of **Vector 24** using Eco147i and AgeI. The vector and insert fragments were assembled by T4 ligation.
Vector 36: Pyl tRNA$_{ACUA}$-PylRS-eGFP(150TAGT)
Vector generated by digesting **Vector 14** with restriction enzymes Eco147i and AgeI. Insert generated from restriction digestion of **Vector 25** using Eco147i and AgeI. The vector and insert fragments were assembled by T4 ligation.
Vector 37: Pyl tRNA$_{GCUA}$-PylRS-eGFP(150TAGC)
Vector generated by digesting **Vector 15** with restriction enzymes Eco147i and AgeI. Insert generated from restriction digestion of **Vector 26** using Eco147i and AgeI. The vector and insert fragments were assembled by T4 ligation.
Vector 38: Pyl tRNA$_{CCUA}$-PylRS-eGFP(150TAGG)
Vector generated by digesting **Vector 16** with restriction enzymes Eco147i and AgeI. Insert generated from restriction digestion of **Vector 27** using Eco147i and AgeI. The vector and insert fragments were assembled by T4 ligation.
Vector 39: (U6-Tyr tRNA$_{CUA}$)x2(H1-Tyr tRNA$_{CUA}$)x2 TyrRS*-eGFP(40TAG)
This Vector was purchased from Addgene (#50831).
Vector 40: Tyr tRNA$_{CUA}$-PylRS-eGFP(150TAG)
Vector generated by digesting **Vector 3** with restriction enzymes Eco147i and AgeI. Insert generated via PCR of **Vector 39** using **Primer 22** and **Primer 23**. The vector and insert were combined using Gibson assembly.
Vector 41: Tyr tRNA$_{CUA}$-TyrRS*-eGFP(150TAG)
Vector generated by digesting **Vector 40** with restriction enzymes NheI and BamHI. Insert generated via PCR of **Vector 39** using **Primer 24** and **Primer 25**. The vector and insert were combined using Gibson assembly.
Vector 42: (Pyl tRNA$_{UCCU\ (Ev2)}$)x4-PylRS-eGFP(150AGGA)
Vector generated by digesting **Vector 35** with Eco147i restriction enzyme. Thermosensitive Alkaline Phosphotase (TAP) was included in reaction mixture to remove 5’ and 3’ phosphate groups and prevent re-ligation. The three inserts were generated via PCR of **Vector 35** using three distinctive pairs of primers: **Primer 26** and **Primer 27**; **Primer 28** and **Primer 29**; and **Primer 30** and **Primer 31**. The vector and inserts were combined using Gibson assembly.
Vector 43: (Tyr tRNA$_{CUA}$)x4-TyrRS*-eGFP(150TAG)
Vector generated by digesting **Vector 41** with Eco147i restriction enzyme. Thermosensitive Alkaline Phosphotase (TAP) was included in reaction mixture to remove 5’ and 3’ phosphate groups and prevent re-ligation. The three inserts were generated via PCR of **Vector**

41 using three distinctive pairs of primers: **Primer 32** and **Primer 33**; **Primer 34** and **Primer 35**; and **Primer 36** and **Primer 37**. The vector and inserts were combined using Gibson assembly.

Vector 44: eRF1(E55D)

This was a kind gift from Jason Chin (Schmied et al., 2014).

Vector 45: eGFP(40TAG,150AGGA)

Vector generated by PCR of **Vector 7** using **Primer 38** and **Primer 39**. Insert generated by PCR of **Vector 7** using **Primer 40** and **Primer 41**. The vector and insert were combined using Gibson assembly.

Vector 46: Pyl tRNA$_{UCCU(Ev2)}$-PylRS-eGFP(40TAG,150AGGA)

Vector generated by digesting **Vector 35** using restriction enzymes BglII and EcoRI. Insert generated by restriction digest of **Vector 45** using BglII and EcoRI. Both the vector and insert isolated via TAE agarose gel electrophoresis and gel extraction. The vector and insert fragments were assembled by T4 ligation.

Vector 47: Tyr tRNA$_{CUA}$-TyrRS*

Vector generated by digesting **Vector 41** with dual cutter restriction enzyme EcoRI and isolating larger fragment. The DNA was re-ligated via T4 ligation.

Vector 48: (Pyl tRNA$_{UCCU(Ev2)}$)x4-PylRS-eGFP(40TAG,150AGGA)

Vector generated by digesting **Vector 42** using restriction enzymes BglII and XbaI. Insert generated by restriction digest of **Vector 45** using BglII and XbaI. The vector and insert fragments were assembled by T4 ligation.

Vector 49: (Tyr tRNA$_{CUA}$)x4-TyrRS*-eGFP(40TAG,150AGGA)

Vector generated by digesting **Vector 43** using restriction enzymes BglII and XbaI. Insert generated by restriction digest of **Vector 45** using BglII and XbaI. The vector and insert fragments were assembled by T4 ligation.

Vector 50: mCherry

This was constructed from pCAG-mCherry.

Vector 51: mCherry-P2A-sGFP(1-10)

Vector generated by digesting **Vector 50** with restriction enzymes BsrGI and BglII. Insert composed a gene strings fragment encoding C-terminal residues of mCherry, P2A linker and sfGFP(1-10) and was ordered from GeneArt (ThermoFisher). Vector and insert combined via Gibson assembly.

Vector 52: sGFP(11)

This Vector was purchased from GeneArt (ThermoFisher).

Vector 53: mCherry-P2A-sGFP(11)

Vector generated by digesting **Vector 51** with MluI and BglII. Insert generated by digestion of **Vector 52** with MluI and BglII. Vector and insert were assembled via T4 ligation.

Vector 54: mCherry-P2A-TAG-sGFP(1-10)

Vector generated by digesting **Vector 51** with PshAI and BglII. Insert generated by PCR of **Vector 51** with **Primer 42** and **Primer 43**. The vector and insert were combined using Gibson assembly.

Vector 55: (Tyr tRNA$_{CUA}$)x4-TyrRS*-mCherry-P2A-TAG-sGFP(1-10)

Vector generated by restriction digestion of **Vector 43** using BglII and XbaI. Insert generated by restriction digest of **Vector 54** with BglII and XbaI. The vector and insert were combined using T4 ligation.

Vector 56: (Pyl tRNA$_{UCCU(Ev2)}$)x4-PylRS-mCherry-P2A-AGGA-sGFP(11)

Vector generated by restriction digest of **Vector 42** with XbaI and BglII. Insert 1 generated by restriction digest of **Vector 51** with PshAI and XbaI. Insert 2 generated by PCR of **Vector 53** using **Primer 44** and **Primer 45**. Insert 2 subjected to restriction digestion with PshAI and BglII to generate sticky ends. The three fragments were combined by T4 ligation.

Vector 57: (Tyr tRNA$_{CUA}$)x4-TyrRS*-sGFP(1-10)-P2A-TAG-sGFP(11)

Vector generated by digesting **Vector 43** with XbaI and BglII. Insert 1 generated by PCR of **Vector 53** with **Primer 46** and **Primer 47**. Insert 2 generated by PCR of **Vector 51** with **Primer 48** and **Primer 49**. The three fragments were combined via Gibson Assembly.

Vector 58: Pyl tRNA$_{CUA(U25C)}$-PylRS-mCherry-P2A-eGFP(150TAG)

Vector 3 digested with NheI and BamHI to generate the vector. Insert 1 generated by PCR of **Vector 3** with **Primer 50** and **Primer 51**. Insert 2 generated by PCR of **Vector 51** with **Primer 52** and **Primer 53**. The three fragments were combined via Gibson assembly.

Vector 59: Pyl tRNA$_{UCUA}$-PylRS-mCherry-P2A-eGFP(150TAGA)

Vector 28 digested with EcoRI and BglII to generate vector. Insert 1 generated by PCR of **Vector 28** with **Primer 50** and **Primer 51.** Insert 2 generated by PCR of **Vector 51** with **Primer 52** and **Primer 53**. The three fragments were combined via Gibson assembly.

Vector 60: Pyl tRNA$_{UCUA(Ev1)}$-PylRS-mCherry-P2A-eGFP(150TAGA)

Vector 29 digested with EcoRI and BglII to generate vector. Insert 1 generated by PCR of **Vector 29** with **Primer 50** and **Primer 51.** Insert 2 generated by PCR of **Vector 51** with **Primer 52** and **Primer 53**. The three fragments were combined via Gibson assembly.

Vector 61: Pyl tRNA$_{UCUA(Ev2)}$-PylRS-mCherry-P2A-eGFP(150TAGA)

Vector 30 digested with EcoRI and BglII to generate vector. Insert 1 generated by PCR of **Vector 30** with **Primer 50** and **Primer 51.** Insert 2 generated by PCR of **Vector 51** with **Primer 52** and **Primer 53**. The three fragments were combined via Gibson assembly.

Vector 62: Pyl tRNA$_{CUAG}$-PylRS-mCherry-P2A-eGFP(150CTAG)
Vector 31 digested with EcoRI and BglII to generate vector. Insert 1 generated by PCR of **Vector 31** with **Primer 50** and **Primer 51.** Insert 2 generated by PCR of **Vector 51** with **Primer 52** and **Primer 53**. The three fragments were combined via Gibson assembly.
Vector 63: Pyl tRNA$_{CUAG(Ev1)}$-PylRS-mCherry-P2A-eGFP(150CTAG)
Vector 32 digested with EcoRI and BglII to generate vector. Insert 1 generated by PCR of **Vector 32** with **Primer 50** and **Primer 51.** Insert 2 generated by PCR of **Vector 51** with **Primer 52** and **Primer 53**. The three fragments were combined via Gibson assembly.
Vector 64: Pyl tRNA$_{UCCU}$-PylRS-mCherry-P2A-eGFP(150AGGA)
Vector 33 digested with EcoRI and BglII to generate vector. Insert 1 generated by PCR of **Vector 33** with **Primer 50** and **Primer 51.** Insert 2 generated by PCR of **Vector 51** with **Primer 52** and **Primer 53**. The three fragments were combined via Gibson assembly.
Vector 65: Pyl tRNA$_{UCCU(Ev1)}$-PylRS-mCherry-P2A-eGFP(150AGGA)
Vector 34 digested with EcoRI and BglII to generate vector. Insert 1 generated by PCR of **Vector 34** with **Primer 50** and **Primer 51.** Insert 2 generated by PCR of **Vector 51** with **Primer 52** and **Primer 53**. The three fragments were combined via Gibson assembly.
Vector 66: Pyl tRNA$_{UCCU(Ev2)}$-PylRS-mCherry-P2A-eGFP(150AGGA)
Vector 35 digested with EcoRI and BglII to generate vector. Insert 1 generated by PCR of **Vector 35** with **Primer 50** and **Primer 51.** Insert 2 generated by PCR of **Vector 51** with **Primer 52** and **Primer 53**. The three fragments were combined via Gibson assembly.
Vector 67: Pyl tRNA$_{ACUA}$-PylRS-mCherry-P2A-eGFP(150TAGT)
Vector 36 digested with EcoRI and BglII to generate vector. Insert 1 generated by PCR of **Vector 36** with **Primer 50** and **Primer 51.** Insert 2 generated by PCR of **Vector 51** with **Primer 52** and **Primer 53**. The three fragments were combined via Gibson assembly.
Vector 68: Pyl tRNA$_{CCUA}$-PylRS-mCherry-P2A-eGFP(150TAGG)
Vector 38 digested with EcoRI and BglII to generate vector. Insert 1 generated by PCR of **Vector 38** with **Primer 50** and **Primer 51.** Insert 2 generated by PCR of **Vector 51** with **Primer 52** and **Primer 53**. The three fragments were combined via Gibson assembly.
Vector 69: Pyl tRNA$_{GCUA}$-PylRS-mCherry-P2A-eGFP(150TAGC)
Vector 37 digested with EcoRI and BglII to generate vector. Insert 1 generated by PCR of **Vector 37** with **Primer 50** and **Primer 51.** Insert 2 generated by PCR of **Vector 51** with **Primer 52** and **Primer 53**. The three fragments were combined via Gibson assembly.
Vector 70: Pyl tRNA$_{CUA}$-PylRS-mCherry-P2A-eGFP
Vector 3 digested with EcoRI and BglII to generate the vector. Insert 1 generated by PCR of **Vector 51** with **Primer 52** and **Primer 53**. Insert 2 generated by PCR of **Vector 4** with **Primer 50** and **Primer 51**. The three fragments were combined via Gibson Assembly.
Vector 71: Tyr tRNA$_{CUA}$-TyrRS*-mCherry-P2A-eGFP(150TAG)
Vector 41 digested with EcoRI and BglII to generate vector. Insert 1 generated by PCR of **Vector 51** with **Primer 52** and **Primer 53**. Insert 2 generated by PCR of **Vector 41** with **Primer 50** and **Primer 51**. The three fragments were combined via Gibson Assembly.
Vector 72: Tyr tRNA$_{CUA}$-EAziRS-mCherry-P2A-eGFP(150TAG)
Vector 71 digested with BamHI and HindIII to generate vector. Insert 1 generated by PCR of **Vector 71** with **Primer 54** and **Primer 55**. Insert 2 generated by PCR of **Vector 71** with **Primer 56** and **Primer 57**. Insert 3 generated by PCR of **Vector 71** with **Primer 58** and **Primer 59**. The three fragments were combined via Gibson Assembly.
Vector 73: (Pyl tRNA$_{UCCU\ (Ev2)}$)x4-PylRS-mCherry-P2A-eGFP(150AGGA)
Vector 42 digested with EcoRI and BglII to generate vector. Insert 1 generated by PCR of **Vector 51** with **Primer 52** and **Primer 53**. Insert 2 generated by PCR of **Vector 42** with **Primer 50** and **Primer 51**. The three fragments were combined via Gibson Assembly.
Vector 74: (Tyr tRNA$_{CUA}$)x4-TyrRS*-mCherry-P2A-eGFP(150TAG)
Vector 43 digested with EcoRI and BglII to generate vector. Insert 1 generated by PCR of **Vector 51** with **Primer 52** and **Primer 53**. Insert 2 generated by PCR of **Vector 43** with **Primer 50** and **Primer 51**. The three fragments were combined via Gibson Assembly.

Transient transfection

HEK293 cells were plated at a $1x10^6$ cells per well of a 24 well plate (Corning, #10380932) in DMEM (Fisher Scientific, #11574516) supplemented with 10% (v/v) FBS (Fisher Scientific, #11573397) and maintained at 37 °C in a high-humidity 5% CO_2 atmosphere for 24 h or until 90% confluent. For transfection, per well, 1.5 μL Lipofectamine 2000 (Life Technologies, #10696343) was suspended in 50 μL Opti-MEM (Fisher Scientific, #11058021), and incubated for 10 min at room temperature. 500 ng of Vector was diluted in 50 μL Opti-MEM and mixed with the lipofectamine/Opti-MEM solution to a final volume of 100 μL and incubated for 30 minutes at room temperature. Media in each well was exchanged for fresh DMEM/10% FBS and, when appropriate, supplemented with 100 mM stock of BocK (Fluorochem, #078520) and/or AzF (Bachem, #4020250) or OMeY (Alfa Aesar, #H63096) to final working concentrations of 1 mM. Vector-lipofectamine solution was then added drop-wise to the well, and the plate incubated at 37 °C in a high-humidity 5% CO_2 atmosphere for 48 h.

Imaging

Cells were imaged using Zeiss AxioCam MRm microscope camera and ZEN (version 2.3) computer imaging programme. All images were taken at 200X magnification, and representative regions of the entire well were captured. GFP fluorescence was detected with Zeiss FSet 38 green fluorescence filter (excitation 470/40 and emission 525/50), with a constant exposure of 550 ms per image. mCherry fluorescence was detected with Zeiss FSet 45 (excitation 560/40 and emission 630/75), with a constant

exposure of 550 ms per image. To analyse dual-fluorescence reporters, GFP and mCherry fluorescent images were composited and the resulting images annotated as ‘Merge’.

Flow cytometry

After transfection, media was aspirated from wells and cells washed with 100 μL DPBS. Then, 50 μL of trypsin was added to each well and the plate incubated at 37 °C, 5% CO_2 for 5 minutes. The plate was sharply tapped to ensure cellular detachment and cells were resuspended in 450 μL DPBS and filtered through a CellTrics® 50 μM filter (Sysmex #04-0042-2317) into a round-bottom flow cytometry tube. Flow cytometry was performed using an S3e cell sorter (Bio-Rad). For each condition, acquisition was allowed to continue until at least 10,000 mCherry-positive cells had been analysed (see quantification and statistical analysis). Each experiment was performed in triplicate.

Immunoblotting

Immediately following microscopical analysis the media was removed from wells and cells washed twice with 1x PBS. 50 μL of RIPA Buffer (Sigma-Aldrich, #R0278) containing 1% v/v protease inhibitor cocktail (Sigma-Aldrich, #P8340) was added dropwise to the centre of the well and plate incubated on ice for 10 minutes. Cells were then scraped from the surface of the well, and all contents transferred to a 1.5 mL Eppendorf tube. Lysates were pelleted (20,000 *g*, 10 min, 4 °C) and supernatant (45 μL) added to 15 μL NuPAGE™ LDS Sample Buffer (Invitrogen, #NP0007). Samples were heated (95 °C, 5 min) and loaded onto a Novex™ WedgeWell™ 4-20% Tris-Glycine 1.0 mm Mini Protein Gel (Invitrogen, #XP04205BOX) for electrophoresis. The gel was transferred to a nitrocellulose membrane using a Trans-Blot Turbo Transfer System (Bio-Rad), stained with Ponceau S to confirm protein transfer and imaged on a ChemiDoc XRS + (Bio-Rad). Once imaged, membranes were blocked with PBST (0.05% [v/v] Tween 20 in PBS) containing 5% (w/v) milk powder at 16 °C for 1 h, and then incubated (4 °C, overnight) with primary mouse anti-eGFP antibody (ThermoFisher, #MA1-952, 1:500 [v/v] dilution). The membrane was then washed three times (10 mL PBST, 5 min per wash). All subsequent washing steps used this procedure. Membranes were incubated in secondary anti-mouse antibody (ThermoFisher, #32430, 1:1000 [v/v] dilution) for 1 h at 16°C, and then washed. The signal was developed by addition of Clarity Max™ Western ECL Substrate (Bio-Rad, #1705062). After imaging on a ChemiDoc XRS + system, the membrane was washed 5 times (10 mL PBST, 5 min per wash) and then incubated (4 °C, overnight) with either mouse anti-GAPDH antibody (ThermoFisher, #MA5-15738, 1:500 [v/v] dilution) or mouse anti-FLAG (Sigma-Aldrich, #F3165, 1:500 [v/v] dilution) before being incubated with the secondary anti-mouse antibody and processed again for imaging as described above.

QUANTIFICATION AND STATISTICAL ANALYSIS

Flow cytometry data was analysed with FlowJo (version 10.7.2, BD Biosciences). Events were gated to ensure only mCherry-positive, single cells were used for quantification purposes. This was achieved by gating for single cells, excluding debris (FSC-A vs SSC-A) and gating to exclude doublets (FSC-A vs FSC-H and SSC-A vs SSC-H). Cells were gated for transfected cells by excluding mCherry negative cells (mCh-A vs FSC-A). Finally, mCherry fluorescence was plotted against eGFP fluorescence and the mean fluorescence intensities were taken to calculate the incorporation efficiency using the formula (Bartoschek et al., 2021):

$$Incoporation\ efficiency = \left(\frac{eGFP}{mCh}\Big/\frac{WT\ eGFP}{WT\ mCh}\right) - \left[\frac{eGFP}{mCh}\Big/\frac{WT\ eGFP}{WT\ mCh}\right]$$

Where ( ) and [ ] indicate samples incubated with and without the designated unnatural amino acid, respectively. Each condition was performed in triplicate (i.e. transient transfection of cells of different passages and split at different time). Data of each replicate containing over 10,000 mCherry positive cells were used to calculate the incorporation efficiency. Average incorporation efficiency was calculated from the three replicates for each condition and are provided in bar charts in Figures 2D, 3D, and 3E. The standard deviation from the means were also calculated and are represented as error bars on Figures 2D, 3D, and 3E and after ± symbols in Figures 3A and 3B. In the results, when directly comparing incorporation efficiencies between two vectors, an independent two-tailed t-test assuming equal variances was used. Incorporation efficiency, average incorporation efficiency, standard deviation from the mean and t-test calculations were performed in Microsoft Excel.

Cell Reports Methods, Volume *1*

Supplemental information

Development of mammalian cell logic gates controlled by unnatural amino acids

Emily M. Mills, Victoria L. Barlow, Arwyn T. Jones, and Yu-Hsuan Tsai

Table of Contents

1. Supplementary Figures 2
Figure S1. Production of eGFP(150XXXX) using different Pyl tRNA variants. Related to Figure 2. 2
Figure S2. Utilisation of TyrRS*/tRNA$_{CUA}$ as the second orthogonal pair. Related to Figure 3. 3
Figure S3. Incorporation of AzF and BocK into a single eGFP using Tyr tRNA$_{CUA}$ and Pyl tRNA$_{UCCU(Ev2)}$. Related to STAR Methods........ 4
Figure S4. Logic gate vector specificity test. Related to Figure 4. 5
2. Methods S1 6
2.1 Table S1. Primers used in this study. Related to STAR methods. 6
2.2 Working vector sequences. Related to STAR methods. 7
2.2.1 Vector 1: Pyl tRNACUA(U25C) 7
2.2.2 Vector 55: 4x(Tyr tRNA$_{CUA}$) TyrRS* mCherry-P2A-TAG-sGFP(1-10) 8
2.2.3 Vector 56: 4x(Pyl tRNA$_{UCCU(Ev2)}$) PylRS mCherry-P2A-AGGA-sGFP(11) 11
2.2.4 Vector 57: 4x(Tyr tRNA$_{CUA}$) TyrRS* sGFP(1-10)-TAG-sGFP(11) 13
2.2.5 Vector 58: Pyl tRNA$_{CUA(U25C)}$ PylRS mCherry-P2A-eGFP(150TAG) 15
2.2.6 Vector 59: Pyl tRNA$_{UCUA}$ PylRS mCherry-P2A-eGFP(150TAGA) 17
2.2.7 Vector 60: Pyl tRNA$_{UCUA(Ev1)}$ PylRS mCherry-P2A-eGFP(150TAGA) 19
2.2.8 Vector 61: Pyl tRNA$_{UCUA(Ev2)}$ PylRS mCherry-P2A-eGFP(150TAGA) 21
2.2.9 Vector 62: Pyl tRNA$_{CUAG}$ PylRS mCherry-P2A-eGFP(150CTAG) 23
2.2.10 Vector 63: Pyl tRNA$_{CUAG(Ev1)}$ PylRS mCherry-P2A-eGFP(150CTAG) 25
2.2.11 Vector 64: Pyl tRNA$_{UCCU}$ PylRS mCherry-P2A-eGFP(150AGGA) 27
2.2.12 Vector 65: Pyl tRNA$_{UCCU(Ev1)}$ PylRS mCherry-P2A-eGFP(150AGGA) 29
2.2.13 Vector 66: Pyl tRNA$_{UCCU(Ev2)}$ PylRS mCherry-P2A-eGFP(150AGGA) 31
2.2.14 Vector 67: Pyl tRNA$_{ACUA}$ PylRS mCherry-P2A-eGFP(150TAGT) 33
2.2.15 Vector 68: Pyl tRNA$_{CCUA}$ PylRS mCherry-P2A-eGFP(150TAGG) 35
2.2.16 Vector 69: Pyl tRNA$_{GCUA}$ PylRS mCherry-P2A-eGFP(150TAGC) 37
2.2.17 Vector 71: Tyr tRNA$_{CUA}$ TyrRS* mCherry-P2A-eGFP(150TAG) 39
2.2.18 Vector 73: 4x(Pyl tRNA$_{UCCU(Ev2)}$) PylRS mCherry-P2A-eGFP(150AGGA) 41
2.2.19 Vector 74: 4x(Tyr tRNA$_{CUA}$) TyrRS* mCherry-P2A-eGFP(150TAG) 43

1. Supplementary Figures

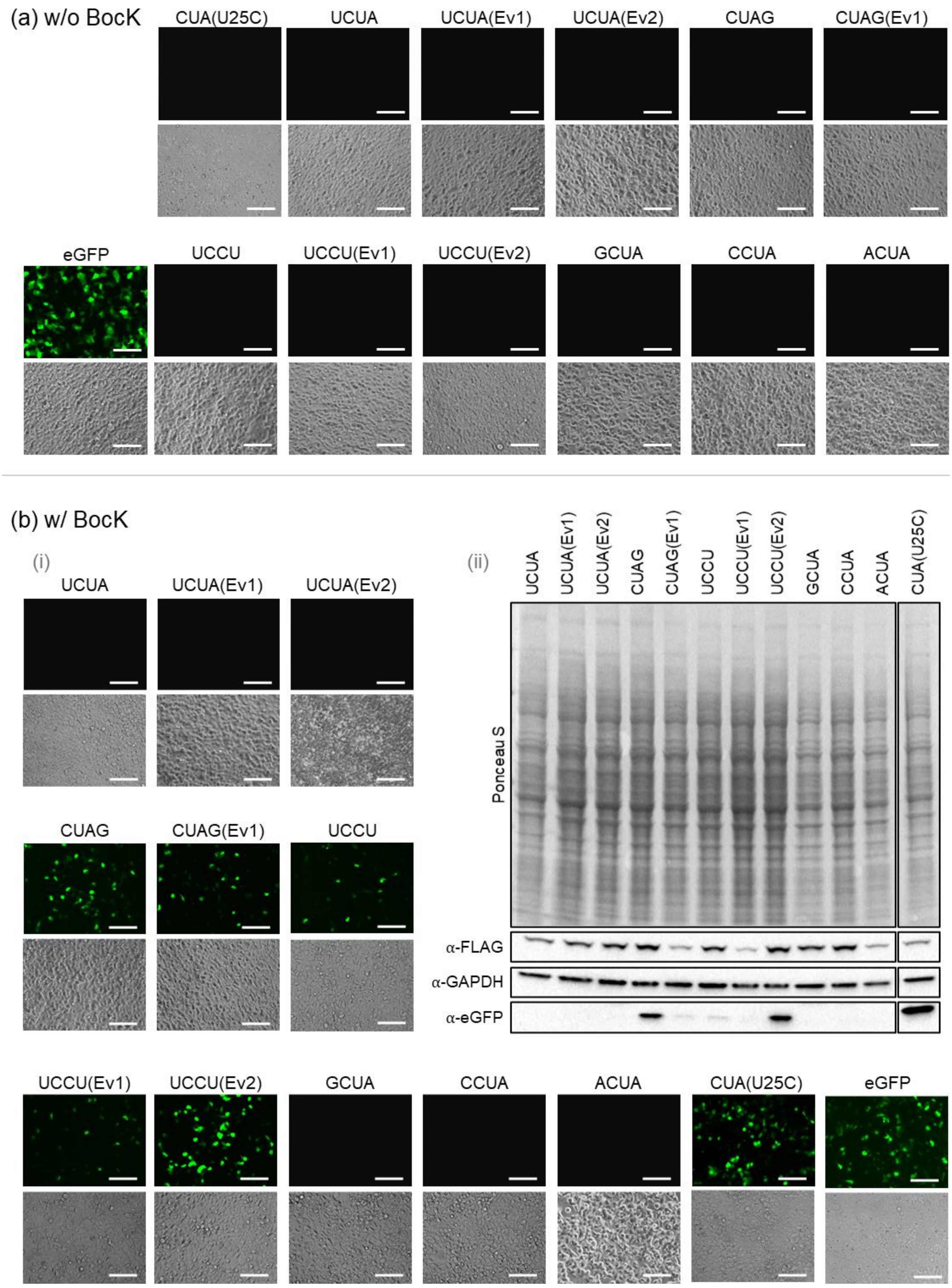

Figure S1. Production of eGFP(150XXXX) using different Pyl tRNA variants. Related to Figure 2. HEK293 cells were transiently transfected with the designated evaluation vector encoding the wild-type *M. mazei* PylRS, a Pyl tRNA variant and an eGFP reporter with the appropriate quadruplet codon at the position for the 150th amino acid residue in the absence (**a**) or presence (**b**) of 1 mM BocK for 24 h. No GFP fluorescence was detected for any of the variants when BocK was not supplemented. Transfection with a vector carrying the wild-type eGFP was used as the positive control. Bright field images show cell confluency in each condition. All images are 223 µm × 167 µm, scale bars denote 50 µm. Immunoblotting with α-FLAG and α-GFP antibodies were used to detect PylRS and full-length eGFP, respectively. An irrelevant lane was digitally deleted from the blot and is indicated by the gap between ACUA and CUA(U25C).

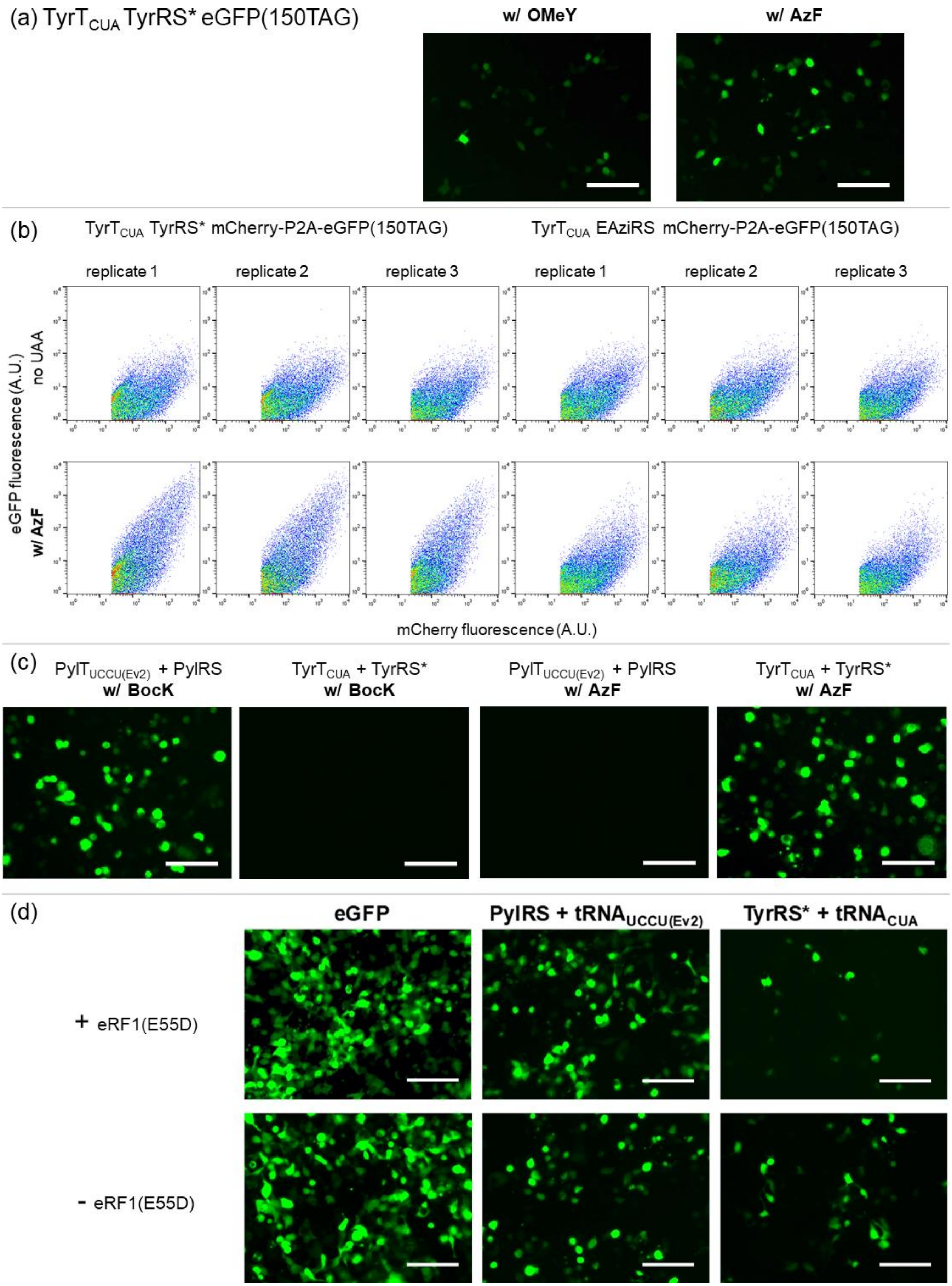

Figure S2. Utilisation of TyrRS*/tRNA$_{CUA}$ as the second orthogonal pair. Related to Figure 3. (**a**) Incorporation of OMeY and AzF into eGFP(150TAG) with TyrRS***.** Fluorescence microscopy images of OMeY and AzF incorporation into the 150th amino acid position of eGFP demonstrate higher levels of eGFP fluorescence when AzF is incorporated. (**b**) Flow cytometry analysis of incorporation of AzF into eGFP(150TAG) by TyrRS* or EAziRS. HEK293 cells transfected with TyrT$_{CUA}$ TyrRS* mCherry-P2A-eGFP(150TAG) or TyrT$_{CUA}$ EAziRS mCherry-P2A-eGFP(150TAG) were incubated in the absence or presence of AzF for 48 hours before flow cytometry analysis. Events were gated for HEK293 cells and to exclude doublets. Cells were then gated to include only transfected (i.e. mCherry positive) cells. Fluorescence intensities are given in arbitrary units (A.U.). Three biological replicates for each condition are shown. The mean fluorescence intensities were used to calculate the incorporation efficiency as per the equation in Figure 2. The incorporation efficiency is 8% ± 2% for TyrT$_{CUA}$ TyrRS* mCherry-P2A-eGFP(150TAG), and 0.6% ± 0.3% for TyrT$_{CUA}$ EAziRS mCherry-P2A-eGFP(150TAG). (**c**) Orthogonality of tRNA/aaRA pairs. HEK293 cells transfected with PylRS/tRNA$_{UCCU(Ev2)}$ or TyrRS*/tRNA$_{CUA}$ reporter vector and incubated for 24 hours in the presence of AzF or BocK, respectively. Fluorescent eGFP was only

detected when the aaRS/tRNA pairs were in the presence of their cognate unnatural amino acid depicting mutual orthogonality of the pairs. (**d**) Impact of eRF1(E55D) on incorporation efficiency of TyrRS*/tRNA$_{CUA}$ or PylRS/tRNA$_{UCCU(Ev2)}$. No substantial increase in eGFP fluorescence was observed. eRF1(E55D) was also expressed alongside wildtype eGFP as a control. All fluorescent images are 223 µm × 167 µm, scale bars denote 50 µm.

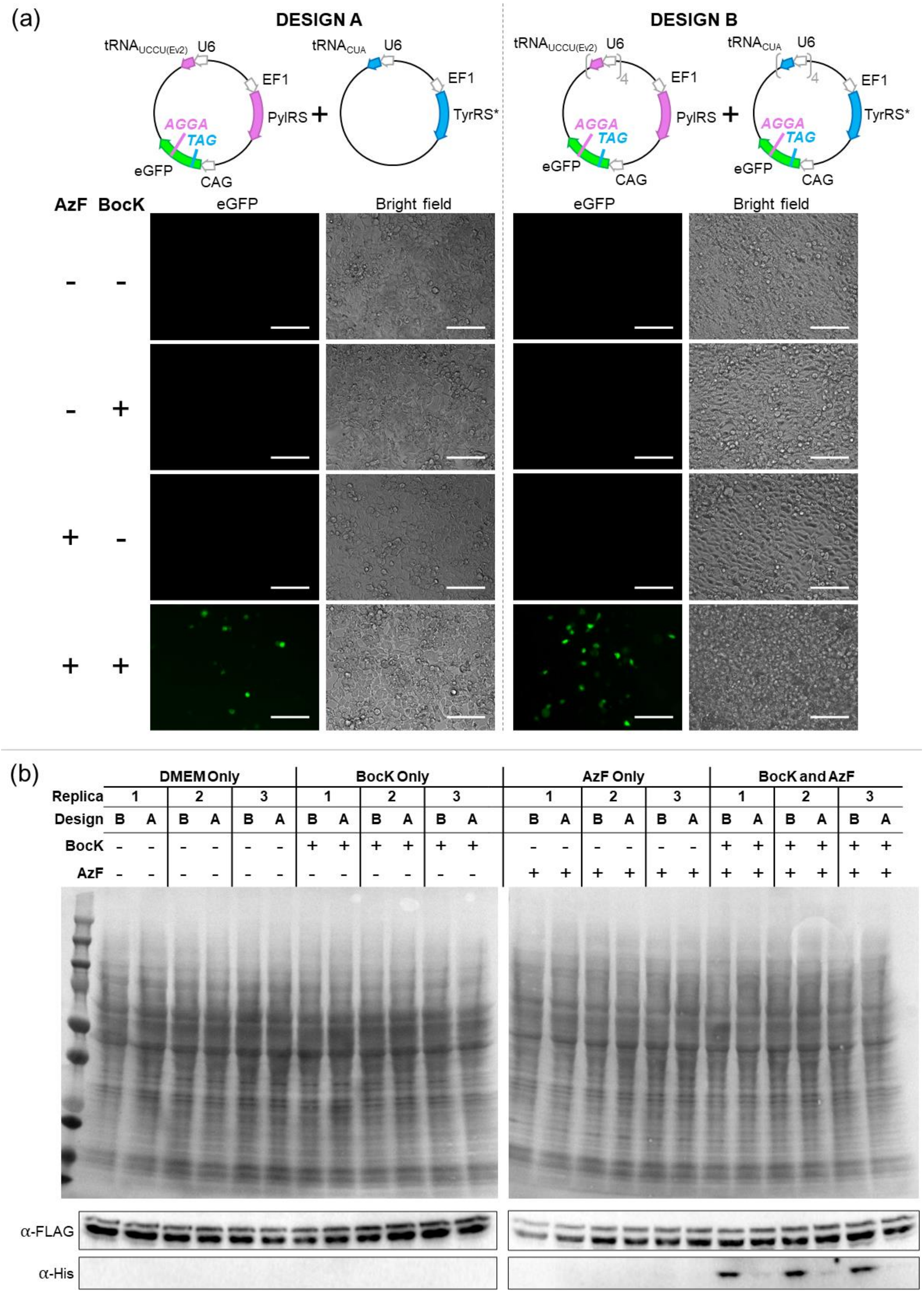

Figure S3. Incorporation of AzF and BocK into a single eGFP using Tyr tRNA$_{CUA}$ and Pyl tRNA$_{UCCU(Ev2)}$. Related to STAR Methods. (**a**) AzF was incorporated at the 40th residue of the mutant eGFP using amber suppression and BocK was incorporated into the 150th residue of the eGFP mutant using AGGA suppression. Double incorporation was investigated with two experimental designs: Design A comprised of a single reporter gene and tRNA for respective pairs, Design B an attempt at optimisation with increased tRNA and reporter copy number. eGFP fluorescence was only detected when cells were supplemented with both AzF and

BocK, Design B demonstrated higher levels of eGFP fluorescence. (**b**) Western blot of Design A vs Design B with three biological replicates of 4 conditions: (1) no unnatural amino acid, (2) BocK only, (3) AzF only and (4) BocK and AzF. Full length eGFP was only detected when both unnatural amino acids are supplemented, with Design B yielding substantially higher eGFP band intensity. All fluorescent images are 223 µm × 167 µm, scale bars denote 50 µm.

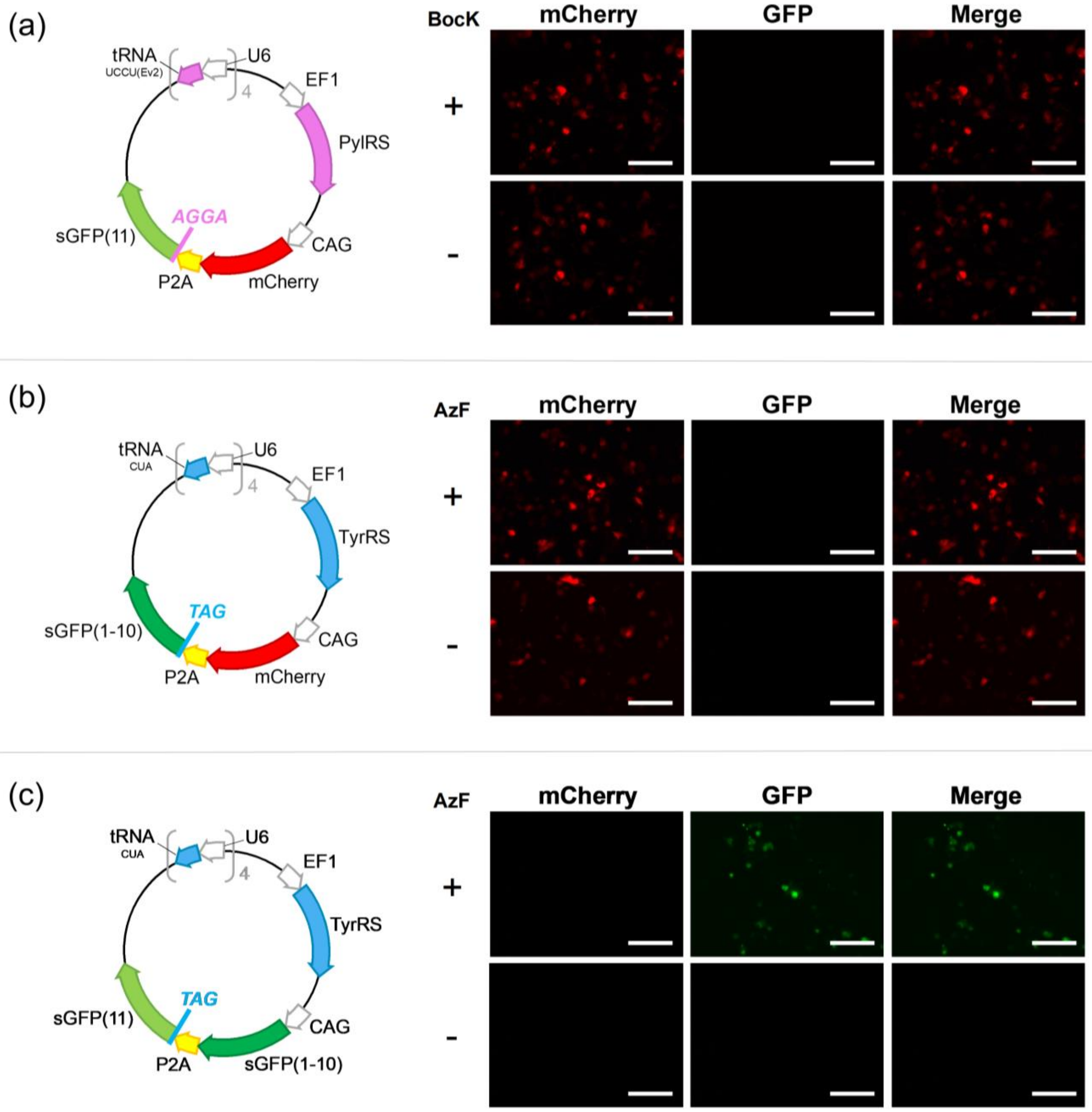

Figure S4. Logic gate vector specificity test. Related to Figure 4. (**a**-**c**) Individual transfection of logic gate vectors in the presence (+) or absence (-) of their respective unnatural amino acid (BocK or AzF) at 1 mM. All images depicted are 223 µm × 167 µm, scale bars denote 50 µm.

2. Methods S1

2.1 Table S1. Primers used in this study. Related to STAR methods.

Primer ID	Primer sequence (5'-3')
**Primer 1**	GGGGGGATACGGGGAAAAGGCCTAAAAACCGCACTTGTCCGGAAAC
**Primer 2**	GGTCAGGGCTGGGGCAGAGGCTTGCACAGGGGCAGACATG
**Primer 3**	ATCATGGCTGATAAGCAGAAGAAC
**Primer 4**	CGTTCTTCTGCTTATCAGCCATGATATAGACTCTAGTGGCTGTTGTAGTTGTACTCCAGCTTG
**Primer 5**	CGTTCTTCTGCTTATCAGCCATGATATAGACCTAGGTGGCTGTTGTAGTTGTACTCCAGCTTG
**Primer 6**	CGTTCTTCTGCTTATCAGCCATGATATAGACTCCTGTGGCTGTTGTAGTTGTACTCCAGCTTG
**Primer 7**	CGTTCTTCTGCTTATCAGCCATGATATAGACACTAGTGGCTGTTGTAGTTGTACTCCAGCTTG
**Primer 8**	CGTTCTTCTGCTTATCAGCCATGATATAGACGCTAGTGGCTGTTGTAGTTGTACTCCAGCTTG
**Primer 9**	CGTTCTTCTGCTTATCAGCCATGATATAGACCCTAGTGGCTGTTGTAGTTGTACTCCAGCTTG
**Primer 10**	TTCGATCTACATGATCAGGTTTCCGGTG
**Primer 11**	ACCTGATCATGTAGATCGAATGGACTTCTAAATCCGTTCAGCCGGGTTAGATTCC
**Primer 12**	ACCTGATCATGTAGATCGAATGGGCTTCTAATCTCGTTCAGCCGGGTTAGATTCC
**Primer 13**	ACCTGATCATGTAGATCGAATGGGCTTCTAATCCGCTTCAGCCGGGTTAGATTCC
**Primer 14**	ACCTGATCATGTAGATCGAATGGACTCTAGAATCCGTTCAGCCGGGTTAGATTCC
**Primer 15**	ACCTGATCATGTAGATCGAATGGCCTCTAGAACCCGTTCAGCCGGGTTAGATTCC
**Primer 16**	ACCTGATCATGTAGATCGAATGGACTTCCTAATCCGTTCAGCCGGGTTAGATTCC
**Primer 17**	ACCTGATCATGTAGATCGAATCCTCTTCCTAATAGGTTCAGCCGGGTTAGATTCC
**Primer 18**	ACCTGATCATGTAGATCGAAGGGGCTTCCTATCCGCTTCAGCCGGGTTAGATTCC
**Primer 19**	ACCTGATCATGTAGATCGAATGGACTACTAAATCCGTTCAGCCGGGTTAGATTCC
**Primer 20**	ACCTGATCATGTAGATCGAATGGACTGCTAAATCCGTTCAGCCGGGTTAGATTCC
**Primer 21**	ACCTGATCATGTAGATCGAATGGACTCCTAAATCCGTTCAGCCGGGTTAGATTCC
**Primer 22**	GAAAAGGAGGCCTACATGCAAATATTAAAAAATGGTGGGGGAAGGATTCGAACCTTC
**Primer 23**	ACCGGAGCGATCGCAACCGGTCGGGCAGGAAGAGGGCCTATTTCCCAT
**Primer 24**	ACTTACGCTTGCCACCATGGCTAGCGACTACAAGGACGACGACGACAAGGCAAGCAGTAACTTGATTAAACAATTGCAAGAG
**Primer 25**	TCCACCACACTGGACTAGTGGATCCTTATCATTAAACGGGCCCTTTCCAGCAAATC
**Primer 26**	GGGGGGATACGGGGAAAAGGCCTCTTAAGAAAAACCGCACTTGTC
**Primer 27**	GTAAACGGCCACAAGTTCGTCGATTGGGCAGGAAGAGGGCCTATTTC
**Primer 28**	TCGACGAACTTGTGGCCGTTTACCCTCTTAAGAAAAACCGCACTTGTC
**Primer 29**	CTGGTGGAGAACTTGCCGAATTGGGCAGGAAGAGGGCCTATTTC
**Primer 30**	TTCGGCAAGTTCTCCACCAGCCTCTTAAGAAAAACCGCACTTGTC
**Primer 31**	AGTGCGGTTTTTCTTAAGAGGTTGGGCAGGAAGAGGGCCTATTTC
**Primer 32**	GGATACGGGGAAAAGGAGGCCTACATGC
**Primer 33**	GTAAACGGCCACAAGTTCGTCGATTGGGCAGGAAGAGGGCCTATTTC
**Primer 34**	TCGACGAACTTGTGGCCGTTTACCATGCAAATATTAAAAAATGGTGGGGGAAG
**Primer 35**	CTGGTGGAGAACTTGCCGAATTGGGCAGGAAGAGGGCCTATTTC

**Primer 36**	AATTCGGCAAGTTCTCCACCAGACATGCAAATATTAAAAAATGGTGGGGGAAG
**Primer 37**	TTTTAATATTTGCATGTAGGGGGCAGGAAGAGGGCCTATTTC
**Primer 38**	AGGGTCAGCTTGCCCTAGGTGGCATCG
**Primer 39**	TCGGCATGGACGAGCTGTACAAGCATCATCACCATCACCACTAAGCTGCCTATCAGAAG
**Primer 40**	ATGCCACCTAGGGCAAGCTGACCCTGAAGTTC
**Primer 41**	TTGTACAGCTCGTCCATGCCGAGAG
**Primer 42**	AGAACCCTGGACCTGGACAAAAG TCGTAGTCCAAAGGAGAAGAACTGTTTACCGGTGTTG
**Primer 43**	TCCCCATAATTTTTGGCAGAGGGAAAAAGATCTC
**Primer 44**	GAGAACCCTGGACCTGGACAAAAGTCGAGGACGTGAC CACATGGTCCTTCATG
**Primer 45**	TTTTTGGCAGAGGGAAA AAGATCTCACATG
**Primer 46**	TTCAGCCTGCTGAAACAGGCTGGCGACGTGGAAGAGAACCCTGGACCTGGACAAAAGTCGTAGCGTGACCACATGGTCCTTCATG
**Primer 47**	TTTTTGGCAGAGGGAAAAAGATCTCACATG
**Primer 48**	TGTCTCATCATTTTGGCAAAGAATTCATGTCCAAAGGAGAAGAACTGTTTACC
**Primer 49**	TTCTCTTCCACGTCGCCAGCCTGTTTCAGCAGGCTGAAATTGGTGGCGCCTCCAGACGCTTTTTCATTTGGATCTTTGCTCAGGACTGTTTG
**Primer 50**	AGCAAGGGCGAGGAGCTG
**Primer 51**	TGTCCCCATAATTTTTGGCAGAGGGAAAAAGATCTTTACTTGTACAGCTCGTCCATGCCGAGAG
**Primer 52**	GTCTCATCATTTTGGCAAAGAATTCGCCACCATGGTGAGCAAGGGCGAG
**Primer 53**	AACAGCTCCTCGCCCTTGCTCACCGACTTTTGTCCAGGTCCAGG
**Primer 54**	TGCCGCCAGAACACAGCTGAAGC
**Primer 55**	GAGTGCGATCGGGCCTTGC
**Primer 56**	CGCAAGGCCCGATCGCACTCCTGTGTGGCTTCGATCCTACCGCTGAC
**Primer 57**	ACTATAACCCTGCAGCAGGTTG
**Primer 58**	ACCTGCTGCAGGGTTATAGTGCCGCCTGTGCCAACAAACAGTACGGTGTGGTGC
**Primer 59**	TCCACCACACTGGACTAGTGGATCCTTATCATTAAACGGGCCCTTTCCAGC

2.2 Working vector sequences. Related to STAR methods.

Vector 1 has kanamycin resistance; all other vectors have ampicillin resistance. Gene sequences are colour-coded using the following: U6 promotor, **PylT** (anticodon highlighted), EF1 promotor, **PylRS**, CAG promotor and chimeric intron, **eGFP** (150th codon highlighted), **TyrT** (anticodon highlighted), **TyrRS**, **mCherry**, **linker**, **P2A**, **sGFP(1-10)**, sGFP(11)7x (AGGA/TAG codon highlighted)

2.2.1 Vector 1: Pyl tRNACUA(U25C)

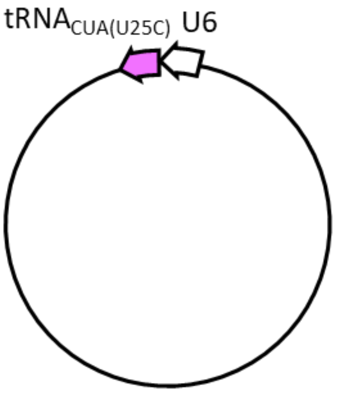

CTAAATTGTAAGCGTTAATATTTTGTTAAAATTCGCGTTAAATTTTTGTTAAATCAGCTCATTTTTTAACCAATAGGCCGAAATCGGC
AAAATCCCTTATAAATCAAAAGAATAGACCGAGATAGGGTTGAGTGGCCGCTACAGGGCGCTCCCATTCGCCATTCAGGCTGCG
CAACTGTTGGGAAGGGCGTTTCGGTGCGGGCCTCTTCGCTATTACGCCAGCTGGCGAAAGGGGGATGTGCTGCAAGGCGATTA
AGTTGGGTAACGCCAGGGTTTTCCCAGTCACGACGTTGTAAAACGACGGCCAGTGAGCGCGACGTAATACGACTCACTATAGGG
CGAATTGAAGGAAGGCCGTCAAGGCCGCATAGGCCTCTTAAGAAAAACCGCACTTGTC**CGGAAACCCCGGGAATCTAACCCGG**
CTGAACGGATTTAGAGTCCGTTCGATCTACATGATCAGGTTTCCGGTGTTTCGTCCTTTCCACAAGATATATAAAGCCAAGAAAT
CGAAATACTTTCAAGTTACGGTAAGCATATGATAGTCCATTTTAAAACATAATTTTAAAACTGCAAACTACCCAAGAAATTATTACTT
TCTACGTCACGTATTTTGTACTAATATCTTTGTGTTTACAGTCAAATTAATTCTAATTATCTCTCTAACAGCCTTGTATCGTATATGC
AAATATGAAGGAATCATGGGAAATAGGCCCTCTTCCTGCCCAACCGGTTGCGATCGCTCCGGTGCCCGTCAGTGGGCAGAGCG
CACATCGCCCACAGTCCCCGAGAAGTTGGGGGGAGGGGTCGGCAATTGAACGGGTGCCTAGAGAAGGTGGCGCGGGGTAAAC
TGGGAAAGTGATGTCGTGTACTGGCTCCGCCTTTTTCCCGAGGGTGGGGGAGAACCGTATATAAGTGCAGTAGTCGCCGTGAAC
GTTCTTTTTCGCAACGGGTTTGCCGCCAGAACACAGCTGAAGCTTCGAGGGGCTCGCATCTCTCCTTCACGCGCCCGCCGCCCT
ACCTGAGGCCGCCATCCACGCCGGTTGAGTCGCGTTCTGCCGCCTCCCGCCTGTGGTGCCTCCTGAACTGCGTCCGCCGTCTA
GGTAAGTTTAAAGCTCAGGTCGAGACCGGGCCTTTGTCCGGCGCTCCCTTGGAGCCTACCTAGACTCAGCCGGCTCTCCACGC
TTTGCCTGACCCTGCTTGCTCAACTCTACGTCTTTGTTTCGTTTTCTGTTCTGCGCCGTTACAGATCCAAGCTGTGACCGGCGCC
TACTCTACAGATAGCGTTTAAACTTACGCTTGCCACCATGGCTAGCGCGGCCGCAGGTACCGGAAGCGGAGCTACTAACTTCAG
CCTGCTGAAGCAGGCTGGAGACGTGGAGGAGAACCCTGGACCTGACAAGAAGCCCCTGAACACCCTGATCAGCGCCACAGGA
CTGTGGATGTCCAGAACCGGCACCATCCACAAGATCAAGCACCACGAGGTGTCCCGGTCCAAAATCTACATCGAGATGGCCTGC
GGCGATCACCTGGTCGTCAACAACAGCAGAAGCAGCCGGACAGCCAGAGCCCTGCGGCACCACAAGTACAGAAAGACCTGCAA
GCGGTGCAGAGTGTCCGACGAGGACCTGAACAAGTTCCTGACCAAGGCCAACGAGGACCAGACCAGCGTGAAAGTGAAGGTG
GTGTCCGCCCCCACCCGGACCAAGAAAGCCATGCCCAAGAGCGTGGCCAGAGCCCCCAAGCCCCTGGAAAACACCGAAGCCG
CTCAGGCCCAGCCCAGCGGCAGCAAGTTCAGCCCCGCCATCCCCGTGTCTACCCAGGAAAGCGTCAGCGTCCCCGCCAGCGT
GTCCACCAGCATCTCTAGCATCTCAACCGGCGCCACAGCTTCTGCCCTGGTCAAGGGCAACACCAACCCCATCACCAGCATGTC
TGCCCCTGTGCAGGCCTCTGGGCCTCATGGGCCTTCCTTTCACTGCCCGCTTTCCAGTCGGGAAACCTGTCGTGCCAGCTGCAT
TAACATGGTCATAGCTGTTTCCTTGCGTATTGGGCGCTCTCCGCTTCCTCGCTCACTGACTCGCTGCGCTCGGTCGTTCGGGTA
AAGCCTGGGGTGCCTAATGAGCAAAAGGCCAGCAAAAGGCCAGGAACCGTAAAAAGGCCGCGTTGCTGGCGTTTTTCCATAGG
CTCCGCCCCCCTGACGAGCATCACAAAAATCGACGCTCAAGTCAGAGGTGGCGAAACCCGACAGGACTATAAAGATACCAGGC
GTTTCCCCCTGGAAGCTCCCTCGTGCGCTCTCCTGTTCCGACCCTGCCGCTTACCGGATACCTGTCCGCCTTTCTCCCTTCGGG
AAGCGTGGCGCTTTCTCATAGCTCACGCTGTAGGTATCTCAGTTCGGTGTAGGTCGTTCGCTCCAAGCTGGGCTGTGTGCACGA
ACCCCCCGTTCAGCCCGACCGCTGCGCCTTATCCGGTAACTATCGTCTTGAGTCCAACCCGGTAAGACACGACTTATCGCCACT
GGCAGCAGCCACTGGTAACAGGATTAGCAGAGCGAGGTATGTAGGCGGTGCTACAGAGTTCTTGAAGTGGTGGCCTAACTACG
GCTACACTAGAAGAACAGTATTTGGTATCTGCGCTCTGCTGAAGCCAGTTACCTTCGGAAAAAGAGTTGGTAGCTCTTGATCCGG
CAAACAAACCACCGCTGGTAGCGGTGGTTTTTTTGTTTGCAAGCAGCAGATTACGCGCAGAAAAAAAGGATCTCAAGAAGATCCT
TTGATCTTTTCTACGGGGTCTGACGCTCAGTGGAACGAAAACTCACGTTAAGGGATTTTGGTCATGAGATTATCAAAAAGGATCTT
CACCTAGATCCTTTTAAATTAAAAATGAAGTTTTAAATCAATCTAAAGTATATATGAGTAAACTTGGTCTGACAGTTATTAGAAAAAT
TCATCCAGCAGACGATAAAACGCAATACGCTGGCTATCCGGTGCCGCAATGCCATACAGCACCAGAAAACGATCCGCCCATTCG
CCGCCCAGTTCTTCCGCAATATCACGGGTGGCCAGCGCAATATCCTGATAACGATCCGCCACGCCCAGACGGCCGCAATCAATA
AAGCCGCTAAAACGGCCATTTTCCACCATAATGTTCGGCAGGCACGCATCACCATGGGTCACCACCAGATCTTCGCCATCCGGC
ATGCTCGCTTTCAGACGCGCAAACAGCTCTGCCGGTGCCAGGCCCTGATGTTCTTCATCCAGATCATCCTGATCCACCAGGCCC
GCTTCCATACGGGTACGCGCACGTTCAATACGATGTTTCGCCTGATGATCAAACGGACAGGTCGCCGGGTCCAGGGTATGCAGA
CGACGCATGGCATCCGCCATAATGCTCACTTTTTCTGCCGGCGCCAGATGGCTAGACAGCAGATCCTGACCCGGCACTTCGCCC
AGCAGCAGCCAATCACGGCCCGCTTCGGTCACCACATCCAGCACCGCCGCACACGGAACACCGGTGGTGGCCAGCCAGCTCA
GACGCGCCGCTTCATCCTGCAGCTCGTTCAGCGCACCGCTCAGATCGGTTTTCACAAACAGCACCGGACGACCCTGCGCGCTC
AGACGAAACACCGCCGCATCAGAGCAGCCAATGGTCTGCTGCGCCCAATCATAGCCAAACAGACGTTCCACCCACGCTGCCGG
GCTACCCGCATGCAGGCCATCCTGTTCAATCATACTCTTCCTTTTTCAATATTATTGAAGCATTTATCAGGGTTATTGTCTCATGAG
CGGATACATATTTGAATGTATTTAGAAAAATAAACAAATAGGGGTTCCGCGCACATTTCCCCGAAAAGTGCCAC

2.2.2 Vector 55: 4x(Tyr $tRNA_{CUA}$) TyrRS* mCherry-P2A-TAG-sGFP(1-10)

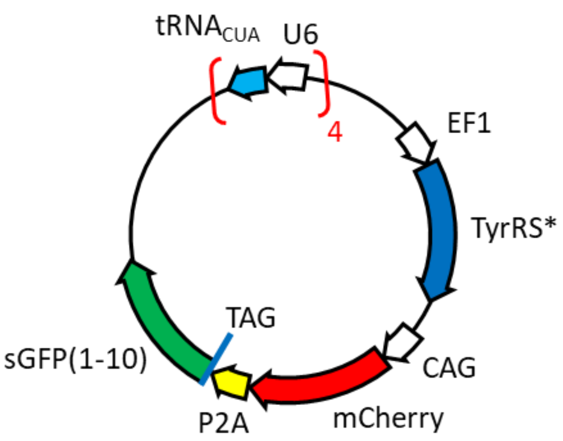

ACTCTTCCTTTTTCAATATTATTGAAGCATTTATCAGGGTTATTGTCTCATGAGCGGATACATATTTGAATGTATTTAGAAAAATAAA
CAAATAGGGGTTCCGCGCACATTTCCCCGAAAAGTGCCACCTAAATTGTAAGCGTTAATATTTTGTTAAAATTCGCGTTAAATTTTT
GTTAAATCAGCTCATTTTTTAACCAATAGGCCGAAATCGGCAAAATCCCTTATAAATCAAAAGAATAGACCGAGATAGGGTTGAGT
GTTGTTCCAGTTTGGAACAAGAGTCCACTATTAAAGAACGTGGACTCCAACGTCAAAGGGCGAAAAACCGTCTATCAGGGCGAT
GGCCCACTACGTGAACCATCACCCTAATCAAGTTTTTTGGGGTCGAGGTGCCGTAAAGCACTAAATCGGAACCCTAAAGGGAGC
CCCCGATTTAGAGCTTGACGGGGAAAGCCGGCGAACGTGGCGAGAAAGGAAGGGAAGAAAGCGAAAGGAGCGGGCGCTAGGG
CGCTGGCAAGTGTAGCGGTCACGCTGCGCGTAACCACCACACCCGCCGCGCTTAATGCGCCGCTACAGGGCGCGTCCCATTC

GCCATTCAGGCTGCGCAACTGTTGGGAAGGGCGATCGGTGCGGGCCTCTTCGCTATTACGCCAGCTGGCGAAAGGGGGATGT
GCTGCAAGGCGATTAAGTTGGGTAACGCCAGGGTTTTCCCAGTCACGACGTTGTAAAACGACGGCCAGTGAGCGCGCCTCGTT
CATTCACGTTTTTGAACCCGTGGAGGACGGGCAGACTCGCGGTGCAAATGTGTTTTACAGCGTGATGGAGCAGATGAAGATGCT
CGACACGCTGCAGAACACGCAGCTAGATTAACCCTAGAAAGATAATCATATTGTGACGTACGTTAAAGATAATCATGCGTAAAATT
GACGCATGTGTTTTATCGGTCTGTATATCGAGGTTTATTTATTAATTTGAATAGATATTAAGTTTTATTATATTTACACTTACATACTA
ATAATAAATTCAACAAACAATTTATTTATGTTTATTTATTTATTAAAAAAAACAAAAACTCAAAATTTCTTCTATAAAGTAACAAAACTT
TTATGAGGGACAGCCCCCCCCCAAAGCCCCCAGGGATGTAATTACGTCCCTCCCCCGCTAGGGGGCAGCAGCGAGCCGCCCG
GGGCTCCGCTCCGGTCCGGCGCTCCCCCCGCATCCCCGAGCCGGCAGCGTGCGGGGACAGCCCGGGCACGGGGAAGGTGG
CACGGGATCGCTTTCCTCTGAACGCTTCTCGCTGCTCTTTGAGCCTGCAGACACCTGGGGGGATACGGGGAAAAGGCCTCTTAA
GAAAAACCGCACTTGTC**TGGTGGGGGAAGGATTCGAACCTTCGAAGTCTGTGACGGCAGATTTAGAGTCTGCTCCCTTTGGCC**
GCTCGGGAACCCCACCGGTGTTTCGTCCTTTCCACAAGATATATAAAGCCAAGAAATCGAAATACTTTCAAGTTACGGTAAGCAT
ATGATAGTCCATTTTAAAACATAATTTTAAAACTGCAAACTACCCAAGAAATTATTACTTTCTACGTCACGTATTTTGTACTAATATC
TTTGTGTTTACAGTCAAATTAATTCTAATTATCTCTCTAACAGCCTTGTATCGTATATGCAAATATGAAGGAATCATGGGAAATAGG
CCCTCTTCCTGCCCAATCGACGAACTTGTGGCCGTTTACCCTCTTAAGAAAAACCGCACTTGTC**TGGTGGGGGAAGGATTCGAA**
CCTTCGAAGTCTGTGACGGCAGATTTAGAGTCTGCTCCCTTTGGCCGCTCGGGAACCCCACCGGTGTTTCGTCCTTTCCACAA
GATATATAAAGCCAAGAAATCGAAATACTTTCAAGTTACGGTAAGCATATGATAGTCCATTTTAAAACATAATTTTAAAACTGCAAA
CTACCCAAGAAATTATTACTTTCTACGTCACGTATTTTGTACTAATATCTTTGTGTTTACAGTCAAATTAATTCTAATTATCTCTCTAA
CAGCCTTGTATCGTATATGCAAATATGAAGGAATCATGGGAAATAGGCCCTCTTCCTGCCCAATTCGGCAAGTTCTCCACCAGCC
TCTTAAGAAAAACCGCACTTGTC**TGGTGGGGGAAGGATTCGAACCTTCGAAGTCTGTGACGGCAGATTTAGAGTCTGCTCCCTT**
TGGCCGCTCGGGAACCCCACCGGTGTTTCGTCCTTTCCACAAGATATATAAAGCCAAGAAATCGAAATACTTTCAAGTTACGGTA
AGCATATGATAGTCCATTTTAAAACATAATTTTAAAACTGCAAACTACCCAAGAAATTATTACTTTCTACGTCACGTATTTTGTACTA
ATATCTTTGTGTTTACAGTCAAATTAATTCTAATTATCTCTCTAACAGCCTTGTATCGTATATGCAAATATGAAGGAATCATGGGAA
ATAGGCCCTCTTCCTGCCCAACCTCTTAAGAAAAACCGCACTTGTCCCTAAAAACCGCACTTGTC**TGGTGGGGGAAGGATTCGA**
ACCTTCGAAGTCTGTGACGGCAGATTTAGAGTCTGCTCCCTTTGGCCGCTCGGGAACCCCACCGGTGTTTCGTCCTTTCCACA
AGATATATAAAGCCAAGAAATCGAAATACTTTCAAGTTACGGTAAGCATATGATAGTCCATTTTAAAACATAATTTTAAAACTGCAA
ACTACCCAAGAAATTATTACTTTCTACGTCACGTATTTTGTACTAATATCTTTGTGTTTACAGTCAAATTAATTCTAATTATCTCTCTA
ACAGCCTTGTATCGTATATGCAAATATGAAGGAATCATGGGAAATAGGCCCTCTTCCTGCCCAACCGGTTGCGATCGCTCCGGT
GCCCGTCAGTGGGCAGAGCGCACATCGCCCACAGTCCCCGAGAAGTTGGGGGGAGGGGTCGGCAATTGAACGGGTGCCTAGA
GAAGGTGGCGCGGGGTAAACTGGGAAAGTGATGTCGTGTACTGGCTCCGCCTTTTTCCCGAGGGTGGGGGAGAACCGTATATA
AGTGCAGTAGTCGCCGTGAACGTTCTTTTTCGCAACGGGTTTGCCGCCAGAACACAGCTGAAGCTTCGAGGGGCTCGCATCTCT
CCTTCACGCGCCCGCCGCCCTACCTGAGGCCGCCATCCACGCCGGTTGAGTCGCGTTCTGCCGCCTCCCGCCTGTGGTGCCT
CCTGAACTGCGTCCGCCGTCTAGGTAAGTTTAAAGCTCAGGTCGAGACCGGGCCTTTGTCCGGCGCTCCCTTGGAGCCTACCTA
GACTCAGCCGGCTCTCCACGCTTTGCCTGACCCTGCTTGCTCAACTCTACGTCTTTGTTTCGTTTTCTGTTCTGCGCCGTTACAG
ATCCAAGCTGTGACCGGCGCCTACTCTACAGATAGCGTTTAAACTTACGCTTGCCACC**ATGGCTAGCGACTACAAGGACGACG**
ACGACAAGGCAAGCAGTAACTTGATTAAACAATTGCAAGAGCGGGGGCTGGTAGCCCAGGTGACGGACGAGGAAGCGTTAG
CAGAGCGACTGGCGCAAGGCCCGATCGCACTCGTGTGGCTTCGATCCTACCGCTGACAGCTTGCATTTGGGGCATCTTGTT
CCATTGTTATGCCTGAAACGCTTCCAGCAGGCGGGCCACAAGCCGGTTGCGCTGGTAGGCGGCGCGACGGGTCTGATTGGC
GACCCGAGCTTCAAAGCTGCCGAGCGTAAGCTGAACACCGAAGAAACTGTTCAGGAGTGGGTGGACAAAATCCGTAAGCAG
GTTGCCCCGTTCCTCGATTTCGACTGTGGAGAAAACTCTGCTATCGCGGCCAATAATTATGACTGGTTCGGCAATATGAATGT
GCTGACCTTCCTGCGCGATATTGGCAAACACTTCTCCGTTAACCAGATGATCAACAAAGAAGCGGTTAAGCAGCGTCTCAACC
GTGAAGATCAGGGGATTTCGTTCACTGAGTTTTCCTACAACCTGCTGCAGGGTTATAGTATGGCCTGTTTGAACAAACAGTAC
GGTGTGGTGCTGCAAATTGGTGGTTCTGACCAGTGGGGTAACATCACTTCTGGTATCGACCTGACCCGTCGTCTGCATCAGAA
TCAGGTGTTTGGCCTGACCGTTCCGCTGATCACTAAAGCAGATGGCACCAAATTTGGTAAAACTGAAGGCGGCGCAGTCTGG
TTGGATCCGAAGAAAACCAGCCCGTACAAATTCTACCAGTTCTGGATCAACACTGCGCGTGCCGACGTTTACCGCTTCCTGAA
GTTCTTCACCTTTATGAGCATTGAAGAGATCAACGCCCTGGAAGAAGAAGATAAAAACAGCGGTAAAGCACCGCGCGCCCAG
TATGTACTGGCGGAGCAGGTGACTCGTCTGGTTCACGGTGAAGAAGGTTTACAGGCGGCAAAACGTATTACCGAATGCCTGT
TCAGCGGTTCTTTGAGTGCGCTGAGTGAAGCGGACTTCGAACAGCTGGCGCAGGACGGCGTACCGATGGTTGAGATGGAAAA
GGGCGCAGACCTGATGCAGGCACTGGTCGATTCTGAACTGCAACCTTCCCGTGGTCAGGCACGTAAAACTATCGCCTCCAAT
GCCATCACCATTAACGGTGAAAAACAGTCCGATCCTGAATACTTCTTTAAAGAAGAAGATCGTCTGTTTGGTCGTTTTACCTTA
CTGCGTCGCGGTAAAAAGAATTACTGTCTGATTTGCTGGAAAGGGCCCGTTTAATGATAAGGATCCACTAGTCCAGTGTGGTG
GAATTGACATTGATTATTGACTAGTTATTAATAGTAATCAATTACGGGGTCATTAGTTCATAGCCCATATATGGAGTTCCGCGTTAC
ATAACTTACGGTAAATGGCCCGCCTGGCTGACCGCCCAACGACCCCCGCCCATTGACGTCAATAATGACGTATGTTCCCATAGT
AACGCCAATAGGGACTTTCCATTGACGTCAATGGGTGGAGTATTTACGGTAAACTGCCCACTTGGCAGTACATCAAGTGTATCAT
ATGCCAAGTACGCCCCCTATTGACGTCAATGACGGTAAATGGCCCGCCTGGCATTATGCCCAGTACATGACCTTATGGGACTTTC
CTACTTGGCAGTACATCTACGTATTAGTCATCGCTATTACCATGGTCGAGGTGAGCCCCACGTTCTGCTTCACTCTCCCCATCTC
CCCCCCCTCCCCACCCCCAATTTTGTATTTATTTATTTTTTAATTATTTTGTGCAGCGATGGGGGCGGGGGGGGGGGGGGCGCG
CGCCAGGCGGGGCGGGGCGGGGCGAGGGGCGGGGCGGGGCGAGGCGGAGAGGTGCGGCGGCAGCCAATCAGAGCGGCGC
GCTCCGAAAGTTTCCTTTTATGGCGAGGCGGCGGCGGCGGCGGCCCTATAAAAAGCGAAGCGCGCGGCGGGCGGGAGTCGCT
GCGTTGCCTTCGCCCCGTGCCCCGCTCCGCGCCGCCTCGCGCCGCCCGCCCCGGCTCTGACTGACCGCGTTACTCCCACAGG
TGAGCGGGCGGGACGGCCCTTCTCCTCCGGGCTGTAATTAGCGCTTGGTTTAATGACGGCTCGTTTCTTTTCTGTGGCTGCGTG
AAAGCCTTAAAGGGCTCCGGGAGGGCCCTTTGTGCGGGGGGGAGCGGCTCGGGGGGTGCGTGCGTGTGTGTGTGCGTGGGG
AGCGCCGCGTGCGGCCCGCGCTGCCCGGCGGCTGTGAGCGCTGCGGGCGCGGCGCGGGGCTTTGTGCGCTCCGCGTGTGC
GCGAGGGGAGCGCGGCCGGGGGCGGTGCCCCGCGGTGCGGGGGGGCTGCGAGGGGAACAAAGGCTGCGTGCGGGGTGTGT
GCGTGGGGGGGTGAGCAGGGGGTGTGGGCGCGGCGGTCGGGCTGTAACCCCCCCCTGCACCCCCCTCCCCGAGTTGCTGAG
CACGGCCCGGCTTCGGGTGCGGGGCTCCGTACGGGGCGTGGCGCGGGGCTCGCCGTGCCGGGCGGGGGGTGGCGGCAGGT
GGGGGTGCCGGGCGGGGCGGGGCCGCCTCGGGCCGGGGAGGGCTCGGGGGAGGGGCGCGGCGGCCCCCGGAGCGCCGG
CGGCTGTCGAGGCGCGGCGAGCCGCAGCCATTGCCTTTTATGGTAATCGTGCGAGAGGGCGCAGGGACTTCCTTTGTCCCAAA
TCTGTGCGGAGCCGAAATCTGGGAGGCGCCGCCGCACCCCCTCTAGCGGGCGCGGGGCGAAGCGGTGCGGCGCCGGCAGGA
AGGAAATGGGCGGGGAGGGCCTTCGTGCGTCGCCGCGCCGCCGTCCCCTTCTCCCTCTCCAGCCTCGGGGCTGTCCGCGGG
GGGACGGCTGCCTTCGGGGGGGACGGGGCAGGGCGGGGTTCGGCTTCTGGCGTGTGACCGGCGGCTCTAGAGCCTCTGCTA
ACCATGTTCATGCCTTCTTCTTTTTCCTACAGCTCCTGGGCAACGTGCTGGTTATTGTGCTGTCTCATCATTTTGGCAAAGAATTC
GCCACC**ATGGTGAGCAAGGGCGAGGAGGATAACATGGCCATCATCAAGGAGTTCATGCGCTTCAAGGTGCACATGGAGGGC**
TCCGTGAACGGCCACGAGTTCGAGATCGAGGGCGAGGGCGAGGGCCGCCCCTACGAGGGCACCCAGACCGCCAAGCTGAA
GGTGACCAAGGGTGGCCCCCTGCCCTTCGCCTGGGACATCCTGTCCCCTCAGTTCATGTACGGCTCCAAGGCCTACGTGAAG

CACCCCGCCGACATCCCCGACTACTTGAAGCTGTCCTTCCCCGAGGGCTTCAAGTGGGAGCGCGTGATGAACTTCGAGGACG
GCGGCGTGGTGACCGTGACCCAGGACTCCTCCCTGCAGGACGGCGAGTTCATCTACAAGGTGAAGCTGCGCGGCACCAACT
TCCCCTCCGACGGCCCCGTAATGCAGAAGAAGACCATGGGCTGGGAGGCCTCCTCCGAGCGGATGTACCCCGAGGACGGCG
CCCTGAAGGGCGAGATCAAGCAGAGGCTGAAGCTGAAGGACGGCGGCCACTACGACGCTGAGGTCAAGACCACCTACAAG
GCCAAGAAGCCCGTGCAGCTGCCCGGCGCCTACAACGTCAACATCAAGTTGGACATCACCTCCCACAACGAGGACTACACC
ATCGTGGAACAGTACGAACGCGCCGAGGGCCGCCACTCCACCGGCGGCATGGACGAGCTGTACGCGTCTGGAGGCGCCAC
CAATTTCAGCCTGCTGAAACAGGCTGGCGACGTGGAAGAGAACCCTGGACCTGGACAAAAGTCGTAGTCCAAAGGAGAAGA
ACTGTTTACCGGTGTTGTGCCAATTTTGGTTGAACTCGATGGTGATGTCAACGGACATAAGTTCTCAGTGAGAGGCGAAGGAG
AAGGTGACGCCACCATTGGAAAATTGACTCTTAAATTCATCTGTACTACTGGTAAACTTCCTGTACCATGGCCGACTCTCGTAA
CAACGCTTACGTACGGAGTTCAGTGCTTTTCGAGATACCCAGACCATATGAAAAGACATGACTTTTTTAAGTCGGCTATGCCTG
AAGGTTACGTGCAAGAAAGAACAATTTCGTTCAAAGATGATGGAAAATATAAAACTAGAGCAGTTGTTAAATTTGAAGGAGAT
ACTTTGGTTAACCGCATTGAACTGAAAGGAACAGATTTTAAAGAAGATGGTAATATTCTTGGACACAAACTCGAATACAATTTT
AATAGTCATAACGTATACATCACTGCTGATAAGCAAAAGAACGGAATTAAAGCGAATTTCACAGTACGCCATAATGTAGAAGA
TGGCAGTGTTCAACTTGCCGACCATTACCAACAAAACACCCCTATTGGAGACGGTCCGGTACTTCTTCCTGATAATCACTACCT
CTCAACACAAACAGTCCTGAGCAAAGATCCAAATGAAAAATGAGAATTCACTCCTCAGGTGCAGGCTGCCTATCAGAAGGTGG
TGGCTGGTGTGGCCAATGCCCTGGCTCACAAATACCACTGAGATCTTTTTCCCTCTGCCAAAAATTATGGGGACATCATGAAGCC
CCTTGAGCATCTGACTTCTGGCTAATAAAGGAAATTTATTTTCATTGCAATAGTGTGTTGGAATTTTTTGTGTCTCTCACTCGGAAG
GACATATGGGAGGGGTCGACAATCAACCTCTGGATTACAAAATTTGTGAAAGATTGACTGGTATTCTTAACTATGTTGCTCCTTTT
ACGCTATGTGGATACGCTGCTTTAATGCCTTTGTATCATGCGTTAACTAAACTTGTTTATTGCAGCTTATAATGGTTACAAATAAAG
CAATAGCATCACAAATTTCACAAATAAAGCATTTTTTTCACTGCATTCTAGTTGTGGTTTGTCCAAACTCATCAATGTATCTTATCAT
GTCTGGAATTGACTCAAATGATGTCAATTAGTCTATCAGAAGCTATCTGGTCTCCCTTCCGGGGGACAAGACATCCCTGTTTAATA
TTTAAACAGCAGTGTTCCCAAACTGGGTTCTTATATCCCTTGCTCTGGTCAACCAGGTTGCAGGGTTTCCTGTCCTCACAGGAAC
GAAGTCCCTAAAGAAACAGTGGCAGCCAGGTTTAGCCCCGGAATTGACTGGATTCCTTTTTTAGGGCCCATTGGTATGGCTTTTT
CCCCGTATCCCCCCAGGTGTCTGCAGGCTCAAAGAGCAGCGAGAAGCGTTCAGAGGAAAGCGATCCCGTGCCACCTTCCCCGT
GCCCGGGCTGTCCCCGCACGCTGCCGGCTCGGGGATGCGGGGGGAGCGCCGGACCGGAGCGGAGCCCCGGGCGGCTCGCT
GCTGCCCCCTAGCGGGGGAGGGACGTAATTACATCCCTGGGGGCTTTGGGGGGGGGCTGTCCCTGATATCTATAACAAGAAAA
TATATATATAATAAGTTATCACGTAAGTAGAACATGAAATAACAATATAATTATCGTATGAGTTAAATCTTAAAAGTCACGTAAAAGA
TAATCATGCGTCATTTTGACTCACGCGGTCGTTATAGTTCAAAATCAGTGACACTTACCGCATTGACAAGCACGCCTCACGGGAG
CTCCAAGCGGCGACTGAGATGTCCTAAATGCACAGCGACGGATTCGCGCTATTTAGAAAGAGAGAGCAATATTTCAAGAATGCAT
GCGTCAATTTTACGCAGACTATCTTTCTAGGGTTAATCTAGCTGCATCAGGATCATATCGTCGGGTCTTTTTTCCGGCTCAGTCAT
CGCCCAAGCTGGCGCTATCTGGGCATCGGGGAGGAAGAAGCCCGTGCCTTTTCCCGCGAGGTTGAAGCGGCATGGAAAGAGTT
TGCCGAGGATGACTGCTGCTGCATTGACGTTGAGCGAAAACGCACGTTTACCATGATGATTCGGGAAGGTGTGGCCATGCACGC
CTTTAACGGTGAACTGTTCGTTCAGGCCACCTGGGATACCAGTTCGTCGCGGCTTTTCCGGACACAGTTCCGGATGGTCAGCCC
GAAGCGCATCAGCAACCCGAACAATACCGGCGACAGCCGGAACTGCCGTGCCGGTGTGCAGATTAATGACAGCGGTGCGGCG
CTGGGATATTACGTCAGCGAGGACGGGTATCCTGGCTGGATGCCGCAGAAATGGACATGGATACCCCGTGAGTTACCCGGCGG
GCGCGCTTGGCGTAATCATGGTCATAGCTGTTTCCTGTGTGAAATTGTTATCCGCTCACAATTCCACACAACATACGAGCCGGAA
GCATAAAGTGTAAAGCCTGGGGTGCCTAATGAGTGAGCTAACTCACATTAATTGCGTTGCGCTCACTGCCCGCTTTCCAGTCGG
GAAACCTGTCGTGCCAGCTGCATTAATGAATCGGCCAACGCGCGGGGAGAGGCGGTTTGCGTATTGGGCGCTCTTCCGCTTCC
TCGCTCACTGACTCGCTGCGCTCGGTCGTTCGGCTGCGGCGAGCGGTATCAGCTCACTCAAAGGCGGTAATACGGTTATCCAC
AGAATCAGGGGATAACGCAGGAAAGAACATGTGAGCAAAAGGCCAGCAAAAGGCCAGGAACCGTAAAAAGGCCGCGTTGCTGG
CGTTTTTCCATAGGCTCCGCCCCCCTGACGAGCATCACAAAAATCGACGCTCAAGTCAGAGGTGGCGAAACCCGACAGGACTAT
AAAGATACCAGGCGTTTCCCCCTGGAAGCTCCCTCGTGCGCTCTCCTGTTCCGACCCTGCCGCTTACCGGATACCTGTCCGCCT
TTCTCCCTTCGGGAAGCGTGGCGCTTTCTCATAGCTCACGCTGTAGGTATCTCAGTTCGGTGTAGGTCGTTCGCTCCAAGCTGG
GCTGTGTGCACGAACCCCCCGTTCAGCCCGACCGCTGCGCCTTATCCGGTAACTATCGTCTTGAGTCCAACCCGGTAAGACACG
ACTTATCGCCACTGGCAGCAGCCACTGGTAACAGGATTAGCAGAGCGAGGTATGTAGGCGGTGCTACAGAGTTCTTGAAGTGGT
GGCCTAACTACGGCTACACTAGAAGGACAGTATTTGGTATCTGCGCTCTGCTGAAGCCAGTTACCTTCGGAAAAAGAGTTGGTA
GCTCTTGATCCGGCAAACAAACCACCGCTGGTAGCGGTGGTTTTTTTGTTTGCAAGCAGCAGATTACGCGCAGAAAAAAAGGAT
CTCAAGAAGATCCTTTGATCTTTTCTACGGGGTCTGACGCTCAGTGGAACGAAAACTCACGTTAAGGGATTTTGGTCATGAGATT
ATCAAAAAGGATCTTCACCTAGATCCTTTTAAATTAAAAATGAAGTTTTAAATCAATCTAAAGTATATATGAGTAAACTTGGTCTGAC
AGTTACCAATGCTTAATCAGTGAGGCACCTATCTCAGCGATCTGTCTATTTCGTTCATCCATAGTTGCCTGACTCCCCGTCGTGTA
GATAACTACGATACGGGAGGGCTTACCATCTGGCCCCAGTGCTGCAATGATACCGCGAGACCCACGCTCACCGGCTCCAGATTT
ATCAGCAATAAACCAGCCAGCCGGAAGGGCCGAGCGCAGAAGTGGTCCTGCAACTTTATCCGCCTCCATCCAGTCTATTAATTG
TTGCCGGGAAGCTAGAGTAAGTAGTTCGCCAGTTAATAGTTTGCGCAACGTTGTTGCCATTGCTACAGGCATCGTGGTGTCACG
CTCGTCGTTTGGTATGGCTTCATTCAGCTCCGGTTCCCAACGATCAAGGCGAGTTACATGATCCCCCATGTTGTGCAAAAAAGCG
GTTAGCTCCTTCGGTCCTCCGATCGTTGTCAGAAGTAAGTTGGCCGCAGTGTTATCACTCATGGTTATGGCAGCACTGCATAATT
CTCTTACTGTCATGCCATCCGTAAGATGCTTTTCTGTGACTGGTGAGTACTCAACCAAGTCATTCTGAGAATAGTGTATGCGGCG
ACCGAGTTGCTCTTGCCCGGCGTCAATACGGGATAATACCGCGCCACATAGCAGAACTTTAAAAGTGCTCATCATTGGAAAACGT
TCTTCGGGGCGAAAACTCTCAAGGATCTTACCGCTGTTGAGATCCAGTTCGATGTAACCCACTCGTGCACCCAACTGATCTTCAG
CATCTTTTACTTTCACCAGCGTTTCTGGGTGAGCAAAAACAGGAAGGCAAAATGCCGCAAAAAAGGGAATAAGGGCGACACGGA
AATGTTGAATACTCAT

2.2.3 Vector 56: 4x(Pyl tRNA$_{UCCU(Ev2)}$) PylRS mCherry-P2A-AGGA-sGFP(11)

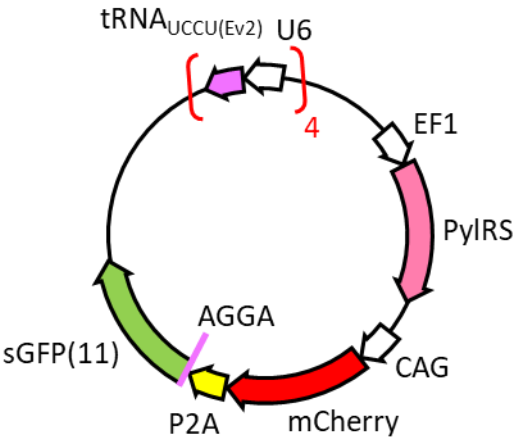

ACTCTTCCTTTTTCAATATTATTGAAGCATTTATCAGGGTTATTGTCTCATGAGCGGATACATATTTGAATGTATTTAGAAAAATAAA
CAAATAGGGGTTCCGCGCACATTTCCCCGAAAAGTGCCACCTAAATTGTAAGCGTTAATATTTTGTTAAAATTCGCGTTAAATTTTT
GTTAAATCAGCTCATTTTTTAACCAATAGGCCGAAATCGGCAAAATCCCTTATAAATCAAAAGAATAGACCGAGATAGGGTTGAGT
GTTGTTCCAGTTTGGAACAAGAGTCCACTATTAAAGAACGTGGACTCCAACGTCAAAGGGCGAAAAACCGTCTATCAGGGCGAT
GGCCCACTACGTGAACCATCACCCTAATCAAGTTTTTTGGGGTCGAGGTGCCGTAAAGCACTAAATCGGAACCCTAAAGGGAGC
CCCCGATTTAGAGCTTGACGGGGAAAGCCGGCGAACGTGGCGAGAAAGGAAGGGAAGAAAGCGAAAGGAGCGGGCGCTAGGG
CGCTGGCAAGTGTAGCGGTCACGCTGCGCGTAACCACCACACCCGCCGCGCTTAATGCGCCGCTACAGGGCGCGTCCCATTC
GCCATTCAGGCTGCGCAACTGTTGGGAAGGGCGATCGGTGCGGGCCTCTTCGCTATTACGCCAGCTGGCGAAAGGGGGATGT
GCTGCAAGGCGATTAAGTTGGGTAACGCCAGGGTTTTCCCAGTCACGACGTTGTAAAACGACGGCCAGTGAGCGCGCCTCGTT
CATTCACGTTTTTGAACCCGTGGAGGACGGGCAGACTCGCGGTGCAAATGTGTTTTACAGCGTGATGGAGCAGATGAAGATGCT
CGACACGCTGCAGAACACGCAGCTAGATTAACCCTAGAAAGATAATCATATTGTGACGTACGTTAAAGATAATCATGCGTAAAATT
GACGCATGTGTTTTATCGGTCTGTATATCGAGGTTTATTTATTAATTTGAATAGATATTAAGTTTTATTATATTTACACTTACATACTA
ATAATAAATTCAACAAACAATTTATTTATGTTTATTTATTTATTAAAAAAAAACAAAAACTCAAAATTTCTTCTATAAAGTAACAAAACTT
TTATGAGGGACAGCCCCCCCCCAAAGCCCCCAGGGATGTAATTACGTCCCTCCCCCGCTAGGGGGCAGCAGCGAGCCGCCCG
GGGCTCCGCTCCGGTCCGGCGCTCCCCCCGCATCCCCGAGCCGGCAGCGTGCGGGGACAGCCCGGGCACGGGGAAGGTGG
CACGGGATCGCTTTCCTCTGAACGCTTCTCGCTGCTCTTTGAGCCTGCAGACACCTGGGGGGATACGGGGAAAAGGCCTCTTAA
GAAAAACCGCACTTGTCCGGAAACCCCGGGAATCTAACCCGGCTGAAGCGGATAGGAAGCCCCTTCGATCTACATGATCAGG
TTTCCGGTGTTTCGTCCTTTCCACAAGATATATAAAGCCAAGAAATCGAAATACTTTCAAGTTACGGTAAGCATATGATAGTCCATT
TTAAAACATAATTTTAAAACTGCAAACTACCCAAGAAATTATTACTTTCTACGTCACGTATTTTGTACTAATATCTTTGTGTTTACAG
TCAAATTAATTCTAATTATCTCTCTAACAGCCTTGTATCGTATATGCAAATATGAAGGAATCATGGGAAATAGGCCCTCTTCCTGCC
CAATCGACGAACTTGTGGCCGTTTACCCTCTTAAGAAAAACCGCACTTGTCCGGAAACCCCGGGAATCTAACCCGGCTGAAGC
GGATAGGAAGCCCCTTCGATCTACATGATCAGGTTTCCGGTGTTTCGTCCTTTCCACAAGATATATAAAGCCAAGAAATCGAAAT
ACTTTCAAGTTACGGTAAGCATATGATAGTCCATTTTAAAACATAATTTTAAAACTGCAAACTACCCAAGAAATTATTACTTTCTACG
TCACGTATTTTGTACTAATATCTTTGTGTTTACAGTCAAATTAATTCTAATTATCTCTCTAACAGCCTTGTATCGTATATGCAAATAT
GAAGGAATCATGGGAAATAGGCCCTCTTCCTGCCCAATTCGGCAAGTTCTCCACCAGCCTCTTAAGAAAAACCGCACTTGTCCG
GAAACCCCGGGAATCTAACCCGGCTGAAGCGGATAGGAAGCCCCTTCGATCTACATGATCAGGTTTCCGGTGTTTCGTCCTTT
CCACAAGATATATAAAGCCAAGAAATCGAAATACTTTCAAGTTACGGTAAGCATATGATAGTCCATTTTAAAACATAATTTTAAAAC
TGCAAACTACCCAAGAAATTATTACTTTCTACGTCACGTATTTTGTACTAATATCTTTGTGTTTACAGTCAAATTAATTCTAATTATC
TCTCTAACAGCCTTGTATCGTATATGCAAATATGAAGGAATCATGGGAAATAGGCCCTCTTCCTGCCCAACCTCTTAAGAAAAACC
GCACTTGTCCCTAAAAACCGCACTTGTCCGGAAACCCCGGGAATCTAACCCGGCTGAAGCGGATAGGAAGCCCCTTCGATCTA
CATGATCAGGTTTCCGGTGTTTCGTCCTTTCCACAAGATATATAAAGCCAAGAAATCGAAATACTTTCAAGTTACGGTAAGCATAT
GATAGTCCATTTTAAAACATAATTTTAAAACTGCAAACTACCCAAGAAATTATTACTTTCTACGTCACGTATTTTGTACTAATATCTT
TGTGTTTACAGTCAAATTAATTCTAATTATCTCTCTAACAGCCTTGTATCGTATATGCAAATATGAAGGAATCATGGGAAATAGGCC
CTCTTCCTGCCCAACCGGTTGCGATCGCTCCGGTGCCCGTCAGTGGGCAGAGCGCACATCGCCCACAGTCCCCGAGAAGTTGG
GGGGAGGGGTCGGCAATTGAACGGGTGCCTAGAGAAGGTGGCGCGGGGTAAACTGGGAAAGTGATGTCGTGTACTGGCTCCG
CCTTTTTCCCGAGGGTGGGGGAGAACCGTATATAAGTGCAGTAGTCGCCGTGAACGTTCTTTTTCGCAACGGGTTTGCCGCCAG
AACACAGCTGAAGCTTCGAGGGGCTCGCATCTCTCCTTCACGCGCCCGCCGCCCTACCTGAGGCCGCCATCCACGCCGGTTGA
GTCGCGTTCTGCCGCCTCCCGCCTGTGGTGCCTCCTGAACTGCGTCCGCCGTCTAGGTAAGTTTAAAGCTCAGGTCGAGACCG
GGCCTTTGTCCGGCGCTCCCTTGGAGCCTACCTAGACTCAGCCGGCTCTCCACGCTTTGCCTGACCCTGCTTGCTCAACTCTAC
GTCTTTGTTTCGTTTTCTGTTCTGCGCCGTTACAGATCCAAGCTGTGACCGGCGCCTACTCTACAGATAGCGTTTAAACTTACGCT
TGCCACCATGGCTAGCGACTACAAGGACGACGACGACAAGGACAAGAAGCCCCTGAACACCCTGATCAGCGCCACAGGACT
GTGGATGTCCAGAACCGGCACCATCCACAAGATCAAGCACCACGAGGTGTCCCGGTCCAAAATCTACATCGAGATGGCCTGC
GGCGATCACCTGGTCGTCAACAACAGCAGAAGCAGCCGGACAGCCAGAGCCCTGCGGCACCACAAGTACAGAAAGACCTGC
AAGCGGTGCAGAGTGTCCGACGAGGACCTGAACAAGTTCCTGACCAAGGCCAACGAGGACCAGACCAGCGTGAAAGTGAAG
GTGGTGTCCGCCCCCACCCGGACCAAGAAAGCCATGCCCAAGAGCGTGGCCAGAGCCCCCAAGCCCCTGGAAAACACCGAA
GCCGCTCAGGCCCAGCCCAGCGGCAGCAAGTTCAGCCCCGCCATCCCCGTGTCTACCCAGGAAAGCGTCAGCGTCCCCGCC
AGCGTGTCCACCAGCATCTCTAGCATCTCAACCGGCGCCACAGCTTCTGCCCTGGTCAAGGGCAACACCAACCCCATCACCA
GCATGTCTGCCCCTGTGCAAGCCTCTGCCCCAGCCCTGACCAAGTCCCAGACCGACCGGCTGGAAGTGCTCCTGAACCCCAA
GGACGAGATCAGCCTGAACAGCGGCAAGCCCTTCCGGGAGCTGGAAAGCGAGCTGCTGAGCCGGCGGAAGAAGGACCTCC
AGCAAATCTACGCCGAGGAACGGGAGAACTACCTGGGCAAGCTGGAAAGAGAGATCACCCGGTTCTTCGTGGACCGGGGCT
TCCTGGAAATCAAGAGCCCCATCCTGATCCCCCTGGAGTACATCGAGCGGATGGGCATCGACAACGACACCGAGCTGAGCA
AGCAGATTTTCCGGGTGGACAAGAACTTCTGCCTGCGGCCCATGCTGGCCCCCAACCTGTACAACTACCTGCGGAAACTGGA
TCGCGCTCTGCCCGACCCCATCAAGATTTTCGAGATCGGCCCCTGCTACCGGAAAGAGAGCGACGGCAAAGAGCACCTGGA
AGAGTTTACAATGCTGAACTTTTGCCAGATGGGCAGCGGCTGCACCAGAGAGAACCTGGAATCCATCATCACCGACTTTCTGA

ACCACCTGGGGATCGACTTCAAGATCGTGGGCGACAGCTGCATGGTGTACGGCGACACCCTGGACGTGATGCACGGCGACC
TGGAACTGTCTAGCGCCGTCGTGGGACCCATCCCTCTGGACCGGGAGTGGGGCATCGATAAGCCCTGGATCGGAGCCGGCT
TCGGCCTGGAACGGCTGCTGAAAGTCAAGCACGACTTTAAGAACATCAAGCGGGCTGCCAGAAGCGAGAGCTACTACAACG
GCATCAGCACCAACCTGTGATGATAAGGATCCACTAGTCCAGTGTGGTGGAATTGACATTGATTATTGACTAGTTATTAATAGTA
ATCAATTACGGGGTCATTAGTTCATAGCCCATATATGGAGTTCCGCGTTACATAACTTACGGTAAATGGCCCGCCTGGCTGACCG
CCCAACGACCCCCGCCCATTGACGTCAATAATGACGTATGTTCCCATAGTAACGCCAATAGGGACTTTCCATTGACGTCAATGGG
TGGAGTATTTACGGTAAACTGCCCACTTGGCAGTACATCAAGTGTATCATATGCCAAGTACGCCCCCTATTGACGTCAATGACGG
TAAATGGCCCGCCTGGCATTATGCCCAGTACATGACCTTATGGGACTTTCCTACTTGGCAGTACATCTACGTATTAGTCATCGCT
ATTACCATGGTCGAGGTGAGCCCCACGTTCTGCTTCACTCTCCCCATCTCCCCCCCCTCCCCACCCCCAATTTTGTATTTATTTAT
TTTTTAATTATTTTGTGCAGCGATGGGGGCGGGGGGGGGGGGGGGGCGCGCGCCAGGCGGGGCGGGGCGGGGCGAGGGGCGG
GGCGGGGCGAGGCGGAGAGGTGCGGCGGCAGCCAATCAGAGCGGCGCGCTCCGAAAGTTTCCTTTTATGGCGAGGCGGCGG
CGGCGGCGGCCCTATAAAAAGCGAAGCGCGCGGCGGGCGGGAGTCGCTGCGTTGCCTTCGCCCCGTGCCCCGCTCCGCGCC
GCCTCGCGCCGCCCGCCCCGGCTCTGACTGACCGCGTTACTCCCACAGGTGAGCGGGCGGGACGGCCCTTCTCCTCCGGGCT
GTAATTAGCGCTTGGTTTAATGACGGCTCGTTTCTTTTCTGTGGCTGCGTGAAAGCCTTAAAGGGCTCCGGGAGGGCCCTTTGT
GCGGGGGGGAGCGGCTCGGGGGGTGCGTGCGTGTGTGTGTGCGTGGGGAGCGCCGCGTGCGGCCCGCGCTGCCCGGCGGC
TGTGAGCGCTGCGGGCGCGGCGCGGGGCTTTGTGCGCTCCGCGTGTGCGCGAGGGGAGCGCGGCCGGGGGCGGTGCCCCG
CGGTGCGGGGGGGCTGCGAGGGGAACAAAGGCTGCGTGCGGGGTGTGTGCGTGGGGGGGTGAGCAGGGGGTGTGGGCGCG
GCGGTCGGGCTGTAACCCCCCCCTGCACCCCCCTCCCCGAGTTGCTGAGCACGGCCCGGCTTCGGGTGCGGGGCTCCGTACG
GGGCGTGGCGCGGGGCTCGCCGTGCCGGGCGGGGGGTGGCGGCAGGTGGGGGTGCCGGGCGGGGCGGGGCCGCCTCGGG
CCGGGGAGGGCTCGGGGGAGGGGCGCGGCGGCCCCCGGAGCGCCGGCGGCTGTCGAGGCGCGGCGAGCCGCAGCCATTG
CCTTTTATGGTAATCGTGCGAGAGGGCGCAGGGACTTCCTTTGTCCCAAATCTGTGCGGAGCCGAAATCTGGGAGGCGCCGCC
GCACCCCCTCTAGCGGGCGCGGGGCGAAGCGGTGCGGCGCCGGCAGGAAGGAAATGGGCGGGGAGGGCCTTCGTGCGTCG
CCGCGCCGCCGTCCCCTTCTCCCTCTCCAGCCTCGGGGCTGTCCGCGGGGGGACGGCTGCCTTCGGGGGGGACGGGGCAGG
GCGGGGTTCGGCTTCTGGCGTGTGACCGGCGGCTCTAGAGCCTCTGCTAACCATGTTCATGCCTTCTTCTTTTTCCTACAGCTC
CTGGGCAACGTGCTGGTTATTGTGCTGTCTCATCATTTTGGCAAAGAATTCGCCACCATGGTGAGCAAGGGCGAGGAGGATAA
CATGGCCATCATCAAGGAGTTCATGCGCTTCAAGGTGCACATGGAGGGCTCCGTGAACGGCCACGAGTTCGAGATCGAGGG
CGAGGGCGAGGGCCGCCCCTACGAGGGCACCCAGACCGCCAAGCTGAAGGTGACCAAGGGTGGCCCCCTGCCCTTCGCCT
GGGACATCCTGTCCCCTCAGTTCATGTACGGCTCCAAGGCCTACGTGAAGCACCCCGCCGACATCCCCGACTACTTGAAGCT
GTCCTTCCCCGAGGGCTTCAAGTGGGAGCGCGTGATGAACTTCGAGGACGGCGGCGTGGTGACCGTGACCCAGGACTCCTC
CCTGCAGGACGGCGAGTTCATCTACAAGGTGAAGCTGCGCGGCACCAACTTCCCCTCCGACGGCCCCGTAATGCAGAAGAA
GACCATGGGCTGGGAGGCCTCCTCCGAGCGGATGTACCCCGAGGACGGCGCCCTGAAGGGCGAGATCAAGCAGAGGCTGA
AGCTGAAGGACGGCGGCCACTACGACGCTGAGGTCAAGACCACCTACAAGGCCAAGAAGCCCGTGCAGCTGCCCGGCGCCT
ACAACGTCAACATCAAGTTGGACATCACCTCCCACAACGAGGACTACACCATCGTGGAACAGTACGAACGCGCCGAGGGCC
GCCACTCCACCGGCGGCATGGACGAGCTGTACGCGTCTGGAGGCGCCACCAATTTCAGCCTGCTGAAACAGGCTGGCGACG
TGGAAGAGAACCCTGGACCTGGACAAAAGTCGAGGACGTGACCACATGGTCCTTCATGAGTATGTAAATGCTGCTGGGATTA
CAGGTGGCTCTGGAGGTAGAGATCATATGGTTCTCCACGAATACGTTAACGCCGCAGGCATCACTGGCGGTAGTGGAGGACG
CGACCATATGGTACTACATGAATATGTCAATGCAGCCGGAATAACCGGAGGGTCCGGAGGCCGGGATCACATGGTGCTGCAT
GAGTATGTGAACGCGGCGGGTATAACTGGTGGGTCGGGCGGACGAGACCATATGGTGCTTCACGAATACGTAAACGCAGCT
GGCATTACTGGCGGATCAGGTGGCAGGGATCACATGGTACTCCATGAGTACGTGAACGCTGCTGGAATCACAGGCGGTAGC
GGCGGTCGGGACCATATGGTCCTGCACGAATATGTCAATGCTGCCGGTATCACCGGCGGCAAATTCATGTGAGAATTCACTCC
TCAGGTGCAGGCTGCCTATCAGAAGGTGGTGGCTGGTGTGGCCAATGCCCTGGCTCACAAATACCACTGAGATCTTTTTCCCTC
TGCCAAAAATTATGGGGACATCATGAAGCCCCTTGAGCATCTGACTTCTGGCTAATAAAGGAAATTTATTTTCATTGCAATAGTGT
GTTGGAATTTTTTGTGTCTCTCACTCGGAAGGACATATGGGAGGGGTCGACAATCAACCTCTGGATTACAAAATTTGTGAAAGATT
GACTGGTATTCTTAACTATGTTGCTCCTTTTACGCTATGTGGATACGCTGCTTTAATGCCTTTGTATCATGCGTTAACTAAACTTGT
TTATTGCAGCTTATAATGGTTACAAATAAAGCAATAGCATCACAAATTTCACAAATAAAGCATTTTTTTCACTGCATTCTAGTTGTG
GTTTGTCCAAACTCATCAATGTATCTTATCATGTCTGGAATTGACTCAAATGATGTCAATTAGTCTATCAGAAGCTATCTGGTCTCC
CTTCCGGGGGACAAGACATCCCTGTTTAATATTTAAACAGCAGTGTTCCCAAACTGGGTTCTTATATCCCTTGCTCTGGTCAACCA
GGTTGCAGGGTTTCCTGTCCTCACAGGAACGAAGTCCCTAAAGAAACAGTGGCAGCCAGGTTTAGCCCCGGAATTGACTGGATT
CCTTTTTTAGGGCCCATTGGTATGGCTTTTTCCCCGTATCCCCCCAGGTGTCTGCAGGCTCAAAGAGCAGCGAGAAGCGTTCAG
AGGAAAGCGATCCCGTGCCACCTTCCCCGTGCCCGGGCTGTCCCCGCACGCTGCCGGCTCGGGGATGCGGGGGGAGCGCCG
GACCGGAGCGGAGCCCCGGGCGGCTCGCTGCTGCCCCCTAGCGGGGGAGGGACGTAATTACATCCCTGGGGGCTTTGGGGG
GGGGCTGTCCCTGATATCTATAACAAGAAAATATATATATAATAAGTTATCACGTAAGTAGAACATGAAATAACAATATAATTATCG
TATGAGTTAAATCTTAAAAGTCACGTAAAAGATAATCATGCGTCATTTTGACTCACGCGGTCGTTATAGTTCAAAATCAGTGACACT
TACCGCATTGACAAGCACGCCTCACGGGAGCTCCAAGCGGCGACTGAGATGTCCTAAATGCACAGCGACGGATTCGCGCTATTT
AGAAAGAGAGAGCAATATTTCAAGAATGCATGCGTCAATTTTACGCAGACTATCTTTCTAGGGTTAATCTAGCTGCATCAGGATCA
TATCGTCGGGTCTTTTTTCCGGCTCAGTCATCGCCCAAGCTGGCGCTATCTGGGCATCGGGGAGGAAGAAGCCCGTGCCTTTTC
CCGCGAGGTTGAAGCGGCATGGAAAGAGTTTGCCGAGGATGACTGCTGCTGCATTGACGTTGAGCGAAAACGCACGTTTACCAT
GATGATTCGGGAAGGTGTGGCCATGCACGCCTTTAACGGTGAACTGTTCGTTCAGGCCACCTGGGATACCAGTTCGTCGCGGCT
TTTCCGGACACAGTTCCGGATGGTCAGCCCGAAGCGCATCAGCAACCCGAACAATACCGGCGACAGCCGGAACTGCCGTGCCG
GTGTGCAGATTAATGACAGCGGTGCGGCGCTGGGATATTACGTCAGCGAGGACGGGTATCCTGGCTGGATGCCGCAGAAATGG
ACATGGATACCCCGTGAGTTACCCGGCGGGCGCGCTTGGCGTAATCATGGTCATAGCTGTTTCCTGTGTGAAATTGTTATCCGC
TCACAATTCCACACAACATACGAGCCGGAAGCATAAAGTGTAAAGCCTGGGGTGCCTAATGAGTGAGCTAACTCACATTAATTGC
GTTGCGCTCACTGCCCGCTTTCCAGTCGGGAAACCTGTCGTGCCAGCTGCATTAATGAATCGGCCAACGCGCGGGGAGAGGCG
GTTTGCGTATTGGGCGCTCTTCCGCTTCCTCGCTCACTGACTCGCTGCGCTCGGTCGTTCGGCTGCGGCGAGCGGTATCAGCT
CACTCAAAGGCGGTAATACGGTTATCCACAGAATCAGGGGATAACGCAGGAAAGAACATGTGAGCAAAAGGCCAGCAAAAGGCC
AGGAACCGTAAAAAGGCCGCGTTGCTGGCGTTTTTCCATAGGCTCCGCCCCCCTGACGAGCATCACAAAAATCGACGCTCAAGT
CAGAGGTGGCGAAACCCGACAGGACTATAAAGATACCAGGCGTTTCCCCCTGGAAGCTCCCTCGTGCGCTCTCCTGTTCCGAC
CCTGCCGCTTACCGGATACCTGTCCGCCTTTCTCCCTTCGGGAAGCGTGGCGCTTTCTCATAGCTCACGCTGTAGGTATCTCAG
TTCGGTGTAGGTCGTTCGCTCCAAGCTGGGCTGTGTGCACGAACCCCCCGTTCAGCCCGACCGCTGCGCCTTATCCGGTAACT
ATCGTCTTGAGTCCAACCCGGTAAGACACGACTTATCGCCACTGGCAGCAGCCACTGGTAACAGGATTAGCAGAGCGAGGTATG
TAGGCGGTGCTACAGAGTTCTTGAAGTGGTGGCCTAACTACGGCTACACTAGAAGGACAGTATTTGGTATCTGCGCTCTGCTGA
AGCCAGTTACCTTCGGAAAAAGAGTTGGTAGCTCTTGATCCGGCAAACAAACCACCGCTGGTAGCGGTGGTTTTTTTGTTTGCAA
GCAGCAGATTACGCGCAGAAAAAAAGGATCTCAAGAAGATCCTTTGATCTTTTCTACGGGGTCTGACGCTCAGTGGAACGAAAAC
TCACGTTAAGGGATTTTGGTCATGAGATTATCAAAAAGGATCTTCACCTAGATCCTTTTAAATTAAAAATGAAGTTTTAAATCAATC

TAAAGTATATATGAGTAAACTTGGTCTGACAGTTACCAATGCTTAATCAGTGAGGCACCTATCTCAGCGATCTGTCTATTTCGTTC
ATCCATAGTTGCCTGACTCCCCGTCGTGTAGATAACTACGATACGGGAGGGCTTACCATCTGGCCCCAGTGCTGCAATGATACC
GCGAGACCCACGCTCACCGGCTCCAGATTTATCAGCAATAAACCAGCCAGCCGGAAGGGCCGAGCGCAGAAGTGGTCCTGCAA
CTTTATCCGCCTCCATCCAGTCTATTAATTGTTGCCGGGAAGCTAGAGTAAGTAGTTCGCCAGTTAATAGTTTGCGCAACGTTGTT
GCCATTGCTACAGGCATCGTGGTGTCACGCTCGTCGTTTGGTATGGCTTCATTCAGCTCCGGTTCCCAACGATCAAGGCGAGTT
ACATGATCCCCCATGTTGTGCAAAAAAGCGGTTAGCTCCTTCGGTCCTCCGATCGTTGTCAGAAGTAAGTTGGCCGCAGTGTTAT
CACTCATGGTTATGGCAGCACTGCATAATTCTCTTACTGTCATGCCATCCGTAAGATGCTTTTCTGTGACTGGTGAGTACTCAACC
AAGTCATTCTGAGAATAGTGTATGCGGCGACCGAGTTGCTCTTGCCCGGCGTCAATACGGGATAATACCGCGCCACATAGCAGA
ACTTTAAAAGTGCTCATCATTGGAAAACGTTCTTCGGGGCGAAAACTCTCAAGGATCTTACCGCTGTTGAGATCCAGTTCGATGT
AACCCACTCGTGCACCCAACTGATCTTCAGCATCTTTTACTTTCACCAGCGTTTCTGGGTGAGCAAAAACAGGAAGGCAAAATGC
CGCAAAAAAGGGAATAAGGGCGACACGGAAATGTTGAATACTCAT

2.2.4 Vector 57: 4x(Tyr $tRNA_{CUA}$) TyrRS* sGFP(1-10)-TAG-sGFP(11)

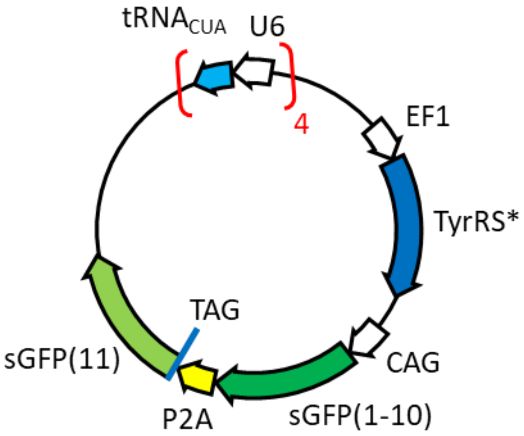

ACTCTTCCTTTTTCAATATTATTGAAGCATTTATCAGGGTTATTGTCTCATGAGCGGATACATATTTGAATGTATTTAGAAAAATAAA
CAAATAGGGGTTCCGCGCACATTTCCCCGAAAAGTGCCACCTAAATTGTAAGCGTTAATATTTTGTTAAAATTCGCGTTAAATTTTT
GTTAAATCAGCTCATTTTTTAACCAATAGGCCGAAATCGGCAAAATCCCTTATAAATCAAAAGAATAGACCGAGATAGGGTTGAGT
GTTGTTCCAGTTTGGAACAAGAGTCCACTATTAAAGAACGTGGACTCCAACGTCAAAGGGCGAAAAACCGTCTATCAGGGCGAT
GGCCCACTACGTGAACCATCACCCTAATCAAGTTTTTTGGGGTCGAGGTGCCGTAAAGCACTAAATCGGAACCCTAAAGGGAGC
CCCCGATTTAGAGCTTGACGGGGAAAGCCGGCGAACGTGGCGAGAAAGGAAGGGAAGAAAGCGAAAGGAGCGGGCGCTAGGG
CGCTGGCAAGTGTAGCGGTCACGCTGCGCGTAACCACCACACCCGCCGCGCTTAATGCGCCGCTACAGGGCGCGTCCCATTC
GCCATTCAGGCTGCGCAACTGTTGGGAAGGGCGATCGGTGCGGGCCTCTTCGCTATTACGCCAGCTGGCGAAAGGGGGATGT
GCTGCAAGGCGATTAAGTTGGGTAACGCCAGGGTTTTCCCAGTCACGACGTTGTAAAACGACGGCCAGTGAGCGCGCCTCGTT
CATTCACGTTTTTGAACCCGTGGAGGACGGGCAGACTCGCGGTGCAAATGTGTTTTACAGCGTGATGGAGCAGATGAAGATGCT
CGACACGCTGCAGAACACGCAGCTAGATTAACCCTAGAAAGATAATCATATTGTGACGTACGTTAAAGATAATCATGCGTAAAATT
GACGCATGTGTTTTATCGGTCTGTATATCGAGGTTTATTTATTAATTTGAATAGATATTAAGTTTTATTATATTTACACTTACATACTA
ATAATAAATTCAACAAACAATTTATTTATGTTTATTTATTTATTAAAAAAAAACAAAAACTCAAAATTTCTTCTATAAAGTAACAAAACTT
TTATGAGGGACAGCCCCCCCCCAAAGCCCCCAGGGATGTAATTACGTCCCTCCCCCGCTAGGGGGCAGCAGCGAGCCGCCCG
GGGCTCCGCTCCGGTCCGGCGCTCCCCCCGCATCCCCGAGCCGGCAGCGTGCGGGGACAGCCCGGGCACGGGGAAGGTGG
CACGGGATCGCTTTCCTCTGAACGCTTCTCGCTGCTCTTTGAGCCTGCAGACACCTGGGGGGATACGGGGAAAAGGCCTCTTAA
GAAAAACCGCACTTGTC**TGGTGGGGGAAGGATTCGAACCTTCGAAGTCTGTGACGGCAGATTTAGAGTCTGCTCCCTTTGGCC**
GCTCGGGAACCCCACCGGTGTTTCGTCCTTTCCACAAGATATATAAAGCCAAGAAATCGAAATACTTTCAAGTTACGGTAAGCAT
ATGATAGTCCATTTTAAAACATAATTTTAAAACTGCAAACTACCCAAGAAATTATTACTTTCTACGTCACGTATTTTGTACTAATATC
TTTGTGTTTACAGTCAAATTAATTCTAATTATCTCTCTAACAGCCTTGTATCGTATATGCAAATATGAAGGAATCATGGGAAATAGG
CCCTCTTCCTGCCCAATCGACGAACTTGTGGCCGTTTACCCTCTTAAGAAAAACCGCACTTGTC**TGGTGGGGGAAGGATTCGAA**
CCTTCGAAGTCTGTGACGGCAGATTTAGAGTCTGCTCCCTTTGGCCGCTCGGGAACCCCACCGGTGTTTCGTCCTTTCCACAA
GATATATAAAGCCAAGAAATCGAAATACTTTCAAGTTACGGTAAGCATATGATAGTCCATTTTAAAACATAATTTTAAAACTGCAAA
CTACCCAAGAAATTATTACTTTCTACGTCACGTATTTTGTACTAATATCTTTGTGTTTACAGTCAAATTAATTCTAATTATCTCTCTAA
CAGCCTTGTATCGTATATGCAAATATGAAGGAATCATGGGAAATAGGCCCTCTTCCTGCCCAATTCGGCAAGTTCTCCACCAGCC
TCTTAAGAAAAACCGCACTTGTC**TGGTGGGGGAAGGATTCGAACCTTCGAAGTCTGTGACGGCAGATTTAGAGTCTGCTCCCTT**
TGGCCGCTCGGGAACCCCACCGGTGTTTCGTCCTTTCCACAAGATATATAAAGCCAAGAAATCGAAATACTTTCAAGTTACGGTA
AGCATATGATAGTCCATTTTAAAACATAATTTTAAAACTGCAAACTACCCAAGAAATTATTACTTTCTACGTCACGTATTTTGTACTA
ATATCTTTGTGTTTACAGTCAAATTAATTCTAATTATCTCTCTAACAGCCTTGTATCGTATATGCAAATATGAAGGAATCATGGGAA
ATAGGCCCTCTTCCTGCCCAACCTCTTAAGAAAAACCGCACTTGTCCCTAAAAACCGCACTTGTC**TGGTGGGGGAAGGATTCGA**
ACCTTCGAAGTCTGTGACGGCAGATTTAGAGTCTGCTCCCTTTGGCCGCTCGGGAACCCCACCGGTGTTTCGTCCTTTCCACA
AGATATATAAAGCCAAGAAATCGAAATACTTTCAAGTTACGGTAAGCATATGATAGTCCATTTTAAAACATAATTTTAAAACTGCAA
ACTACCCAAGAAATTATTACTTTCTACGTCACGTATTTTGTACTAATATCTTTGTGTTTACAGTCAAATTAATTCTAATTATCTCTCTA
ACAGCCTTGTATCGTATATGCAAATATGAAGGAATCATGGGAAATAGGCCCTCTTCCTGCCCAACCGGTTGCGATCGCTCCGGT
GCCCGTCAGTGGGCAGAGCGCACATCGCCCACAGTCCCCGAGAAGTTGGGGGGAGGGGTCGGCAATTGAACGGGTGCCTAGA
GAAGGTGGCGCGGGGTAAACTGGGAAAGTGATGTCGTGTACTGGCTCCGCCTTTTTCCCGAGGGTGGGGGAGAACCGTATATA
AGTGCAGTAGTCGCCGTGAACGTTCTTTTTCGCAACGGGTTTGCCGCCAGAACACAGCTGAAGCTTCGAGGGGCTCGCATCTCT
CCTTCACGCGCCCGCCGCCCTACCTGAGGCCGCCATCCACGCCGGTTGAGTCGCGTTCTGCCGCCTCCCGCCTGTGGTGCCT
CCTGAACTGCGTCCGCCGTCTAGGTAAGTTTAAAGCTCAGGTCGAGACCGGGCCTTTGTCCGGCGCTCCCTTGGAGCCTACCTA
GACTCAGCCGGCTCTCCACGCTTTGCCTGACCCTGCTTGCTCAACTCTACGTCTTTGTTTCGTTTTCTGTTCTGCGCCGTTACAG
ATCCAAGCTGTGACCGGCGCCTACTCTACAGATAGCGTTTAAACTTACGCTTGCCACC**ATGGCTAGCGACTACAAGGACGACG**

ACGACAAGGCAAGCAGTAACTTGATTAAACAATTGCAAGAGCGGGGGCTGGTAGCCCAGGTGACGGACGAGGAAGCGTTAG
CAGAGCGACTGGCGCAAGGCCCGATCGCACTCGTGTGTGGCTTCGATCCTACCGCTGACAGCTTGCATTTGGGGCATCTTGTT
CCATTGTTATGCCTGAAACGCTTCCAGCAGGCGGGCCACAAGCCGGTTGCGCTGGTAGGCGGCGCGACGGGTCTGATTGGC
GACCCGAGCTTCAAAGCTGCCGAGCGTAAGCTGAACACCGAAGAAACTGTTCAGGAGTGGGTGGACAAAATCCGTAAGCAG
GTTGCCCCGTTCCTCGATTTCGACTGTGGAGAAAACTCTGCTATCGCGGCCAATAATTATGACTGGTTCGGCAATATGAATGT
GCTGACCTTCCTGCGCGATATTGGCAAACACTTCTCCGTTAACCAGATGATCAACAAAGAAGCGGTTAAGCAGCGTCTCAACC
GTGAAGATCAGGGGATTTCGTTCACTGAGTTTTCCTACAACCTGCTGCAGGGTTATAGTATGGCCTGTTTGAACAAACAGTAC
GGTGTGGTGCTGCAAATTGGTGGTTCTGACCAGTGGGGTAACATCACTTCTGGTATCGACCTGACCCGTCGTCTGCATCAGAA
TCAGGTGTTTGGCCTGACCGTTCCGCTGATCACTAAAGCAGATGGCACCAAATTTGGTAAAACTGAAGGCGGCGCAGTCTGG
TTGGATCCGAAGAAAACCAGCCCGTACAAATTCTACCAGTTCTGGATCAACACTGCGCGTGCCGACGTTTACCGCTTCCTGAA
GTTCTTCACCTTTATGAGCATTGAAGAGATCAACGCCCTGGAAGAAGAAGATAAAAACAGCGGTAAAGCACCGCGCGCCCAG
TATGTACTGGCGGAGCAGGTGACTCGTCTGGTTCACGGTGAAGAAGGTTTACAGGCGGCAAAACGTATTACCGAATGCCTGT
TCAGCGGTTCTTTGAGTGCGCTGAGTGAAGCGGACTTCGAACAGCTGGCGCAGGACGGCGTACCGATGGTTGAGATGGAAAA
GGGCGCAGACCTGATGCAGGCACTGGTCGATTCTGAACTGCAACCTTCCCGTGGTCAGGCACGTAAAACTATCGCCTCCAAT
GCCATCACCATTAACGGTGAAAAACAGTCCGATCCTGAATACTTCTTTAAAGAAGAAGATCGTCTGTTTGGTCGTTTTACCTTA
CTGCGTCGCGGTAAAAAGAATTACTGTCTGATTTGCTGGAAAGGGCCCGTTTAATGATAAGGATCCACTAGTCCAGTGTGGTG
GAATTGACATTGATTATTGACTAGTTATTAATAGTAATCAATTACGGGGTCATTAGTTCATAGCCCATATATGGAGTTCCGCGTTAC
ATAACTTACGGTAAATGGCCCGCCTGGCTGACCGCCCAACGACCCCCGCCCATTGACGTCAATAATGACGTATGTTCCCATAGT
AACGCCAATAGGGACTTTCCATTGACGTCAATGGGTGGAGTATTTACGGTAAACTGCCCACTTGGCAGTACATCAAGTGTATCAT
ATGCCAAGTACGCCCCCTATTGACGTCAATGACGGTAAATGGCCCGCCTGGCATTATGCCCAGTACATGACCTTATGGGACTTTC
CTACTTGGCAGTACATCTACGTATTAGTCATCGCTATTACCATGGTCGAGGTGAGCCCCACGTTCTGCTTCACTCTCCCCATCTC
CCCCCCCTCCCCACCCCCAATTTTGTATTTATTTATTTTTTAATTATTTTGTGCAGCGATGGGGGCGGGGGGGGGGGGGGCGCG
CGCCAGGCGGGGCGGGGCGGGGCGAGGGGCGGGGCGGGGCGAGGCGGAGAGGTGCGGCGGCAGCCAATCAGAGCGGCGC
GCTCCGAAAGTTTCCTTTTATGGCGAGGCGGCGGCGGCGGCGGCCCTATAAAAAGCGAAGCGCGCGGCGGGCGGGAGTCGCT
GCGTTGCCTTCGCCCCGTGCCCCGCTCCGCGCCGCCTCGCGCCGCCCGCCCCGGCTCTGACTGACCGCGTTACTCCCACAGG
TGAGCGGGCGGGACGGCCCTTCTCCTCCGGGCTGTAATTAGCGCTTGGTTTAATGACGGCTCGTTTCTTTTCTGTGGCTGCGTG
AAAGCCTTAAAGGGCTCCGGGAGGGCCCTTTGTGCGGGGGGGAGCGGCTCGGGGGGTGCGTGCGTGTGTGTGTGCGTGGGG
AGCGCCGCGTGCGGCCCGCGCTGCCCGGCGGCTGTGAGCGCTGCGGGCGCGGCGCGGGGCTTTGTGCGCTCCGCGTGTGC
GCGAGGGGAGCGCGGCCGGGGGCGGTGCCCCGCGGTGCGGGGGGGCTGCGAGGGGAACAAAGGCTGCGTGCGGGGTGTGT
GCGTGGGGGGGTGAGCAGGGGGTGTGGGCGCGGCGGTCGGGCTGTAACCCCCCCCTGCACCCCCCTCCCCGAGTTGCTGAG
CACGGCCCGGCTTCGGGTGCGGGGCTCCGTACGGGGCGTGGCGCGGGGCTCGCCGTGCCGGGCGGGGGGTGGCGGCAGGT
GGGGGTGCCGGGCGGGGCGGGGCCGCCTCGGGCCGGGGAGGGCTCGGGGGAGGGGCGCGGCGGCCCCCGGAGCGCCGG
CGGCTGTCGAGGCGCGGCGAGCCGCAGCCATTGCCTTTTATGGTAATCGTGCGAGAGGGCGCAGGGACTTCCTTTGTCCCAAA
TCTGTGCGGAGCCGAAATCTGGGAGGCGCCGCCGCACCCCCTCTAGCGGGCGCGGGGCGAAGCGGTGCGGCGCCGGCAGGA
AGGAAATGGGCGGGGAGGGCCTTCGTGCGTCGCCGCGCCGCCGTCCCCTTCTCCCTCTCCAGCCTCGGGGCTGTCCGCGGG
GGGACGGCTGCCTTCGGGGGGGACGGGGCAGGGCGGGGTTCGGCTTCTGGCGTGTGACCGGCGGCTCTAGAGCCTCTGCTA
ACCATGTTCATGCCTTCTTCTTTTTCCTACAGCTCCTGGGCAACGTGCTGGTTATTGTGCTGTCTCATCATTTTGGCAAAGAATTC
GCCACC**ATGTCCAAAGGAGAAGAACTGTTTACCGGTGTTGTGCCAATTTTGGTTGAACTCGATGGTGATGTCAACGGACATAA**
GTTCTCAGTGAGAGGCGAAGGAGAAGGTGACGCCACCATTGGAAAATTGACTCTTAAATTCATCTGTACTACTGGTAAACTTC
CTGTACCATGGCCGACTCTCGTAACAACGCTTACGTACGGAGTTCAGTGCTTTTCGAGATACCCAGACCATATGAAAAGACAT
GACTTTTTTAAGTCGGCTATGCCTGAAGGTTACGTGCAAGAAAGAACAATTTCGTTCAAAGATGATGGAAAATATAAAACTAG
AGCAGTTGTTAAATTTGAAGGAGATACTTTGGTTAACCGCATTGAACTGAAAGGAACAGATTTTAAAGAAGATGGTAATATTCT
TGGACACAAACTCGAATACAATTTTAATAGTCATAACGTATACATCACTGCTGATAAGCAAAAGAACGGAATTAAAGCGAATT
TCACAGTACGCCATAATGTAGAAGATGGCAGTGTTCAACTTGCCGACCATTACCAACAAAACACCCCTATTGGAGACGGTCCG
GTACTTCTTCCTGATAATCACTACCTCTCAACACAAACAGTCCTGAGCAAAGATCCAAATGAAAAAGCGTCTGGAGGCGCCAC
CAATTTCAGCCTGCTGAAACAGGCTGGCGACGTGGAAGAGAACCCTGGACCTGGACAAAAGTCGTAGCGTGACCACATGGTC
CTTCATGAGTATGTAAATGCTGCTGGGATTACAGGTGGCTCTGGAGGTAGAGATCATATGGTTCTCCACGAATACGTTAACGC
CGCAGGCATCACTGGCGGTAGTGGAGGACGCGACCATATGGTACTACATGAATATGTCAATGCAGCCGGAATAACCGGAGG
GTCCGGAGGCCGGGATCACATGGTGCTGCATGAGTATGTGAACGCGGCGGGTATAACTGGTGGGTCGGGCGGACGAGACCA
TATGGTGCTTCACGAATACGTAAACGCAGCTGGCATTACTGGCGGATCAGGTGGCAGGGATCACATGGTACTCCATGAGTAC
GTGAACGCTGCTGGAATCACAGGCGGTAGCGGCGGTCGGGACCATATGGTCCTGCACGAATATGTCAATGCTGCCGGTATCA
CCGGCGGCAAATTCATGTGAGAATTCACTCCTCAGGTGCAGGCTGCCTATCAGAAGGTGGTGGCTGGTGTGGCCAATGCCCTG
GCTCACAAATACCACTGAGATCTTTTTCCCTCTGCCAAAAATTATGGGGACATCATGAAGCCCCTTGAGCATCTGACTTCTGGCTA
ATAAAGGAAATTTATTTTCATTGCAATAGTGTGTTGGAATTTTTTGTGTCTCTCACTCGGAAGGACATATGGGAGGGGTCGACAAT
CAACCTCTGGATTACAAAATTTGTGAAAGATTGACTGGTATTCTTAACTATGTTGCTCCTTTTACGCTATGTGGATACGCTGCTTTA
ATGCCTTTGTATCATGCGTTAACTAAACTTGTTTATTGCAGCTTATAATGGTTACAAATAAAGCAATAGCATCACAAATTTCACAAA
TAAAGCATTTTTTTCACTGCATTCTAGTTGTGGTTTGTCCAAACTCATCAATGTATCTTATCATGTCTGGAATTGACTCAAATGATG
TCAATTAGTCTATCAGAAGCTATCTGGTCTCCCTTCCGGGGGACAAGACATCCCTGTTTAATATTTAAACAGCAGTGTTCCCAAAC
TGGGTTCTTATATCCCTTGCTCTGGTCAACCAGGTTGCAGGGTTTCCTGTCCTCACAGGAACGAAGTCCCTAAAGAAACAGTGGC
AGCCAGGTTTAGCCCCGGAATTGACTGGATTCCTTTTTTAGGGCCCATTGGTATGGCTTTTTCCCCGTATCCCCCCAGGTGTCTG
CAGGCTCAAAGAGCAGCGAGAAGCGTTCAGAGGAAAGCGATCCCGTGCCACCTTCCCCGTGCCCGGGCTGTCCCCGCACGCT
GCCGGCTCGGGGATGCGGGGGGAGCGCCGGACCGGAGCGGAGCCCCGGGCGGCTCGCTGCTGCCCCCTAGCGGGGGAGGG
ACGTAATTACATCCCTGGGGGCTTTGGGGGGGGGCTGTCCCTGATATCTATAACAAGAAAATATATATATAATAAGTTATCACGTA
AGTAGAACATGAAATAACAATATAATTATCGTATGAGTTAAATCTTAAAAGTCACGTAAAAGATAATCATGCGTCATTTTGACTCAC
GCGGTCGTTATAGTTCAAAATCAGTGACACTTACCGCATTGACAAGCACGCCTCACGGGAGCTCCAAGCGGCGACTGAGATGTC
CTAAATGCACAGCGACGGATTCGCGCTATTTAGAAAGAGAGAGCAATATTTCAAGAATGCATGCGTCAATTTTACGCAGACTATC
TTTCTAGGGTTAATCTAGCTGCATCAGGATCATATCGTCGGGTCTTTTTTCCGGCTCAGTCATCGCCCAAGCTGGCGCTATCTGG
GCATCGGGGAGGAAGAAGCCCGTGCCTTTTCCCGCGAGGTTGAAGCGGCATGGAAAGAGTTTGCCGAGGATGACTGCTGCTGC
ATTGACGTTGAGCGAAAACGCACGTTTACCATGATGATTCGGGAAGGTGTGGCCATGCACGCCTTTAACGGTGAACTGTTCGTT
CAGGCCACCTGGGATACCAGTTCGTCGCGGCTTTTCCGGACACAGTTCCGGATGGTCAGCCCGAAGCGCATCAGCAACCCGAA
CAATACCGGCGACAGCCGGAACTGCCGTGCCGGTGTGCAGATTAATGACAGCGGTGCGGCGCTGGGATATTACGTCAGCGAG
GACGGGTATCCTGGCTGGATGCCGCAGAAATGGACATGGATACCCCGTGAGTTACCCGGCGGGCGCGCTTGGCGTAATCATGG
TCATAGCTGTTTCCTGTGTGAAATTGTTATCCGCTCACAATTCCACACAACATACGAGCCGGAAGCATAAAGTGTAAAGCCTGGG
GTGCCTAATGAGTGAGCTAACTCACATTAATTGCGTTGCGCTCACTGCCCGCTTTCCAGTCGGGAAACCTGTCGTGCCAGCTGC

ATTAATGAATCGGCCAACGCGCGGGGAGAGGCGGTTTGCGTATTGGGCGCTCTTCCGCTTCCTCGCTCACTGACTCGCTGCGC
TCGGTCGTTCGGCTGCGGCGAGCGGTATCAGCTCACTCAAAGGCGGTAATACGGTTATCCACAGAATCAGGGGATAACGCAGG
AAAGAACATGTGAGCAAAAGGCCAGCAAAAGGCCAGGAACCGTAAAAAGGCCGCGTTGCTGGCGTTTTTCCATAGGCTCCGCC
CCCCTGACGAGCATCACAAAAATCGACGCTCAAGTCAGAGGTGGCGAAACCCGACAGGACTATAAAGATACCAGGCGTTTCCCC
CTGGAAGCTCCCTCGTGCGCTCTCCTGTTCCGACCCTGCCGCTTACCGGATACCTGTCCGCCTTTCTCCCTTCGGGAAGCGTGG
CGCTTTCTCATAGCTCACGCTGTAGGTATCTCAGTTCGGTGTAGGTCGTTCGCTCCAAGCTGGGCTGTGTGCACGAACCCCCCG
TTCAGCCCGACCGCTGCGCCTTATCCGGTAACTATCGTCTTGAGTCCAACCCGGTAAGACACGACTTATCGCCACTGGCAGCAG
CCACTGGTAACAGGATTAGCAGAGCGAGGTATGTAGGCGGTGCTACAGAGTTCTTGAAGTGGTGGCCTAACTACGGCTACACTA
GAAGGACAGTATTTGGTATCTGCGCTCTGCTGAAGCCAGTTACCTTCGGAAAAAGAGTTGGTAGCTCTTGATCCGGCAAACAAAC
CACCGCTGGTAGCGGTGGTTTTTTTGTTTGCAAGCAGCAGATTACGCGCAGAAAAAAAGGATCTCAAGAAGATCCTTTGATCTTT
TCTACGGGGTCTGACGCTCAGTGGAACGAAAACTCACGTTAAGGGATTTTGGTCATGAGATTATCAAAAAGGATCTTCACCTAGA
TCCTTTTAAATTAAAAATGAAGTTTTAAATCAATCTAAAGTATATATGAGTAAACTTGGTCTGACAGTTACCAATGCTTAATCAGTGA
GGCACCTATCTCAGCGATCTGTCTATTTCGTTCATCCATAGTTGCCTGACTCCCCGTCGTGTAGATAACTACGATACGGGAGGGC
TTACCATCTGGCCCCAGTGCTGCAATGATACCGCGAGACCCACGCTCACCGGCTCCAGATTTATCAGCAATAAACCAGCCAGCC
GGAAGGGCCGAGCGCAGAAGTGGTCCTGCAACTTTATCCGCCTCCATCCAGTCTATTAATTGTTGCCGGGAAGCTAGAGTAAGT
AGTTCGCCAGTTAATAGTTTGCGCAACGTTGTTGCCATTGCTACAGGCATCGTGGTGTCACGCTCGTCGTTTGGTATGGCTTCAT
TCAGCTCCGGTTCCCAACGATCAAGGCGAGTTACATGATCCCCCATGTTGTGCAAAAAAGCGGTTAGCTCCTTCGGTCCTCCGA
TCGTTGTCAGAAGTAAGTTGGCCGCAGTGTTATCACTCATGGTTATGGCAGCACTGCATAATTCTCTTACTGTCATGCCATCCGTA
AGATGCTTTTCTGTGACTGGTGAGTACTCAACCAAGTCATTCTGAGAATAGTGTATGCGGCGACCGAGTTGCTCTTGCCCGGCGT
CAATACGGGATAATACCGCGCCACATAGCAGAACTTTAAAAGTGCTCATCATTGGAAAACGTTCTTCGGGGCGAAAACTCTCAAG
GATCTTACCGCTGTTGAGATCCAGTTCGATGTAACCCACTCGTGCACCCAACTGATCTTCAGCATCTTTTACTTTCACCAGCGTTT
CTGGGTGAGCAAAAACAGGAAGGCAAAATGCCGCAAAAAAGGGAATAAGGGCGACACGGAAATGTTGAATACTCAT

2.2.5 Vector 58: Pyl $tRNA_{CUA(U25C)}$ PylRS mCherry-P2A-eGFP(150TAG)

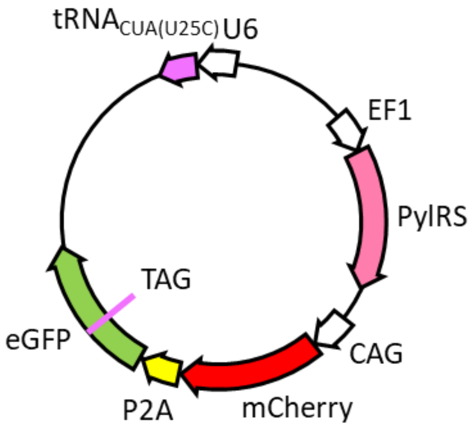

ACTCTTCCTTTTTCAATATTATTGAAGCATTTATCAGGGTTATTGTCTCATGAGCGGATACATATTTGAATGTATTTAGAAAAATAAA
CAAATAGGGGTTCCGCGCACATTTCCCCGAAAAGTGCCACCTAAATTGTAAGCGTTAATATTTTGTTAAAATTCGCGTTAAATTTTT
GTTAAATCAGCTCATTTTTTAACCAATAGGCCGAAATCGGCAAAATCCCTTATAAATCAAAAGAATAGACCGAGATAGGGTTGAGT
GTTGTTCCAGTTTGGAACAAGAGTCCACTATTAAAGAACGTGGACTCCAACGTCAAAGGGCGAAAAACCGTCTATCAGGGCGAT
GGCCCACTACGTGAACCATCACCCTAATCAAGTTTTTTGGGGTCGAGGTGCCGTAAAGCACTAAATCGGAACCCTAAAGGGAGC
CCCCGATTTAGAGCTTGACGGGGAAAGCCGGCGAACGTGGCGAGAAAGGAAGGGAAGAAAGCGAAAGGAGCGGGCGCTAGGG
CGCTGGCAAGTGTAGCGGTCACGCTGCGCGTAACCACCACACCCGCCGCGCTTAATGCGCCGCTACAGGGCGCGTCCCATTC
GCCATTCAGGCTGCGCAACTGTTGGGAAGGGCGATCGGTGCGGGCCTCTTCGCTATTACGCCAGCTGGCGAAAGGGGGATGT
GCTGCAAGGCGATTAAGTTGGGTAACGCCAGGGTTTTCCCAGTCACGACGTTGTAAAACGACGGCCAGTGAGCGCGCCTCGTT
CATTCACGTTTTTGAACCCGTGGAGGACGGGCAGACTCGCGGTGCAAATGTGTTTTACAGCGTGATGGAGCAGATGAAGATGCT
CGACACGCTGCAGAACACGCAGCTAGATTAACCCTAGAAAGATAATCATATTGTGACGTACGTTAAAGATAATCATGCGTAAAATT
GACGCATGTGTTTTATCGGTCTGTATATCGAGGTTTATTTATTAATTTGAATAGATATTAAGTTTTATTATATTTACACTTACATACTA
ATAATAAATTCAACAAACAATTTATTTATGTTTATTTATTTATTAAAAAAAAACAAAAACTCAAAATTTCTTCTATAAAGTAACAAAACTT
TTATGAGGGACAGCCCCCCCCCAAAGCCCCCAGGGATGTAATTACGTCCCTCCCCCGCTAGGGGGCAGCAGCGAGCCGCCCG
GGGCTCCGCTCCGGTCCGGCGCTCCCCCCGCATCCCCGAGCCGGCAGCGTGCGGGGACAGCCCGGGCACGGGGAAGGTGG
CACGGGATCGCTTTCCTCTGAACGCTTCTCGCTGCTCTTTGAGCCTGCAGACACCTGGGGGGATACGGGGAAAAGGCCTAAAAA
CCGCACTTGTC**CGGAAACCCCGGGAATCTAACCCGGCTGAACGGATTTAGAGTCCGTTCGATCTACATGATCAGGTTTCC**GGT
GTTTCGTCCTTTCCACAAGATATATAAAGCCAAGAAATCGAAATACTTTCAAGTTACGGTAAGCATATGATAGTCCATTTTAAAACA
TAATTTTAAAACTGCAAACTACCCAAGAAATTATTACTTTCTACGTCACGTATTTTGTACTAATATCTTTGTGTTTACAGTCAAATTA
ATTCTAATTATCTCTCTAACAGCCTTGTATCGTATATGCAAATATGAAGGAATCATGGGAAATAGGCCCTCTTCCTGCCCAACCGG
TTGCGATCGCTCCGGTGCCCGTCAGTGGGCAGAGCGCACATCGCCCACAGTCCCCGAGAAGTTGGGGGGAGGGGTCGGCAAT
TGAACGGGTGCCTAGAGAAGGTGGCGCGGGGTAAACTGGGAAAGTGATGTCGTGTACTGGCTCCGCCTTTTTCCCGAGGGTGG
GGGAGAACCGTATATAAGTGCAGTAGTCGCCGTGAACGTTCTTTTTCGCAACGGGTTTGCCGCCAGAACACAGCTGAAGCTTCG
AGGGGCTCGCATCTCTCCTTCACGCGCCCGCCGCCCTACCTGAGGCCGCCATCCACGCCGGTTGAGTCGCGTTCTGCCGCCTC
CCGCCTGTGGTGCCTCCTGAACTGCGTCCGCCGTCTAGGTAAGTTTAAAGCTCAGGTCGAGACCGGGCCTTTGTCCGGCGCTC
CCTTGGAGCCTACCTAGACTCAGCCGGCTCTCCACGCTTTGCCTGACCCTGCTTGCTCAACTCTACGTCTTTGTTTCGTTTTCTG
TTCTGCGCCGTTACAGATCCAAGCTGTGACCGGCGCCTACTCTACAGATAGCGTTTAAACTTACGCTTGCCACC**ATGGCTAGCG**
ACTACAAGGACGACGACGACAAGGACAAGAAGCCCCTGAACACCCTGATCAGCGCCACAGGACTGTGGATGTCCAGAACCG
GCACCATCCACAAGATCAAGCACCACGAGGTGTCCCGGTCCAAAATCTACATCGAGATGGCCTGCGGCGATCACCTGGTCGT
CAACAACAGCAGAAGCAGCCGGACAGCCAGAGCCCTGCGGCACCACAAGTACAGAAAGACCTGCAAGCGGTGCAGAGTGT
CCGACGAGGACCTGAACAAGTTCCTGACCAAGGCCAACGAGGACCAGACCAGCGTGAAAGTGAAGGTGGTGTCCGCCCCCA
CCCGGACCAAGAAAGCCATGCCCAAGAGCGTGGCCAGAGCCCCCAAGCCCCTGGAAAACACCGAAGCCGCTCAGGCCCAG

CCCAGCGGCAGCAAGTTCAGCCCCGCCATCCCCGTGTCTACCCAGGAAAGCGTCAGCGTCCCCGCCAGCGTGTCCACCAGC
ATCTCTAGCATCTCAACCGGCGCCACAGCTTCTGCCCTGGTCAAGGGCAACACCAACCCCATCACCAGCATGTCTGCCCCTGT
GCAAGCCTCTGCCCCAGCCCTGACCAAGTCCCAGACCGACCGGCTGGAAGTGCTCCTGAACCCCAAGGACGAGATCAGCCT
GAACAGCGGCAAGCCCTTCCGGGAGCTGGAAAGCGAGCTGCTGAGCCGGCGGAAGAAGGACCTCCAGCAAATCTACGCCG
AGGAACGGGAGAACTACCTGGGCAAGCTGGAAAGAGAGATCACCCGGTTCTTCGTGGACCGGGGCTTCCTGGAAATCAAGA
GCCCCATCCTGATCCCCCTGGAGTACATCGAGCGGATGGGCATCGACAACGACACCGAGCTGAGCAAGCAGATTTTCCGGGT
GGACAAGAACTTCTGCCTGCGGCCCATGCTGGCCCCCAACCTGTACAACTACCTGCGGAAACTGGATCGCGCTCTGCCCGAC
CCCATCAAGATTTTCGAGATCGGCCCCTGCTACCGGAAAGAGAGCGACGGCAAAGAGCACCTGGAAGAGTTTACAATGCTGA
ACTTTTGCCAGATGGGCAGCGGCTGCACCAGAGAGAACCTGGAATCCATCATCACCGACTTTCTGAACCACCTGGGGATCGA
CTTCAAGATCGTGGGCGACAGCTGCATGGTGTACGGCGACACCCTGGACGTGATGCACGGCGACCTGGAACTGTCTAGCGC
CGTCGTGGGACCCATCCCTCTGGACCGGGAGTGGGGCATCGATAAGCCCTGGATCGGAGCCGGCTTCGGCCTGGAACGGCT
GCTGAAAGTCAAGCACGACTTTAAGAACATCAAGCGGGCTGCCAGAAGCGAGAGCTACTACAACGGCATCAGCACCAACCT
GTGATGATAAGGATCCACTAGTCCAGTGTGGTGGAATTGACATTGATTATTGACTAGTTATTAATAGTAATCAATTACGGGGTCAT
TAGTTCATAGCCCATATATGGAGTTCCGCGTTACATAACTTACGGTAAATGGCCCGCCTGGCTGACCGCCCAACGACCCCCGCC
CATTGACGTCAATAATGACGTATGTTCCCATAGTAACGCCAATAGGGACTTTCCATTGACGTCAATGGGTGGAGTATTTACGGTA
AACTGCCCACTTGGCAGTACATCAAGTGTATCATATGCCAAGTACGCCCCCTATTGACGTCAATGACGGTAAATGGCCCGCCTG
GCATTATGCCCAGTACATGACCTTATGGGACTTTCCTACTTGGCAGTACATCTACGTATTAGTCATCGCTATTACCATGGTCGAGG
TGAGCCCCACGTTCTGCTTCACTCTCCCCATCTCCCCCCCCTCCCCACCCCCAATTTTGTATTTATTTATTTTTTAATTATTTTGTG
CAGCGATGGGGGCGGGGGGGGGGGGGGCGCGCGCCAGGCGGGGCGGGGCGGGGCGAGGGGCGGGGCGGGGCGAGGCG
GAGAGGTGCGGCGGCAGCCAATCAGAGCGGCGCGCTCCGAAAGTTTCCTTTTATGGCGAGGCGGCGGCGGCGGCGGCCCTAT
AAAAAGCGAAGCGCGCGGCGGGCGGGAGTCGCTGCGTTGCCTTCGCCCCGTGCCCCGCTCCGCGCCGCCTCGCGCCGCCCG
CCCCGGCTCTGACTGACCGCGTTACTCCCACAGGTGAGCGGGCGGGACGGCCCTTCTCCTCCGGGCTGTAATTAGCGCTTGGT
TTAATGACGGCTCGTTTCTTTTCTGTGGCTGCGTGAAAGCCTTAAAGGGCTCCGGGAGGGCCCTTTGTGCGGGGGGGAGCGGC
TCGGGGGGTGCGTGCGTGTGTGTGTGCGTGGGGAGCGCCGCGTGCGGCCCGCGCTGCCCGGCGGCTGTGAGCGCTGCGGG
CGCGGCGCGGGGCTTTGTGCGCTCCGCGTGTGCGCGAGGGGAGCGCGGCCGGGGGCGGTGCCCCGCGGTGCGGGGGGGCT
GCGAGGGGAACAAAGGCTGCGTGCGGGGTGTGTGCGTGGGGGGGTGAGCAGGGGGTGTGGGCGCGGCGGTCGGGCTGTAA
CCCCCCCCTGCACCCCCCTCCCCGAGTTGCTGAGCACGGCCCGGCTTCGGGTGCGGGGCTCCGTACGGGGCGTGGCGCGGG
GCTCGCCGTGCCGGGCGGGGGGTGGCGGCAGGTGGGGGTGCCGGGCGGGGCGGGGCCGCCTCGGGCCGGGGAGGGCTCG
GGGGAGGGGCGCGGCGGCCCCCGGAGCGCCGGCGGCTGTCGAGGCGCGGCGAGCCGCAGCCATTGCCTTTTATGGTAATCG
TGCGAGAGGGCGCAGGGACTTCCTTTGTCCCAAATCTGTGCGGAGCCGAAATCTGGGAGGCGCCGCCGCACCCCCTCTAGCG
GGCGCGGGGCGAAGCGGTGCGGCGCCGGCAGGAAGGAAATGGGCGGGGAGGGCCTTCGTGCGTCGCCGCGCCGCCGTCCC
CTTCTCCCTCTCCAGCCTCGGGGCTGTCCGCGGGGGGACGGCTGCCTTCGGGGGGGACGGGGCAGGGCGGGGTTCGGCTTC
TGGCGTGTGACCGGCGGCTCTAGAGCCTCTGCTAACCATGTTCATGCCTTCTTCTTTTTCCTACAGCTCCTGGGCAACGTGCTG
GTTATTGTGCTGTCTCATCATTTTGGCAAAGAATTCGCCACCATGGTGAGCAAGGGCGAGGAGGATAACATGGCCATCATCAA
GGAGTTCATGCGCTTCAAGGTGCACATGGAGGGCTCCGTGAACGGCCACGAGTTCGAGATCGAGGGCGAGGGCGAGGGCC
GCCCCTACGAGGGCACCCAGACCGCCAAGCTGAAGGTGACCAAGGGTGGCCCCCTGCCCTTCGCCTGGGACATCCTGTCCC
CTCAGTTCATGTACGGCTCCAAGGCCTACGTGAAGCACCCCGCCGACATCCCCGACTACTTGAAGCTGTCCTTCCCCGAGGG
CTTCAAGTGGGAGCGCGTGATGAACTTCGAGGACGGCGGCGTGGTGACCGTGACCCAGGACTCCTCCCTGCAGGACGGCGA
GTTCATCTACAAGGTGAAGCTGCGCGGCACCAACTTCCCCTCCGACGGCCCCGTAATGCAGAAGAAGACCATGGGCTGGGA
GGCCTCCTCCGAGCGGATGTACCCCGAGGACGGCGCCCTGAAGGGCGAGATCAAGCAGAGGCTGAAGCTGAAGGACGGCG
GCCACTACGACGCTGAGGTCAAGACCACCTACAAGGCCAAGAAGCCCGTGCAGCTGCCCGGCGCCTACAACGTCAACATCA
AGTTGGACATCACCTCCCACAACGAGGACTACACCATCGTGGAACAGTACGAACGCGCCGAGGGCCGCCACTCCACCGGCG
GCATGGACGAGCTGTACGCGTCTGGAGGCGCCACCAATTTCAGCCTGCTGAAACAGGCTGGCGACGTGGAAGAGAACCCTG
GACCTGGACAAAAGTCGGTGAGCAAGGGCGAGGAGCTGTTCACCGGGGTGGTGCCCATCCTGGTCGAGCTGGACGGCGACG
TAAACGGCCACAAGTTCAGCGTGTCCGGCGAGGGCGAGGGCGATGCCACCTACGGCAAGCTGACCCTGAAGTTCATCTGCA
CCACCGGCAAGCTGCCCGTGCCCTGGCCCACCCTCGTGACCACCCTGACCTACGGCGTGCAGTGCTTCAGCCGCTACCCCGA
CCACATGAAGCAGCACGACTTCTTCAAGTCCGCCATGCCCGAAGGCTACGTCCAGGAGCGCACCATCTTCTTCAAGGACGAC
GGCAACTACAAGACCCGCGCCGAGGTGAAGTTCGAGGGCGACACCCTGGTGAACCGCATCGAGCTGAAGGGCATCGACTTC
AAGGAGGACGGCAACATCCTGGGGCACAAGCTGGAGTACAACTACAACAGCCACTAGGTCTATATCATGGCCGACAAGCAG
AAGAACGGCATCAAGGTGAACTTCAAGATCCGCCACAACATCGAGGACGGCAGCGTGCAGCTCGCCGACCACTACCAGCAG
AACACCCCCATCGGCGACGGCCCCGTGCTGCTGCCCGACAACCACTACCTGAGCACCCAGTCCGCCCTGAGCAAAGACCCC
AACGAGAAGCGCGATCACATGGTCCTGCTGGAGTTCGTGACCGCCGCCGGGATCACTCTCGGCATGGACGAGCTGTACAAGT
AAGAATTCACTCCTCAGGTGCAGGCTGCCTATCAGAAGGTGGTGGCTGGTGTGGCCAATGCCCTGGCTCACAAATACCACTGAG
ATCTTTTTCCCTCTGCCAAAAATTATGGGGACATCATGAAGCCCCTTGAGCATCTGACTTCTGGCTAATAAAGGAAATTTATTTTCA
TTGCAATAGTGTGTTGGAATTTTTTGTGTCTCTCACTCGGAAGGACATATGGGAGGGGTCGACAATCAACCTCTGGATTACAAAA
TTTGTGAAAGATTGACTGGTATTCTTAACTATGTTGCTCCTTTTACGCTATGTGGATACGCTGCTTTAATGCCTTTGTATCATGCGT
TAACTAAACTTGTTTATTGCAGCTTATAATGGTTACAAATAAAGCAATAGCATCACAAATTTCACAAATAAAGCATTTTTTTCACTGC
ATTCTAGTTGTGGTTTGTCCAAACTCATCAATGTATCTTATCATGTCTGGAATTGACTCAAATGATGTCAATTAGTCTATCAGAAGC
TATCTGGTCTCCCTTCCGGGGGACAAGACATCCCTGTTTAATATTTAAACAGCAGTGTTCCCAAACTGGGTTCTTATATCCCTTGC
TCTGGTCAACCAGGTTGCAGGGTTTCCTGTCCTCACAGGAACGAAGTCCCTAAAGAAACAGTGGCAGCCAGGTTTAGCCCCGGA
ATTGACTGGATTCCTTTTTTAGGGCCCATTGGTATGGCTTTTTCCCCGTATCCCCCCAGGTGTCTGCAGGCTCAAAGAGCAGCGA
GAAGCGTTCAGAGGAAAGCGATCCCGTGCCACCTTCCCCGTGCCCGGGCTGTCCCCGCACGCTGCCGGCTCGGGGATGCGGG
GGGAGCGCCGGACCGGAGCGGAGCCCCGGGCGGCTCGCTGCTGCCCCCTAGCGGGGGAGGGACGTAATTACATCCCTGGGG
GCTTTGGGGGGGGGCTGTCCCTGATATCTATAACAAGAAAATATATATATATAATAAGTTATCACGTAAGTAGAACATGAAATAACAA
TATAATTATCGTATGAGTTAAATCTTAAAAGTCACGTAAAAGATAATCATGCGTCATTTTGACTCACGCGGTCGTTATAGTTCAAAA
TCAGTGACACTTACCGCATTGACAAGCACGCCTCACGGGAGCTCCAAGCGGCGACTGAGATGTCCTAAATGCACAGCGACGGA
TTCGCGCTATTTAGAAAGAGAGAGCAATATTTCAAGAATGCATGCGTCAATTTTACGCAGACTATCTTTCTAGGGTTAATCTAGCT
GCATCAGGATCATATCGTCGGGTCTTTTTTCCGGCTCAGTCATCGCCCAAGCTGGCGCTATCTGGGCATCGGGGAGGAAGAAGC
CCGTGCCTTTTCCCGCGAGGTTGAAGCGGCATGGAAAGAGTTTGCCGAGGATGACTGCTGCTGCATTGACGTTGAGCGAAAAC
GCACGTTTACCATGATGATTCGGGAAGGTGTGGCCATGCACGCCTTTAACGGTGAACTGTTCGTTCAGGCCACCTGGGATACCA
GTTCGTCGCGGCTTTTCCGGACACAGTTCCGGATGGTCAGCCCGAAGCGCATCAGCAACCCGAACAATACCGGCGACAGCCGG
AACTGCCGTGCCGGTGTGCAGATTAATGACAGCGGTGCGGCGCTGGGATATTACGTCAGCGAGGACGGGTATCCTGGCTGGAT
GCCGCAGAAATGGACATGGATACCCCGTGAGTTACCCGGCGGGCGCGCTTGGCGTAATCATGGTCATAGCTGTTTCCTGTGTG
AAATTGTTATCCGCTCACAATTCCACACAACATACGAGCCGGAAGCATAAAGTGTAAAGCCTGGGGTGCCTAATGAGTGAGCTAA

CTCACATTAATTGCGTTGCGCTCACTGCCCGCTTTCCAGTCGGGAAACCTGTCGTGCCAGCTGCATTAATGAATCGGCCAACGC
GCGGGGAGAGGCGGTTTGCGTATTGGGCGCTCTTCCGCTTCCTCGCTCACTGACTCGCTGCGCTCGGTCGTTCGGCTGCGGC
GAGCGGTATCAGCTCACTCAAAGGCGGTAATACGGTTATCCACAGAATCAGGGGATAACGCAGGAAAGAACATGTGAGCAAAAG
GCCAGCAAAAGGCCAGGAACCGTAAAAAGGCCGCGTTGCTGGCGTTTTTCCATAGGCTCCGCCCCCCTGACGAGCATCACAAA
AATCGACGCTCAAGTCAGAGGTGGCGAAACCCGACAGGACTATAAAGATACCAGGCGTTTCCCCCTGGAAGCTCCCTCGTGCG
CTCTCCTGTTCCGACCCTGCCGCTTACCGGATACCTGTCCGCCTTTCTCCCTTCGGGAAGCGTGGCGCTTTCTCATAGCTCACG
CTGTAGGTATCTCAGTTCGGTGTAGGTCGTTCGCTCCAAGCTGGGCTGTGTGCACGAACCCCCCGTTCAGCCCGACCGCTGCG
CCTTATCCGGTAACTATCGTCTTGAGTCCAACCCGGTAAGACACGACTTATCGCCACTGGCAGCAGCCACTGGTAACAGGATTA
GCAGAGCGAGGTATGTAGGCGGTGCTACAGAGTTCTTGAAGTGGTGGCCTAACTACGGCTACACTAGAAGGACAGTATTTGGTA
TCTGCGCTCTGCTGAAGCCAGTTACCTTCGGAAAAAGAGTTGGTAGCTCTTGATCCGGCAAACAAACCACCGCTGGTAGCGGTG
GTTTTTTTGTTTGCAAGCAGCAGATTACGCGCAGAAAAAAAGGATCTCAAGAAGATCCTTTGATCTTTTCTACGGGGTCTGACGCT
CAGTGGAACGAAAACTCACGTTAAGGGATTTTGGTCATGAGATTATCAAAAAGGATCTTCACCTAGATCCTTTTAAATTAAAAATG
AAGTTTTAAATCAATCTAAAGTATATATGAGTAAACTTGGTCTGACAGTTACCAATGCTTAATCAGTGAGGCACCTATCTCAGCGAT
CTGTCTATTTCGTTCATCCATAGTTGCCTGACTCCCCGTCGTGTAGATAACTACGATACGGGAGGGCTTACCATCTGGCCCCAGT
GCTGCAATGATACCGCGAGACCCACGCTCACCGGCTCCAGATTTATCAGCAATAAACCAGCCAGCCGGAAGGGCCGAGCGCAG
AAGTGGTCCTGCAACTTTATCCGCCTCCATCCAGTCTATTAATTGTTGCCGGGAAGCTAGAGTAAGTAGTTCGCCAGTTAATAGTT
TGCGCAACGTTGTTGCCATTGCTACAGGCATCGTGGTGTCACGCTCGTCGTTTGGTATGGCTTCATTCAGCTCCGGTTCCCAAC
GATCAAGGCGAGTTACATGATCCCCCATGTTGTGCAAAAAAGCGGTTAGCTCCTTCGGTCCTCCGATCGTTGTCAGAAGTAAGTT
GGCCGCAGTGTTATCACTCATGGTTATGGCAGCACTGCATAATTCTCTTACTGTCATGCCATCCGTAAGATGCTTTTCTGTGACTG
GTGAGTACTCAACCAAGTCATTCTGAGAATAGTGTATGCGGCGACCGAGTTGCTCTTGCCCGGCGTCAATACGGGATAATACCG
CGCCACATAGCAGAACTTTAAAAGTGCTCATCATTGGAAAACGTTCTTCGGGGCGAAAACTCTCAAGGATCTTACCGCTGTTGAG
ATCCAGTTCGATGTAACCCACTCGTGCACCCAACTGATCTTCAGCATCTTTTACTTTCACCAGCGTTTCTGGGTGAGCAAAAACA
GGAAGGCAAAATGCCGCAAAAAAGGGAATAAGGGCGACACGGAAATGTTGAATACTCAT

2.2.6 Vector 59: Pyl $tRNA_{UCUA}$ PylRS mCherry-P2A-eGFP(150TAGA)

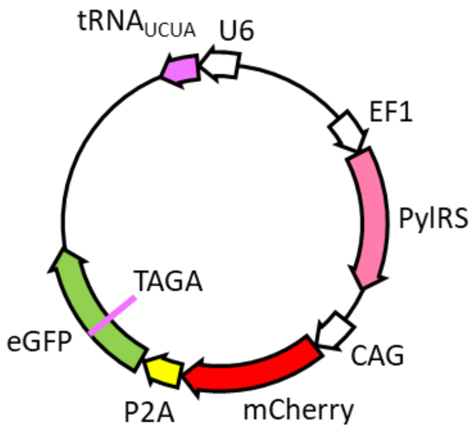

ACTCTTCCTTTTTCAATATTATTGAAGCATTTATCAGGGTTATTGTCTCATGAGCGGATACATATTTGAATGTATTTAGAAAAATAAA
CAAATAGGGGTTCCGCGCACATTTCCCCGAAAAGTGCCACCTAAATTGTAAGCGTTAATATTTTGTTAAAATTCGCGTTAAATTTTT
GTTAAATCAGCTCATTTTTTAACCAATAGGCCGAAATCGGCAAAATCCCTTATAAATCAAAAGAATAGACCGAGATAGGGTTGAGT
GTTGTTCCAGTTTGGAACAAGAGTCCACTATTAAAGAACGTGGACTCCAACGTCAAAGGGCGAAAAACCGTCTATCAGGGCGAT
GGCCCACTACGTGAACCATCACCCTAATCAAGTTTTTTGGGGTCGAGGTGCCGTAAAGCACTAAATCGGAACCCTAAAGGGAGC
CCCCGATTTAGAGCTTGACGGGGAAAGCCGGCGAACGTGGCGAGAAAGGAAGGGAAGAAAGCGAAAGGAGCGGGCGCTAGGG
CGCTGGCAAGTGTAGCGGTCACGCTGCGCGTAACCACCACACCCGCCGCGCTTAATGCGCCGCTACAGGGCGCGTCCCATTC
GCCATTCAGGCTGCGCAACTGTTGGGAAGGGCGATCGGTGCGGGCCTCTTCGCTATTACGCCAGCTGGCGAAAGGGGGATGT
GCTGCAAGGCGATTAAGTTGGGTAACGCCAGGGTTTTCCCAGTCACGACGTTGTAAAACGACGGCCAGTGAGCGCGCCTCGTT
CATTCACGTTTTTGAACCCGTGGAGGACGGGCAGACTCGCGGTGCAAATGTGTTTTACAGCGTGATGGAGCAGATGAAGATGCT
CGACACGCTGCAGAACACGCAGCTAGATTAACCCTAGAAAGATAATCATATTGTGACGTACGTTAAAGATAATCATGCGTAAAATT
GACGCATGTGTTTTATCGGTCTGTATATCGAGGTTTATTTATTAATTTGAATAGATATTAAGTTTTATTATATTTACACTTACATACTA
ATAATAAATTCAACAAACAATTTATTTATGTTTATTTATTTATTAAAAAAAAACAAAAACTCAAAATTTCTTCTATAAAGTAACAAAACTT
TTATGAGGGACAGCCCCCCCCCAAAGCCCCCAGGGATGTAATTACGTCCCTCCCCCGCTAGGGGGCAGCAGCGAGCCGCCCG
GGGCTCCGCTCCGGTCCGGCGCTCCCCCCGCATCCCCGAGCCGGCAGCGTGCGGGGACAGCCCGGGCACGGGGAAGGTGG
CACGGGATCGCTTTCCTCTGAACGCTTCTCGCTGCTCTTTGAGCCTGCAGACACCTGGGGGGATACGGGGAAAAGGCCTAAAAA
CCGCACTTGTC**CGGAAACCCCGGGAATCTAACCCGGCTGAACGGATTTAGAAGTCCATTCGATCTACATGATCAGGTTTCC**GG
TGTTTCGTCCTTTCCACAAGATATATAAAGCCAAGAAATCGAAATACTTTCAAGTTACGGTAAGCATATGATAGTCCATTTTAAAAC
ATAATTTTAAAACTGCAAACTACCCAAGAAATTATTACTTTCTACGTCACGTATTTTGTACTAATATCTTTGTGTTTACAGTCAAATT
AATTCTAATTATCTCTCTAACAGCCTTGTATCGTATATGCAAATATGAAGGAATCATGGGAAATAGGCCCTCTTCCTGCCCAACCG
GTTGCGATCGCTCCGGTGCCCGTCAGTGGGCAGAGCGCACATCGCCCACAGTCCCCGAGAAGTTGGGGGGAGGGGTCGGCAA
TTGAACGGGTGCCTAGAGAAGGTGGCGCGGGGTAAACTGGGAAAGTGATGTCGTGTACTGGCTCCGCCTTTTTCCCGAGGGTG
GGGGAGAACCGTATATAAGTGCAGTAGTCGCCGTGAACGTTCTTTTTCGCAACGGGTTTGCCGCCAGAACACAGCTGAAGCTTC
GAGGGGCTCGCATCTCTCCTTCACGCGCCCGCCGCCCTACCTGAGGCCGCCATCCACGCCGGTTGAGTCGCGTTCTGCCGCCT
CCCGCCTGTGGTGCCTCCTGAACTGCGTCCGCCGTCTAGGTAAGTTTAAAGCTCAGGTCGAGACCGGGCCTTTGTCCGGCGCT
CCCTTGGAGCCTACCTAGACTCAGCCGGCTCTCCACGCTTTGCCTGACCCTGCTTGCTCAACTCTACGTCTTTGTTTCGTTTTCT
GTTCTGCGCCGTTACAGATCCAAGCTGTGACCGGCGCCTACTCTACAGATAGCGTTTAAACTTACGCTTGCCACC**ATGGCTAGC**
GACTACAAGGACGACGACGACAAGGACAAGAAGCCCCTGAACACCCTGATCAGCGCCACAGGACTGTGGATGTCCAGAACC
GGCACCATCCACAAGATCAAGCACCACGAGGTGTCCCGGTCCAAAATCTACATCGAGATGGCCTGCGGCGATCACCTGGTCG
TCAACAACAGCAGAAGCAGCCGGACAGCCAGAGCCCTGCGGCACCACAAGTACAGAAAGACCTGCAAGCGGTGCAGAGTGT
CCGACGAGGACCTGAACAAGTTCCTGACCAAGGCCAACGAGGACCAGACCAGCGTGAAAGTGAAGGTGGTGTCCGCCCCCA

CCCGGACCAAGAAAGCCATGCCCAAGAGCGTGGCCAGAGCCCCCAAGCCCCTGGAAAACACCGAAGCCGCTCAGGCCCAG
CCCAGCGGCAGCAAGTTCAGCCCCGCCATCCCCGTGTCTACCCAGGAAAGCGTCAGCGTCCCCGCCAGCGTGTCCACCAGC
ATCTCTAGCATCTCAACCGGCGCCACAGCTTCTGCCCTGGTCAAGGGCAACACCAACCCCATCACCAGCATGTCTGCCCCTGT
GCAAGCCTCTGCCCCAGCCCTGACCAAGTCCCAGACCGACCGGCTGGAAGTGCTCCTGAACCCCAAGGACGAGATCAGCCT
GAACAGCGGCAAGCCCTTCCGGGAGCTGGAAAGCGAGCTGCTGAGCCGGCGGAAGAAGGACCTCCAGCAAATCTACGCCG
AGGAACGGGAGAACTACCTGGGCAAGCTGGAAAGAGAGATCACCCGGTTCTTCGTGGACCGGGGCTTCCTGGAAATCAAGA
GCCCCATCCTGATCCCCCTGGAGTACATCGAGCGGATGGGCATCGACAACGACACCGAGCTGAGCAAGCAGATTTTCCGGGT
GGACAAGAACTTCTGCCTGCGGCCCATGCTGGCCCCCAACCTGTACAACTACCTGCGGAAACTGGATCGCGCTCTGCCCGAC
CCCATCAAGATTTTCGAGATCGGCCCCTGCTACCGGAAAGAGAGCGACGGCAAAGAGCACCTGGAAGAGTTTACAATGCTGA
ACTTTTGCCAGATGGGCAGCGGCTGCACCAGAGAGAACCTGGAATCCATCATCACCGACTTTCTGAACCACCTGGGGATCGA
CTTCAAGATCGTGGGCGACAGCTGCATGGTGTACGGCGACACCCTGGACGTGATGCACGGCGACCTGGAACTGTCTAGCGC
CGTCGTGGGACCCATCCCTCTGGACCGGGAGTGGGGCATCGATAAGCCCTGGATCGGAGCCGGCTTCGGCCTGGAACGGCT
GCTGAAAGTCAAGCACGACTTTAAGAACATCAAGCGGGCTGCCAGAAGCGAGAGCTACTACAACGGCATCAGCACCAACCT
GTGATGATAAGGATCCACTAGTCCAGTGTGGTGGAATTGACATTGATTATTGACTAGTTATTAATAGTAATCAATTACGGGGTCAT
TAGTTCATAGCCCATATATGGAGTTCCGCGTTACATAACTTACGGTAAATGGCCCGCCTGGCTGACCGCCCAACGACCCCCGCC
CATTGACGTCAATAATGACGTATGTTCCCATAGTAACGCCAATAGGGACTTTCCATTGACGTCAATGGGTGGAGTATTTACGGTA
AACTGCCCACTTGGCAGTACATCAAGTGTATCATATGCCAAGTACGCCCCCTATTGACGTCAATGACGGTAAATGGCCCGCCTG
GCATTATGCCCAGTACATGACCTTATGGGACTTTCCTACTTGGCAGTACATCTACGTATTAGTCATCGCTATTACCATGGTCGAGG
TGAGCCCCACGTTCTGCTTCACTCTCCCCATCTCCCCCCCCTCCCCACCCCCAATTTTGTATTTATTTATTTTTTAATTATTTTGTG
CAGCGATGGGGGCGGGGGGGGGGGGGGGGCGCGCGCCAGGCGGGGCGGGGCGGGGCGAGGGGCGGGGCGGGGCGAGGCG
GAGAGGTGCGGCGGCAGCCAATCAGAGCGGCGCGCTCCGAAAGTTTCCTTTTATGGCGAGGCGGCGGCGGCGGCGGCCCTAT
AAAAAGCGAAGCGCGCGGCGGGCGGGAGTCGCTGCGTTGCCTTCGCCCCGTGCCCCGCTCCGCGCCGCCTCGCGCCGCCCG
CCCCGGCTCTGACTGACCGCGTTACTCCCACAGGTGAGCGGGCGGGACGGCCCTTCTCCTCCGGGCTGTAATTAGCGCTTGGT
TTAATGACGGCTCGTTTCTTTTCTGTGGCTGCGTGAAAGCCTTAAAGGGCTCCGGGAGGGCCCTTTGTGCGGGGGGGAGCGGC
TCGGGGGGTGCGTGCGTGTGTGTGTGCGTGGGGAGCGCCGCGTGCGGCCCGCGCTGCCCGGCGGCTGTGAGCGCTGCGGG
CGCGGCGCGGGGCTTTGTGCGCTCCGCGTGTGCGCGAGGGGAGCGCGGCCGGGGGCGGTGCCCCGCGGTGCGGGGGGGCT
GCGAGGGGAACAAAGGCTGCGTGCGGGGTGTGTGCGTGGGGGGGTGAGCAGGGGGTGTGGGCGCGGCGGTCGGGCTGTAA
CCCCCCCCTGCACCCCCCTCCCCGAGTTGCTGAGCACGGCCCGGCTTCGGGTGCGGGGCTCCGTACGGGGCGTGGCGCGGG
GCTCGCCGTGCCGGGCGGGGGGTGGCGGCAGGTGGGGGTGCCGGGCGGGGCGGGGCCGCCTCGGGCCGGGGAGGGCTCG
GGGGAGGGGCGCGGCGGCCCCCGGAGCGCCGGCGGCTGTCGAGGCGCGGCGAGCCGCAGCCATTGCCTTTTATGGTAATCG
TGCGAGAGGGCGCAGGGACTTCCTTTGTCCCAAATCTGTGCGGAGCCGAAATCTGGGAGGCGCCGCCGCACCCCCTCTAGCG
GGCGCGGGGCGAAGCGGTGCGGCGCCGGCAGGAAGGAAATGGGCGGGGAGGGCCTTCGTGCGTCGCCGCGCCGCCGTCCC
CTTCTCCCTCTCCAGCCTCGGGGCTGTCCGCGGGGGGACGGCTGCCTTCGGGGGGGACGGGGCAGGGCGGGGTTCGGCTTC
TGGCGTGTGACCGGCGGCTCTAGAGCCTCTGCTAACCATGTTCATGCCTTCTTCTTTTTCCTACAGCTCCTGGGCAACGTGCTG
GTTATTGTGCTGTCTCATCATTTTGGCAAAGAATTCGCCACCATGGTGAGCAAGGGCGAGGAGGATAACATGGCCATCATCAA
GGAGTTCATGCGCTTCAAGGTGCACATGGAGGGCTCCGTGAACGGCCACGAGTTCGAGATCGAGGGCGAGGGCGAGGGCC
GCCCCTACGAGGGCACCCAGACCGCCAAGCTGAAGGTGACCAAGGGTGGCCCCCTGCCCTTCGCCTGGGACATCCTGTCCC
CTCAGTTCATGTACGGCTCCAAGGCCTACGTGAAGCACCCCGCCGACATCCCCGACTACTTGAAGCTGTCCTTCCCCGAGGG
CTTCAAGTGGGAGCGCGTGATGAACTTCGAGGACGGCGGCGTGGTGACCGTGACCCAGGACTCCTCCCTGCAGGACGGCGA
GTTCATCTACAAGGTGAAGCTGCGCGGCACCAACTTCCCCTCCGACGGCCCCGTAATGCAGAAGAAGACCATGGGCTGGGA
GGCCTCCTCCGAGCGGATGTACCCCGAGGACGGCGCCCTGAAGGGCGAGATCAAGCAGAGGCTGAAGCTGAAGGACGGCG
GCCACTACGACGCTGAGGTCAAGACCACCTACAAGGCCAAGAAGCCCGTGCAGCTGCCCGGCGCCTACAACGTCAACATCA
AGTTGGACATCACCTCCCACAACGAGGACTACACCATCGTGGAACAGTACGAACGCGCCGAGGGCCGCCACTCCACCGGCG
GCATGGACGAGCTGTACGCGTCTGGAGGCGCCACCAATTTCAGCCTGCTGAAACAGGCTGGCGACGTGGAAGAGAACCCTG
GACCTGGACAAAAGTCGGTGAGCAAGGGCGAGGAGCTGTTCACCGGGGTGGTGCCCATCCTGGTCGAGCTGGACGGCGACG
TAAACGGCCACAAGTTCAGCGTGTCCGGCGAGGGCGAGGGCGATGCCACCTACGGCAAGCTGACCCTGAAGTTCATCTGCA
CCACCGGCAAGCTGCCCGTGCCCTGGCCCACCCTCGTGACCACCCTGACCTACGGCGTGCAGTGCTTCAGCCGCTACCCCGA
CCACATGAAGCAGCACGACTTCTTCAAGTCCGCCATGCCCGAAGGCTACGTCCAGGAGCGCACCATCTTCTTCAAGGACGAC
GGCAACTACAAGACCCGCGCCGAGGTGAAGTTCGAGGGCGACACCCTGGTGAACCGCATCGAGCTGAAGGGCATCGACTTC
AAGGAGGACGGCAACATCCTGGGGCACAAGCTGGAGTACAACTACAACAGCCACTAGAGTCTATATCATGGCCGACAAGCA
GAAGAACGGCATCAAGGTGAACTTCAAGATCCGCCACAACATCGAGGACGGCAGCGTGCAGCTCGCCGACCACTACCAGCA
GAACACCCCCATCGGCGACGGCCCCGTGCTGCTGCCCGACAACCACTACCTGAGCACCCAGTCCGCCCTGAGCAAAGACCC
CAACGAGAAGCGCGATCACATGGTCCTGCTGGAGTTCGTGACCGCCGCCGGGATCACTCTCGGCATGGACGAGCTGTACAA
GTAAGAATTCACTCCTCAGGTGCAGGCTGCCTATCAGAAGGTGGTGGCTGGTGTGGCCAATGCCCTGGCTCACAAATACCACTG
AGATCTTTTTCCCTCTGCCAAAAATTATGGGGACATCATGAAGCCCCTTGAGCATCTGACTTCTGGCTAATAAAGGAAATTTATTT
TCATTGCAATAGTGTGTTGGAATTTTTTGTGTCTCTCACTCGGAAGGACATATGGGAGGGGTCGACAATCAACCTCTGGATTACA
AAATTTGTGAAAGATTGACTGGTATTCTTAACTATGTTGCTCCTTTTACGCTATGTGGATACGCTGCTTTAATGCCTTTGTATCATG
CGTTAACTAAACTTGTTTATTGCAGCTTATAATGGTTACAAATAAAGCAATAGCATCACAAATTTCACAAATAAAGCATTTTTTTCAC
TGCATTCTAGTTGTGGTTTGTCCAAACTCATCAATGTATCTTATCATGTCTGGAATTGACTCAAATGATGTCAATTAGTCTATCAGA
AGCTATCTGGTCTCCCTTCCGGGGGACAAGACATCCCTGTTTAATATTTAAACAGCAGTGTTCCCAAACTGGGTTCTTATATCCCT
TGCTCTGGTCAACCAGGTTGCAGGGTTTCCTGTCCTCACAGGAACGAAGTCCCTAAAGAAACAGTGGCAGCCAGGTTTAGCCCC
GGAATTGACTGGATTCCTTTTTTAGGGCCCATTGGTATGGCTTTTTCCCCGTATCCCCCCAGGTGTCTGCAGGCTCAAAGAGCAG
CGAGAAGCGTTCAGAGGAAAGCGATCCCGTGCCACCTTCCCCGTGCCCGGGCTGTCCCCGCACGCTGCCGGCTCGGGGATGC
GGGGGGAGCGCCGGACCGGAGCGGAGCCCCGGGCGGCTCGCTGCTGCCCCCTAGCGGGGGAGGGACGTAATTACATCCCTG
GGGGCTTTGGGGGGGGGCTGTCCCTGATATCTATAACAAGAAAATATATATATATAATAAGTTATCACGTAAGTAGAACATGAAATAA
CAATATAATTATCGTATGAGTTAAATCTTAAAAGTCACGTAAAAGATAATCATGCGTCATTTTGACTCACGCGGTCGTTATAGTTCA
AAATCAGTGACACTTACCGCATTGACAAGCACGCCTCACGGGAGCTCCAAGCGGCGACTGAGATGTCCTAAATGCACAGCGACG
GATTCGCGCTATTTAGAAAGAGAGAGCAATATTTCAAGAATGCATGCGTCAATTTTACGCAGACTATCTTTCTAGGGTTAATCTAG
CTGCATCAGGATCATATCGTCGGGTCTTTTTTCCGGCTCAGTCATCGCCCAAGCTGGCGCTATCTGGGCATCGGGGAGGAAGAA
GCCCGTGCCTTTTCCCGCGAGGTTGAAGCGGCATGGAAAGAGTTTGCCGAGGATGACTGCTGCTGCATTGACGTTGAGCGAAA
ACGCACGTTTACCATGATGATTCGGGAAGGTGTGGCCATGCACGCCTTTAACGGTGAACTGTTCGTTCAGGCCACCTGGGATAC
CAGTTCGTCGCGGCTTTTCCGGACACAGTTCCGGATGGTCAGCCCGAAGCGCATCAGCAACCCGAACAATACCGGCGACAGCC
GGAACTGCCGTGCCGGTGTGCAGATTAATGACAGCGGTGCGGCGCTGGGATATTACGTCAGCGAGGACGGGTATCCTGGCTG
GATGCCGCAGAAATGGACATGGATACCCCGTGAGTTACCCGGCGGGCGCGCTTGGCGTAATCATGGTCATAGCTGTTTCCTGT

GTGAAATTGTTATCCGCTCACAATTCCACACAACATACGAGCCGGAAGCATAAAGTGTAAAGCCTGGGGTGCCTAATGAGTGAG
CTAACTCACATTAATTGCGTTGCGCTCACTGCCCGCTTTCCAGTCGGGAAACCTGTCGTGCCAGCTGCATTAATGAATCGGCCAA
CGCGCGGGGAGAGGCGGTTTGCGTATTGGGCGCTCTTCCGCTTCCTCGCTCACTGACTCGCTGCGCTCGGTCGTTCGGCTGCG
GCGAGCGGTATCAGCTCACTCAAAGGCGGTAATACGGTTATCCACAGAATCAGGGGATAACGCAGGAAAGAACATGTGAGCAAA
AGGCCAGCAAAAGGCCAGGAACCGTAAAAAGGCCGCGTTGCTGGCGTTTTTCCATAGGCTCCGCCCCCCTGACGAGCATCACA
AAAATCGACGCTCAAGTCAGAGGTGGCGAAACCCGACAGGACTATAAAGATACCAGGCGTTTCCCCCTGGAAGCTCCCTCGTGC
GCTCTCCTGTTCCGACCCTGCCGCTTACCGGATACCTGTCCGCCTTTCTCCCTTCGGGAAGCGTGGCGCTTTCTCATAGCTCAC
GCTGTAGGTATCTCAGTTCGGTGTAGGTCGTTCGCTCCAAGCTGGGCTGTGTGCACGAACCCCCCGTTCAGCCCGACCGCTGC
GCCTTATCCGGTAACTATCGTCTTGAGTCCAACCCGGTAAGACACGACTTATCGCCACTGGCAGCAGCCACTGGTAACAGGATT
AGCAGAGCGAGGTATGTAGGCGGTGCTACAGAGTTCTTGAAGTGGTGGCCTAACTACGGCTACACTAGAAGGACAGTATTTGGT
ATCTGCGCTCTGCTGAAGCCAGTTACCTTCGGAAAAAGAGTTGGTAGCTCTTGATCCGGCAAACAAACCACCGCTGGTAGCGGT
GGTTTTTTTGTTTGCAAGCAGCAGATTACGCGCAGAAAAAAAGGATCTCAAGAAGATCCTTTGATCTTTTCTACGGGGTCTGACG
CTCAGTGGAACGAAAACTCACGTTAAGGGATTTTGGTCATGAGATTATCAAAAAGGATCTTCACCTAGATCCTTTTAAATTAAAAAT
GAAGTTTTAAATCAATCTAAAGTATATATGAGTAAACTTGGTCTGACAGTTACCAATGCTTAATCAGTGAGGCACCTATCTCAGCG
ATCTGTCTATTTCGTTCATCCATAGTTGCCTGACTCCCCGTCGTGTAGATAACTACGATACGGGAGGGCTTACCATCTGGCCCCA
GTGCTGCAATGATACCGCGAGACCCACGCTCACCGGCTCCAGATTTATCAGCAATAAACCAGCCAGCCGGAAGGGCCGAGCGC
AGAAGTGGTCCTGCAACTTTATCCGCCTCCATCCAGTCTATTAATTGTTGCCGGGAAGCTAGAGTAAGTAGTTCGCCAGTTAATA
GTTTGCGCAACGTTGTTGCCATTGCTACAGGCATCGTGGTGTCACGCTCGTCGTTTGGTATGGCTTCATTCAGCTCCGGTTCCCA
ACGATCAAGGCGAGTTACATGATCCCCCATGTTGTGCAAAAAAGCGGTTAGCTCCTTCGGTCCTCCGATCGTTGTCAGAAGTAAG
TTGGCCGCAGTGTTATCACTCATGGTTATGGCAGCACTGCATAATTCTCTTACTGTCATGCCATCCGTAAGATGCTTTTCTGTGAC
TGGTGAGTACTCAACCAAGTCATTCTGAGAATAGTGTATGCGGCGACCGAGTTGCTCTTGCCCGGCGTCAATACGGGATAATAC
CGCGCCACATAGCAGAACTTTAAAAGTGCTCATCATTGGAAAACGTTCTTCGGGGCGAAAACTCTCAAGGATCTTACCGCTGTTG
AGATCCAGTTCGATGTAACCCACTCGTGCACCCAACTGATCTTCAGCATCTTTTACTTTCACCAGCGTTTCTGGGTGAGCAAAAA
CAGGAAGGCAAAATGCCGCAAAAAAGGGAATAAGGGCGACACGGAAATGTTGAATACTCAT

2.2.7 Vector 60: Pyl tRNA$_{UCUA(Ev1)}$ PylRS mCherry-P2A-eGFP(150TAGA)

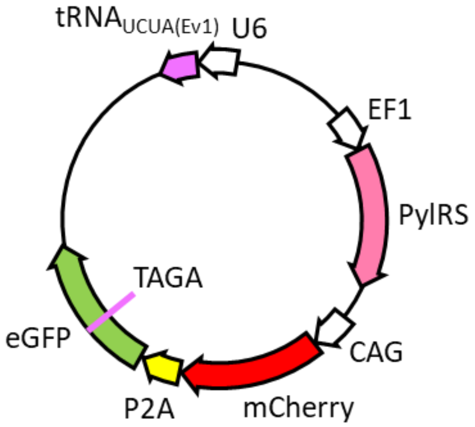

ACTCTTCCTTTTTCAATATTATTGAAGCATTTATCAGGGTTATTGTCTCATGAGCGGATACATATTTGAATGTATTTAGAAAAATAAA
CAAATAGGGGTTCCGCGCACATTTCCCCGAAAAGTGCCACCTAAATTGTAAGCGTTAATATTTTGTTAAAATTCGCGTTAAATTTTT
GTTAAATCAGCTCATTTTTTAACCAATAGGCCGAAATCGGCAAAATCCCTTATAAATCAAAAGAATAGACCGAGATAGGGTTGAGT
GTTGTTCCAGTTTGGAACAAGAGTCCACTATTAAAGAACGTGGACTCCAACGTCAAAGGGCGAAAAACCGTCTATCAGGGCGAT
GGCCCACTACGTGAACCATCACCCTAATCAAGTTTTTTGGGGTCGAGGTGCCGTAAAGCACTAAATCGGAACCCTAAAGGGAGC
CCCCGATTTAGAGCTTGACGGGGAAAGCCGGCGAACGTGGCGAGAAAGGAAGGGAAGAAAGCGAAAGGAGCGGGCGCTAGGG
CGCTGGCAAGTGTAGCGGTCACGCTGCGCGTAACCACCACACCCGCCGCGCTTAATGCGCCGCTACAGGGCGCGTCCCATTC
GCCATTCAGGCTGCGCAACTGTTGGGAAGGGCGATCGGTGCGGGCCTCTTCGCTATTACGCCAGCTGGCGAAAGGGGGATGT
GCTGCAAGGCGATTAAGTTGGGTAACGCCAGGGTTTTCCCAGTCACGACGTTGTAAAACGACGGCCAGTGAGCGCGCCTCGTT
CATTCACGTTTTTGAACCCGTGGAGGACGGGCAGACTCGCGGTGCAAATGTGTTTTACAGCGTGATGGAGCAGATGAAGATGCT
CGACACGCTGCAGAACACGCAGCTAGATTAACCCTAGAAAGATAATCATATTGTGACGTACGTTAAAGATAATCATGCGTAAAATT
GACGCATGTGTTTTATCGGTCTGTATATCGAGGTTTATTTATTAATTTGAATAGATATTAAGTTTTATTATATTTACACTTACATACTA
ATAATAAATTCAACAAACAATTTATTTATGTTTATTTATTTATTAAAAAAAAACAAAAACTCAAAATTTCTTCTATAAAGTAACAAAACTT
TTATGAGGGACAGCCCCCCCCCAAAGCCCCCAGGGATGTAATTACGTCCCTCCCCCGCTAGGGGGCAGCAGCGAGCCGCCCG
GGGCTCCGCTCCGGTCCGGCGCTCCCCCCGCATCCCCGAGCCGGCAGCGTGCGGGGACAGCCCGGGCACGGGGAAGGTGG
CACGGGATCGCTTTCCTCTGAACGCTTCTCGCTGCTCTTTGAGCCTGCAGACACCTGGGGGGATACGGGGAAAAGGCCTAAAAA
CCGCACTTGTC**CGGAAACCCCGGGAATCTAACCCGGCTGAACGAGATTAGAAGCCCATTCGATCTACATGATCAGGTTTCC**G
GTGTTTCGTCCTTTCCACAAGATATATAAAGCCAAGAAATCGAAATACTTTCAAGTTACGGTAAGCATATGATAGTCCATTTTAAAA
CATAATTTTAAAACTGCAAACTACCCAAGAAATTATTACTTTCTACGTCACGTATTTTGTACTAATATCTTTGTGTTTACAGTCAAAT
TAATTCTAATTATCTCTCTAACAGCCTTGTATCGTATATGCAAATATGAAGGAATCATGGGAAATAGGCCCTCTTCCTGCCCAACC
GGTTGCGATCGCTCCGGTGCCCGTCAGTGGGCAGAGCGCACATCGCCCACAGTCCCCGAGAAGTTGGGGGGAGGGGTCGGCA
ATTGAACGGGTGCCTAGAGAAGGTGGCGCGGGGTAAACTGGGAAAGTGATGTCGTGTACTGGCTCCGCCTTTTTCCCGAGGGT
GGGGGAGAACCGTATATAAGTGCAGTAGTCGCCGTGAACGTTCTTTTTCGCAACGGGTTTGCCGCCAGAACACAGCTGAAGCTT
CGAGGGGCTCGCATCTCTCCTTCACGCGCCCGCCGCCCTACCTGAGGCCGCCATCCACGCCGGTTGAGTCGCGTTCTGCCGC
CTCCCGCCTGTGGTGCCTCCTGAACTGCGTCCGCCGTCTAGGTAAGTTTAAAGCTCAGGTCGAGACCGGGCCTTTGTCCGGCG
CTCCCTTGGAGCCTACCTAGACTCAGCCGGCTCTCCACGCTTTGCCTGACCCTGCTTGCTCAACTCTACGTCTTTGTTTCGTTTT
CTGTTCTGCGCCGTTACAGATCCAAGCTGTGACCGGCGCCTACTCTACAGATAGCGTTTAAACTTACGCTTGCCACC**ATGGCTA**
GCGACTACAAGGACGACGACGACAAGGACAAGAAGCCCCTGAACACCCTGATCAGCGCCACAGGACTGTGGATGTCCAGAA
CCGGCACCATCCACAAGATCAAGCACCACGAGGTGTCCCGGTCCAAAATCTACATCGAGATGGCCTGCGGCGATCACCTGGT
CGTCAACAACAGCAGAAGCAGCCGGACAGCCAGAGCCCTGCGGCACCACAAGTACAGAAAGACCTGCAAGCGGTGCAGAG

TGTCCGACGAGGACCTGAACAAGTTCCTGACCAAGGCCAACGAGGACCAGACCAGCGTGAAAGTGAAGGTGGTGTCCGCCC
CCACCCGGACCAAGAAAGCCATGCCCAAGAGCGTGGCCAGAGCCCCCAAGCCCCTGGAAAACACCGAAGCCGCTCAGGCC
CAGCCCAGCGGCAGCAAGTTCAGCCCCGCCATCCCCGTGTCTACCCAGGAAAGCGTCAGCGTCCCCGCCAGCGTGTCCACC
AGCATCTCTAGCATCTCAACCGGCGCCACAGCTTCTGCCCTGGTCAAGGGCAACACCAACCCCATCACCAGCATGTCTGCCC
CTGTGCAAGCCTCTGCCCCAGCCCTGACCAAGTCCCAGACCGACCGGCTGGAAGTGCTCCTGAACCCCAAGGACGAGATCA
GCCTGAACAGCGGCAAGCCCTTCCGGGAGCTGGAAAGCGAGCTGCTGAGCCGGCGGAAGAAGGACCTCCAGCAAATCTACG
CCGAGGAACGGGAGAACTACCTGGGCAAGCTGGAAAGAGAGATCACCCGGTTCTTCGTGGACCGGGGCTTCCTGGAAATCA
AGAGCCCCATCCTGATCCCCCTGGAGTACATCGAGCGGATGGGCATCGACAACGACACCGAGCTGAGCAAGCAGATTTTCCG
GGTGGACAAGAACTTCTGCCTGCGGCCCATGCTGGCCCCCAACCTGTACAACTACCTGCGGAAACTGGATCGCGCTCTGCCC
GACCCCATCAAGATTTTCGAGATCGGCCCCTGCTACCGGAAAGAGAGCGACGGCAAAGAGCACCTGGAAGAGTTTACAATGC
TGAACTTTTGCCAGATGGGCAGCGGCTGCACCAGAGAGAACCTGGAATCCATCATCACCGACTTTCTGAACCACCTGGGGAT
CGACTTCAAGATCGTGGGCGACAGCTGCATGGTGTACGGCGACACCCTGGACGTGATGCACGGCGACCTGGAACTGTCTAG
CGCCGTCGTGGGACCCATCCCTCTGGACCGGGAGTGGGGCATCGATAAGCCCTGGATCGGAGCCGGCTTCGGCCTGGAACG
GCTGCTGAAAGTCAAGCACGACTTTAAGAACATCAAGCGGGCTGCCAGAAGCGAGAGCTACTACAACGGCATCAGCACCAA
CCTGTGATGATAAGGATCCACTAGTCCAGTGTGGTGGAATTGACATTGATTATTGACTAGTTATTAATAGTAATCAATTACGGGGT
CATTAGTTCATAGCCCATATATGGAGTTCCGCGTTACATAACTTACGGTAAATGGCCCGCCTGGCTGACCGCCCAACGACCCCC
GCCCATTGACGTCAATAATGACGTATGTTCCCATAGTAACGCCAATAGGGACTTTCCATTGACGTCAATGGGTGGAGTATTTACG
GTAAACTGCCCACTTGGCAGTACATCAAGTGTATCATATGCCAAGTACGCCCCCTATTGACGTCAATGACGGTAAATGGCCCGCC
TGGCATTATGCCCAGTACATGACCTTATGGGACTTTCCTACTTGGCAGTACATCTACGTATTAGTCATCGCTATTACCATGGTCGA
GGTGAGCCCCACGTTCTGCTTCACTCTCCCCATCTCCCCCCCCTCCCCACCCCCAATTTTGTATTTATTTATTTTTTAATTATTTTG
TGCAGCGATGGGGGCGGGGGGGGGGGGGGCGCGCGCCAGGCGGGGCGGGGCGGGGCGAGGGGCGGGGCGGGGCGAGGC
GGAGAGGTGCGGCGGCAGCCAATCAGAGCGGCGCGCTCCGAAAGTTTCCTTTTATGGCGAGGCGGCGGCGGCGGCGGCCCT
ATAAAAAGCGAAGCGCGCGGCGGGCGGGAGTCGCTGCGTTGCCTTCGCCCCGTGCCCCGCTCCGCGCCGCCTCGCGCCGCC
CGCCCCGGCTCTGACTGACCGCGTTACTCCCACAGGTGAGCGGGCGGGACGGCCCTTCTCCTCCGGGCTGTAATTAGCGCTTG
GTTTAATGACGGCTCGTTTCTTTTCTGTGGCTGCGTGAAAGCCTTAAAGGGCTCCGGGAGGGCCCTTTGTGCGGGGGGGAGCG
GCTCGGGGGGTGCGTGCGTGTGTGTGTGCGTGGGGAGCGCCGCGTGCGGCCCGCGCTGCCCGGCGGCTGTGAGCGCTGCG
GGCGCGGCGCGGGGCTTTGTGCGCTCCGCGTGTGCGCGAGGGGAGCGCGGCCGGGGGCGGTGCCCCGCGGTGCGGGGGG
GCTGCGAGGGGAACAAAGGCTGCGTGCGGGGTGTGTGCGTGGGGGGGTGAGCAGGGGGTGTGGGCGCGGCGGTCGGGCTG
TAACCCCCCCCTGCACCCCCCTCCCCGAGTTGCTGAGCACGGCCCGGCTTCGGGTGCGGGGCTCCGTACGGGGCGTGGCGC
GGGGCTCGCCGTGCCGGGCGGGGGGTGGCGGCAGGTGGGGGTGCCGGGCGGGGCGGGGCCGCCTCGGGCCGGGGAGGG
CTCGGGGGAGGGGCGCGGCGGCCCCCGGAGCGCCGGCGGCTGTCGAGGCGCGGCGAGCCGCAGCCATTGCCTTTTATGGTA
ATCGTGCGAGAGGGCGCAGGGACTTCCTTTGTCCCAAATCTGTGCGGAGCCGAAATCTGGGAGGCGCCGCCGCACCCCCTCTA
GCGGGCGCGGGGCGAAGCGGTGCGGCGCCGGCAGGAAGGAAATGGGCGGGGAGGGCCTTCGTGCGTCGCCGCGCCGCCGT
CCCCTTCTCCCTCTCCAGCCTCGGGGCTGTCCGCGGGGGGACGGCTGCCTTCGGGGGGGACGGGGCAGGGCGGGGTTCGGC
TTCTGGCGTGTGACCGGCGGCTCTAGAGCCTCTGCTAACCATGTTCATGCCTTCTTCTTTTTCCTACAGCTCCTGGGCAACGTGC
TGGTTATTGTGCTGTCTCATCATTTTGGCAAAGAATTCGCCACCATGGTGAGCAAGGGCGAGGAGGATAACATGGCCATCATCA
AGGAGTTCATGCGCTTCAAGGTGCACATGGAGGGCTCCGTGAACGGCCACGAGTTCGAGATCGAGGGCGAGGGCGAGGGCC
GCCCCTACGAGGGCACCCAGACCGCCAAGCTGAAGGTGACCAAGGGTGGCCCCCTGCCCTTCGCCTGGGACATCCTGTCCC
CTCAGTTCATGTACGGCTCCAAGGCCTACGTGAAGCACCCCGCCGACATCCCCGACTACTTGAAGCTGTCCTTCCCCGAGGG
CTTCAAGTGGGAGCGCGTGATGAACTTCGAGGACGGCGGCGTGGTGACCGTGACCCAGGACTCCTCCCTGCAGGACGGCGA
GTTCATCTACAAGGTGAAGCTGCGCGGCACCAACTTCCCCTCCGACGGCCCCGTAATGCAGAAGAAGACCATGGGCTGGGA
GGCCTCCTCCGAGCGGATGTACCCCGAGGACGGCGCCCTGAAGGGCGAGATCAAGCAGAGGCTGAAGCTGAAGGACGGCG
GCCACTACGACGCTGAGGTCAAGACCACCTACAAGGCCAAGAAGCCCGTGCAGCTGCCCGGCGCCTACAACGTCAACATCA
AGTTGGACATCACCTCCCACAACGAGGACTACACCATCGTGGAACAGTACGAACGCGCCGAGGGCCGCCACTCCACCGGCG
GCATGGACGAGCTGTACGCGTCTGGAGGCGCCACCAATTTCAGCCTGCTGAAACAGGCTGGCGACGTGGAAGAGAACCCTG
GACCTGGACAAAAGTCGGTGAGCAAGGGCGAGGAGCTGTTCACCGGGGTGGTGCCCATCCTGGTCGAGCTGGACGGCGACG
TAAACGGCCACAAGTTCAGCGTGTCCGGCGAGGGCGAGGGCGATGCCACCTACGGCAAGCTGACCCTGAAGTTCATCTGCA
CCACCGGCAAGCTGCCCGTGCCCTGGCCCACCCTCGTGACCACCCTGACCTACGGCGTGCAGTGCTTCAGCCGCTACCCCGA
CCACATGAAGCAGCACGACTTCTTCAAGTCCGCCATGCCCGAAGGCTACGTCCAGGAGCGCACCATCTTCTTCAAGGACGAC
GGCAACTACAAGACCCGCGCCGAGGTGAAGTTCGAGGGCGACACCCTGGTGAACCGCATCGAGCTGAAGGGCATCGACTTC
AAGGAGGACGGCAACATCCTGGGGCACAAGCTGGAGTACAACTACAACAGCCACTAGAGTCTATATCATGGCCGACAAGCA
GAAGAACGGCATCAAGGTGAACTTCAAGATCCGCCACAACATCGAGGACGGCAGCGTGCAGCTCGCCGACCACTACCAGCA
GAACACCCCCATCGGCGACGGCCCCGTGCTGCTGCCCGACAACCACTACCTGAGCACCCAGTCCGCCCTGAGCAAAGACCC
CAACGAGAAGCGCGATCACATGGTCCTGCTGGAGTTCGTGACCGCCGCCGGGATCACTCTCGGCATGGACGAGCTGTACAA
GTAAGAATTCACTCCTCAGGTGCAGGCTGCCTATCAGAAGGTGGTGGCTGGTGTGGCCAATGCCCTGGCTCACAAATACCACTG
AGATCTTTTTCCCTCTGCCAAAAATTATGGGGACATCATGAAGCCCCTTGAGCATCTGACTTCTGGCTAATAAAGGAAATTTATTT
TCATTGCAATAGTGTGTTGGAATTTTTTGTGTCTCTCACTCGGAAGGACATATGGGAGGGGTCGACAATCAACCTCTGGATTACA
AAATTTGTGAAAGATTGACTGGTATTCTTAACTATGTTGCTCCTTTTACGCTATGTGGATACGCTGCTTTAATGCCTTTGTATCATG
CGTTAACTAAACTTGTTTATTGCAGCTTATAATGGTTACAAATAAAGCAATAGCATCACAAATTTCACAAATAAAGCATTTTTTTCAC
TGCATTCTAGTTGTGGTTTGTCCAAACTCATCAATGTATCTTATCATGTCTGGAATTGACTCAAATGATGTCAATTAGTCTATCAGA
AGCTATCTGGTCTCCCTTCCGGGGGACAAGACATCCCTGTTTAATATTTAAACAGCAGTGTTCCCAAACTGGGTTCTTATATCCCT
TGCTCTGGTCAACCAGGTTGCAGGGTTTCCTGTCCTCACAGGAACGAAGTCCCTAAAGAAACAGTGGCAGCCAGGTTTAGCCCC
GGAATTGACTGGATTCCTTTTTTAGGGCCCATTGGTATGGCTTTTTCCCCGTATCCCCCCAGGTGTCTGCAGGCTCAAAGAGCAG
CGAGAAGCGTTCAGAGGAAAGCGATCCCGTGCCACCTTCCCCGTGCCCGGGCTGTCCCCGCACGCTGCCGGCTCGGGGATGC
GGGGGGAGCGCCGGACCGGAGCGGAGCCCCGGGCGGCTCGCTGCTGCCCCCTAGCGGGGGAGGGACGTAATTACATCCCTG
GGGGCTTTGGGGGGGGGCTGTCCCTGATATCTATAACAAGAAAATATATATATAATAAGTTATCACGTAAGTAGAACATGAAATAA
CAATATAATTATCGTATGAGTTAAATCTTAAAAGTCACGTAAAAGATAATCATGCGTCATTTTGACTCACGCGGTCGTTATAGTTCA
AAATCAGTGACACTTACCGCATTGACAAGCACGCCTCACGGGAGCTCCAAGCGGCGACTGAGATGTCCTAAATGCACAGCGACG
GATTCGCGCTATTTAGAAAGAGAGAGCAATATTTCAAGAATGCATGCGTCAATTTTACGCAGACTATCTTTCTAGGGTTAATCTAG
CTGCATCAGGATCATATCGTCGGGTCTTTTTTCCGGCTCAGTCATCGCCCAAGCTGGCGCTATCTGGGCATCGGGGAGGAAGAA
GCCCGTGCCTTTTCCCGCGAGGTTGAAGCGGCATGGAAAGAGTTTGCCGAGGATGACTGCTGCTGCATTGACGTTGAGCGAAA
ACGCACGTTTACCATGATGATTCGGGAAGGTGTGGCCATGCACGCCTTTAACGGTGAACTGTTCGTTCAGGCCACCTGGGATAC
CAGTTCGTCGCGGCTTTTCCGGACACAGTTCCGGATGGTCAGCCCGAAGCGCATCAGCAACCCGAACAATACCGGCGACAGCC
GGAACTGCCGTGCCGGTGTGCAGATTAATGACAGCGGTGCGGCGCTGGGATATTACGTCAGCGAGGACGGGTATCCTGGCTG

GATGCCGCAGAAATGGACATGGATACCCCGTGAGTTACCCGGCGGGCGCGCTTGGCGTAATCATGGTCATAGCTGTTTCCTGT
GTGAAATTGTTATCCGCTCACAATTCCACACAACATACGAGCCGGAAGCATAAAGTGTAAAGCCTGGGGTGCCTAATGAGTGAG
CTAACTCACATTAATTGCGTTGCGCTCACTGCCCGCTTTCCAGTCGGGAAACCTGTCGTGCCAGCTGCATTAATGAATCGGCCAA
CGCGCGGGGAGAGGCGGTTTGCGTATTGGGCGCTCTTCCGCTTCCTCGCTCACTGACTCGCTGCGCTCGGTCGTTCGGCTGCG
GCGAGCGGTATCAGCTCACTCAAAGGCGGTAATACGGTTATCCACAGAATCAGGGGATAACGCAGGAAAGAACATGTGAGCAAA
AGGCCAGCAAAAGGCCAGGAACCGTAAAAAGGCCGCGTTGCTGGCGTTTTTCCATAGGCTCCGCCCCCCTGACGAGCATCACA
AAAATCGACGCTCAAGTCAGAGGTGGCGAAACCCGACAGGACTATAAAGATACCAGGCGTTTCCCCCTGGAAGCTCCCTCGTGC
GCTCTCCTGTTCCGACCCTGCCGCTTACCGGATACCTGTCCGCCTTTCTCCCTTCGGGAAGCGTGGCGCTTTCTCATAGCTCAC
GCTGTAGGTATCTCAGTTCGGTGTAGGTCGTTCGCTCCAAGCTGGGCTGTGTGCACGAACCCCCCGTTCAGCCCGACCGCTGC
GCCTTATCCGGTAACTATCGTCTTGAGTCCAACCCGGTAAGACACGACTTATCGCCACTGGCAGCAGCCACTGGTAACAGGATT
AGCAGAGCGAGGTATGTAGGCGGTGCTACAGAGTTCTTGAAGTGGTGGCCTAACTACGGCTACACTAGAAGGACAGTATTTGGT
ATCTGCGCTCTGCTGAAGCCAGTTACCTTCGGAAAAAGAGTTGGTAGCTCTTGATCCGGCAAACAAACCACCGCTGGTAGCGGT
GGTTTTTTTGTTTGCAAGCAGCAGATTACGCGCAGAAAAAAAGGATCTCAAGAAGATCCTTTGATCTTTTCTACGGGGTCTGACG
CTCAGTGGAACGAAAACTCACGTTAAGGGATTTTGGTCATGAGATTATCAAAAAGGATCTTCACCTAGATCCTTTTAAATTAAAAAT
GAAGTTTTAAATCAATCTAAAGTATATATGAGTAAACTTGGTCTGACAGTTACCAATGCTTAATCAGTGAGGCACCTATCTCAGCG
ATCTGTCTATTTCGTTCATCCATAGTTGCCTGACTCCCCGTCGTGTAGATAACTACGATACGGGAGGGCTTACCATCTGGCCCCA
GTGCTGCAATGATACCGCGAGACCCACGCTCACCGGCTCCAGATTTATCAGCAATAAACCAGCCAGCCGGAAGGGCCGAGCGC
AGAAGTGGTCCTGCAACTTTATCCGCCTCCATCCAGTCTATTAATTGTTGCCGGGAAGCTAGAGTAAGTAGTTCGCCAGTTAATA
GTTTGCGCAACGTTGTTGCCATTGCTACAGGCATCGTGGTGTCACGCTCGTCGTTTGGTATGGCTTCATTCAGCTCCGGTTCCCA
ACGATCAAGGCGAGTTACATGATCCCCCATGTTGTGCAAAAAAGCGGTTAGCTCCTTCGGTCCTCCGATCGTTGTCAGAAGTAAG
TTGGCCGCAGTGTTATCACTCATGGTTATGGCAGCACTGCATAATTCTCTTACTGTCATGCCATCCGTAAGATGCTTTTCTGTGAC
TGGTGAGTACTCAACCAAGTCATTCTGAGAATAGTGTATGCGGCGACCGAGTTGCTCTTGCCCGGCGTCAATACGGGATAATAC
CGCGCCACATAGCAGAACTTTAAAAGTGCTCATCATTGGAAAACGTTCTTCGGGGCGAAAACTCTCAAGGATCTTACCGCTGTTG
AGATCCAGTTCGATGTAACCCACTCGTGCACCCAACTGATCTTCAGCATCTTTTACTTTCACCAGCGTTTCTGGGTGAGCAAAAA
CAGGAAGGCAAAATGCCGCAAAAAAGGGAATAAGGGCGACACGGAAATGTTGAATACTCAT

2.2.8 Vector 61: Pyl tRNA$_{UCUA(Ev2)}$ PylRS mCherry-P2A-eGFP(150TAGA)

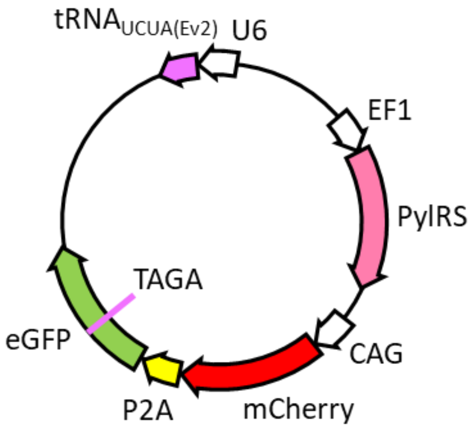

ACTCTTCCTTTTTCAATATTATTGAAGCATTTATCAGGGTTATTGTCTCATGAGCGGATACATATTTGAATGTATTTAGAAAAATAAA
CAAATAGGGGTTCCGCGCACATTTCCCCGAAAAGTGCCACCTAAATTGTAAGCGTTAATATTTTGTTAAAATTCGCGTTAAATTTTT
GTTAAATCAGCTCATTTTTTAACCAATAGGCCGAAATCGGCAAAATCCCTTATAAATCAAAAGAATAGACCGAGATAGGGTTGAGT
GTTGTTCCAGTTTGGAACAAGAGTCCACTATTAAAGAACGTGGACTCCAACGTCAAAGGGCGAAAAACCGTCTATCAGGGCGAT
GGCCCACTACGTGAACCATCACCCTAATCAAGTTTTTTGGGGTCGAGGTGCCGTAAAGCACTAAATCGGAACCCTAAAGGGAGC
CCCCGATTTAGAGCTTGACGGGGAAAGCCGGCGAACGTGGCGAGAAAGGAAGGGAAGAAAGCGAAAGGAGCGGGCGCTAGGG
CGCTGGCAAGTGTAGCGGTCACGCTGCGCGTAACCACCACACCCGCCGCGCTTAATGCGCCGCTACAGGGCGCGTCCCATTC
GCCATTCAGGCTGCGCAACTGTTGGGAAGGGCGATCGGTGCGGGCCTCTTCGCTATTACGCCAGCTGGCGAAAGGGGGATGT
GCTGCAAGGCGATTAAGTTGGGTAACGCCAGGGTTTTCCCAGTCACGACGTTGTAAAACGACGGCCAGTGAGCGCGCCTCGTT
CATTCACGTTTTTGAACCCGTGGAGGACGGGCAGACTCGCGGTGCAAATGTGTTTTACAGCGTGATGGAGCAGATGAAGATGCT
CGACACGCTGCAGAACACGCAGCTAGATTAACCCTAGAAAGATAATCATATTGTGACGTACGTTAAAGATAATCATGCGTAAAATT
GACGCATGTGTTTTATCGGTCTGTATATCGAGGTTTATTTATTAATTTGAATAGATATTAAGTTTTATTATATTTACACTTACATACTA
ATAATAAATTCAACAAACAATTTATTTATGTTTATTTATTTATTAAAAAAAACAAAAACTCAAAATTTCTTCTATAAAGTAACAAAACTT
TTATGAGGGACAGCCCCCCCCCAAAGCCCCCAGGGATGTAATTACGTCCCTCCCCCGCTAGGGGGCAGCAGCGAGCCGCCCG
GGGCTCCGCTCCGGTCCGGCGCTCCCCCCGCATCCCCGAGCCGGCAGCGTGCGGGGACAGCCCGGGCACGGGGAAGGTGG
CACGGGATCGCTTTCCTCTGAACGCTTCTCGCTGCTCTTTGAGCCTGCAGACACCTGGGGGGATACGGGGAAAAGGCCTAAAAA
CCGCACTTGTC**CGGAAACCCCGGGAATCTAACCCGGCTGAAGCGGATTAGAAGCCCATTCGATCTACATGATCAGGTTTCC**G
GTGTTTCGTCCTTTCCACAAGATATATAAAGCCAAGAAATCGAAATACTTTCAAGTTACGGTAAGCATATGATAGTCCATTTTAAAA
CATAATTTTAAAACTGCAAACTACCCAAGAAATTATTACTTTCTACGTCACGTATTTTGTACTAATATCTTTGTGTTTACAGTCAAAT
TAATTCTAATTATCTCTCTAACAGCCTTGTATCGTATATGCAAATATGAAGGAATCATGGGAAATAGGCCCTCTTCCTGCCCAACC
GGTTGCGATCGCTCCGGTGCCCGTCAGTGGGCAGAGCGCACATCGCCCACAGTCCCCGAGAAGTTGGGGGGAGGGGTCGGCA
ATTGAACGGGTGCCTAGAGAAGGTGGCGCGGGGTAAACTGGGAAAGTGATGTCGTGTACTGGCTCCGCCTTTTTCCCGAGGGT
GGGGGAGAACCGTATATAAGTGCAGTAGTCGCCGTGAACGTTCTTTTTCGCAACGGGTTTGCCGCCAGAACACAGCTGAAGCTT
CGAGGGGCTCGCATCTCTCCTTCACGCGCCCGCCGCCCTACCTGAGGCCGCCATCCACGCCGGTTGAGTCGCGTTCTGCCGC
CTCCCGCCTGTGGTGCCTCCTGAACTGCGTCCGCCGTCTAGGTAAGTTTAAAGCTCAGGTCGAGACCGGGCCTTTGTCCGGCG
CTCCCTTGGAGCCTACCTAGACTCAGCCGGCTCTCCACGCTTTGCCTGACCCTGCTTGCTCAACTCTACGTCTTTGTTTCGTTTT
CTGTTCTGCGCCGTTACAGATCCAAGCTGTGACCGGCGCCTACTCTACAGATAGCGTTTAAACTTACGCTTGCCACC**ATGGCTA**
GCGACTACAAGGACGACGACGACAAGGACAAGAAGCCCCTGAACACCCTGATCAGCGCCACAGGACTGTGGATGTCCAGAA
CCGGCACCATCCACAAGATCAAGCACCACGAGGTGTCCCGGTCCAAAATCTACATCGAGATGGCCTGCGGCGATCACCTGGT

CGTCAACAACAGCAGAAGCAGCCGGACAGCCAGAGCCCTGCGGCACCACAAGTACAGAAAGACCTGCAAGCGGTGCAGAG
TGTCCGACGAGGACCTGAACAAGTTCCTGACCAAGGCCAACGAGGACCAGACCAGCGTGAAAGTGAAGGTGGTGTCCGCCC
CCACCCGGACCAAGAAAGCCATGCCCAAGAGCGTGGCCAGAGCCCCCAAGCCCCTGGAAAACACCGAAGCCGCTCAGGCC
CAGCCCAGCGGCAGCAAGTTCAGCCCCGCCATCCCCGTGTCTACCCAGGAAAGCGTCAGCGTCCCCGCCAGCGTGTCCACC
AGCATCTCTAGCATCTCAACCGGCGCCACAGCTTCTGCCCTGGTCAAGGGCAACACCAACCCCATCACCAGCATGTCTGCCC
CTGTGCAAGCCTCTGCCCCAGCCCTGACCAAGTCCCAGACCGACCGGCTGGAAGTGCTCCTGAACCCCAAGGACGAGATCA
GCCTGAACAGCGGCAAGCCCTTCCGGGAGCTGGAAAGCGAGCTGCTGAGCCGGCGGAAGAAGGACCTCCAGCAAATCTACG
CCGAGGAACGGGAGAACTACCTGGGCAAGCTGGAAAGAGAGATCACCCGGTTCTTCGTGGACCGGGGCTTCCTGGAAATCA
AGAGCCCCATCCTGATCCCCCTGGAGTACATCGAGCGGATGGGCATCGACAACGACACCGAGCTGAGCAAGCAGATTTTCCG
GGTGGACAAGAACTTCTGCCTGCGGCCCATGCTGGCCCCCAACCTGTACAACTACCTGCGGAAACTGGATCGCGCTCTGCCC
GACCCCATCAAGATTTTCGAGATCGGCCCCTGCTACCGGAAAGAGAGCGACGGCAAAGAGCACCTGGAAGAGTTTACAATGC
TGAACTTTTGCCAGATGGGCAGCGGCTGCACCAGAGAGAACCTGGAATCCATCATCACCGACTTTCTGAACCACCTGGGGAT
CGACTTCAAGATCGTGGGCGACAGCTGCATGGTGTACGGCGACACCCTGGACGTGATGCACGGCGACCTGGAACTGTCTAG
CGCCGTCGTGGGACCCATCCCTCTGGACCGGGAGTGGGGCATCGATAAGCCCTGGATCGGAGCCGGCTTCGGCCTGGAACG
GCTGCTGAAAGTCAAGCACGACTTTAAGAACATCAAGCGGGCTGCCAGAAGCGAGAGCTACTACAACGGCATCAGCACCAA
CCTGTGATGATAAGGATCCACTAGTCCAGTGTGGTGGAATTGACATTGATTATTGACTAGTTATTAATAGTAATCAATTACGGGGT
CATTAGTTCATAGCCCATATATGGAGTTCCGCGTTACATAACTTACGGTAAATGGCCCGCCTGGCTGACCGCCCAACGACCCCC
GCCCATTGACGTCAATAATGACGTATGTTCCCATAGTAACGCCAATAGGGACTTTCCATTGACGTCAATGGGTGGAGTATTTACG
GTAAACTGCCCACTTGGCAGTACATCAAGTGTATCATATGCCAAGTACGCCCCCTATTGACGTCAATGACGGTAAATGGCCCGCC
TGGCATTATGCCCAGTACATGACCTTATGGGACTTTCCTACTTGGCAGTACATCTACGTATTAGTCATCGCTATTACCATGGTCGA
GGTGAGCCCCACGTTCTGCTTCACTCTCCCCATCTCCCCCCCCTCCCCACCCCCAATTTTGTATTTATTTATTTTTTAATTATTTTG
TGCAGCGATGGGGGCGGGGGGGGGGGGGGCGCGCGCCAGGCGGGGCGGGGCGGGGCGAGGGGCGGGGCGGGGCGAGGC
GGAGAGGTGCGGCGGCAGCCAATCAGAGCGGCGCGCTCCGAAAGTTTCCTTTTATGGCGAGGCGGCGGCGGCGGCGGCCCT
ATAAAAAGCGAAGCGCGCGGCGGGCGGGAGTCGCTGCGTTGCCTTCGCCCCGTGCCCCGCTCCGCGCCGCCTCGCGCCGCC
CGCCCCGGCTCTGACTGACCGCGTTACTCCCACAGGTGAGCGGGCGGGACGGCCCTTCTCCTCCGGGCTGTAATTAGCGCTTG
GTTTAATGACGGCTCGTTTCTTTTCTGTGGCTGCGTGAAAGCCTTAAAGGGCTCCGGGAGGGCCCTTTGTGCGGGGGGGAGCG
GCTCGGGGGGTGCGTGCGTGTGTGTGTGCGTGGGGAGCGCCGCGTGCGGCCCGCGCTGCCCGGCGGCTGTGAGCGCTGCG
GGCGCGGCGCGGGGCTTTGTGCGCTCCGCGTGTGCGCGAGGGGAGCGCGGCCGGGGGCGGTGCCCCGCGGTGCGGGGGG
GCTGCGAGGGGAACAAAGGCTGCGTGCGGGGTGTGTGCGTGGGGGGGTGAGCAGGGGGTGTGGGCGCGGCGGTCGGGCTG
TAACCCCCCCCTGCACCCCCCTCCCCGAGTTGCTGAGCACGGCCCGGCTTCGGGTGCGGGGCTCCGTACGGGGCGTGGCGC
GGGGCTCGCCGTGCCGGGCGGGGGGTGGCGGCAGGTGGGGGTGCCGGGCGGGGCGGGGCCGCCTCGGGCCGGGGAGGG
CTCGGGGGAGGGGCGCGGCGGCCCCCGGAGCGCCGGCGGCTGTCGAGGCGCGGCGAGCCGCAGCCATTGCCTTTTATGGTA
ATCGTGCGAGAGGGCGCAGGGACTTCCTTTGTCCCAAATCTGTGCGGAGCCGAAATCTGGGAGGCGCCGCCGCACCCCCTCTA
GCGGGCGCGGGGCGAAGCGGTGCGGCGCCGGCAGGAAGGAAATGGGCGGGGAGGGCCTTCGTGCGTCGCCGCGCCGCCGT
CCCCTTCTCCCTCTCCAGCCTCGGGGCTGTCCGCGGGGGGACGGCTGCCTTCGGGGGGGACGGGGCAGGGCGGGGTTCGGC
TTCTGGCGTGTGACCGGCGGCTCTAGAGCCTCTGCTAACCATGTTCATGCCTTCTTCTTTTTCCTACAGCTCCTGGGCAACGTGC
TGGTTATTGTGCTGTCTCATCATTTTGGCAAAGAATTCGCCACCATGGTGAGCAAGGGCGAGGAGGATAACATGGCCATCATCA
AGGAGTTCATGCGCTTCAAGGTGCACATGGAGGGCTCCGTGAACGGCCACGAGTTCGAGATCGAGGGCGAGGGCGAGGGCC
GCCCCTACGAGGGCACCCAGACCGCCAAGCTGAAGGTGACCAAGGGTGGCCCCCTGCCCTTCGCCTGGGACATCCTGTCCC
CTCAGTTCATGTACGGCTCCAAGGCCTACGTGAAGCACCCCGCCGACATCCCCGACTACTTGAAGCTGTCCTTCCCCGAGGG
CTTCAAGTGGGAGCGCGTGATGAACTTCGAGGACGGCGGCGTGGTGACCGTGACCCAGGACTCCTCCCTGCAGGACGGCGA
GTTCATCTACAAGGTGAAGCTGCGCGGCACCAACTTCCCCTCCGACGGCCCCGTAATGCAGAAGAAGACCATGGGCTGGGA
GGCCTCCTCCGAGCGGATGTACCCCGAGGACGGCGCCCTGAAGGGCGAGATCAAGCAGAGGCTGAAGCTGAAGGACGGCG
GCCACTACGACGCTGAGGTCAAGACCACCTACAAGGCCAAGAAGCCCGTGCAGCTGCCCGGCGCCTACAACGTCAACATCA
AGTTGGACATCACCTCCCACAACGAGGACTACACCATCGTGGAACAGTACGAACGCGCCGAGGGCCGCCACTCCACCGGCG
GCATGGACGAGCTGTACGCGTCTGGAGGCGCCACCAATTTCAGCCTGCTGAAACAGGCTGGCGACGTGGAAGAGAACCCTG
GACCTGGACAAAAGTCGGTGAGCAAGGGCGAGGAGCTGTTCACCGGGGTGGTGCCCATCCTGGTCGAGCTGGACGGCGACG
TAAACGGCCACAAGTTCAGCGTGTCCGGCGAGGGCGAGGGCGATGCCACCTACGGCAAGCTGACCCTGAAGTTCATCTGCA
CCACCGGCAAGCTGCCCGTGCCCTGGCCCACCCTCGTGACCACCCTGACCTACGGCGTGCAGTGCTTCAGCCGCTACCCCGA
CCACATGAAGCAGCACGACTTCTTCAAGTCCGCCATGCCCGAAGGCTACGTCCAGGAGCGCACCATCTTCTTCAAGGACGAC
GGCAACTACAAGACCCGCGCCGAGGTGAAGTTCGAGGGCGACACCCTGGTGAACCGCATCGAGCTGAAGGGCATCGACTTC
AAGGAGGACGGCAACATCCTGGGGCACAAGCTGGAGTACAACTACAACAGCCACTAGAGTCTATATCATGGCCGACAAGCA
GAAGAACGGCATCAAGGTGAACTTCAAGATCCGCCACAACATCGAGGACGGCAGCGTGCAGCTCGCCGACCACTACCAGCA
GAACACCCCCATCGGCGACGGCCCCGTGCTGCTGCCCGACAACCACTACCTGAGCACCCAGTCCGCCCTGAGCAAAGACCC
CAACGAGAAGCGCGATCACATGGTCCTGCTGGAGTTCGTGACCGCCGCCGGGATCACTCTCGGCATGGACGAGCTGTACAA
GTAAGAATTCACTCCTCAGGTGCAGGCTGCCTATCAGAAGGTGGTGGCTGGTGTGGCCAATGCCCTGGCTCACAAATACCACTG
AGATCTTTTTCCCTCTGCCAAAAATTATGGGGACATCATGAAGCCCCTTGAGCATCTGACTTCTGGCTAATAAAGGAAATTTATTT
TCATTGCAATAGTGTGTTGGAATTTTTTGTGTCTCTCACTCGGAAGGACATATGGGAGGGGTCGACAATCAACCTCTGGATTACA
AAATTTGTGAAAGATTGACTGGTATTCTTAACTATGTTGCTCCTTTTACGCTATGTGGATACGCTGCTTTAATGCCTTTGTATCATG
CGTTAACTAAACTTGTTTATTGCAGCTTATAATGGTTACAAATAAAGCAATAGCATCACAAATTTCACAAATAAAGCATTTTTTTCAC
TGCATTCTAGTTGTGGTTTGTCCAAACTCATCAATGTATCTTATCATGTCTGGAATTGACTCAAATGATGTCAATTAGTCTATCAGA
AGCTATCTGGTCTCCCTTCCGGGGGACAAGACATCCCTGTTTAATATTTAAACAGCAGTGTTCCCAAACTGGGTTCTTATATCCCT
TGCTCTGGTCAACCAGGTTGCAGGGTTTCCTGTCCTCACAGGAACGAAGTCCCTAAAGAAACAGTGGCAGCCAGGTTTAGCCCC
GGAATTGACTGGATTCCTTTTTTAGGGCCCATTGGTATGGCTTTTTCCCCGTATCCCCCCAGGTGTCTGCAGGCTCAAAGAGCAG
CGAGAAGCGTTCAGAGGAAAGCGATCCCGTGCCACCTTCCCCGTGCCCGGGCTGTCCCCGCACGCTGCCGGCTCGGGGATGC
GGGGGGAGCGCCGGACCGGAGCGGAGCCCCGGGCGGCTCGCTGCTGCCCCCTAGCGGGGGAGGGACGTAATTACATCCCTG
GGGGCTTTGGGGGGGGGCTGTCCCTGATATCTATAACAAGAAAATATATATATAATAAGTTATCACGTAAGTAGAACATGAAATAA
CAATATAATTATCGTATGAGTTAAATCTTAAAAGTCACGTAAAAGATAATCATGCGTCATTTTGACTCACGCGGTCGTTATAGTTCA
AAATCAGTGACACTTACCGCATTGACAAGCACGCCTCACGGGAGCTCCAAGCGGCGACTGAGATGTCCTAAATGCACAGCGACG
GATTCGCGCTATTTAGAAAGAGAGAGCAATATTTCAAGAATGCATGCGTCAATTTTACGCAGACTATCTTTCTAGGGTTAATCTAG
CTGCATCAGGATCATATCGTCGGGTCTTTTTTCCGGCTCAGTCATCGCCCAAGCTGGCGCTATCTGGGCATCGGGGAGGAAGAA
GCCCGTGCCTTTTCCCGCGAGGTTGAAGCGGCATGGAAAGAGTTTGCCGAGGATGACTGCTGCTGCATTGACGTTGAGCGAAA
ACGCACGTTTACCATGATGATTCGGGAAGGTGTGGCCATGCACGCCTTTAACGGTGAACTGTTCGTTCAGGCCACCTGGGATAC
CAGTTCGTCGCGGCTTTTCCGGACACAGTTCCGGATGGTCAGCCCGAAGCGCATCAGCAACCCGAACAATACCGGCGACAGCC

GGAACTGCCGTGCCGGTGTGCAGATTAATGACAGCGGTGCGGCGCTGGGATATTACGTCAGCGAGGACGGGTATCCTGGCTG
GATGCCGCAGAAATGGACATGGATACCCCGTGAGTTACCCGGCGGGCGCGCTTGGCGTAATCATGGTCATAGCTGTTTCCTGT
GTGAAATTGTTATCCGCTCACAATTCCACACAACATACGAGCCGGAAGCATAAAGTGTAAAGCCTGGGGTGCCTAATGAGTGAG
CTAACTCACATTAATTGCGTTGCGCTCACTGCCCGCTTTCCAGTCGGGAAACCTGTCGTGCCAGCTGCATTAATGAATCGGCCAA
CGCGCGGGGAGAGGCGGTTTGCGTATTGGGCGCTCTTCCGCTTCCTCGCTCACTGACTCGCTGCGCTCGGTCGTTCGGCTGCG
GCGAGCGGTATCAGCTCACTCAAAGGCGGTAATACGGTTATCCACAGAATCAGGGGATAACGCAGGAAAGAACATGTGAGCAAA
AGGCCAGCAAAAGGCCAGGAACCGTAAAAAGGCCGCGTTGCTGGCGTTTTTCCATAGGCTCCGCCCCCCTGACGAGCATCACA
AAAATCGACGCTCAAGTCAGAGGTGGCGAAACCCGACAGGACTATAAAGATACCAGGCGTTTCCCCCTGGAAGCTCCCTCGTGC
GCTCTCCTGTTCCGACCCTGCCGCTTACCGGATACCTGTCCGCCTTTCTCCCTTCGGGAAGCGTGGCGCTTTCTCATAGCTCAC
GCTGTAGGTATCTCAGTTCGGTGTAGGTCGTTCGCTCCAAGCTGGGCTGTGTGCACGAACCCCCCGTTCAGCCCGACCGCTGC
GCCTTATCCGGTAACTATCGTCTTGAGTCCAACCCGGTAAGACACGACTTATCGCCACTGGCAGCAGCCACTGGTAACAGGATT
AGCAGAGCGAGGTATGTAGGCGGTGCTACAGAGTTCTTGAAGTGGTGGCCTAACTACGGCTACACTAGAAGGACAGTATTTGGT
ATCTGCGCTCTGCTGAAGCCAGTTACCTTCGGAAAAAGAGTTGGTAGCTCTTGATCCGGCAAACAAACCACCGCTGGTAGCGGT
GGTTTTTTTGTTTGCAAGCAGCAGATTACGCGCAGAAAAAAAGGATCTCAAGAAGATCCTTTGATCTTTTCTACGGGGTCTGACG
CTCAGTGGAACGAAAACTCACGTTAAGGGATTTTGGTCATGAGATTATCAAAAAGGATCTTCACCTAGATCCTTTTAAATTAAAAAT
GAAGTTTTAAATCAATCTAAAGTATATATGAGTAAACTTGGTCTGACAGTTACCAATGCTTAATCAGTGAGGCACCTATCTCAGCG
ATCTGTCTATTTCGTTCATCCATAGTTGCCTGACTCCCCGTCGTGTAGATAACTACGATACGGGAGGGCTTACCATCTGGCCCCA
GTGCTGCAATGATACCGCGAGACCCACGCTCACCGGCTCCAGATTTATCAGCAATAAACCAGCCAGCCGGAAGGGCCGAGCGC
AGAAGTGGTCCTGCAACTTTATCCGCCTCCATCCAGTCTATTAATTGTTGCCGGGAAGCTAGAGTAAGTAGTTCGCCAGTTAATA
GTTTGCGCAACGTTGTTGCCATTGCTACAGGCATCGTGGTGTCACGCTCGTCGTTTGGTATGGCTTCATTCAGCTCCGGTTCCCA
ACGATCAAGGCGAGTTACATGATCCCCCATGTTGTGCAAAAAAGCGGTTAGCTCCTTCGGTCCTCCGATCGTTGTCAGAAGTAAG
TTGGCCGCAGTGTTATCACTCATGGTTATGGCAGCACTGCATAATTCTCTTACTGTCATGCCATCCGTAAGATGCTTTTCTGTGAC
TGGTGAGTACTCAACCAAGTCATTCTGAGAATAGTGTATGCGGCGACCGAGTTGCTCTTGCCCGGCGTCAATACGGGATAATAC
CGCGCCACATAGCAGAACTTTAAAAGTGCTCATCATTGGAAAACGTTCTTCGGGGCGAAAACTCTCAAGGATCTTACCGCTGTTG
AGATCCAGTTCGATGTAACCCACTCGTGCACCCAACTGATCTTCAGCATCTTTTACTTTCACCAGCGTTTCTGGGTGAGCAAAAA
CAGGAAGGCAAAATGCCGCAAAAAAGGGAATAAGGGCGACACGGAAATGTTGAATACTCAT

2.2.9 Vector 62: Pyl tRNA$_{CUAG}$ PylRS mCherry-P2A-eGFP(150CTAG)

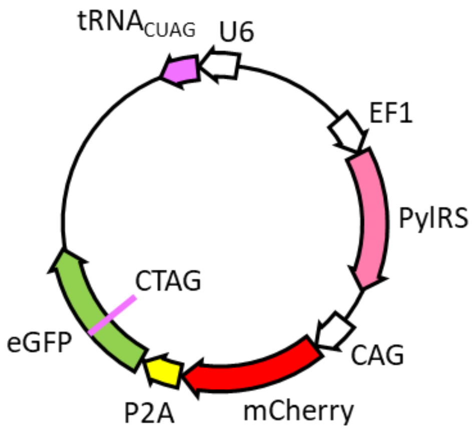

ACTCTTCCTTTTTCAATATTATTGAAGCATTTATCAGGGTTATTGTCTCATGAGCGGATACATATTTGAATGTATTTAGAAAAATAAA
CAAATAGGGGTTCCGCGCACATTTCCCCGAAAAGTGCCACCTAAATTGTAAGCGTTAATATTTTGTTAAAATTCGCGTTAAATTTTT
GTTAAATCAGCTCATTTTTTAACCAATAGGCCGAAATCGGCAAAATCCCTTATAAATCAAAAGAATAGACCGAGATAGGGTTGAGT
GTTGTTCCAGTTTGGAACAAGAGTCCACTATTAAAGAACGTGGACTCCAACGTCAAAGGGCGAAAAACCGTCTATCAGGGCGAT
GGCCCACTACGTGAACCATCACCCTAATCAAGTTTTTTGGGGTCGAGGTGCCGTAAAGCACTAAATCGGAACCCTAAAGGGAGC
CCCCGATTTAGAGCTTGACGGGGAAAGCCGGCGAACGTGGCGAGAAAGGAAGGGAAGAAAGCGAAAGGAGCGGGCGCTAGGG
CGCTGGCAAGTGTAGCGGTCACGCTGCGCGTAACCACCACACCCGCCGCGCTTAATGCGCCGCTACAGGGCGCGTCCCATTC
GCCATTCAGGCTGCGCAACTGTTGGGAAGGGCGATCGGTGCGGGCCTCTTCGCTATTACGCCAGCTGGCGAAAGGGGGATGT
GCTGCAAGGCGATTAAGTTGGGTAACGCCAGGGTTTTCCCAGTCACGACGTTGTAAAACGACGGCCAGTGAGCGCGCCTCGTT
CATTCACGTTTTTGAACCCGTGGAGGACGGGCAGACTCGCGGTGCAAATGTGTTTTACAGCGTGATGGAGCAGATGAAGATGCT
CGACACGCTGCAGAACACGCAGCTAGATTAACCCTAGAAAGATAATCATATTGTGACGTACGTTAAAGATAATCATGCGTAAAATT
GACGCATGTGTTTTATCGGTCTGTATATCGAGGTTTATTTATTAATTTGAATAGATATTAAGTTTTATTATATTTACACTTACATACTA
ATAATAAATTCAACAAACAATTTATTTATGTTTATTTATTTATTAAAAAAAAACAAAAACTCAAAATTTCTTCTATAAAGTAACAAAACTT
TTATGAGGGACAGCCCCCCCCCAAAGCCCCCAGGGATGTAATTACGTCCCTCCCCCGCTAGGGGGCAGCAGCGAGCCGCCCG
GGGCTCCGCTCCGGTCCGGCGCTCCCCCCGCATCCCCGAGCCGGCAGCGTGCGGGGACAGCCCGGGCACGGGGAAGGTGG
CACGGGATCGCTTTCCTCTGAACGCTTCTCGCTGCTCTTTGAGCCTGCAGACACCTGGGGGGATACGGGGAAAAGGCCTAAAAA
CCGCACTTGTC**CGGAAACCCCGGGAATCTAACCCGGCTGAACGGATTCTAGAGTCCATTCGATCTACATGATCAGGTTTCC**GG
TGTTTCGTCCTTTCCACAAGATATATAAAGCCAAGAAATCGAAATACTTTCAAGTTACGGTAAGCATATGATAGTCCATTTTAAAAC
ATAATTTTAAAACTGCAAACTACCCAAGAAATTATTACTTTCTACGTCACGTATTTTGTACTAATATCTTTGTGTTTACAGTCAAATT
AATTCTAATTATCTCTCTAACAGCCTTGTATCGTATATGCAAATATGAAGGAATCATGGGAAATAGGCCCTCTTCCTGCCCAACCG
GTTGCGATCGCTCCGGTGCCCGTCAGTGGGCAGAGCGCACATCGCCCACAGTCCCCGAGAAGTTGGGGGGAGGGGTCGGCAA
TTGAACGGGTGCCTAGAGAAGGTGGCGCGGGGTAAACTGGGAAAGTGATGTCGTGTACTGGCTCCGCCTTTTTCCCGAGGGTG
GGGGAGAACCGTATATAAGTGCAGTAGTCGCCGTGAACGTTCTTTTTCGCAACGGGTTTGCCGCCAGAACACAGCTGAAGCTTC
GAGGGGCTCGCATCTCTCCTTCACGCGCCCGCCGCCCTACCTGAGGCCGCCATCCACGCCGGTTGAGTCGCGTTCTGCCGCCT
CCCGCCTGTGGTGCCTCCTGAACTGCGTCCGCCGTCTAGGTAAGTTTAAAGCTCAGGTCGAGACCGGGCCTTTGTCCGGCGCT
CCCTTGGAGCCTACCTAGACTCAGCCGGCTCTCCACGCTTTGCCTGACCCTGCTTGCTCAACTCTACGTCTTTGTTTCGTTTTCT
GTTCTGCGCCGTTACAGATCCAAGCTGTGACCGGCGCCTACTCTACAGATAGCGTTTAAACTTACGCTTGCCACC**ATGGCTAGC**
GACTACAAGGACGACGACGACAAGGACAAGAAGCCCCTGAACACCCTGATCAGCGCCACAGGACTGTGGATGTCCAGAACC

GGCACCATCCACAAGATCAAGCACCACGAGGTGTCCCGGTCCAAAATCTACATCGAGATGGCCTGCGGCGATCACCTGGTCG
TCAACAACAGCAGAAGCAGCCGGACAGCCAGAGCCCTGCGGCACCACAAGTACAGAAAGACCTGCAAGCGGTGCAGAGTGT
CCGACGAGGACCTGAACAAGTTCCTGACCAAGGCCAACGAGGACCAGACCAGCGTGAAAGTGAAGGTGGTGTCCGCCCCCA
CCCGGACCAAGAAAGCCATGCCCAAGAGCGTGGCCAGAGCCCCCAAGCCCCTGGAAAACACCGAAGCCGCTCAGGCCCAG
CCCAGCGGCAGCAAGTTCAGCCCCGCCATCCCCGTGTCTACCCAGGAAAGCGTCAGCGTCCCCGCCAGCGTGTCCACCAGC
ATCTCTAGCATCTCAACCGGCGCCACAGCTTCTGCCCTGGTCAAGGGCAACACCAACCCCATCACCAGCATGTCTGCCCCTGT
GCAAGCCTCTGCCCCAGCCCTGACCAAGTCCCAGACCGACCGGCTGGAAGTGCTCCTGAACCCCAAGGACGAGATCAGCCT
GAACAGCGGCAAGCCCTTCCGGGAGCTGGAAAGCGAGCTGCTGAGCCGGCGGAAGAAGGACCTCCAGCAAATCTACGCCG
AGGAACGGGAGAACTACCTGGGCAAGCTGGAAAGAGAGATCACCCGGTTCTTCGTGGACCGGGGCTTCCTGGAAATCAAGA
GCCCCATCCTGATCCCCCTGGAGTACATCGAGCGGATGGGCATCGACAACGACACCGAGCTGAGCAAGCAGATTTTCCGGGT
GGACAAGAACTTCTGCCTGCGGCCCATGCTGGCCCCCAACCTGTACAACTACCTGCGGAAACTGGATCGCGCTCTGCCCGAC
CCCATCAAGATTTTCGAGATCGGCCCCTGCTACCGGAAAGAGAGCGACGGCAAAGAGCACCTGGAAGAGTTTACAATGCTGA
ACTTTTGCCAGATGGGCAGCGGCTGCACCAGAGAGAACCTGGAATCCATCATCACCGACTTTCTGAACCACCTGGGGATCGA
CTTCAAGATCGTGGGCGACAGCTGCATGGTGTACGGCGACACCCTGGACGTGATGCACGGCGACCTGGAACTGTCTAGCGC
CGTCGTGGGACCCATCCCTCTGGACCGGGAGTGGGGCATCGATAAGCCCTGGATCGGAGCCGGCTTCGGCCTGGAACGGCT
GCTGAAAGTCAAGCACGACTTTAAGAACATCAAGCGGGCTGCCAGAAGCGAGAGCTACTACAACGGCATCAGCACCAACCT
GTGATGATAAGGATCCACTAGTCCAGTGTGGTGGAATTGACATTGATTATTGACTAGTTATTAATAGTAATCAATTACGGGGTCAT
TAGTTCATAGCCCATATATGGAGTTCCGCGTTACATAACTTACGGTAAATGGCCCGCCTGGCTGACCGCCCAACGACCCCCGCC
CATTGACGTCAATAATGACGTATGTTCCCATAGTAACGCCAATAGGGACTTTCCATTGACGTCAATGGGTGGAGTATTTACGGTA
AACTGCCCACTTGGCAGTACATCAAGTGTATCATATGCCAAGTACGCCCCCTATTGACGTCAATGACGGTAAATGGCCCGCCTG
GCATTATGCCCAGTACATGACCTTATGGGACTTTCCTACTTGGCAGTACATCTACGTATTAGTCATCGCTATTACCATGGTCGAGG
TGAGCCCCACGTTCTGCTTCACTCTCCCCATCTCCCCCCCCTCCCCACCCCCAATTTTGTATTTATTTATTTTTTAATTATTTTGTG
CAGCGATGGGGGCGGGGGGGGGGGGGGCGCGCGCCAGGCGGGGCGGGGCGGGGCGAGGGGCGGGGCGGGGCGAGGCG
GAGAGGTGCGGCGGCAGCCAATCAGAGCGGCGCGCTCCGAAAGTTTCCTTTTATGGCGAGGCGGCGGCGGCGGCGGCCCTAT
AAAAAGCGAAGCGCGCGGCGGGCGGGAGTCGCTGCGTTGCCTTCGCCCCGTGCCCCGCTCCGCGCCGCCTCGCGCCGCCCG
CCCCGGCTCTGACTGACCGCGTTACTCCCACAGGTGAGCGGGCGGGACGGCCCTTCTCCTCCGGGCTGTAATTAGCGCTTGGT
TTAATGACGGCTCGTTTCTTTTCTGTGGCTGCGTGAAAGCCTTAAAGGGCTCCGGGAGGGCCCTTTGTGCGGGGGGGAGCGGC
TCGGGGGGTGCGTGCGTGTGTGTGTGCGTGGGGAGCGCCGCGTGCGGCCCGCGCTGCCCGGCGGCTGTGAGCGCTGCGGG
CGCGGCGCGGGGCTTTGTGCGCTCCGCGTGTGCGCGAGGGGAGCGCGGCCGGGGGCGGTGCCCCGCGGTGCGGGGGGGCT
GCGAGGGGAACAAAGGCTGCGTGCGGGGTGTGTGCGTGGGGGGGTGAGCAGGGGGTGTGGGCGCGGCGGTCGGGCTGTAA
CCCCCCCCTGCACCCCCCTCCCCGAGTTGCTGAGCACGGCCCGGCTTCGGGTGCGGGGCTCCGTACGGGGCGTGGCGCGGG
GCTCGCCGTGCCGGGCGGGGGGTGGCGGCAGGTGGGGGTGCCGGGCGGGGCGGGGCCGCCTCGGGCCGGGGAGGGCTCG
GGGGAGGGGCGCGGCGGCCCCCGGAGCGCCGGCGGCTGTCGAGGCGCGGCGAGCCGCAGCCATTGCCTTTTATGGTAATCG
TGCGAGAGGGCGCAGGGACTTCCTTTGTCCCAAATCTGTGCGGAGCCGAAATCTGGGAGGCGCCGCCGCACCCCCTCTAGCG
GGCGCGGGGCGAAGCGGTGCGGCGCCGGCAGGAAGGAAATGGGCGGGGAGGGCCTTCGTGCGTCGCCGCGCCGCCGTCCC
CTTCTCCCTCTCCAGCCTCGGGGCTGTCCGCGGGGGGACGGCTGCCTTCGGGGGGGACGGGGCAGGGCGGGGTTCGGCTTC
TGGCGTGTGACCGGCGGCTCTAGAGCCTCTGCTAACCATGTTCATGCCTTCTTCTTTTTCCTACAGCTCCTGGGCAACGTGCTG
GTTATTGTGCTGTCTCATCATTTTGGCAAAGAATTCGCCACCATGGTGAGCAAGGGCGAGGAGGATAACATGGCCATCATCAA
GGAGTTCATGCGCTTCAAGGTGCACATGGAGGGCTCCGTGAACGGCCACGAGTTCGAGATCGAGGGCGAGGGCGAGGGCC
GCCCCTACGAGGGCACCCAGACCGCCAAGCTGAAGGTGACCAAGGGTGGCCCCCTGCCCTTCGCCTGGGACATCCTGTCCC
CTCAGTTCATGTACGGCTCCAAGGCCTACGTGAAGCACCCCGCCGACATCCCCGACTACTTGAAGCTGTCCTTCCCCGAGGG
CTTCAAGTGGGAGCGCGTGATGAACTTCGAGGACGGCGGCGTGGTGACCGTGACCCAGGACTCCTCCCTGCAGGACGGCGA
GTTCATCTACAAGGTGAAGCTGCGCGGCACCAACTTCCCCTCCGACGGCCCCGTAATGCAGAAGAAGACCATGGGCTGGGA
GGCCTCCTCCGAGCGGATGTACCCCGAGGACGGCGCCCTGAAGGGCGAGATCAAGCAGAGGCTGAAGCTGAAGGACGGCG
GCCACTACGACGCTGAGGTCAAGACCACCTACAAGGCCAAGAAGCCCGTGCAGCTGCCCGGCGCCTACAACGTCAACATCA
AGTTGGACATCACCTCCCACAACGAGGACTACACCATCGTGGAACAGTACGAACGCGCCGAGGGCCGCCACTCCACCGGCG
GCATGGACGAGCTGTACGCGTCTGGAGGCGCCACCAATTTCAGCCTGCTGAAACAGGCTGGCGACGTGGAAGAGAACCCTG
GACCTGGACAAAAGTCGGTGAGCAAGGGCGAGGAGCTGTTCACCGGGGTGGTGCCCATCCTGGTCGAGCTGGACGGCGACG
TAAACGGCCACAAGTTCAGCGTGTCCGGCGAGGGCGAGGGCGATGCCACCTACGGCAAGCTGACCCTGAAGTTCATCTGCA
CCACCGGCAAGCTGCCCGTGCCCTGGCCCACCCTCGTGACCACCCTGACCTACGGCGTGCAGTGCTTCAGCCGCTACCCCGA
CCACATGAAGCAGCACGACTTCTTCAAGTCCGCCATGCCCGAAGGCTACGTCCAGGAGCGCACCATCTTCTTCAAGGACGAC
GGCAACTACAAGACCCGCGCCGAGGTGAAGTTCGAGGGCGACACCCTGGTGAACCGCATCGAGCTGAAGGGCATCGACTTC
AAGGAGGACGGCAACATCCTGGGGCACAAGCTGGAGTACAACTACAACAGCCACCTAGGTCTATATCATGGCCGACAAGCA
GAAGAACGGCATCAAGGTGAACTTCAAGATCCGCCACAACATCGAGGACGGCAGCGTGCAGCTCGCCGACCACTACCAGCA
GAACACCCCCATCGGCGACGGCCCCGTGCTGCTGCCCGACAACCACTACCTGAGCACCCAGTCCGCCCTGAGCAAAGACCC
CAACGAGAAGCGCGATCACATGGTCCTGCTGGAGTTCGTGACCGCCGCCGGGATCACTCTCGGCATGGACGAGCTGTACAA
GTAAGAATTCACTCCTCAGGTGCAGGCTGCCTATCAGAAGGTGGTGGCTGGTGTGGCCAATGCCCTGGCTCACAAATACCACTG
AGATCTTTTTCCCTCTGCCAAAAATTATGGGGACATCATGAAGCCCCTTGAGCATCTGACTTCTGGCTAATAAAGGAAATTTATTT
TCATTGCAATAGTGTGTTGGAATTTTTTGTGTCTCTCACTCGGAAGGACATATGGGAGGGGTCGACAATCAACCTCTGGATTACA
AAATTTGTGAAAGATTGACTGGTATTCTTAACTATGTTGCTCCTTTTACGCTATGTGGATACGCTGCTTTAATGCCTTTGTATCATG
CGTTAACTAAACTTGTTTATTGCAGCTTATAATGGTTACAAATAAAGCAATAGCATCACAAATTTCACAAATAAAGCATTTTTTTCAC
TGCATTCTAGTTGTGGTTTGTCCAAACTCATCAATGTATCTTATCATGTCTGGAATTGACTCAAATGATGTCAATTAGTCTATCAGA
AGCTATCTGGTCTCCCTTCCGGGGGACAAGACATCCCTGTTTAATATTTAAACAGCAGTGTTCCCAAACTGGGTTCTTATATCCCT
TGCTCTGGTCAACCAGGTTGCAGGGTTTCCTGTCCTCACAGGAACGAAGTCCCTAAAGAAACAGTGGCAGCCAGGTTTAGCCCC
GGAATTGACTGGATTCCTTTTTTAGGGCCCATTGGTATGGCTTTTTCCCCGTATCCCCCCAGGTGTCTGCAGGCTCAAAGAGCAG
CGAGAAGCGTTCAGAGGAAAGCGATCCCGTGCCACCTTCCCCGTGCCCGGGCTGTCCCCGCACGCTGCCGGCTCGGGGATGC
GGGGGGAGCGCCGGACCGGAGCGGAGCCCCGGGCGGCTCGCTGCTGCCCCCTAGCGGGGGAGGGACGTAATTACATCCCTG
GGGGCTTTGGGGGGGGGCTGTCCCTGATATCTATAACAAGAAAATATATATATAATAAGTTATCACGTAAGTAGAACATGAAATAA
CAATATAATTATCGTATGAGTTAAATCTTAAAAGTCACGTAAAAGATAATCATGCGTCATTTTGACTCACGCGGTCGTTATAGTTCA
AAATCAGTGACACTTACCGCATTGACAAGCACGCCTCACGGGAGCTCCAAGCGGCGACTGAGATGTCCTAAATGCACAGCGACG
GATTCGCGCTATTTAGAAAGAGAGAGCAATATTTCAAGAATGCATGCGTCAATTTTACGCAGACTATCTTTCTAGGGTTAATCTAG
CTGCATCAGGATCATATCGTCGGGTCTTTTTTCCGGCTCAGTCATCGCCCAAGCTGGCGCTATCTGGGCATCGGGGAGGAAGAA
GCCCGTGCCTTTTCCCGCGAGGTTGAAGCGGCATGGAAAGAGTTTGCCGAGGATGACTGCTGCTGCATTGACGTTGAGCGAAA
ACGCACGTTTACCATGATGATTCGGGAAGGTGTGGCCATGCACGCCTTTAACGGTGAACTGTTCGTTCAGGCCACCTGGGATAC

CAGTTCGTCGCGGCTTTTCCGGACACAGTTCCGGATGGTCAGCCCGAAGCGCATCAGCAACCCGAACAATACCGGCGACAGCC
GGAACTGCCGTGCCGGTGTGCAGATTAATGACAGCGGTGCGGCGCTGGGATATTACGTCAGCGAGGACGGGTATCCTGGCTG
GATGCCGCAGAAATGGACATGGATACCCCGTGAGTTACCCGGCGGGCGCGCTTGGCGTAATCATGGTCATAGCTGTTTCCTGT
GTGAAATTGTTATCCGCTCACAATTCCACACAACATACGAGCCGGAAGCATAAAGTGTAAAGCCTGGGGTGCCTAATGAGTGAG
CTAACTCACATTAATTGCGTTGCGCTCACTGCCCGCTTTCCAGTCGGGAAACCTGTCGTGCCAGCTGCATTAATGAATCGGCCAA
CGCGCGGGGAGAGGCGGTTTGCGTATTGGGCGCTCTTCCGCTTCCTCGCTCACTGACTCGCTGCGCTCGGTCGTTCGGCTGCG
GCGAGCGGTATCAGCTCACTCAAAGGCGGTAATACGGTTATCCACAGAATCAGGGGATAACGCAGGAAAGAACATGTGAGCAAA
AGGCCAGCAAAAGGCCAGGAACCGTAAAAAGGCCGCGTTGCTGGCGTTTTTCCATAGGCTCCGCCCCCCTGACGAGCATCACA
AAAATCGACGCTCAAGTCAGAGGTGGCGAAACCCGACAGGACTATAAAGATACCAGGCGTTTCCCCCTGGAAGCTCCCTCGTGC
GCTCTCCTGTTCCGACCCTGCCGCTTACCGGATACCTGTCCGCCTTTCTCCCTTCGGGAAGCGTGGCGCTTTCTCATAGCTCAC
GCTGTAGGTATCTCAGTTCGGTGTAGGTCGTTCGCTCCAAGCTGGGCTGTGTGCACGAACCCCCCGTTCAGCCCGACCGCTGC
GCCTTATCCGGTAACTATCGTCTTGAGTCCAACCCGGTAAGACACGACTTATCGCCACTGGCAGCAGCCACTGGTAACAGGATT
AGCAGAGCGAGGTATGTAGGCGGTGCTACAGAGTTCTTGAAGTGGTGGCCTAACTACGGCTACACTAGAAGGACAGTATTTGGT
ATCTGCGCTCTGCTGAAGCCAGTTACCTTCGGAAAAAGAGTTGGTAGCTCTTGATCCGGCAAACAAACCACCGCTGGTAGCGGT
GGTTTTTTTGTTTGCAAGCAGCAGATTACGCGCAGAAAAAAAGGATCTCAAGAAGATCCTTTGATCTTTTCTACGGGGTCTGACG
CTCAGTGGAACGAAAACTCACGTTAAGGGATTTTGGTCATGAGATTATCAAAAAGGATCTTCACCTAGATCCTTTTAAATTAAAAAT
GAAGTTTTAAATCAATCTAAAGTATATATGAGTAAACTTGGTCTGACAGTTACCAATGCTTAATCAGTGAGGCACCTATCTCAGCG
ATCTGTCTATTTCGTTCATCCATAGTTGCCTGACTCCCCGTCGTGTAGATAACTACGATACGGGAGGGCTTACCATCTGGCCCCA
GTGCTGCAATGATACCGCGAGACCCACGCTCACCGGCTCCAGATTTATCAGCAATAAACCAGCCAGCCGGAAGGGCCGAGCGC
AGAAGTGGTCCTGCAACTTTATCCGCCTCCATCCAGTCTATTAATTGTTGCCGGGAAGCTAGAGTAAGTAGTTCGCCAGTTAATA
GTTTGCGCAACGTTGTTGCCATTGCTACAGGCATCGTGGTGTCACGCTCGTCGTTTGGTATGGCTTCATTCAGCTCCGGTTCCCA
ACGATCAAGGCGAGTTACATGATCCCCCATGTTGTGCAAAAAAGCGGTTAGCTCCTTCGGTCCTCCGATCGTTGTCAGAAGTAAG
TTGGCCGCAGTGTTATCACTCATGGTTATGGCAGCACTGCATAATTCTCTTACTGTCATGCCATCCGTAAGATGCTTTTCTGTGAC
TGGTGAGTACTCAACCAAGTCATTCTGAGAATAGTGTATGCGGCGACCGAGTTGCTCTTGCCCGGCGTCAATACGGGATAATAC
CGCGCCACATAGCAGAACTTTAAAAGTGCTCATCATTGGAAAACGTTCTTCGGGGCGAAAACTCTCAAGGATCTTACCGCTGTTG
AGATCCAGTTCGATGTAACCCACTCGTGCACCCAACTGATCTTCAGCATCTTTTACTTTCACCAGCGTTTCTGGGTGAGCAAAAA
CAGGAAGGCAAAATGCCGCAAAAAAGGGAATAAGGGCGACACGGAAATGTTGAATACTCAT

2.2.10 Vector 63: Pyl $tRNA_{CUAG(Ev1)}$ PylRS mCherry-P2A-eGFP(150CTAG)

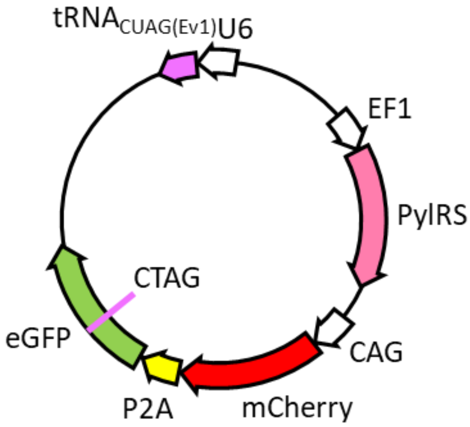

ACTCTTCCTTTTTCAATATTATTGAAGCATTTATCAGGGTTATTGTCTCATGAGCGGATACATATTTGAATGTATTTAGAAAAATAAA
CAAATAGGGGTTCCGCGCACATTTCCCCGAAAAGTGCCACCTAAATTGTAAGCGTTAATATTTTGTTAAAATTCGCGTTAAATTTTT
GTTAAATCAGCTCATTTTTTAACCAATAGGCCGAAATCGGCAAAATCCCTTATAAATCAAAAGAATAGACCGAGATAGGGTTGAGT
GTTGTTCCAGTTTGGAACAAGAGTCCACTATTAAAGAACGTGGACTCCAACGTCAAAGGGCGAAAAACCGTCTATCAGGGCGAT
GGCCCACTACGTGAACCATCACCCTAATCAAGTTTTTTGGGGTCGAGGTGCCGTAAAGCACTAAATCGGAACCCTAAAGGGAGC
CCCCGATTTAGAGCTTGACGGGGAAAGCCGGCGAACGTGGCGAGAAAGGAAGGGAAGAAAGCGAAAGGAGCGGGCGCTAGGG
CGCTGGCAAGTGTAGCGGTCACGCTGCGCGTAACCACCACACCCGCCGCGCTTAATGCGCCGCTACAGGGCGCGTCCCATTC
GCCATTCAGGCTGCGCAACTGTTGGGAAGGGCGATCGGTGCGGGCCTCTTCGCTATTACGCCAGCTGGCGAAAGGGGGATGT
GCTGCAAGGCGATTAAGTTGGGTAACGCCAGGGTTTTCCCAGTCACGACGTTGTAAAACGACGGCCAGTGAGCGCGCCTCGTT
CATTCACGTTTTTGAACCCGTGGAGGACGGGCAGACTCGCGGTGCAAATGTGTTTTACAGCGTGATGGAGCAGATGAAGATGCT
CGACACGCTGCAGAACACGCAGCTAGATTAACCCTAGAAAGATAATCATATTGTGACGTACGTTAAAGATAATCATGCGTAAAATT
GACGCATGTGTTTTATCGGTCTGTATATCGAGGTTTATTTATTAATTTGAATAGATATTAAGTTTTATTATATTTACACTTACATACTA
ATAATAAATTCAACAAACAATTTATTTATGTTTATTTATTTATTAAAAAAAAACAAAAACTCAAAATTTCTTCTATAAAGTAACAAAACTT
TTATGAGGGACAGCCCCCCCCCAAAGCCCCCAGGGATGTAATTACGTCCCTCCCCCGCTAGGGGGCAGCAGCGAGCCGCCCG
GGGCTCCGCTCCGGTCCGGCGCTCCCCCCGCATCCCCGAGCCGGCAGCGTGCGGGGACAGCCCGGGCACGGGGAAGGTGG
CACGGGATCGCTTTCCTCTGAACGCTTCTCGCTGCTCTTTGAGCCTGCAGACACCTGGGGGGATACGGGGAAAAGGCCTAAAAA
CCGCACTTGTC**CGGAAACCCCGGGAATCTAACCCGGCTGAACGGGTTCTAGAGGCCATTCGATCTACATGATCAGGTTTCC**G
GTGTTTCGTCCTTTCCACAAGATATATAAAGCCAAGAAATCGAAATACTTTCAAGTTACGGTAAGCATATGATAGTCCATTTTAAAA
CATAATTTTAAAACTGCAAACTACCCAAGAAATTATTACTTTCTACGTCACGTATTTTGTACTAATATCTTTGTGTTTACAGTCAAAT
TAATTCTAATTATCTCTCTAACAGCCTTGTATCGTATATGCAAATATGAAGGAATCATGGGAAATAGGCCCTCTTCCTGCCCAACC
GGTTGCGATCGCTCCGGTGCCCGTCAGTGGGCAGAGCGCACATCGCCCACAGTCCCCGAGAAGTTGGGGGGAGGGGTCGGCA
ATTGAACGGGTGCCTAGAGAAGGTGGCGCGGGGTAAACTGGGAAAGTGATGTCGTGTACTGGCTCCGCCTTTTTCCCGAGGGT
GGGGGAGAACCGTATATAAGTGCAGTAGTCGCCGTGAACGTTCTTTTTCGCAACGGGTTTGCCGCCAGAACACAGCTGAAGCTT
CGAGGGGCTCGCATCTCTCCTTCACGCGCCCGCCGCCCTACCTGAGGCCGCCATCCACGCCGGTTGAGTCGCGTTCTGCCGC
CTCCCGCCTGTGGTGCCTCCTGAACTGCGTCCGCCGTCTAGGTAAGTTTAAAGCTCAGGTCGAGACCGGGCCTTTGTCCGGCG
CTCCCTTGGAGCCTACCTAGACTCAGCCGGCTCTCCACGCTTTGCCTGACCCTGCTTGCTCAACTCTACGTCTTTGTTTCGTTTT
CTGTTCTGCGCCGTTACAGATCCAAGCTGTGACCGGCGCCTACTCTACAGATAGCGTTTAAACTTACGCTTGCCACC**ATGGCTA**

GCGACTACAAGGACGACGACGACAAGGACAAGAAGCCCCTGAACACCCTGATCAGCGCCACAGGACTGTGGATGTCCAGAA
CCGGCACCATCCACAAGATCAAGCACCACGAGGTGTCCCGGTCCAAAATCTACATCGAGATGGCCTGCGGCGATCACCTGGT
CGTCAACAACAGCAGAAGCAGCCGGACAGCCAGAGCCCTGCGGCACCACAAGTACAGAAAGACCTGCAAGCGGTGCAGAG
TGTCCGACGAGGACCTGAACAAGTTCCTGACCAAGGCCAACGAGGACCAGACCAGCGTGAAAGTGAAGGTGGTGTCCGCCC
CCACCCGGACCAAGAAAGCCATGCCCAAGAGCGTGGCCAGAGCCCCCAAGCCCCTGGAAAACACCGAAGCCGCTCAGGCC
CAGCCCAGCGGCAGCAAGTTCAGCCCCGCCATCCCCGTGTCTACCCAGGAAAGCGTCAGCGTCCCCGCCAGCGTGTCCACC
AGCATCTCTAGCATCTCAACCGGCGCCACAGCTTCTGCCCTGGTCAAGGGCAACACCAACCCCATCACCAGCATGTCTGCCC
CTGTGCAAGCCTCTGCCCCAGCCCTGACCAAGTCCCAGACCGACCGGCTGGAAGTGCTCCTGAACCCCAAGGACGAGATCA
GCCTGAACAGCGGCAAGCCCTTCCGGGAGCTGGAAAGCGAGCTGCTGAGCCGGCGGAAGAAGGACCTCCAGCAAATCTACG
CCGAGGAACGGGAGAACTACCTGGGCAAGCTGGAAAGAGAGATCACCCGGTTCTTCGTGGACCGGGGCTTCCTGGAAATCA
AGAGCCCCATCCTGATCCCCCTGGAGTACATCGAGCGGATGGGCATCGACAACGACACCGAGCTGAGCAAGCAGATTTTCCG
GGTGGACAAGAACTTCTGCCTGCGGCCCATGCTGGCCCCCAACCTGTACAACTACCTGCGGAAACTGGATCGCGCTCTGCCC
GACCCCATCAAGATTTTCGAGATCGGCCCCTGCTACCGGAAAGAGAGCGACGGCAAAGAGCACCTGGAAGAGTTTACAATGC
TGAACTTTTGCCAGATGGGCAGCGGCTGCACCAGAGAGAACCTGGAATCCATCATCACCGACTTTCTGAACCACCTGGGGAT
CGACTTCAAGATCGTGGGCGACAGCTGCATGGTGTACGGCGACACCCTGGACGTGATGCACGGCGACCTGGAACTGTCTAG
CGCCGTCGTGGGACCCATCCCTCTGGACCGGGAGTGGGGCATCGATAAGCCCTGGATCGGAGCCGGCTTCGGCCTGGAACG
GCTGCTGAAAGTCAAGCACGACTTTAAGAACATCAAGCGGGCTGCCAGAAGCGAGAGCTACTACAACGGCATCAGCACCAA
CCTGTGATGATAAGGATCCACTAGTCCAGTGTGGTGGAATTGACATTGATTATTGACTAGTTATTAATAGTAATCAATTACGGGGT
CATTAGTTCATAGCCCATATATGGAGTTCCGCGTTACATAACTTACGGTAAATGGCCCGCCTGGCTGACCGCCCAACGACCCCC
GCCCATTGACGTCAATAATGACGTATGTTCCCATAGTAACGCCAATAGGGACTTTCCATTGACGTCAATGGGTGGAGTATTTACG
GTAAACTGCCCACTTGGCAGTACATCAAGTGTATCATATGCCAAGTACGCCCCCTATTGACGTCAATGACGGTAAATGGCCCGCC
TGGCATTATGCCCAGTACATGACCTTATGGGACTTTCCTACTTGGCAGTACATCTACGTATTAGTCATCGCTATTACCATGGTCGA
GGTGAGCCCCACGTTCTGCTTCACTCTCCCCATCTCCCCCCCCTCCCCACCCCCAATTTTGTATTTATTTATTTTTTAATTATTTTG
TGCAGCGATGGGGGCGGGGGGGGGGGGGGGCGCGCGCCAGGCGGGGCGGGGCGGGGCGAGGGGCGGGGCGGGGCGAGGC
GGAGAGGTGCGGCGGCAGCCAATCAGAGCGGCGCGCTCCGAAAGTTTCCTTTTATGGCGAGGCGGCGGCGGCGGCGGCCCT
ATAAAAAGCGAAGCGCGCGGCGGGCGGGAGTCGCTGCGTTGCCTTCGCCCCGTGCCCCGCTCCGCGCCGCCTCGCGCCGCC
CGCCCCGGCTCTGACTGACCGCGTTACTCCCACAGGTGAGCGGGCGGGACGGCCCTTCTCCTCCGGGCTGTAATTAGCGCTTG
GTTTAATGACGGCTCGTTTCTTTTCTGTGGCTGCGTGAAAGCCTTAAAGGGCTCCGGGAGGGCCCTTTGTGCGGGGGGGAGCG
GCTCGGGGGGTGCGTGCGTGTGTGTGTGCGTGGGGAGCGCCGCGTGCGGCCCGCGCTGCCCGGCGGCTGTGAGCGCTGCG
GGCGCGGCGCGGGGCTTTGTGCGCTCCGCGTGTGCGCGAGGGGAGCGCGGCCGGGGGCGGTGCCCCGCGGTGCGGGGGG
GCTGCGAGGGGAACAAAGGCTGCGTGCGGGGTGTGTGCGTGGGGGGGTGAGCAGGGGGTGTGGGCGCGGCGGTCGGGCTG
TAACCCCCCCCTGCACCCCCCTCCCCGAGTTGCTGAGCACGGCCCGGCTTCGGGTGCGGGGCTCCGTACGGGGCGTGGCGC
GGGGCTCGCCGTGCCGGGCGGGGGGTGGCGGCAGGTGGGGGTGCCGGGCGGGGCGGGGCCGCCTCGGGCCGGGGAGGG
CTCGGGGGAGGGGCGCGGCGGCCCCCGGAGCGCCGGCGGCTGTCGAGGCGCGGCGAGCCGCAGCCATTGCCTTTTATGGTA
ATCGTGCGAGAGGGCGCAGGGACTTCCTTTGTCCCAAATCTGTGCGGAGCCGAAATCTGGGAGGCGCCGCCGCACCCCCTCTA
GCGGGCGCGGGGCGAAGCGGTGCGGCGCCGGCAGGAAGGAAATGGGCGGGGAGGGCCTTCGTGCGTCGCCGCGCCGCCGT
CCCCTTCTCCCTCTCCAGCCTCGGGGCTGTCCGCGGGGGGACGGCTGCCTTCGGGGGGGACGGGGCAGGGCGGGGTTCGGC
TTCTGGCGTGTGACCGGCGGCTCTAGAGCCTCTGCTAACCATGTTCATGCCTTCTTCTTTTTCCTACAGCTCCTGGGCAACGTGC
TGGTTATTGTGCTGTCTCATCATTTTGGCAAAGAATTCGCCACCATGGTGAGCAAGGGCGAGGAGGATAACATGGCCATCATCA
AGGAGTTCATGCGCTTCAAGGTGCACATGGAGGGCTCCGTGAACGGCCACGAGTTCGAGATCGAGGGCGAGGGCGAGGGCC
GCCCCTACGAGGGCACCCAGACCGCCAAGCTGAAGGTGACCAAGGGTGGCCCCCTGCCCTTCGCCTGGGACATCCTGTCCC
CTCAGTTCATGTACGGCTCCAAGGCCTACGTGAAGCACCCCGCCGACATCCCCGACTACTTGAAGCTGTCCTTCCCCGAGGG
CTTCAAGTGGGAGCGCGTGATGAACTTCGAGGACGGCGGCGTGGTGACCGTGACCCAGGACTCCTCCCTGCAGGACGGCGA
GTTCATCTACAAGGTGAAGCTGCGCGGCACCAACTTCCCCTCCGACGGCCCCGTAATGCAGAAGAAGACCATGGGCTGGGA
GGCCTCCTCCGAGCGGATGTACCCCGAGGACGGCGCCCTGAAGGGCGAGATCAAGCAGAGGCTGAAGCTGAAGGACGGCG
GCCACTACGACGCTGAGGTCAAGACCACCTACAAGGCCAAGAAGCCCGTGCAGCTGCCCGGCGCCTACAACGTCAACATCA
AGTTGGACATCACCTCCCACAACGAGGACTACACCATCGTGGAACAGTACGAACGCGCCGAGGGCCGCCACTCCACCGGCG
GCATGGACGAGCTGTACGCGTCTGGAGGCGCCACCAATTTCAGCCTGCTGAAACAGGCTGGCGACGTGGAAGAGAACCCTG
GACCTGGACAAAAGTCGGTGAGCAAGGGCGAGGAGCTGTTCACCGGGGTGGTGCCCATCCTGGTCGAGCTGGACGGCGACG
TAAACGGCCACAAGTTCAGCGTGTCCGGCGAGGGCGAGGGCGATGCCACCTACGGCAAGCTGACCCTGAAGTTCATCTGCA
CCACCGGCAAGCTGCCCGTGCCCTGGCCCACCCTCGTGACCACCCTGACCTACGGCGTGCAGTGCTTCAGCCGCTACCCCGA
CCACATGAAGCAGCACGACTTCTTCAAGTCCGCCATGCCCGAAGGCTACGTCCAGGAGCGCACCATCTTCTTCAAGGACGAC
GGCAACTACAAGACCCGCGCCGAGGTGAAGTTCGAGGGCGACACCCTGGTGAACCGCATCGAGCTGAAGGGCATCGACTTC
AAGGAGGACGGCAACATCCTGGGGCACAAGCTGGAGTACAACTACAACAGCCACCTAGGTCTATATCATGGCCGACAAGCA
GAAGAACGGCATCAAGGTGAACTTCAAGATCCGCCACAACATCGAGGACGGCAGCGTGCAGCTCGCCGACCACTACCAGCA
GAACACCCCCATCGGCGACGGCCCCGTGCTGCTGCCCGACAACCACTACCTGAGCACCCAGTCCGCCCTGAGCAAAGACCC
CAACGAGAAGCGCGATCACATGGTCCTGCTGGAGTTCGTGACCGCCGCCGGGATCACTCTCGGCATGGACGAGCTGTACAA
GTAAGAATTCACTCCTCAGGTGCAGGCTGCCTATCAGAAGGTGGTGGCTGGTGTGGCCAATGCCCTGGCTCACAAATACCACTG
AGATCTTTTTCCCTCTGCCAAAAATTATGGGGACATCATGAAGCCCCTTGAGCATCTGACTTCTGGCTAATAAAGGAAATTTATTT
TCATTGCAATAGTGTGTTGGAATTTTTTGTGTCTCTCACTCGGAAGGACATATGGGAGGGGTCGACAATCAACCTCTGGATTACA
AAATTTGTGAAAGATTGACTGGTATTCTTAACTATGTTGCTCCTTTTACGCTATGTGGATACGCTGCTTTAATGCCTTTGTATCATG
CGTTAACTAAACTTGTTTATTGCAGCTTATAATGGTTACAAATAAAGCAATAGCATCACAAATTTCACAAATAAAGCATTTTTTTCAC
TGCATTCTAGTTGTGGTTTGTCCAAACTCATCAATGTATCTTATCATGTCTGGAATTGACTCAAATGATGTCAATTAGTCTATCAGA
AGCTATCTGGTCTCCCTTCCGGGGGACAAGACATCCCTGTTTAATATTTAAACAGCAGTGTTCCCAAACTGGGTTCTTATATCCCT
TGCTCTGGTCAACCAGGTTGCAGGGTTTCCTGTCCTCACAGGAACGAAGTCCCTAAAGAAACAGTGGCAGCCAGGTTTAGCCCC
GGAATTGACTGGATTCCTTTTTTAGGGCCCATTGGTATGGCTTTTTCCCCGTATCCCCCCAGGTGTCTGCAGGCTCAAAGAGCAG
CGAGAAGCGTTCAGAGGAAAGCGATCCCGTGCCACCTTCCCCGTGCCCGGGCTGTCCCCGCACGCTGCCGGCTCGGGGATGC
GGGGGGAGCGCCGGACCGGAGCGGAGCCCCGGGCGGCTCGCTGCTGCCCCCTAGCGGGGGAGGGACGTAATTACATCCCTG
GGGGCTTTGGGGGGGGGCTGTCCCTGATATCTATAACAAGAAAATATATATATATAATAAGTTATCACGTAAGTAGAACATGAAATAA
CAATATAATTATCGTATGAGTTAAATCTTAAAAGTCACGTAAAAGATAATCATGCGTCATTTTGACTCACGCGGTCGTTATAGTTCA
AAATCAGTGACACTTACCGCATTGACAAGCACGCCTCACGGGAGCTCCAAGCGGCGACTGAGATGTCCTAAATGCACAGCGACG
GATTCGCGCTATTTAGAAAGAGAGAGCAATATTTCAAGAATGCATGCGTCAATTTTACGCAGACTATCTTTCTAGGGTTAATCTAG
CTGCATCAGGATCATATCGTCGGGTCTTTTTTCCGGCTCAGTCATCGCCCAAGCTGGCGCTATCTGGGCATCGGGGAGGAAGAA
GCCCGTGCCTTTTCCCGCGAGGTTGAAGCGGCATGGAAAGAGTTTGCCGAGGATGACTGCTGCTGCATTGACGTTGAGCGAAA

ACGCACGTTTACCATGATGATTCGGGAAGGTGTGGCCATGCACGCCTTTAACGGTGAACTGTTCGTTCAGGCCACCTGGGATAC
CAGTTCGTCGCGGCTTTTCCGGACACAGTTCCGGATGGTCAGCCCGAAGCGCATCAGCAACCCGAACAATACCGGCGACAGCC
GGAACTGCCGTGCCGGTGTGCAGATTAATGACAGCGGTGCGGCGCTGGGATATTACGTCAGCGAGGACGGGTATCCTGGCTG
GATGCCGCAGAAATGGACATGGATACCCCGTGAGTTACCCGGCGGGCGCGCTTGGCGTAATCATGGTCATAGCTGTTTCCTGT
GTGAAATTGTTATCCGCTCACAATTCCACACAACATACGAGCCGGAAGCATAAAGTGTAAAGCCTGGGGTGCCTAATGAGTGAG
CTAACTCACATTAATTGCGTTGCGCTCACTGCCCGCTTTCCAGTCGGGAAACCTGTCGTGCCAGCTGCATTAATGAATCGGCCAA
CGCGCGGGGAGAGGCGGTTTGCGTATTGGGCGCTCTTCCGCTTCCTCGCTCACTGACTCGCTGCGCTCGGTCGTTCGGCTGCG
GCGAGCGGTATCAGCTCACTCAAAGGCGGTAATACGGTTATCCACAGAATCAGGGGATAACGCAGGAAAGAACATGTGAGCAAA
AGGCCAGCAAAAGGCCAGGAACCGTAAAAAGGCCGCGTTGCTGGCGTTTTTCCATAGGCTCCGCCCCCCTGACGAGCATCACA
AAAATCGACGCTCAAGTCAGAGGTGGCGAAACCCGACAGGACTATAAAGATACCAGGCGTTTCCCCCTGGAAGCTCCCTCGTGC
GCTCTCCTGTTCCGACCCTGCCGCTTACCGGATACCTGTCCGCCTTTCTCCCTTCGGGAAGCGTGGCGCTTTCTCATAGCTCAC
GCTGTAGGTATCTCAGTTCGGTGTAGGTCGTTCGCTCCAAGCTGGGCTGTGTGCACGAACCCCCCGTTCAGCCCGACCGCTGC
GCCTTATCCGGTAACTATCGTCTTGAGTCCAACCCGGTAAGACACGACTTATCGCCACTGGCAGCAGCCACTGGTAACAGGATT
AGCAGAGCGAGGTATGTAGGCGGTGCTACAGAGTTCTTGAAGTGGTGGCCTAACTACGGCTACACTAGAAGGACAGTATTTGGT
ATCTGCGCTCTGCTGAAGCCAGTTACCTTCGGAAAAAGAGTTGGTAGCTCTTGATCCGGCAAACAAACCACCGCTGGTAGCGGT
GGTTTTTTTGTTTGCAAGCAGCAGATTACGCGCAGAAAAAAAGGATCTCAAGAAGATCCTTTGATCTTTTCTACGGGGTCTGACG
CTCAGTGGAACGAAAACTCACGTTAAGGGATTTTGGTCATGAGATTATCAAAAAGGATCTTCACCTAGATCCTTTTAAATTAAAAAT
GAAGTTTTAAATCAATCTAAAGTATATATGAGTAAACTTGGTCTGACAGTTACCAATGCTTAATCAGTGAGGCACCTATCTCAGCG
ATCTGTCTATTTCGTTCATCCATAGTTGCCTGACTCCCCGTCGTGTAGATAACTACGATACGGGAGGGCTTACCATCTGGCCCCA
GTGCTGCAATGATACCGCGAGACCCACGCTCACCGGCTCCAGATTTATCAGCAATAAACCAGCCAGCCGGAAGGGCCGAGCGC
AGAAGTGGTCCTGCAACTTTATCCGCCTCCATCCAGTCTATTAATTGTTGCCGGGAAGCTAGAGTAAGTAGTTCGCCAGTTAATA
GTTTGCGCAACGTTGTTGCCATTGCTACAGGCATCGTGGTGTCACGCTCGTCGTTTGGTATGGCTTCATTCAGCTCCGGTTCCCA
ACGATCAAGGCGAGTTACATGATCCCCCATGTTGTGCAAAAAAGCGGTTAGCTCCTTCGGTCCTCCGATCGTTGTCAGAAGTAAG
TTGGCCGCAGTGTTATCACTCATGGTTATGGCAGCACTGCATAATTCTCTTACTGTCATGCCATCCGTAAGATGCTTTTCTGTGAC
TGGTGAGTACTCAACCAAGTCATTCTGAGAATAGTGTATGCGGCGACCGAGTTGCTCTTGCCCGGCGTCAATACGGGATAATAC
CGCGCCACATAGCAGAACTTTAAAAGTGCTCATCATTGGAAAACGTTCTTCGGGGCGAAAACTCTCAAGGATCTTACCGCTGTTG
AGATCCAGTTCGATGTAACCCACTCGTGCACCCAACTGATCTTCAGCATCTTTTACTTTCACCAGCGTTTCTGGGTGAGCAAAAA
CAGGAAGGCAAAATGCCGCAAAAAAGGGAATAAGGGCGACACGGAAATGTTGAATACTCAT

2.2.11 Vector 64: Pyl tRNA$_{UCCU}$ PylRS mCherry-P2A-eGFP(150AGGA)

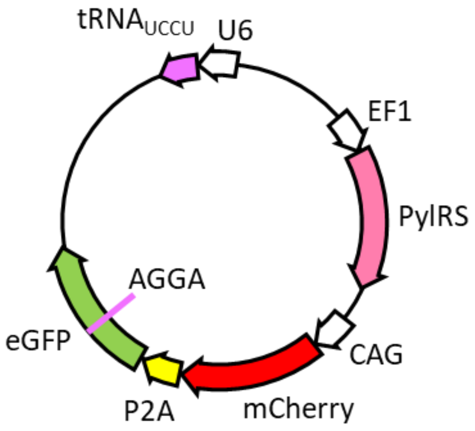

ACTCTTCCTTTTTCAATATTATTGAAGCATTTATCAGGGTTATTGTCTCATGAGCGGATACATATTTGAATGTATTTAGAAAAATAAA
CAAATAGGGGTTCCGCGCACATTTCCCCGAAAAGTGCCACCTAAATTGTAAGCGTTAATATTTTGTTAAAATTCGCGTTAAATTTTT
GTTAAATCAGCTCATTTTTTAACCAATAGGCCGAAATCGGCAAAATCCCTTATAAATCAAAAGAATAGACCGAGATAGGGTTGAGT
GTTGTTCCAGTTTGGAACAAGAGTCCACTATTAAAGAACGTGGACTCCAACGTCAAAGGGCGAAAAACCGTCTATCAGGGCGAT
GGCCCACTACGTGAACCATCACCCTAATCAAGTTTTTTGGGGTCGAGGTGCCGTAAAGCACTAAATCGGAACCCTAAAGGGAGC
CCCCGATTTAGAGCTTGACGGGGAAAGCCGGCGAACGTGGCGAGAAAGGAAGGGAAGAAAGCGAAAGGAGCGGGCGCTAGGG
CGCTGGCAAGTGTAGCGGTCACGCTGCGCGTAACCACCACACCCGCCGCGCTTAATGCGCCGCTACAGGGCGCGTCCCATTC
GCCATTCAGGCTGCGCAACTGTTGGGAAGGGCGATCGGTGCGGGCCTCTTCGCTATTACGCCAGCTGGCGAAAGGGGGATGT
GCTGCAAGGCGATTAAGTTGGGTAACGCCAGGGTTTTCCCAGTCACGACGTTGTAAAACGACGGCCAGTGAGCGCGCCTCGTT
CATTCACGTTTTTGAACCCGTGGAGGACGGGCAGACTCGCGGTGCAAATGTGTTTTACAGCGTGATGGAGCAGATGAAGATGCT
CGACACGCTGCAGAACACGCAGCTAGATTAACCCTAGAAAGATAATCATATTGTGACGTACGTTAAAGATAATCATGCGTAAAATT
GACGCATGTGTTTTATCGGTCTGTATATCGAGGTTTATTTATTAATTTGAATAGATATTAAGTTTTATTATATTTACACTTACATACTA
ATAATAAATTCAACAAACAATTTATTTATGTTTATTTATTTATTAAAAAAAAACAAAAACTCAAAATTTCTTCTATAAAGTAACAAAACTT
TTATGAGGGACAGCCCCCCCCCAAAGCCCCCAGGGATGTAATTACGTCCCTCCCCCGCTAGGGGGCAGCAGCGAGCCGCCCG
GGGCTCCGCTCCGGTCCGGCGCTCCCCCCGCATCCCCGAGCCGGCAGCGTGCGGGGACAGCCCGGGCACGGGGAAGGTGG
CACGGGATCGCTTTCCTCTGAACGCTTCTCGCTGCTCTTTGAGCCTGCAGACACCTGGGGGGATACGGGGAAAAGGCCTAAAAA
CCGCACTTGTC**CGGAAACCCCGGGAATCTAACCCGGCTGAACGGATTAGGAAGTCCATTCGATCTACATGATCAGGTTTCC**G
GTGTTTCGTCCTTTCCACAAGATATATAAAGCCAAGAAATCGAAATACTTTCAAGTTACGGTAAGCATATGATAGTCCATTTTAAAA
CATAATTTTAAAACTGCAAACTACCCAAGAAATTATTACTTTCTACGTCACGTATTTTGTACTAATATCTTTGTGTTTACAGTCAAAT
TAATTCTAATTATCTCTCTAACAGCCTTGTATCGTATATGCAAATATGAAGGAATCATGGGAAATAGGCCCTCTTCCTGCCCAACC
GGTTGCGATCGCTCCGGTGCCCGTCAGTGGGCAGAGCGCACATCGCCCACAGTCCCCGAGAAGTTGGGGGGAGGGGTCGGCA
ATTGAACGGGTGCCTAGAGAAGGTGGCGCGGGGTAAACTGGGAAAGTGATGTCGTGTACTGGCTCCGCCTTTTTCCCGAGGGT
GGGGGAGAACCGTATATAAGTGCAGTAGTCGCCGTGAACGTTCTTTTTCGCAACGGGTTTGCCGCCAGAACACAGCTGAAGCTT
CGAGGGGCTCGCATCTCTCCTTCACGCGCCCGCCGCCCTACCTGAGGCCGCCATCCACGCCGGTTGAGTCGCGTTCTGCCGC
CTCCCGCCTGTGGTGCCTCCTGAACTGCGTCCGCCGTCTAGGTAAGTTTAAAGCTCAGGTCGAGACCGGGCCTTTGTCCGGCG
CTCCCTTGGAGCCTACCTAGACTCAGCCGGCTCTCCACGCTTTGCCTGACCCTGCTTGCTCAACTCTACGTCTTTGTTTCGTTTT

CTGTTCTGCGCCGTTACAGATCCAAGCTGTGACCGGCGCCTACTCTACAGATAGCGTTTAAACTTACGCTTGCCACCATGGCTA
GCGACTACAAGGACGACGACGACAAGGACAAGAAGCCCCTGAACACCCTGATCAGCGCCACAGGACTGTGGATGTCCAGAA
CCGGCACCATCCACAAGATCAAGCACCACGAGGTGTCCCGGTCCAAAATCTACATCGAGATGGCCTGCGGCGATCACCTGGT
CGTCAACAACAGCAGAAGCAGCCGGACAGCCAGAGCCCTGCGGCACCACAAGTACAGAAAGACCTGCAAGCGGTGCAGAG
TGTCCGACGAGGACCTGAACAAGTTCCTGACCAAGGCCAACGAGGACCAGACCAGCGTGAAAGTGAAGGTGGTGTCCGCCC
CCACCCGGACCAAGAAAGCCATGCCCAAGAGCGTGGCCAGAGCCCCCAAGCCCCTGGAAAACACCGAAGCCGCTCAGGCC
CAGCCCAGCGGCAGCAAGTTCAGCCCCGCCATCCCCGTGTCTACCCAGGAAAGCGTCAGCGTCCCCGCCAGCGTGTCCACC
AGCATCTCTAGCATCTCAACCGGCGCCACAGCTTCTGCCCTGGTCAAGGGCAACACCAACCCCATCACCAGCATGTCTGCCC
CTGTGCAAGCCTCTGCCCCAGCCCTGACCAAGTCCCAGACCGACCGGCTGGAAGTGCTCCTGAACCCCAAGGACGAGATCA
GCCTGAACAGCGGCAAGCCCTTCCGGGAGCTGGAAAGCGAGCTGCTGAGCCGGCGGAAGAAGGACCTCCAGCAAATCTACG
CCGAGGAACGGGAGAACTACCTGGGCAAGCTGGAAAGAGAGATCACCCGGTTCTTCGTGGACCGGGGCTTCCTGGAAATCA
AGAGCCCCATCCTGATCCCCCTGGAGTACATCGAGCGGATGGGCATCGACAACGACACCGAGCTGAGCAAGCAGATTTTCCG
GGTGGACAAGAACTTCTGCCTGCGGCCCATGCTGGCCCCCAACCTGTACAACTACCTGCGGAAACTGGATCGCGCTCTGCCC
GACCCCATCAAGATTTTCGAGATCGGCCCCTGCTACCGGAAAGAGAGCGACGGCAAAGAGCACCTGGAAGAGTTTACAATGC
TGAACTTTTGCCAGATGGGCAGCGGCTGCACCAGAGAGAACCTGGAATCCATCATCACCGACTTTCTGAACCACCTGGGGAT
CGACTTCAAGATCGTGGGCGACAGCTGCATGGTGTACGGCGACACCCTGGACGTGATGCACGGCGACCTGGAACTGTCTAG
CGCCGTCGTGGGACCCATCCCTCTGGACCGGGAGTGGGGCATCGATAAGCCCTGGATCGGAGCCGGCTTCGGCCTGGAACG
GCTGCTGAAAGTCAAGCACGACTTTAAGAACATCAAGCGGGCTGCCAGAAGCGAGAGCTACTACAACGGCATCAGCACCAA
CCTGTGATGATAAGGATCCACTAGTCCAGTGTGGTGGAATTGACATTGATTATTGACTAGTTATTAATAGTAATCAATTACGGGGT
CATTAGTTCATAGCCCATATATGGAGTTCCGCGTTACATAACTTACGGTAAATGGCCCGCCTGGCTGACCGCCCAACGACCCCC
GCCCATTGACGTCAATAATGACGTATGTTCCCATAGTAACGCCAATAGGGACTTTCCATTGACGTCAATGGGTGGAGTATTTACG
GTAAACTGCCCACTTGGCAGTACATCAAGTGTATCATATGCCAAGTACGCCCCCTATTGACGTCAATGACGGTAAATGGCCCGCC
TGGCATTATGCCCAGTACATGACCTTATGGGACTTTCCTACTTGGCAGTACATCTACGTATTAGTCATCGCTATTACCATGGTCGA
GGTGAGCCCCACGTTCTGCTTCACTCTCCCCATCTCCCCCCCCTCCCCACCCCCAATTTTGTATTTATTTATTTTTTAATTATTTTG
TGCAGCGATGGGGGCGGGGGGGGGGGGGGGCGCGCGCCAGGCGGGGCGGGGCGGGGCGAGGGGCGGGGCGGGGCGAGGC
GGAGAGGTGCGGCGGCAGCCAATCAGAGCGGCGCGCTCCGAAAGTTTCCTTTTATGGCGAGGCGGCGGCGGCGGCGGCCCT
ATAAAAAGCGAAGCGCGCGGCGGGCGGGAGTCGCTGCGTTGCCTTCGCCCCGTGCCCCGCTCCGCGCCGCCTCGCGCCGCC
CGCCCCGGCTCTGACTGACCGCGTTACTCCCACAGGTGAGCGGGCGGGACGGCCCTTCTCCTCCGGGCTGTAATTAGCGCTTG
GTTTAATGACGGCTCGTTTCTTTTCTGTGGCTGCGTGAAAGCCTTAAAGGGCTCCGGGAGGGCCCTTTGTGCGGGGGGGAGCG
GCTCGGGGGGTGCGTGCGTGTGTGTGTGCGTGGGGAGCGCCGCGTGCGGCCCGCGCTGCCCGGCGGCTGTGAGCGCTGCG
GGCGCGGCGCGGGGCTTTGTGCGCTCCGCGTGTGCGCGAGGGGAGCGCGGCCGGGGGCGGTGCCCCGCGGTGCGGGGGG
GCTGCGAGGGGAACAAAGGCTGCGTGCGGGGTGTGTGCGTGGGGGGGTGAGCAGGGGGTGTGGGCGCGGCGGTCGGGCTG
TAACCCCCCCCTGCACCCCCCTCCCCGAGTTGCTGAGCACGGCCCGGCTTCGGGTGCGGGGCTCCGTACGGGGCGTGGCGC
GGGGCTCGCCGTGCCGGGCGGGGGGTGGCGGCAGGTGGGGGTGCCGGGCGGGGCGGGGCCGCCTCGGGCCGGGGAGGG
CTCGGGGGAGGGGCGCGGCGGCCCCCGGAGCGCCGGCGGCTGTCGAGGCGCGGCGAGCCGCAGCCATTGCCTTTTATGGTA
ATCGTGCGAGAGGGCGCAGGGACTTCCTTTGTCCCAAATCTGTGCGGAGCCGAAATCTGGGAGGCGCCGCCGCACCCCCTCTA
GCGGGCGCGGGGCGAAGCGGTGCGGCGCCGGCAGGAAGGAAATGGGCGGGGAGGGCCTTCGTGCGTCGCCGCGCCGCCGT
CCCCTTCTCCCTCTCCAGCCTCGGGGCTGTCCGCGGGGGGACGGCTGCCTTCGGGGGGGACGGGGCAGGGCGGGGTTCGGC
TTCTGGCGTGTGACCGGCGGCTCTAGAGCCTCTGCTAACCATGTTCATGCCTTCTTCTTTTTCCTACAGCTCCTGGGCAACGTGC
TGGTTATTGTGCTGTCTCATCATTTTGGCAAAGAATTCGCCACCATGGTGAGCAAGGGCGAGGAGGATAACATGGCCATCATCA
AGGAGTTCATGCGCTTCAAGGTGCACATGGAGGGCTCCGTGAACGGCCACGAGTTCGAGATCGAGGGCGAGGGCGAGGGCC
GCCCCTACGAGGGCACCCAGACCGCCAAGCTGAAGGTGACCAAGGGTGGCCCCCTGCCCTTCGCCTGGGACATCCTGTCCC
CTCAGTTCATGTACGGCTCCAAGGCCTACGTGAAGCACCCCGCCGACATCCCCGACTACTTGAAGCTGTCCTTCCCCGAGGG
CTTCAAGTGGGAGCGCGTGATGAACTTCGAGGACGGCGGCGTGGTGACCGTGACCCAGGACTCCTCCCTGCAGGACGGCGA
GTTCATCTACAAGGTGAAGCTGCGCGGCACCAACTTCCCCTCCGACGGCCCCGTAATGCAGAAGAAGACCATGGGCTGGGA
GGCCTCCTCCGAGCGGATGTACCCCGAGGACGGCGCCCTGAAGGGCGAGATCAAGCAGAGGCTGAAGCTGAAGGACGGCG
GCCACTACGACGCTGAGGTCAAGACCACCTACAAGGCCAAGAAGCCCGTGCAGCTGCCCGGCGCCTACAACGTCAACATCA
AGTTGGACATCACCTCCCACAACGAGGACTACACCATCGTGGAACAGTACGAACGCGCCGAGGGCCGCCACTCCACCGGCG
GCATGGACGAGCTGTACGCGTCTGGAGGCGCCACCAATTTCAGCCTGCTGAAACAGGCTGGCGACGTGGAAGAGAACCCTG
GACCTGGACAAAAGTCGGTGAGCAAGGGCGAGGAGCTGTTCACCGGGGTGGTGCCCATCCTGGTCGAGCTGGACGGCGACG
TAAACGGCCACAAGTTCAGCGTGTCCGGCGAGGGCGAGGGCGATGCCACCTACGGCAAGCTGACCCTGAAGTTCATCTGCA
CCACCGGCAAGCTGCCCGTGCCCTGGCCCACCCTCGTGACCACCCTGACCTACGGCGTGCAGTGCTTCAGCCGCTACCCCGA
CCACATGAAGCAGCACGACTTCTTCAAGTCCGCCATGCCCGAAGGCTACGTCCAGGAGCGCACCATCTTCTTCAAGGACGAC
GGCAACTACAAGACCCGCGCCGAGGTGAAGTTCGAGGGCGACACCCTGGTGAACCGCATCGAGCTGAAGGGCATCGACTTC
AAGGAGGACGGCAACATCCTGGGGCACAAGCTGGAGTACAACTACAACAGCCACAGGAGTCTATATCATGGCCGACAAGCA
GAAGAACGGCATCAAGGTGAACTTCAAGATCCGCCACAACATCGAGGACGGCAGCGTGCAGCTCGCCGACCACTACCAGCA
GAACACCCCCATCGGCGACGGCCCCGTGCTGCTGCCCGACAACCACTACCTGAGCACCCAGTCCGCCCTGAGCAAAGACCC
CAACGAGAAGCGCGATCACATGGTCCTGCTGGAGTTCGTGACCGCCGCCGGGATCACTCTCGGCATGGACGAGCTGTACAA
GTAAGAATTCACTCCTCAGGTGCAGGCTGCCTATCAGAAGGTGGTGGCTGGTGTGGCCAATGCCCTGGCTCACAAATACCACTG
AGATCTTTTTCCCTCTGCCAAAAATTATGGGGACATCATGAAGCCCCTTGAGCATCTGACTTCTGGCTAATAAAGGAAATTTATTT
TCATTGCAATAGTGTGTTGGAATTTTTTGTGTCTCTCACTCGGAAGGACATATGGGAGGGGTCGACAATCAACCTCTGGATTACA
AAATTTGTGAAAGATTGACTGGTATTCTTAACTATGTTGCTCCTTTTACGCTATGTGGATACGCTGCTTTAATGCCTTTGTATCATG
CGTTAACTAAACTTGTTTATTGCAGCTTATAATGGTTACAAATAAAGCAATAGCATCACAAATTTCACAAATAAAGCATTTTTTTCAC
TGCATTCTAGTTGTGGTTTGTCCAAACTCATCAATGTATCTTATCATGTCTGGAATTGACTCAAATGATGTCAATTAGTCTATCAGA
AGCTATCTGGTCTCCCTTCCGGGGGACAAGACATCCCTGTTTAATATTTAAACAGCAGTGTTCCCAAACTGGGTTCTTATATCCCT
TGCTCTGGTCAACCAGGTTGCAGGGTTTCCTGTCCTCACAGGAACGAAGTCCCTAAAGAAACAGTGGCAGCCAGGTTTAGCCCC
GGAATTGACTGGATTCCTTTTTTAGGGCCCATTGGTATGGCTTTTTCCCCGTATCCCCCCAGGTGTCTGCAGGCTCAAAGAGCAG
CGAGAAGCGTTCAGAGGAAAGCGATCCCGTGCCACCTTCCCCGTGCCCGGGCTGTCCCCGCACGCTGCCGGCTCGGGGATGC
GGGGGGAGCGCCGGACCGGAGCGGAGCCCCGGGCGGCTCGCTGCTGCCCCCTAGCGGGGGAGGGACGTAATTACATCCCTG
GGGGCTTTGGGGGGGGGCTGTCCCTGATATCTATAACAAGAAAATATATATATAATAAGTTATCACGTAAGTAGAACATGAAATAA
CAATATAATTATCGTATGAGTTAAATCTTAAAAGTCACGTAAAAGATAATCATGCGTCATTTTGACTCACGCGGTCGTTATAGTTCA
AAATCAGTGACACTTACCGCATTGACAAGCACGCCTCACGGGAGCTCCAAGCGGCGACTGAGATGTCCTAAATGCACAGCGACG
GATTCGCGCTATTTAGAAAGAGAGAGCAATATTTCAAGAATGCATGCGTCAATTTTACGCAGACTATCTTTCTAGGGTTAATCTAG
CTGCATCAGGATCATATCGTCGGGTCTTTTTCCGGCTCAGTCATCGCCCAAGCTGGCGCTATCTGGGCATCGGGGAGGAAGAA

GCCCGTGCCTTTTCCCGCGAGGTTGAAGCGGCATGGAAAGAGTTTGCCGAGGATGACTGCTGCTGCATTGACGTTGAGCGAAA
ACGCACGTTTACCATGATGATTCGGGAAGGTGTGGCCATGCACGCCTTTAACGGTGAACTGTTCGTTCAGGCCACCTGGGATAC
CAGTTCGTCGCGGCTTTTCCGGACACAGTTCCGGATGGTCAGCCCGAAGCGCATCAGCAACCCGAACAATACCGGCGACAGCC
GGAACTGCCGTGCCGGTGTGCAGATTAATGACAGCGGTGCGGCGCTGGGATATTACGTCAGCGAGGACGGGTATCCTGGCTG
GATGCCGCAGAAATGGACATGGATACCCCGTGAGTTACCCGGCGGGCGCGCTTGGCGTAATCATGGTCATAGCTGTTTCCTGT
GTGAAATTGTTATCCGCTCACAATTCCACACAACATACGAGCCGGAAGCATAAAGTGTAAAGCCTGGGGTGCCTAATGAGTGAG
CTAACTCACATTAATTGCGTTGCGCTCACTGCCCGCTTTCCAGTCGGGAAACCTGTCGTGCCAGCTGCATTAATGAATCGGCCAA
CGCGCGGGGAGAGGCGGTTTGCGTATTGGGCGCTCTTCCGCTTCCTCGCTCACTGACTCGCTGCGCTCGGTCGTTCGGCTGCG
GCGAGCGGTATCAGCTCACTCAAAGGCGGTAATACGGTTATCCACAGAATCAGGGGATAACGCAGGAAAGAACATGTGAGCAAA
AGGCCAGCAAAAGGCCAGGAACCGTAAAAAGGCCGCGTTGCTGGCGTTTTTCCATAGGCTCCGCCCCCCTGACGAGCATCACA
AAAATCGACGCTCAAGTCAGAGGTGGCGAAACCCGACAGGACTATAAAGATACCAGGCGTTTCCCCCTGGAAGCTCCCTCGTGC
GCTCTCCTGTTCCGACCCTGCCGCTTACCGGATACCTGTCCGCCTTTCTCCCTTCGGGAAGCGTGGCGCTTTCTCATAGCTCAC
GCTGTAGGTATCTCAGTTCGGTGTAGGTCGTTCGCTCCAAGCTGGGCTGTGTGCACGAACCCCCCGTTCAGCCCGACCGCTGC
GCCTTATCCGGTAACTATCGTCTTGAGTCCAACCCGGTAAGACACGACTTATCGCCACTGGCAGCAGCCACTGGTAACAGGATT
AGCAGAGCGAGGTATGTAGGCGGTGCTACAGAGTTCTTGAAGTGGTGGCCTAACTACGGCTACACTAGAAGGACAGTATTTGGT
ATCTGCGCTCTGCTGAAGCCAGTTACCTTCGGAAAAAGAGTTGGTAGCTCTTGATCCGGCAAACAAACCACCGCTGGTAGCGGT
GGTTTTTTTGTTTGCAAGCAGCAGATTACGCGCAGAAAAAAAGGATCTCAAGAAGATCCTTTGATCTTTTCTACGGGGTCTGACG
CTCAGTGGAACGAAAACTCACGTTAAGGGATTTTGGTCATGAGATTATCAAAAAGGATCTTCACCTAGATCCTTTTAAATTAAAAAT
GAAGTTTTAAATCAATCTAAAGTATATATGAGTAAACTTGGTCTGACAGTTACCAATGCTTAATCAGTGAGGCACCTATCTCAGCG
ATCTGTCTATTTCGTTCATCCATAGTTGCCTGACTCCCCGTCGTGTAGATAACTACGATACGGGAGGGCTTACCATCTGGCCCCA
GTGCTGCAATGATACCGCGAGACCCACGCTCACCGGCTCCAGATTTATCAGCAATAAACCAGCCAGCCGGAAGGGCCGAGCGC
AGAAGTGGTCCTGCAACTTTATCCGCCTCCATCCAGTCTATTAATTGTTGCCGGGAAGCTAGAGTAAGTAGTTCGCCAGTTAATA
GTTTGCGCAACGTTGTTGCCATTGCTACAGGCATCGTGGTGTCACGCTCGTCGTTTGGTATGGCTTCATTCAGCTCCGGTTCCCA
ACGATCAAGGCGAGTTACATGATCCCCCATGTTGTGCAAAAAAGCGGTTAGCTCCTTCGGTCCTCCGATCGTTGTCAGAAGTAAG
TTGGCCGCAGTGTTATCACTCATGGTTATGGCAGCACTGCATAATTCTCTTACTGTCATGCCATCCGTAAGATGCTTTTCTGTGAC
TGGTGAGTACTCAACCAAGTCATTCTGAGAATAGTGTATGCGGCGACCGAGTTGCTCTTGCCCGGCGTCAATACGGGATAATAC
CGCGCCACATAGCAGAACTTTAAAAGTGCTCATCATTGGAAAACGTTCTTCGGGGCGAAAACTCTCAAGGATCTTACCGCTGTTG
AGATCCAGTTCGATGTAACCCACTCGTGCACCCAACTGATCTTCAGCATCTTTTACTTTCACCAGCGTTTCTGGGTGAGCAAAAA
CAGGAAGGCAAAATGCCGCAAAAAAGGGAATAAGGGCGACACGGAAATGTTGAATACTCAT

2.2.12 Vector 65: Pyl tRNA$_{UCCU(Ev1)}$ PylRS mCherry-P2A-eGFP(150AGGA)

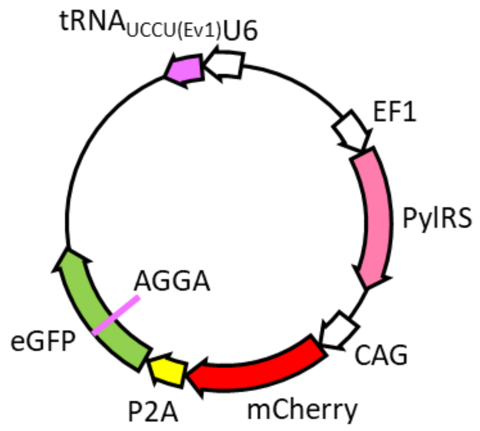

ACTCTTCCTTTTTCAATATTATTGAAGCATTTATCAGGGTTATTGTCTCATGAGCGGATACATATTTGAATGTATTTAGAAAAATAAA
CAAATAGGGGTTCCGCGCACATTTCCCCGAAAAGTGCCACCTAAATTGTAAGCGTTAATATTTTGTTAAAATTCGCGTTAAATTTTT
GTTAAATCAGCTCATTTTTTAACCAATAGGCCGAAATCGGCAAAATCCCTTATAAATCAAAAGAATAGACCGAGATAGGGTTGAGT
GTTGTTCCAGTTTGGAACAAGAGTCCACTATTAAAGAACGTGGACTCCAACGTCAAAGGGCGAAAAACCGTCTATCAGGGCGAT
GGCCCACTACGTGAACCATCACCCTAATCAAGTTTTTTGGGGTCGAGGTGCCGTAAAGCACTAAATCGGAACCCTAAAGGGAGC
CCCCGATTTAGAGCTTGACGGGGAAAGCCGGCGAACGTGGCGAGAAAGGAAGGGAAGAAAGCGAAAGGAGCGGGCGCTAGGG
CGCTGGCAAGTGTAGCGGTCACGCTGCGCGTAACCACCACACCCGCCGCGCTTAATGCGCCGCTACAGGGCGCGTCCCATTC
GCCATTCAGGCTGCGCAACTGTTGGGAAGGGCGATCGGTGCGGGCCTCTTCGCTATTACGCCAGCTGGCGAAAGGGGGATGT
GCTGCAAGGCGATTAAGTTGGGTAACGCCAGGGTTTTCCCAGTCACGACGTTGTAAAACGACGGCCAGTGAGCGCGCCTCGTT
CATTCACGTTTTTGAACCCGTGGAGGACGGGCAGACTCGCGGTGCAAATGTGTTTTACAGCGTGATGGAGCAGATGAAGATGCT
CGACACGCTGCAGAACACGCAGCTAGATTAACCCTAGAAAGATAATCATATTGTGACGTACGTTAAAGATAATCATGCGTAAAATT
GACGCATGTGTTTTATCGGTCTGTATATCGAGGTTTATTTATTAATTTGAATAGATATTAAGTTTTATTATATTTACACTTACATACTA
ATAATAAATTCAACAAACAATTTATTTATGTTTATTTATTTATTAAAAAAAAACAAAAACTCAAAATTTCTTCTATAAAGTAACAAAACTT
TTATGAGGGACAGCCCCCCCCCAAAGCCCCCAGGGATGTAATTACGTCCCTCCCCCGCTAGGGGGCAGCAGCGAGCCGCCCG
GGGCTCCGCTCCGGTCCGGCGCTCCCCCCGCATCCCCGAGCCGGCAGCGTGCGGGGACAGCCCGGGCACGGGGAAGGTGG
CACGGGATCGCTTTCCTCTGAACGCTTCTCGCTGCTCTTTGAGCCTGCAGACACCTGGGGGGATACGGGGAAAAGGCCTAAAAA
CCGCACTTGTC**CGGAAACCCCGGGAATCTAACCCGGCTGAACCTATTAGGAAGAGGATTCGATCTACATGATCAGGTTTCC**G
GTGTTTCGTCCTTTCCACAAGATATATAAAGCCAAGAAATCGAAATACTTTCAAGTTACGGTAAGCATATGATAGTCCATTTTAAAA
CATAATTTTAAAACTGCAAACTACCCAAGAAATTATTACTTTCTACGTCACGTATTTTGTACTAATATCTTTGTGTTTACAGTCAAAT
TAATTCTAATTATCTCTCTAACAGCCTTGTATCGTATATGCAAATATGAAGGAATCATGGGAAATAGGCCCTCTTCCTGCCCAACC
GGTTGCGATCGCTCCGGTGCCCGTCAGTGGGCAGAGCGCACATCGCCCACAGTCCCCGAGAAGTTGGGGGGAGGGGTCGGCA
ATTGAACGGGTGCCTAGAGAAGGTGGCGCGGGGTAAACTGGGAAAGTGATGTCGTGTACTGGCTCCGCCTTTTTCCCGAGGGT
GGGGGAGAACCGTATATAAGTGCAGTAGTCGCCGTGAACGTTCTTTTTCGCAACGGGTTTGCCGCCAGAACACAGCTGAAGCTT
CGAGGGGCTCGCATCTCTCCTTCACGCGCCCGCCGCCCTACCTGAGGCCGCCATCCACGCCGGTTGAGTCGCGTTCTGCCGC
CTCCCGCCTGTGGTGCCTCCTGAACTGCGTCCGCCGTCTAGGTAAGTTTAAAGCTCAGGTCGAGACCGGGCCTTTGTCCGGCG

CTCCCTTGGAGCCTACCTAGACTCAGCCGGCTCTCCACGCTTTGCCTGACCCTGCTTGCTCAACTCTACGTCTTTGTTTCGTTTT
CTGTTCTGCGCCGTTACAGATCCAAGCTGTGACCGGCGCCTACTCTACAGATAGCGTTTAAACTTACGCTTGCCACCATGGCTA
GCGACTACAAGGACGACGACGACAAGGACAAGAAGCCCCTGAACACCCTGATCAGCGCCACAGGACTGTGGATGTCCAGAA
CCGGCACCATCCACAAGATCAAGCACCACGAGGTGTCCCGGTCCAAAATCTACATCGAGATGGCCTGCGGCGATCACCTGGT
CGTCAACAACAGCAGAAGCAGCCGGACAGCCAGAGCCCTGCGGCACCACAAGTACAGAAAGACCTGCAAGCGGTGCAGAG
TGTCCGACGAGGACCTGAACAAGTTCCTGACCAAGGCCAACGAGGACCAGACCAGCGTGAAAGTGAAGGTGGTGTCCGCCC
CCACCCGGACCAAGAAAGCCATGCCCAAGAGCGTGGCCAGAGCCCCCAAGCCCCTGGAAAACACCGAAGCCGCTCAGGCC
CAGCCCAGCGGCAGCAAGTTCAGCCCCGCCATCCCCGTGTCTACCCAGGAAAGCGTCAGCGTCCCCGCCAGCGTGTCCACC
AGCATCTCTAGCATCTCAACCGGCGCCACAGCTTCTGCCCTGGTCAAGGGCAACACCAACCCCATCACCAGCATGTCTGCCC
CTGTGCAAGCCTCTGCCCCAGCCCTGACCAAGTCCCAGACCGACCGGCTGGAAGTGCTCCTGAACCCCAAGGACGAGATCA
GCCTGAACAGCGGCAAGCCCTTCCGGGAGCTGGAAAGCGAGCTGCTGAGCCGGCGGAAGAAGGACCTCCAGCAAATCTACG
CCGAGGAACGGGAGAACTACCTGGGCAAGCTGGAAAGAGAGATCACCCGGTTCTTCGTGGACCGGGGCTTCCTGGAAATCA
AGAGCCCCATCCTGATCCCCCTGGAGTACATCGAGCGGATGGGCATCGACAACGACACCGAGCTGAGCAAGCAGATTTTCCG
GGTGGACAAGAACTTCTGCCTGCGGCCCATGCTGGCCCCCAACCTGTACAACTACCTGCGGAAACTGGATCGCGCTCTGCCC
GACCCCATCAAGATTTTCGAGATCGGCCCCTGCTACCGGAAAGAGAGCGACGGCAAAGAGCACCTGGAAGAGTTTACAATGC
TGAACTTTTGCCAGATGGGCAGCGGCTGCACCAGAGAGAACCTGGAATCCATCATCACCGACTTTCTGAACCACCTGGGGAT
CGACTTCAAGATCGTGGGCGACAGCTGCATGGTGTACGGCGACACCCTGGACGTGATGCACGGCGACCTGGAACTGTCTAG
CGCCGTCGTGGGACCCATCCCTCTGGACCGGGAGTGGGGCATCGATAAGCCCTGGATCGGAGCCGGCTTCGGCCTGGAACG
GCTGCTGAAAGTCAAGCACGACTTTAAGAACATCAAGCGGGCTGCCAGAAGCGAGAGCTACTACAACGGCATCAGCACCAA
CCTGTGATGATAAGGATCCACTAGTCCAGTGTGGTGGAATTGACATTGATTATTGACTAGTTATTAATAGTAATCAATTACGGGGT
CATTAGTTCATAGCCCATATATGGAGTTCCGCGTTACATAACTTACGGTAAATGGCCCGCCTGGCTGACCGCCCAACGACCCCC
GCCCATTGACGTCAATAATGACGTATGTTCCCATAGTAACGCCAATAGGGACTTTCCATTGACGTCAATGGGTGGAGTATTTACG
GTAAACTGCCCACTTGGCAGTACATCAAGTGTATCATATGCCAAGTACGCCCCCTATTGACGTCAATGACGGTAAATGGCCCGCC
TGGCATTATGCCCAGTACATGACCTTATGGGACTTTCCTACTTGGCAGTACATCTACGTATTAGTCATCGCTATTACCATGGTCGA
GGTGAGCCCCACGTTCTGCTTCACTCTCCCCATCTCCCCCCCCTCCCCACCCCCAATTTTGTATTTATTTATTTTTTAATTATTTTG
TGCAGCGATGGGGGCGGGGGGGGGGGGGGGCGCGCGCCAGGCGGGGCGGGGCGGGGCGAGGGGCGGGGCGGGGCGAGGC
GGAGAGGTGCGGCGGCAGCCAATCAGAGCGGCGCGCTCCGAAAGTTTCCTTTTATGGCGAGGCGGCGGCGGCGGCGGCCCT
ATAAAAAGCGAAGCGCGCGGCGGGCGGGAGTCGCTGCGTTGCCTTCGCCCCGTGCCCCGCTCCGCGCCGCCTCGCGCCGCC
CGCCCCGGCTCTGACTGACCGCGTTACTCCCACAGGTGAGCGGGCGGGACGGCCCTTCTCCTCCGGGCTGTAATTAGCGCTTG
GTTTAATGACGGCTCGTTTCTTTTCTGTGGCTGCGTGAAAGCCTTAAAGGGCTCCGGGAGGGCCCTTTGTGCGGGGGGGAGCG
GCTCGGGGGGTGCGTGCGTGTGTGTGTGCGTGGGGAGCGCCGCGTGCGGCCCGCGCTGCCCGGCGGCTGTGAGCGCTGCG
GGCGCGGCGCGGGGCTTTGTGCGCTCCGCGTGTGCGCGAGGGGAGCGCGGCCGGGGGCGGTGCCCCGCGGTGCGGGGGG
GCTGCGAGGGGAACAAAGGCTGCGTGCGGGGTGTGTGCGTGGGGGGGTGAGCAGGGGGTGTGGGCGCGGCGGTCGGGCTG
TAACCCCCCCCTGCACCCCCCTCCCCGAGTTGCTGAGCACGGCCCGGCTTCGGGTGCGGGGCTCCGTACGGGGCGTGGCGC
GGGGCTCGCCGTGCCGGGCGGGGGGTGGCGGCAGGTGGGGGTGCCGGGCGGGGCGGGGCCGCCTCGGGCCGGGGAGGG
CTCGGGGGAGGGGCGCGGCGGCCCCCGGAGCGCCGGCGGCTGTCGAGGCGCGGCGAGCCGCAGCCATTGCCTTTTATGGTA
ATCGTGCGAGAGGGCGCAGGGACTTCCTTTGTCCCAAATCTGTGCGGAGCCGAAATCTGGGAGGCGCCGCCGCACCCCCTCTA
GCGGGCGCGGGGCGAAGCGGTGCGGCGCCGGCAGGAAGGAAATGGGCGGGGAGGGCCTTCGTGCGTCGCCGCGCCGCCGT
CCCCTTCTCCCTCTCCAGCCTCGGGGCTGTCCGCGGGGGGACGGCTGCCTTCGGGGGGGACGGGGCAGGGCGGGGTTCGGC
TTCTGGCGTGTGACCGGCGGCTCTAGAGCCTCTGCTAACCATGTTCATGCCTTCTTCTTTTTCCTACAGCTCCTGGGCAACGTGC
TGGTTATTGTGCTGTCTCATCATTTTGGCAAAGAATTCGCCACCATGGTGAGCAAGGGCGAGGAGGATAACATGGCCATCATCA
AGGAGTTCATGCGCTTCAAGGTGCACATGGAGGGCTCCGTGAACGGCCACGAGTTCGAGATCGAGGGCGAGGGCGAGGGCC
GCCCCTACGAGGGCACCCAGACCGCCAAGCTGAAGGTGACCAAGGGTGGCCCCCTGCCCTTCGCCTGGGACATCCTGTCCC
CTCAGTTCATGTACGGCTCCAAGGCCTACGTGAAGCACCCCGCCGACATCCCCGACTACTTGAAGCTGTCCTTCCCCGAGGG
CTTCAAGTGGGAGCGCGTGATGAACTTCGAGGACGGCGGCGTGGTGACCGTGACCCAGGACTCCTCCCTGCAGGACGGCGA
GTTCATCTACAAGGTGAAGCTGCGCGGCACCAACTTCCCCTCCGACGGCCCCGTAATGCAGAAGAAGACCATGGGCTGGGA
GGCCTCCTCCGAGCGGATGTACCCCGAGGACGGCGCCCTGAAGGGCGAGATCAAGCAGAGGCTGAAGCTGAAGGACGGCG
GCCACTACGACGCTGAGGTCAAGACCACCTACAAGGCCAAGAAGCCCGTGCAGCTGCCCGGCGCCTACAACGTCAACATCA
AGTTGGACATCACCTCCCACAACGAGGACTACACCATCGTGGAACAGTACGAACGCGCCGAGGGCCGCCACTCCACCGGCG
GCATGGACGAGCTGTACGCGTCTGGAGGCGCCACCAATTTCAGCCTGCTGAAACAGGCTGGCGACGTGGAAGAGAACCCTG
GACCTGGACAAAAGTCGGTGAGCAAGGGCGAGGAGCTGTTCACCGGGGTGGTGCCCATCCTGGTCGAGCTGGACGGCGACG
TAAACGGCCACAAGTTCAGCGTGTCCGGCGAGGGCGAGGGCGATGCCACCTACGGCAAGCTGACCCTGAAGTTCATCTGCA
CCACCGGCAAGCTGCCCGTGCCCTGGCCCACCCTCGTGACCACCCTGACCTACGGCGTGCAGTGCTTCAGCCGCTACCCCGA
CCACATGAAGCAGCACGACTTCTTCAAGTCCGCCATGCCCGAAGGCTACGTCCAGGAGCGCACCATCTTCTTCAAGGACGAC
GGCAACTACAAGACCCGCGCCGAGGTGAAGTTCGAGGGCGACACCCTGGTGAACCGCATCGAGCTGAAGGGCATCGACTTC
AAGGAGGACGGCAACATCCTGGGGCACAAGCTGGAGTACAACTACAACAGCCACAGGAGTCTATATCATGGCCGACAAGCA
GAAGAACGGCATCAAGGTGAACTTCAAGATCCGCCACAACATCGAGGACGGCAGCGTGCAGCTCGCCGACCACTACCAGCA
GAACACCCCCATCGGCGACGGCCCCGTGCTGCTGCCCGACAACCACTACCTGAGCACCCAGTCCGCCCTGAGCAAAGACCC
CAACGAGAAGCGCGATCACATGGTCCTGCTGGAGTTCGTGACCGCCGCCGGGATCACTCTCGGCATGGACGAGCTGTACAA
GTAAGAATTCACTCCTCAGGTGCAGGCTGCCTATCAGAAGGTGGTGGCTGGTGTGGCCAATGCCCTGGCTCACAAATACCACTG
AGATCTTTTTCCCTCTGCCAAAAATTATGGGGACATCATGAAGCCCCTTGAGCATCTGACTTCTGGCTAATAAAGGAAATTTATTT
TCATTGCAATAGTGTGTTGGAATTTTTTGTGTCTCTCACTCGGAAGGACATATGGGAGGGGTCGACAATCAACCTCTGGATTACA
AAATTTGTGAAAGATTGACTGGTATTCTTAACTATGTTGCTCCTTTTACGCTATGTGGATACGCTGCTTTAATGCCTTTGTATCATG
CGTTAACTAAACTTGTTTATTGCAGCTTATAATGGTTACAAATAAAGCAATAGCATCACAAATTTCACAAATAAAGCATTTTTTTCAC
TGCATTCTAGTTGTGGTTTGTCCAAACTCATCAATGTATCTTATCATGTCTGGAATTGACTCAAATGATGTCAATTAGTCTATCAGA
AGCTATCTGGTCTCCCTTCCGGGGGACAAGACATCCCTGTTTAATATTTAAACAGCAGTGTTCCCAAACTGGGTTCTTATATCCCT
TGCTCTGGTCAACCAGGTTGCAGGGTTTCCTGTCCTCACAGGAACGAAGTCCCTAAAGAAACAGTGGCAGCCAGGTTTAGCCCC
GGAATTGACTGGATTCCTTTTTTAGGGCCCATTGGTATGGCTTTTTCCCCGTATCCCCCCAGGTGTCTGCAGGCTCAAAGAGCAG
CGAGAAGCGTTCAGAGGAAAGCGATCCCGTGCCACCTTCCCCGTGCCCGGGCTGTCCCCGCACGCTGCCGGCTCGGGGATGC
GGGGGGAGCGCCGGACCGGAGCGGAGCCCCGGGCGGCTCGCTGCTGCCCCCTAGCGGGGGAGGGACGTAATTACATCCCTG
GGGGCTTTGGGGGGGGGCTGTCCCTGATATCTATAACAAGAAAATATATATATATAATAAGTTATCACGTAAGTAGAACATGAAATAA
CAATATAATTATCGTATGAGTTAAATCTTAAAAGTCACGTAAAAGATAATCATGCGTCATTTTGACTCACGCGGTCGTTATAGTTCA
AAATCAGTGACACTTACCGCATTGACAAGCACGCCTCACGGGAGCTCCAAGCGGCGACTGAGATGTCCTAAATGCACAGCGACG
GATTCGCGCTATTTAGAAAGAGAGAGCAATATTTCAAGAATGCATGCGTCAATTTTACGCAGACTATCTTTCTAGGGTTAATCTAG

CTGCATCAGGATCATATCGTCGGGTCTTTTTTCCGGCTCAGTCATCGCCCAAGCTGGCGCTATCTGGGCATCGGGGAGGAAGAA
GCCCGTGCCTTTTCCCGCGAGGTTGAAGCGGCATGGAAAGAGTTTGCCGAGGATGACTGCTGCTGCATTGACGTTGAGCGAAA
ACGCACGTTTACCATGATGATTCGGGAAGGTGTGGCCATGCACGCCTTTAACGGTGAACTGTTCGTTCAGGCCACCTGGGATAC
CAGTTCGTCGCGGCTTTTCCGGACACAGTTCCGGATGGTCAGCCCGAAGCGCATCAGCAACCCGAACAATACCGGCGACAGCC
GGAACTGCCGTGCCGGTGTGCAGATTAATGACAGCGGTGCGGCGCTGGGATATTACGTCAGCGAGGACGGGTATCCTGGCTG
GATGCCGCAGAAATGGACATGGATACCCCGTGAGTTACCCGGCGGGCGCGCTTGGCGTAATCATGGTCATAGCTGTTTCCTGT
GTGAAATTGTTATCCGCTCACAATTCCACACAACATACGAGCCGGAAGCATAAAGTGTAAAGCCTGGGGTGCCTAATGAGTGAG
CTAACTCACATTAATTGCGTTGCGCTCACTGCCCGCTTTCCAGTCGGGAAACCTGTCGTGCCAGCTGCATTAATGAATCGGCCAA
CGCGCGGGGAGAGGCGGTTTGCGTATTGGGCGCTCTTCCGCTTCCTCGCTCACTGACTCGCTGCGCTCGGTCGTTCGGCTGCG
GCGAGCGGTATCAGCTCACTCAAAGGCGGTAATACGGTTATCCACAGAATCAGGGGATAACGCAGGAAAGAACATGTGAGCAAA
AGGCCAGCAAAAGGCCAGGAACCGTAAAAAGGCCGCGTTGCTGGCGTTTTTCCATAGGCTCCGCCCCCCTGACGAGCATCACA
AAAATCGACGCTCAAGTCAGAGGTGGCGAAACCCGACAGGACTATAAAGATACCAGGCGTTTCCCCCTGGAAGCTCCCTCGTGC
GCTCTCCTGTTCCGACCCTGCCGCTTACCGGATACCTGTCCGCCTTTCTCCCTTCGGGAAGCGTGGCGCTTTCTCATAGCTCAC
GCTGTAGGTATCTCAGTTCGGTGTAGGTCGTTCGCTCCAAGCTGGGCTGTGTGCACGAACCCCCCGTTCAGCCCGACCGCTGC
GCCTTATCCGGTAACTATCGTCTTGAGTCCAACCCGGTAAGACACGACTTATCGCCACTGGCAGCAGCCACTGGTAACAGGATT
AGCAGAGCGAGGTATGTAGGCGGTGCTACAGAGTTCTTGAAGTGGTGGCCTAACTACGGCTACACTAGAAGGACAGTATTTGGT
ATCTGCGCTCTGCTGAAGCCAGTTACCTTCGGAAAAAGAGTTGGTAGCTCTTGATCCGGCAAACAAACCACCGCTGGTAGCGGT
GGTTTTTTTGTTTGCAAGCAGCAGATTACGCGCAGAAAAAAAGGATCTCAAGAAGATCCTTTGATCTTTTCTACGGGGTCTGACG
CTCAGTGGAACGAAAACTCACGTTAAGGGATTTTGGTCATGAGATTATCAAAAAGGATCTTCACCTAGATCCTTTTAAATTAAAAAT
GAAGTTTTAAATCAATCTAAAGTATATATGAGTAAACTTGGTCTGACAGTTACCAATGCTTAATCAGTGAGGCACCTATCTCAGCG
ATCTGTCTATTTCGTTCATCCATAGTTGCCTGACTCCCCGTCGTGTAGATAACTACGATACGGGAGGGCTTACCATCTGGCCCCA
GTGCTGCAATGATACCGCGAGACCCACGCTCACCGGCTCCAGATTTATCAGCAATAAACCAGCCAGCCGGAAGGGCCGAGCGC
AGAAGTGGTCCTGCAACTTTATCCGCCTCCATCCAGTCTATTAATTGTTGCCGGGAAGCTAGAGTAAGTAGTTCGCCAGTTAATA
GTTTGCGCAACGTTGTTGCCATTGCTACAGGCATCGTGGTGTCACGCTCGTCGTTTGGTATGGCTTCATTCAGCTCCGGTTCCCA
ACGATCAAGGCGAGTTACATGATCCCCCATGTTGTGCAAAAAAGCGGTTAGCTCCTTCGGTCCTCCGATCGTTGTCAGAAGTAAG
TTGGCCGCAGTGTTATCACTCATGGTTATGGCAGCACTGCATAATTCTCTTACTGTCATGCCATCCGTAAGATGCTTTTCTGTGAC
TGGTGAGTACTCAACCAAGTCATTCTGAGAATAGTGTATGCGGCGACCGAGTTGCTCTTGCCCGGCGTCAATACGGGATAATAC
CGCGCCACATAGCAGAACTTTAAAAGTGCTCATCATTGGAAAACGTTCTTCGGGGCGAAAACTCTCAAGGATCTTACCGCTGTTG
AGATCCAGTTCGATGTAACCCACTCGTGCACCCAACTGATCTTCAGCATCTTTTACTTTCACCAGCGTTTCTGGGTGAGCAAAAA
CAGGAAGGCAAAATGCCGCAAAAAAGGGAATAAGGGCGACACGGAAATGTTGAATACTCAT

2.2.13 Vector 66: Pyl $tRNA_{UCCU(Ev2)}$ PylRS mCherry-P2A-eGFP(150AGGA)

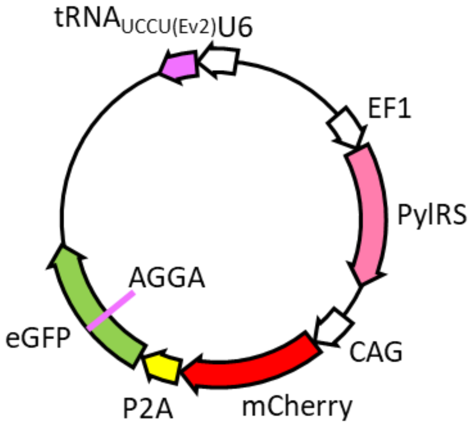

ACTCTTCCTTTTTCAATATTATTGAAGCATTTATCAGGGTTATTGTCTCATGAGCGGATACATATTTGAATGTATTTAGAAAAATAAA
CAAATAGGGGTTCCGCGCACATTTCCCCGAAAAGTGCCACCTAAATTGTAAGCGTTAATATTTTGTTAAAATTCGCGTTAAATTTTT
GTTAAATCAGCTCATTTTTTAACCAATAGGCCGAAATCGGCAAAATCCCTTATAAATCAAAAGAATAGACCGAGATAGGGTTGAGT
GTTGTTCCAGTTTGGAACAAGAGTCCACTATTAAAGAACGTGGACTCCAACGTCAAAGGGCGAAAAACCGTCTATCAGGGCGAT
GGCCCACTACGTGAACCATCACCCTAATCAAGTTTTTTGGGGTCGAGGTGCCGTAAAGCACTAAATCGGAACCCTAAAGGGAGC
CCCCGATTTAGAGCTTGACGGGGAAAGCCGGCGAACGTGGCGAGAAAGGAAGGGAAGAAAGCGAAAGGAGCGGGCGCTAGGG
CGCTGGCAAGTGTAGCGGTCACGCTGCGCGTAACCACCACACCCGCCGCGCTTAATGCGCCGCTACAGGGCGCGTCCCATTC
GCCATTCAGGCTGCGCAACTGTTGGGAAGGGCGATCGGTGCGGGCCTCTTCGCTATTACGCCAGCTGGCGAAAGGGGGATGT
GCTGCAAGGCGATTAAGTTGGGTAACGCCAGGGTTTTCCCAGTCACGACGTTGTAAAACGACGGCCAGTGAGCGCGCCTCGTT
CATTCACGTTTTTGAACCCGTGGAGGACGGGCAGACTCGCGGTGCAAATGTGTTTTACAGCGTGATGGAGCAGATGAAGATGCT
CGACACGCTGCAGAACACGCAGCTAGATTAACCCTAGAAAGATAATCATATTGTGACGTACGTTAAAGATAATCATGCGTAAAATT
GACGCATGTGTTTTATCGGTCTGTATATCGAGGTTTATTTATTAATTTGAATAGATATTAAGTTTTATTATATTTACACTTACATACTA
ATAATAAATTCAACAAACAATTTATTTATGTTTATTTATTTATTAAAAAAAAACAAAAACTCAAAATTTCTTCTATAAAGTAACAAAACTT
TTATGAGGGACAGCCCCCCCCCAAAGCCCCCAGGGATGTAATTACGTCCCTCCCCCGCTAGGGGGCAGCAGCGAGCCGCCCG
GGGCTCCGCTCCGGTCCGGCGCTCCCCCCGCATCCCCGAGCCGGCAGCGTGCGGGGACAGCCCGGGCACGGGGAAGGTGG
CACGGGATCGCTTTCCTCTGAACGCTTCTCGCTGCTCTTTGAGCCTGCAGACACCTGGGGGGATACGGGGAAAAGGCCTAAAAA
CCGCACTTGTCCGGAAACCCCGGGAATCTAACCCGGCTGAAGCGGATAGGAAGCCCCTTCGATCTACATGATCAGGTTTCCG
GTGTTTCGTCCTTTCCACAAGATATATAAAGCCAAGAAATCGAAATACTTTCAAGTTACGGTAAGCATATGATAGTCCATTTTAAAA
CATAATTTTAAAACTGCAAACTACCCAAGAAATTATTACTTTCTACGTCACGTATTTTGTACTAATATCTTTGTGTTTACAGTCAAAT
TAATTCTAATTATCTCTCTAACAGCCTTGTATCGTATATGCAAATATGAAGGAATCATGGGAAATAGGCCCTCTTCCTGCCCAACC
GGTTGCGATCGCTCCGGTGCCCGTCAGTGGGCAGAGCGCACATCGCCCACAGTCCCCGAGAAGTTGGGGGGAGGGGTCGGCA
ATTGAACGGGTGCCTAGAGAAGGTGGCGCGGGGTAAACTGGGAAAGTGATGTCGTGTACTGGCTCCGCCTTTTTCCCGAGGGT
GGGGGAGAACCGTATATAAGTGCAGTAGTCGCCGTGAACGTTCTTTTTCGCAACGGGTTTGCCGCCAGAACACAGCTGAAGCTT
CGAGGGGCTCGCATCTCTCCTTCACGCGCCCGCCGCCCTACCTGAGGCCGCCATCCACGCCGGTTGAGTCGCGTTCTGCCGC

CTCCCGCCTGTGGTGCCTCCTGAACTGCGTCCGCCGTCTAGGTAAGTTTAAAGCTCAGGTCGAGACCGGGCCTTTGTCCGGCG
CTCCCTTGGAGCCTACCTAGACTCAGCCGGCTCTCCACGCTTTGCCTGACCCTGCTTGCTCAACTCTACGTCTTTGTTTCGTTTT
CTGTTCTGCGCCGTTACAGATCCAAGCTGTGACCGGCGCCTACTCTACAGATAGCGTTTAAACTTACGCTTGCCACCATGGCTA
GCGACTACAAGGACGACGACGACAAGGACAAGAAGCCCCTGAACACCCTGATCAGCGCCACAGGACTGTGGATGTCCAGAA
CCGGCACCATCCACAAGATCAAGCACCACGAGGTGTCCCGGTCCAAAATCTACATCGAGATGGCCTGCGGCGATCACCTGGT
CGTCAACAACAGCAGAAGCAGCCGGACAGCCAGAGCCCTGCGGCACCACAAGTACAGAAAGACCTGCAAGCGGTGCAGAG
TGTCCGACGAGGACCTGAACAAGTTCCTGACCAAGGCCAACGAGGACCAGACCAGCGTGAAAGTGAAGGTGGTGTCCGCCC
CCACCCGGACCAAGAAAGCCATGCCCAAGAGCGTGGCCAGAGCCCCCAAGCCCCTGGAAAACACCGAAGCCGCTCAGGCC
CAGCCCAGCGGCAGCAAGTTCAGCCCCGCCATCCCCGTGTCTACCCAGGAAAGCGTCAGCGTCCCCGCCAGCGTGTCCACC
AGCATCTCTAGCATCTCAACCGGCGCCACAGCTTCTGCCCTGGTCAAGGGCAACACCAACCCCATCACCAGCATGTCTGCCC
CTGTGCAAGCCTCTGCCCCAGCCCTGACCAAGTCCCAGACCGACCGGCTGGAAGTGCTCCTGAACCCCAAGGACGAGATCA
GCCTGAACAGCGGCAAGCCCTTCCGGGAGCTGGAAAGCGAGCTGCTGAGCCGGCGGAAGAAGGACCTCCAGCAAATCTACG
CCGAGGAACGGGAGAACTACCTGGGCAAGCTGGAAAGAGAGATCACCCGGTTCTTCGTGGACCGGGGCTTCCTGGAAATCA
AGAGCCCCATCCTGATCCCCCTGGAGTACATCGAGCGGATGGGCATCGACAACGACACCGAGCTGAGCAAGCAGATTTTCCG
GGTGGACAAGAACTTCTGCCTGCGGCCCATGCTGGCCCCCAACCTGTACAACTACCTGCGGAAACTGGATCGCGCTCTGCCC
GACCCCATCAAGATTTTCGAGATCGGCCCCTGCTACCGGAAAGAGAGCGACGGCAAAGAGCACCTGGAAGAGTTTACAATGC
TGAACTTTTGCCAGATGGGCAGCGGCTGCACCAGAGAGAACCTGGAATCCATCATCACCGACTTTCTGAACCACCTGGGGAT
CGACTTCAAGATCGTGGGCGACAGCTGCATGGTGTACGGCGACACCCTGGACGTGATGCACGGCGACCTGGAACTGTCTAG
CGCCGTCGTGGGACCCATCCCTCTGGACCGGGAGTGGGGCATCGATAAGCCCTGGATCGGAGCCGGCTTCGGCCTGGAACG
GCTGCTGAAAGTCAAGCACGACTTTAAGAACATCAAGCGGGCTGCCAGAAGCGAGAGCTACTACAACGGCATCAGCACCAA
CCTGTGATGATAAGGATCCACTAGTCCAGTGTGGTGGAATTGACATTGATTATTGACTAGTTATTAATAGTAATCAATTACGGGGT
CATTAGTTCATAGCCCATATATGGAGTTCCGCGTTACATAACTTACGGTAAATGGCCCGCCTGGCTGACCGCCCAACGACCCCC
GCCCATTGACGTCAATAATGACGTATGTTCCCATAGTAACGCCAATAGGGACTTTCCATTGACGTCAATGGGTGGAGTATTTACG
GTAAACTGCCCACTTGGCAGTACATCAAGTGTATCATATGCCAAGTACGCCCCCTATTGACGTCAATGACGGTAAATGGCCCGCC
TGGCATTATGCCCAGTACATGACCTTATGGGACTTTCCTACTTGGCAGTACATCTACGTATTAGTCATCGCTATTACCATGGTCGA
GGTGAGCCCCACGTTCTGCTTCACTCTCCCCATCTCCCCCCCCTCCCCACCCCCAATTTTGTATTTATTTATTTTTTAATTATTTTG
TGCAGCGATGGGGGCGGGGGGGGGGGGGGGCGCGCGCCAGGCGGGGCGGGGCGGGGCGAGGGGCGGGGCGGGGCGAGGC
GGAGAGGTGCGGCGGCAGCCAATCAGAGCGGCGCGCTCCGAAAGTTTCCTTTTATGGCGAGGCGGCGGCGGCGGCGGCCCT
ATAAAAAGCGAAGCGCGCGGCGGGCGGGAGTCGCTGCGTTGCCTTCGCCCCGTGCCCCGCTCCGCGCCGCCTCGCGCCGCC
CGCCCCGGCTCTGACTGACCGCGTTACTCCCACAGGTGAGCGGGCGGGACGGCCCTTCTCCTCCGGGCTGTAATTAGCGCTTG
GTTTAATGACGGCTCGTTTCTTTTCTGTGGCTGCGTGAAAGCCTTAAAGGGCTCCGGGAGGGCCCTTTGTGCGGGGGGGAGCG
GCTCGGGGGGTGCGTGCGTGTGTGTGTGCGTGGGGAGCGCCGCGTGCGGCCCGCGCTGCCCGGCGGCTGTGAGCGCTGCG
GGCGCGGCGCGGGGCTTTGTGCGCTCCGCGTGTGCGCGAGGGGAGCGCGGCCGGGGGCGGTGCCCCGCGGTGCGGGGGG
GCTGCGAGGGGAACAAAGGCTGCGTGCGGGGTGTGTGCGTGGGGGGGTGAGCAGGGGGTGTGGGCGCGGCGGTCGGGCTG
TAACCCCCCCCTGCACCCCCCTCCCCGAGTTGCTGAGCACGGCCCGGCTTCGGGTGCGGGGCTCCGTACGGGGCGTGGCGC
GGGGCTCGCCGTGCCGGGCGGGGGGTGGCGGCAGGTGGGGGTGCCGGGCGGGGCGGGGCCGCCTCGGGCCGGGGAGGG
CTCGGGGGAGGGGCGCGGCGGCCCCCGGAGCGCCGGCGGCTGTCGAGGCGCGGCGAGCCGCAGCCATTGCCTTTTATGGTA
ATCGTGCGAGAGGGCGCAGGGACTTCCTTTGTCCCAAATCTGTGCGGAGCCGAAATCTGGGAGGCGCCGCCGCACCCCCTCTA
GCGGGCGCGGGGCGAAGCGGTGCGGCGCCGGCAGGAAGGAAATGGGCGGGGAGGGCCTTCGTGCGTCGCCGCGCCGCCGT
CCCCTTCTCCCTCTCCAGCCTCGGGGCTGTCCGCGGGGGGACGGCTGCCTTCGGGGGGGACGGGGCAGGGCGGGGTTCGGC
TTCTGGCGTGTGACCGGCGGCTCTAGAGCCTCTGCTAACCATGTTCATGCCTTCTTCTTTTTCCTACAGCTCCTGGGCAACGTGC
TGGTTATTGTGCTGTCTCATCATTTTGGCAAAGAATTCGCCACCATGGTGAGCAAGGGCGAGGAGGATAACATGGCCATCATCA
AGGAGTTCATGCGCTTCAAGGTGCACATGGAGGGCTCCGTGAACGGCCACGAGTTCGAGATCGAGGGCGAGGGCGAGGGCC
GCCCCTACGAGGGCACCCAGACCGCCAAGCTGAAGGTGACCAAGGGTGGCCCCCTGCCCTTCGCCTGGGACATCCTGTCCC
CTCAGTTCATGTACGGCTCCAAGGCCTACGTGAAGCACCCCGCCGACATCCCCGACTACTTGAAGCTGTCCTTCCCCGAGGG
CTTCAAGTGGGAGCGCGTGATGAACTTCGAGGACGGCGGCGTGGTGACCGTGACCCAGGACTCCTCCCTGCAGGACGGCGA
GTTCATCTACAAGGTGAAGCTGCGCGGCACCAACTTCCCCTCCGACGGCCCCGTAATGCAGAAGAAGACCATGGGCTGGGA
GGCCTCCTCCGAGCGGATGTACCCCGAGGACGGCGCCCTGAAGGGCGAGATCAAGCAGAGGCTGAAGCTGAAGGACGGCG
GCCACTACGACGCTGAGGTCAAGACCACCTACAAGGCCAAGAAGCCCGTGCAGCTGCCCGGCGCCTACAACGTCAACATCA
AGTTGGACATCACCTCCCACAACGAGGACTACACCATCGTGGAACAGTACGAACGCGCCGAGGGCCGCCACTCCACCGGCG
GCATGGACGAGCTGTACGCGTCTGGAGGCGCCACCAATTTCAGCCTGCTGAAACAGGCTGGCGACGTGGAAGAGAACCCTG
GACCTGGACAAAAGTCGGTGAGCAAGGGCGAGGAGCTGTTCACCGGGGTGGTGCCCATCCTGGTCGAGCTGGACGGCGACG
TAAACGGCCACAAGTTCAGCGTGTCCGGCGAGGGCGAGGGCGATGCCACCTACGGCAAGCTGACCCTGAAGTTCATCTGCA
CCACCGGCAAGCTGCCCGTGCCCTGGCCCACCCTCGTGACCACCCTGACCTACGGCGTGCAGTGCTTCAGCCGCTACCCCGA
CCACATGAAGCAGCACGACTTCTTCAAGTCCGCCATGCCCGAAGGCTACGTCCAGGAGCGCACCATCTTCTTCAAGGACGAC
GGCAACTACAAGACCCGCGCCGAGGTGAAGTTCGAGGGCGACACCCTGGTGAACCGCATCGAGCTGAAGGGCATCGACTTC
AAGGAGGACGGCAACATCCTGGGGCACAAGCTGGAGTACAACTACAACAGCCACAGGAGTCTATATCATGGCCGACAAGCA
GAAGAACGGCATCAAGGTGAACTTCAAGATCCGCCACAACATCGAGGACGGCAGCGTGCAGCTCGCCGACCACTACCAGCA
GAACACCCCCATCGGCGACGGCCCCGTGCTGCTGCCCGACAACCACTACCTGAGCACCCAGTCCGCCCTGAGCAAAGACCC
CAACGAGAAGCGCGATCACATGGTCCTGCTGGAGTTCGTGACCGCCGCCGGGATCACTCTCGGCATGGACGAGCTGTACAA
GTAAGAATTCACTCCTCAGGTGCAGGCTGCCTATCAGAAGGTGGTGGCTGGTGTGGCCAATGCCCTGGCTCACAAATACCACTG
AGATCTTTTTCCCTCTGCCAAAAATTATGGGGACATCATGAAGCCCCTTGAGCATCTGACTTCTGGCTAATAAAGGAAATTTATTT
TCATTGCAATAGTGTGTTGGAATTTTTTGTGTCTCTCACTCGGAAGGACATATGGGAGGGGTCGACAATCAACCTCTGGATTACA
AAATTTGTGAAAGATTGACTGGTATTCTTAACTATGTTGCTCCTTTTACGCTATGTGGATACGCTGCTTTAATGCCTTTGTATCATG
CGTTAACTAAACTTGTTTATTGCAGCTTATAATGGTTACAAATAAAGCAATAGCATCACAAATTTCACAAATAAAGCATTTTTTTCAC
TGCATTCTAGTTGTGGTTTGTCCAAACTCATCAATGTATCTTATCATGTCTGGAATTGACTCAAATGATGTCAATTAGTCTATCAGA
AGCTATCTGGTCTCCCTTCCGGGGGACAAGACATCCCTGTTTAATATTTAAACAGCAGTGTTCCCAAACTGGGTTCTTATATCCCT
TGCTCTGGTCAACCAGGTTGCAGGGTTTCCTGTCCTCACAGGAACGAAGTCCCTAAAGAAACAGTGGCAGCCAGGTTTAGCCCC
GGAATTGACTGGATTCCTTTTTTAGGGCCCATTGGTATGGCTTTTTCCCCGTATCCCCCCAGGTGTCTGCAGGCTCAAAGAGCAG
CGAGAAGCGTTCAGAGGAAAGCGATCCCGTGCCACCTTCCCCGTGCCCGGGCTGTCCCCGCACGCTGCCGGCTCGGGGATGC
GGGGGGAGCGCCGGACCGGAGCGGAGCCCCGGGCGGCTCGCTGCTGCCCCCTAGCGGGGGAGGGACGTAATTACATCCCTG
GGGGCTTTGGGGGGGGGCTGTCCCTGATATCTATAACAAGAAAATATATATATAATAAGTTATCACGTAAGTAGAACATGAAATAA
CAATATAATTATCGTATGAGTTAAATCTTAAAAGTCACGTAAAAGATAATCATGCGTCATTTTGACTCACGCGGTCGTTATAGTTCA
AAATCAGTGACACTTACCGCATTGACAAGCACGCCTCACGGGAGCTCCAAGCGGCGACTGAGATGTCCTAAATGCACAGCGACG

GATTCGCGCTATTTAGAAAGAGAGAGCAATATTTCAAGAATGCATGCGTCAATTTTACGCAGACTATCTTTCTAGGGTTAATCTAG
CTGCATCAGGATCATATCGTCGGGTCTTTTTTCCGGCTCAGTCATCGCCCAAGCTGGCGCTATCTGGGCATCGGGGAGGAAGAA
GCCCGTGCCTTTTCCCGCGAGGTTGAAGCGGCATGGAAAGAGTTTGCCGAGGATGACTGCTGCTGCATTGACGTTGAGCGAAA
ACGCACGTTTACCATGATGATTCGGGAAGGTGTGGCCATGCACGCCTTTAACGGTGAACTGTTCGTTCAGGCCACCTGGGATAC
CAGTTCGTCGCGGCTTTTCCGGACACAGTTCCGGATGGTCAGCCCGAAGCGCATCAGCAACCCGAACAATACCGGCGACAGCC
GGAACTGCCGTGCCGGTGTGCAGATTAATGACAGCGGTGCGGCGCTGGGATATTACGTCAGCGAGGACGGGTATCCTGGCTG
GATGCCGCAGAAATGGACATGGATACCCCGTGAGTTACCCGGCGGGCGCGCTTGGCGTAATCATGGTCATAGCTGTTTCCTGT
GTGAAATTGTTATCCGCTCACAATTCCACACAACATACGAGCCGGAAGCATAAAGTGTAAAGCCTGGGGTGCCTAATGAGTGAG
CTAACTCACATTAATTGCGTTGCGCTCACTGCCCGCTTTCCAGTCGGGAAACCTGTCGTGCCAGCTGCATTAATGAATCGGCCAA
CGCGCGGGGAGAGGCGGTTTGCGTATTGGGCGCTCTTCCGCTTCCTCGCTCACTGACTCGCTGCGCTCGGTCGTTCGGCTGCG
GCGAGCGGTATCAGCTCACTCAAAGGCGGTAATACGGTTATCCACAGAATCAGGGGATAACGCAGGAAAGAACATGTGAGCAAA
AGGCCAGCAAAAGGCCAGGAACCGTAAAAAGGCCGCGTTGCTGGCGTTTTTCCATAGGCTCCGCCCCCCTGACGAGCATCACA
AAAATCGACGCTCAAGTCAGAGGTGGCGAAACCCGACAGGACTATAAAGATACCAGGCGTTTCCCCCTGGAAGCTCCCTCGTGC
GCTCTCCTGTTCCGACCCTGCCGCTTACCGGATACCTGTCCGCCTTTCTCCCTTCGGGAAGCGTGGCGCTTTCTCATAGCTCAC
GCTGTAGGTATCTCAGTTCGGTGTAGGTCGTTCGCTCCAAGCTGGGCTGTGTGCACGAACCCCCCGTTCAGCCCGACCGCTGC
GCCTTATCCGGTAACTATCGTCTTGAGTCCAACCCGGTAAGACACGACTTATCGCCACTGGCAGCAGCCACTGGTAACAGGATT
AGCAGAGCGAGGTATGTAGGCGGTGCTACAGAGTTCTTGAAGTGGTGGCCTAACTACGGCTACACTAGAAGGACAGTATTTGGT
ATCTGCGCTCTGCTGAAGCCAGTTACCTTCGGAAAAAGAGTTGGTAGCTCTTGATCCGGCAAACAAACCACCGCTGGTAGCGGT
GGTTTTTTTGTTTGCAAGCAGCAGATTACGCGCAGAAAAAAAGGATCTCAAGAAGATCCTTTGATCTTTTCTACGGGGTCTGACG
CTCAGTGGAACGAAAACTCACGTTAAGGGATTTTGGTCATGAGATTATCAAAAAGGATCTTCACCTAGATCCTTTTAAATTAAAAAT
GAAGTTTTAAATCAATCTAAAGTATATATGAGTAAACTTGGTCTGACAGTTACCAATGCTTAATCAGTGAGGCACCTATCTCAGCG
ATCTGTCTATTTCGTTCATCCATAGTTGCCTGACTCCCCGTCGTGTAGATAACTACGATACGGGAGGGCTTACCATCTGGCCCCA
GTGCTGCAATGATACCGCGAGACCCACGCTCACCGGCTCCAGATTTATCAGCAATAAACCAGCCAGCCGGAAGGGCCGAGCGC
AGAAGTGGTCCTGCAACTTTATCCGCCTCCATCCAGTCTATTAATTGTTGCCGGGAAGCTAGAGTAAGTAGTTCGCCAGTTAATA
GTTTGCGCAACGTTGTTGCCATTGCTACAGGCATCGTGGTGTCACGCTCGTCGTTTGGTATGGCTTCATTCAGCTCCGGTTCCCA
ACGATCAAGGCGAGTTACATGATCCCCCATGTTGTGCAAAAAAGCGGTTAGCTCCTTCGGTCCTCCGATCGTTGTCAGAAGTAAG
TTGGCCGCAGTGTTATCACTCATGGTTATGGCAGCACTGCATAATTCTCTTACTGTCATGCCATCCGTAAGATGCTTTTCTGTGAC
TGGTGAGTACTCAACCAAGTCATTCTGAGAATAGTGTATGCGGCGACCGAGTTGCTCTTGCCCGGCGTCAATACGGGATAATAC
CGCGCCACATAGCAGAACTTTAAAAGTGCTCATCATTGGAAAACGTTCTTCGGGGCGAAAACTCTCAAGGATCTTACCGCTGTTG
AGATCCAGTTCGATGTAACCCACTCGTGCACCCAACTGATCTTCAGCATCTTTTACTTTCACCAGCGTTTCTGGGTGAGCAAAAA
CAGGAAGGCAAAATGCCGCAAAAAAGGGAATAAGGGCGACACGGAAATGTTGAATACTCAT

2.2.14 Vector 67: Pyl $tRNA_{ACUA}$ PylRS mCherry-P2A-eGFP(150TAGT)

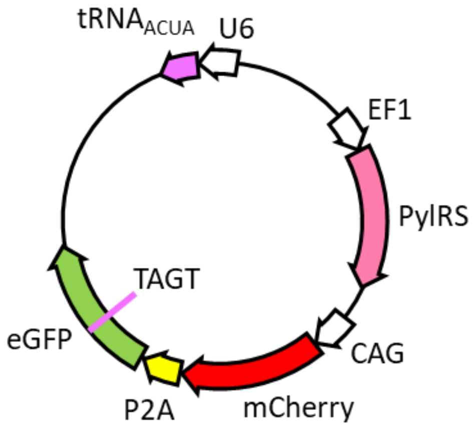

ACTCTTCCTTTTTCAATATTATTGAAGCATTTATCAGGGTTATTGTCTCATGAGCGGATACATATTTGAATGTATTTAGAAAAATAAA
CAAATAGGGGTTCCGCGCACATTTCCCCGAAAAGTGCCACCTAAATTGTAAGCGTTAATATTTTGTTAAAATTCGCGTTAAATTTTT
GTTAAATCAGCTCATTTTTTAACCAATAGGCCGAAATCGGCAAAATCCCTTATAAATCAAAAGAATAGACCGAGATAGGGTTGAGT
GTTGTTCCAGTTTGGAACAAGAGTCCACTATTAAAGAACGTGGACTCCAACGTCAAAGGGCGAAAAACCGTCTATCAGGGCGAT
GGCCCACTACGTGAACCATCACCCTAATCAAGTTTTTTGGGGTCGAGGTGCCGTAAAGCACTAAATCGGAACCCTAAAGGGAGC
CCCCGATTTAGAGCTTGACGGGGAAAGCCGGCGAACGTGGCGAGAAAGGAAGGGAAGAAAGCGAAAGGAGCGGGCGCTAGGG
CGCTGGCAAGTGTAGCGGTCACGCTGCGCGTAACCACCACACCCGCCGCGCTTAATGCGCCGCTACAGGGCGCGTCCCATTC
GCCATTCAGGCTGCGCAACTGTTGGGAAGGGCGATCGGTGCGGGCCTCTTCGCTATTACGCCAGCTGGCGAAAGGGGGATGT
GCTGCAAGGCGATTAAGTTGGGTAACGCCAGGGTTTTCCCAGTCACGACGTTGTAAAACGACGGCCAGTGAGCGCGCCTCGTT
CATTCACGTTTTTGAACCCGTGGAGGACGGGCAGACTCGCGGTGCAAATGTGTTTTACAGCGTGATGGAGCAGATGAAGATGCT
CGACACGCTGCAGAACACGCAGCTAGATTAACCCTAGAAAGATAATCATATTGTGACGTACGTTAAAGATAATCATGCGTAAAATT
GACGCATGTGTTTTATCGGTCTGTATATCGAGGTTTATTTATTAATTTGAATAGATATTAAGTTTTATTATATTTACACTTACATACTA
ATAATAAATTCAACAAACAATTTATTTATGTTTATTTATTTATTAAAAAAAAACAAAAACTCAAAATTTCTTCTATAAAGTAACAAAACTT
TTATGAGGGACAGCCCCCCCCCAAAGCCCCCAGGGATGTAATTACGTCCCTCCCCCGCTAGGGGGCAGCAGCGAGCCGCCCG
GGGCTCCGCTCCGGTCCGGCGCTCCCCCCGCATCCCCGAGCCGGCAGCGTGCGGGGACAGCCCGGGCACGGGGAAGGTGG
CACGGGATCGCTTTCCTCTGAACGCTTCTCGCTGCTCTTTGAGCCTGCAGACACCTGGGGGGATACGGGGAAAAGGCCTAAAAA
CCGCACTTGTC**CGGAAACCCCGGGAATCTAACCCGGCTGAACGGATTTAGTAGTCCATTCGATCTACATGATCAGGTTTCC**GG
TGTTTCGTCCTTTCCACAAGATATATAAAGCCAAGAAATCGAAATACTTTCAAGTTACGGTAAGCATATGATAGTCCATTTTAAAAC
ATAATTTTAAAACTGCAAACTACCCAAGAAATTATTACTTTCTACGTCACGTATTTTGTACTAATATCTTTGTGTTTACAGTCAAATT
AATTCTAATTATCTCTCTAACAGCCTTGTATCGTATATGCAAATATGAAGGAATCATGGGAAATAGGCCCTCTTCCTGCCCAACCG
GTTGCGATCGCTCCGGTGCCCGTCAGTGGGCAGAGCGCACATCGCCCACAGTCCCCGAGAAGTTGGGGGGAGGGGTCGGCAA
TTGAACGGGTGCCTAGAGAAGGTGGCGCGGGGTAAACTGGGAAAGTGATGTCGTGTACTGGCTCCGCCTTTTTCCCGAGGGTG
GGGGAGAACCGTATATAAGTGCAGTAGTCGCCGTGAACGTTCTTTTTCGCAACGGGTTTGCCGCCAGAACACAGCTGAAGCTTC

GAGGGGCTCGCATCTCTCCTTCACGCGCCCGCCGCCCTACCTGAGGCCGCCATCCACGCCGGTTGAGTCGCGTTCTGCCGCCT
CCCGCCTGTGGTGCCTCCTGAACTGCGTCCGCCGTCTAGGTAAGTTTAAAGCTCAGGTCGAGACCGGGCCTTTGTCCGGCGCT
CCCTTGGAGCCTACCTAGACTCAGCCGGCTCTCCACGCTTTGCCTGACCCTGCTTGCTCAACTCTACGTCTTTGTTTCGTTTTCT
GTTCTGCGCCGTTACAGATCCAAGCTGTGACCGGCGCCTACTCTACAGATAGCGTTTAAACTTACGCTTGCCACCATGGCTAGC
GACTACAAGGACGACGACGACAAGGACAAGAAGCCCCTGAACACCCTGATCAGCGCCACAGGACTGTGGATGTCCAGAACC
GGCACCATCCACAAGATCAAGCACCACGAGGTGTCCCGGTCCAAAATCTACATCGAGATGGCCTGCGGCGATCACCTGGTCG
TCAACAACAGCAGAAGCAGCCGGACAGCCAGAGCCCTGCGGCACCACAAGTACAGAAAGACCTGCAAGCGGTGCAGAGTGT
CCGACGAGGACCTGAACAAGTTCCTGACCAAGGCCAACGAGGACCAGACCAGCGTGAAAGTGAAGGTGGTGTCCGCCCCCA
CCCGGACCAAGAAAGCCATGCCCAAGAGCGTGGCCAGAGCCCCCAAGCCCCTGGAAAACACCGAAGCCGCTCAGGCCCAG
CCCAGCGGCAGCAAGTTCAGCCCCGCCATCCCCGTGTCTACCCAGGAAAGCGTCAGCGTCCCCGCCAGCGTGTCCACCAGC
ATCTCTAGCATCTCAACCGGCGCCACAGCTTCTGCCCTGGTCAAGGGCAACACCAACCCCATCACCAGCATGTCTGCCCCTGT
GCAAGCCTCTGCCCCAGCCCTGACCAAGTCCCAGACCGACCGGCTGGAAGTGCTCCTGAACCCCAAGGACGAGATCAGCCT
GAACAGCGGCAAGCCCTTCCGGGAGCTGGAAAGCGAGCTGCTGAGCCGGCGGAAGAAGGACCTCCAGCAAATCTACGCCG
AGGAACGGGAGAACTACCTGGGCAAGCTGGAAAGAGAGATCACCCGGTTCTTCGTGGACCGGGGCTTCCTGGAAATCAAGA
GCCCCATCCTGATCCCCCTGGAGTACATCGAGCGGATGGGCATCGACAACGACACCGAGCTGAGCAAGCAGATTTTCCGGGT
GGACAAGAACTTCTGCCTGCGGCCCATGCTGGCCCCCAACCTGTACAACTACCTGCGGAAACTGGATCGCGCTCTGCCCGAC
CCCATCAAGATTTTCGAGATCGGCCCCTGCTACCGGAAAGAGAGCGACGGCAAAGAGCACCTGGAAGAGTTTACAATGCTGA
ACTTTTGCCAGATGGGCAGCGGCTGCACCAGAGAGAACCTGGAATCCATCATCACCGACTTTCTGAACCACCTGGGGATCGA
CTTCAAGATCGTGGGCGACAGCTGCATGGTGTACGGCGACACCCTGGACGTGATGCACGGCGACCTGGAACTGTCTAGCGC
CGTCGTGGGACCCATCCCTCTGGACCGGGAGTGGGGCATCGATAAGCCCTGGATCGGAGCCGGCTTCGGCCTGGAACGGCT
GCTGAAAGTCAAGCACGACTTTAAGAACATCAAGCGGGCTGCCAGAAGCGAGAGCTACTACAACGGCATCAGCACCAACCT
GTGATGATAAGGATCCACTAGTCCAGTGTGGTGGAATTGACATTGATTATTGACTAGTTATTAATAGTAATCAATTACGGGGTCAT
TAGTTCATAGCCCATATATGGAGTTCCGCGTTACATAACTTACGGTAAATGGCCCGCCTGGCTGACCGCCCAACGACCCCCGCC
CATTGACGTCAATAATGACGTATGTTCCCATAGTAACGCCAATAGGGACTTTCCATTGACGTCAATGGGTGGAGTATTTACGGTA
AACTGCCCACTTGGCAGTACATCAAGTGTATCATATGCCAAGTACGCCCCCTATTGACGTCAATGACGGTAAATGGCCCGCCTG
GCATTATGCCCAGTACATGACCTTATGGGACTTTCCTACTTGGCAGTACATCTACGTATTAGTCATCGCTATTACCATGGTCGAGG
TGAGCCCCACGTTCTGCTTCACTCTCCCCATCTCCCCCCCCTCCCCACCCCCAATTTTGTATTTATTTATTTTTTAATTATTTTGTG
CAGCGATGGGGGCGGGGGGGGGGGGGGCGCGCGCCAGGCGGGGCGGGGCGGGGCGAGGGGCGGGGCGGGGCGAGGCG
GAGAGGTGCGGCGGCAGCCAATCAGAGCGGCGCGCTCCGAAAGTTTCCTTTTATGGCGAGGCGGCGGCGGCGGCGGCCCTAT
AAAAAGCGAAGCGCGCGGCGGGCGGGAGTCGCTGCGTTGCCTTCGCCCCGTGCCCCGCTCCGCGCCGCCTCGCGCCGCCCG
CCCCGGCTCTGACTGACCGCGTTACTCCCACAGGTGAGCGGGCGGGACGGCCCTTCTCCTCCGGGCTGTAATTAGCGCTTGGT
TTAATGACGGCTCGTTTCTTTTCTGTGGCTGCGTGAAAGCCTTAAAGGGCTCCGGGAGGGCCCTTTGTGCGGGGGGGAGCGGC
TCGGGGGGTGCGTGCGTGTGTGTGTGCGTGGGGAGCGCCGCGTGCGGCCCGCGCTGCCCGGCGGCTGTGAGCGCTGCGGG
CGCGGCGCGGGGCTTTGTGCGCTCCGCGTGTGCGCGAGGGGAGCGCGGCCGGGGGCGGTGCCCCGCGGTGCGGGGGGGCT
GCGAGGGGAACAAAGGCTGCGTGCGGGGTGTGTGCGTGGGGGGGTGAGCAGGGGGTGTGGGCGCGGCGGTCGGGCTGTAA
CCCCCCCCTGCACCCCCCTCCCCGAGTTGCTGAGCACGGCCCGGCTTCGGGTGCGGGGCTCCGTACGGGGCGTGGCGCGGG
GCTCGCCGTGCCGGGCGGGGGGTGGCGGCAGGTGGGGGTGCCGGGCGGGGCGGGGCCGCCTCGGGCCGGGGAGGGCTCG
GGGGAGGGGCGCGGCGGCCCCCGGAGCGCCGGCGGCTGTCGAGGCGCGGCGAGCCGCAGCCATTGCCTTTTATGGTAATCG
TGCGAGAGGGCGCAGGGACTTCCTTTGTCCCAAATCTGTGCGGAGCCGAAATCTGGGAGGCGCCGCCGCACCCCCTCTAGCG
GGCGCGGGGCGAAGCGGTGCGGCGCCGGCAGGAAGGAAATGGGCGGGGAGGGCCTTCGTGCGTCGCCGCGCCGCCGTCCC
CTTCTCCCTCTCCAGCCTCGGGGCTGTCCGCGGGGGGACGGCTGCCTTCGGGGGGGACGGGGCAGGGCGGGGTTCGGCTTC
TGGCGTGTGACCGGCGGCTCTAGAGCCTCTGCTAACCATGTTCATGCCTTCTTCTTTTTCCTACAGCTCCTGGGCAACGTGCTG
GTTATTGTGCTGTCTCATCATTTTGGCAAAGAATTCGCCACCATGGTGAGCAAGGGCGAGGAGGATAACATGGCCATCATCAA
GGAGTTCATGCGCTTCAAGGTGCACATGGAGGGCTCCGTGAACGGCCACGAGTTCGAGATCGAGGGCGAGGGCGAGGGCC
GCCCCTACGAGGGCACCCAGACCGCCAAGCTGAAGGTGACCAAGGGTGGCCCCCTGCCCTTCGCCTGGGACATCCTGTCCC
CTCAGTTCATGTACGGCTCCAAGGCCTACGTGAAGCACCCCGCCGACATCCCCGACTACTTGAAGCTGTCCTTCCCCGAGGG
CTTCAAGTGGGAGCGCGTGATGAACTTCGAGGACGGCGGCGTGGTGACCGTGACCCAGGACTCCTCCCTGCAGGACGGCGA
GTTCATCTACAAGGTGAAGCTGCGCGGCACCAACTTCCCCTCCGACGGCCCCGTAATGCAGAAGAAGACCATGGGCTGGGA
GGCCTCCTCCGAGCGGATGTACCCCGAGGACGGCGCCCTGAAGGGCGAGATCAAGCAGAGGCTGAAGCTGAAGGACGGCG
GCCACTACGACGCTGAGGTCAAGACCACCTACAAGGCCAAGAAGCCCGTGCAGCTGCCCGGCGCCTACAACGTCAACATCA
AGTTGGACATCACCTCCCACAACGAGGACTACACCATCGTGGAACAGTACGAACGCGCCGAGGGCCGCCACTCCACCGGCG
GCATGGACGAGCTGTACGCGTCTGGAGGCGCCACCAATTTCAGCCTGCTGAAACAGGCTGGCGACGTGGAAGAGAACCCTG
GACCTGGACAAAAGTCGGTGAGCAAGGGCGAGGAGCTGTTCACCGGGGTGGTGCCCATCCTGGTCGAGCTGGACGGCGACG
TAAACGGCCACAAGTTCAGCGTGTCCGGCGAGGGCGAGGGCGATGCCACCTACGGCAAGCTGACCCTGAAGTTCATCTGCA
CCACCGGCAAGCTGCCCGTGCCCTGGCCCACCCTCGTGACCACCCTGACCTACGGCGTGCAGTGCTTCAGCCGCTACCCCGA
CCACATGAAGCAGCACGACTTCTTCAAGTCCGCCATGCCCGAAGGCTACGTCCAGGAGCGCACCATCTTCTTCAAGGACGAC
GGCAACTACAAGACCCGCGCCGAGGTGAAGTTCGAGGGCGACACCCTGGTGAACCGCATCGAGCTGAAGGGCATCGACTTC
AAGGAGGACGGCAACATCCTGGGGCACAAGCTGGAGTACAACTACAACAGCCACTAGTGTCTATATCATGGCCGACAAGCA
GAAGAACGGCATCAAGGTGAACTTCAAGATCCGCCACAACATCGAGGACGGCAGCGTGCAGCTCGCCGACCACTACCAGCA
GAACACCCCCATCGGCGACGGCCCCGTGCTGCTGCCCGACAACCACTACCTGAGCACCCAGTCCGCCCTGAGCAAAGACCC
CAACGAGAAGCGCGATCACATGGTCCTGCTGGAGTTCGTGACCGCCGCCGGGATCACTCTCGGCATGGACGAGCTGTACAA
GTAAGAATTCACTCCTCAGGTGCAGGCTGCCTATCAGAAGGTGGTGGCTGGTGTGGCCAATGCCCTGGCTCACAAATACCACTG
AGATCTTTTTCCCTCTGCCAAAAATTATGGGGACATCATGAAGCCCCTTGAGCATCTGACTTCTGGCTAATAAAGGAAATTTATTT
TCATTGCAATAGTGTGTTGGAATTTTTTGTGTCTCTCACTCGGAAGGACATATGGGAGGGGTCGACAATCAACCTCTGGATTACA
AAATTTGTGAAAGATTGACTGGTATTCTTAACTATGTTGCTCCTTTTACGCTATGTGGATACGCTGCTTTAATGCCTTTGTATCATG
CGTTAACTAAACTTGTTTATTGCAGCTTATAATGGTTACAAATAAAGCAATAGCATCACAAATTTCACAAATAAAGCATTTTTTTCAC
TGCATTCTAGTTGTGGTTTGTCCAAACTCATCAATGTATCTTATCATGTCTGGAATTGACTCAAATGATGTCAATTAGTCTATCAGA
AGCTATCTGGTCTCCCTTCCGGGGGACAAGACATCCCTGTTTAATATTTAAACAGCAGTGTTCCCAAACTGGGTTCTTATATCCCT
TGCTCTGGTCAACCAGGTTGCAGGGTTTCCTGTCCTCACAGGAACGAAGTCCCTAAAGAAACAGTGGCAGCCAGGTTTAGCCCC
GGAATTGACTGGATTCCTTTTTTAGGGCCCATTGGTATGGCTTTTTCCCCGTATCCCCCCAGGTGTCTGCAGGCTCAAAGAGCAG
CGAGAAGCGTTCAGAGGAAAGCGATCCCGTGCCACCTTCCCCGTGCCCGGGCTGTCCCCGCACGCTGCCGGCTCGGGGATGC
GGGGGGAGCGCCGGACCGGAGCGGAGCCCCGGGCGGCTCGCTGCTGCCCCCTAGCGGGGGAGGGACGTAATTACATCCCTG
GGGGCTTTGGGGGGGGGCTGTCCCTGATATCTATAACAAGAAAATATATATATAATAAGTTATCACGTAAGTAGAACATGAAATAA
CAATATAATTATCGTATGAGTTAAATCTTAAAAGTCACGTAAAAGATAATCATGCGTCATTTTGACTCACGCGGTCGTTATAGTTCA

AAATCAGTGACACTTACCGCATTGACAAGCACGCCTCACGGGAGCTCCAAGCGGCGACTGAGATGTCCTAAATGCACAGCGACG
GATTCGCGCTATTTAGAAAGAGAGAGCAATATTTCAAGAATGCATGCGTCAATTTTACGCAGACTATCTTTCTAGGGTTAATCTAG
CTGCATCAGGATCATATCGTCGGGTCTTTTTTCCGGCTCAGTCATCGCCCAAGCTGGCGCTATCTGGGCATCGGGGAGGAAGAA
GCCCGTGCCTTTTCCCGCGAGGTTGAAGCGGCATGGAAAGAGTTTGCCGAGGATGACTGCTGCTGCATTGACGTTGAGCGAAA
ACGCACGTTTACCATGATGATTCGGGAAGGTGTGGCCATGCACGCCTTTAACGGTGAACTGTTCGTTCAGGCCACCTGGGATAC
CAGTTCGTCGCGGCTTTTCCGGACACAGTTCCGGATGGTCAGCCCGAAGCGCATCAGCAACCCGAACAATACCGGCGACAGCC
GGAACTGCCGTGCCGGTGTGCAGATTAATGACAGCGGTGCGGCGCTGGGATATTACGTCAGCGAGGACGGGTATCCTGGCTG
GATGCCGCAGAAATGGACATGGATACCCCGTGAGTTACCCGGCGGGCGCGCTTGGCGTAATCATGGTCATAGCTGTTTCCTGT
GTGAAATTGTTATCCGCTCACAATTCCACACAACATACGAGCCGGAAGCATAAAGTGTAAAGCCTGGGGTGCCTAATGAGTGAG
CTAACTCACATTAATTGCGTTGCGCTCACTGCCCGCTTTCCAGTCGGGAAACCTGTCGTGCCAGCTGCATTAATGAATCGGCCAA
CGCGCGGGGAGAGGCGGTTTGCGTATTGGGCGCTCTTCCGCTTCCTCGCTCACTGACTCGCTGCGCTCGGTCGTTCGGCTGCG
GCGAGCGGTATCAGCTCACTCAAAGGCGGTAATACGGTTATCCACAGAATCAGGGGATAACGCAGGAAAGAACATGTGAGCAAA
AGGCCAGCAAAAGGCCAGGAACCGTAAAAAGGCCGCGTTGCTGGCGTTTTTCCATAGGCTCCGCCCCCCTGACGAGCATCACA
AAAATCGACGCTCAAGTCAGAGGTGGCGAAACCCGACAGGACTATAAAGATACCAGGCGTTTCCCCCTGGAAGCTCCCTCGTGC
GCTCTCCTGTTCCGACCCTGCCGCTTACCGGATACCTGTCCGCCTTTCTCCCTTCGGGAAGCGTGGCGCTTTCTCATAGCTCAC
GCTGTAGGTATCTCAGTTCGGTGTAGGTCGTTCGCTCCAAGCTGGGCTGTGTGCACGAACCCCCCGTTCAGCCCGACCGCTGC
GCCTTATCCGGTAACTATCGTCTTGAGTCCAACCCGGTAAGACACGACTTATCGCCACTGGCAGCAGCCACTGGTAACAGGATT
AGCAGAGCGAGGTATGTAGGCGGTGCTACAGAGTTCTTGAAGTGGTGGCCTAACTACGGCTACACTAGAAGGACAGTATTTGGT
ATCTGCGCTCTGCTGAAGCCAGTTACCTTCGGAAAAAGAGTTGGTAGCTCTTGATCCGGCAAACAAACCACCGCTGGTAGCGGT
GGTTTTTTTGTTTGCAAGCAGCAGATTACGCGCAGAAAAAAAGGATCTCAAGAAGATCCTTTGATCTTTTCTACGGGGTCTGACG
CTCAGTGGAACGAAAACTCACGTTAAGGGATTTTGGTCATGAGATTATCAAAAAGGATCTTCACCTAGATCCTTTTAAATTAAAAAT
GAAGTTTTAAATCAATCTAAAGTATATATGAGTAAACTTGGTCTGACAGTTACCAATGCTTAATCAGTGAGGCACCTATCTCAGCG
ATCTGTCTATTTCGTTCATCCATAGTTGCCTGACTCCCCGTCGTGTAGATAACTACGATACGGGAGGGCTTACCATCTGGCCCCA
GTGCTGCAATGATACCGCGAGACCCACGCTCACCGGCTCCAGATTTATCAGCAATAAACCAGCCAGCCGGAAGGGCCGAGCGC
AGAAGTGGTCCTGCAACTTTATCCGCCTCCATCCAGTCTATTAATTGTTGCCGGGAAGCTAGAGTAAGTAGTTCGCCAGTTAATA
GTTTGCGCAACGTTGTTGCCATTGCTACAGGCATCGTGGTGTCACGCTCGTCGTTTGGTATGGCTTCATTCAGCTCCGGTTCCCA
ACGATCAAGGCGAGTTACATGATCCCCCATGTTGTGCAAAAAAGCGGTTAGCTCCTTCGGTCCTCCGATCGTTGTCAGAAGTAAG
TTGGCCGCAGTGTTATCACTCATGGTTATGGCAGCACTGCATAATTCTCTTACTGTCATGCCATCCGTAAGATGCTTTTCTGTGAC
TGGTGAGTACTCAACCAAGTCATTCTGAGAATAGTGTATGCGGCGACCGAGTTGCTCTTGCCCGGCGTCAATACGGGATAATAC
CGCGCCACATAGCAGAACTTTAAAAGTGCTCATCATTGGAAAACGTTCTTCGGGGCGAAAACTCTCAAGGATCTTACCGCTGTTG
AGATCCAGTTCGATGTAACCCACTCGTGCACCCAACTGATCTTCAGCATCTTTTACTTTCACCAGCGTTTCTGGGTGAGCAAAAA
CAGGAAGGCAAAATGCCGCAAAAAAGGGAATAAGGGCGACACGGAAATGTTGAATACTCAT

2.2.15 Vector 68: Pyl tRNA$_{CCUA}$ PylRS mCherry-P2A-eGFP(150TAGG)

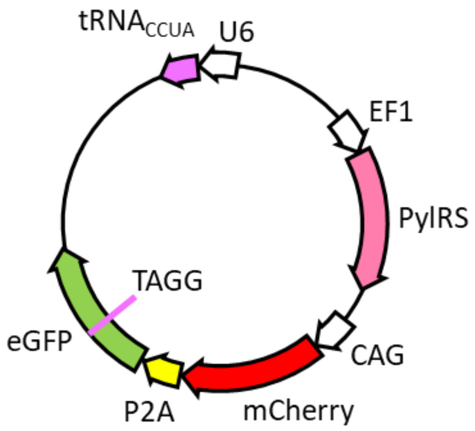

ACTCTTCCTTTTTCAATATTATTGAAGCATTTATCAGGGTTATTGTCTCATGAGCGGATACATATTTGAATGTATTTAGAAAAATAAA
CAAATAGGGGTTCCGCGCACATTTCCCCGAAAAGTGCCACCTAAATTGTAAGCGTTAATATTTTGTTAAAATTCGCGTTAAATTTTT
GTTAAATCAGCTCATTTTTTAACCAATAGGCCGAAATCGGCAAAATCCCTTATAAATCAAAAGAATAGACCGAGATAGGGTTGAGT
GTTGTTCCAGTTTGGAACAAGAGTCCACTATTAAAGAACGTGGACTCCAACGTCAAAGGGCGAAAAACCGTCTATCAGGGCGAT
GGCCCACTACGTGAACCATCACCCTAATCAAGTTTTTTGGGGTCGAGGTGCCGTAAAGCACTAAATCGGAACCCTAAAGGGAGC
CCCCGATTTAGAGCTTGACGGGGAAAGCCGGCGAACGTGGCGAGAAAGGAAGGGAAGAAAGCGAAAGGAGCGGGCGCTAGGG
CGCTGGCAAGTGTAGCGGTCACGCTGCGCGTAACCACCACACCCGCCGCGCTTAATGCGCCGCTACAGGGCGCGTCCCATTC
GCCATTCAGGCTGCGCAACTGTTGGGAAGGGCGATCGGTGCGGGCCTCTTCGCTATTACGCCAGCTGGCGAAAGGGGGATGT
GCTGCAAGGCGATTAAGTTGGGTAACGCCAGGGTTTTCCCAGTCACGACGTTGTAAAACGACGGCCAGTGAGCGCGCCTCGTT
CATTCACGTTTTTGAACCCGTGGAGGACGGGCAGACTCGCGGTGCAAATGTGTTTTACAGCGTGATGGAGCAGATGAAGATGCT
CGACACGCTGCAGAACACGCAGCTAGATTAACCCTAGAAAGATAATCATATTGTGACGTACGTTAAAGATAATCATGCGTAAAATT
GACGCATGTGTTTTATCGGTCTGTATATCGAGGTTTATTTATTAATTTGAATAGATATTAAGTTTTATTATATTTACACTTACATACTA
ATAATAAATTCAACAAACAATTTATTTATGTTTATTTATTTATTAAAAAAAAACAAAAACTCAAAATTTCTTCTATAAAGTAACAAAACTT
TTATGAGGGACAGCCCCCCCCCAAAGCCCCCAGGGATGTAATTACGTCCCTCCCCCGCTAGGGGGCAGCAGCGAGCCGCCCG
GGGCTCCGCTCCGGTCCGGCGCTCCCCCCGCATCCCCGAGCCGGCAGCGTGCGGGGACAGCCCGGGCACGGGGAAGGTGG
CACGGGATCGCTTTCCTCTGAACGCTTCTCGCTGCTCTTTGAGCCTGCAGACACCTGGGGGGATACGGGGAAAAGGCCTAAAAA
CCGCACTTGTC**CGGAAACCCCGGGAATCTAACCCGGCTGAACGGATTTAGGAGTCCATTCGATCTACATGATCAGGTTTCC**GG
TGTTTCGTCCTTTCCACAAGATATATAAAGCCAAGAAATCGAAATACTTTCAAGTTACGGTAAGCATATGATAGTCCATTTTAAAAC
ATAATTTTAAAACTGCAAACTACCCAAGAAATTATTACTTTCTACGTCACGTATTTTGTACTAATATCTTTGTGTTTACAGTCAAATT
AATTCTAATTATCTCTCTAACAGCCTTGTATCGTATATGCAAATATGAAGGAATCATGGGAAATAGGCCCTCTTCCTGCCCAACCG
GTTGCGATCGCTCCGGTGCCCGTCAGTGGGCAGAGCGCACATCGCCCACAGTCCCCGAGAAGTTGGGGGGAGGGGTCGGCAA
TTGAACGGGTGCCTAGAGAAGGTGGCGCGGGGTAAACTGGGAAAGTGATGTCGTGTACTGGCTCCGCCTTTTTCCCGAGGGTG

GGGGAGAACCGTATATAAGTGCAGTAGTCGCCGTGAACGTTCTTTTTCGCAACGGGTTTGCCGCCAGAACACAGCTGAAGCTTC
GAGGGGCTCGCATCTCTCCTTCACGCGCCCGCCGCCCTACCTGAGGCCGCCATCCACGCCGGTTGAGTCGCGTTCTGCCGCCT
CCCGCCTGTGGTGCCTCCTGAACTGCGTCCGCCGTCTAGGTAAGTTTAAAGCTCAGGTCGAGACCGGGCCTTTGTCCGGCGCT
CCCTTGGAGCCTACCTAGACTCAGCCGGCTCTCCACGCTTTGCCTGACCCTGCTTGCTCAACTCTACGTCTTTGTTTCGTTTTCT
GTTCTGCGCCGTTACAGATCCAAGCTGTGACCGGCGCCTACTCTACAGATAGCGTTTAAACTTACGCTTGCCACC**ATGGCTAGC**
GACTACAAGGACGACGACGACAAGGACAAGAAGCCCCTGAACACCCTGATCAGCGCCACAGGACTGTGGATGTCCAGAACC
GGCACCATCCACAAGATCAAGCACCACGAGGTGTCCCGGTCCAAAATCTACATCGAGATGGCCTGCGGCGATCACCTGGTCG
TCAACAACAGCAGAAGCAGCCGGACAGCCAGAGCCCTGCGGCACCACAAGTACAGAAAGACCTGCAAGCGGTGCAGAGTGT
CCGACGAGGACCTGAACAAGTTCCTGACCAAGGCCAACGAGGACCAGACCAGCGTGAAAGTGAAGGTGGTGTCCGCCCCCA
CCCGGACCAAGAAAGCCATGCCCAAGAGCGTGGCCAGAGCCCCCAAGCCCCTGGAAAACACCGAAGCCGCTCAGGCCCAG
CCCAGCGGCAGCAAGTTCAGCCCCGCCATCCCCGTGTCTACCCAGGAAAGCGTCAGCGTCCCCGCCAGCGTGTCCACCAGC
ATCTCTAGCATCTCAACCGGCGCCACAGCTTCTGCCCTGGTCAAGGGCAACACCAACCCCATCACCAGCATGTCTGCCCCTGT
GCAAGCCTCTGCCCCAGCCCTGACCAAGTCCCAGACCGACCGGCTGGAAGTGCTCCTGAACCCCAAGGACGAGATCAGCCT
GAACAGCGGCAAGCCCTTCCGGGAGCTGGAAAGCGAGCTGCTGAGCCGGCGGAAGAAGGACCTCCAGCAAATCTACGCCG
AGGAACGGGAGAACTACCTGGGCAAGCTGGAAAGAGAGATCACCCGGTTCTTCGTGGACCGGGGCTTCCTGGAAATCAAGA
GCCCCATCCTGATCCCCCTGGAGTACATCGAGCGGATGGGCATCGACAACGACACCGAGCTGAGCAAGCAGATTTTCCGGGT
GGACAAGAACTTCTGCCTGCGGCCCATGCTGGCCCCCAACCTGTACAACTACCTGCGGAAACTGGATCGCGCTCTGCCCGAC
CCCATCAAGATTTTCGAGATCGGCCCCTGCTACCGGAAAGAGAGCGACGGCAAAGAGCACCTGGAAGAGTTTACAATGCTGA
ACTTTTGCCAGATGGGCAGCGGCTGCACCAGAGAGAACCTGGAATCCATCATCACCGACTTTCTGAACCACCTGGGGATCGA
CTTCAAGATCGTGGGCGACAGCTGCATGGTGTACGGCGACACCCTGGACGTGATGCACGGCGACCTGGAACTGTCTAGCGC
CGTCGTGGGACCCATCCCTCTGGACCGGGAGTGGGGCATCGATAAGCCCTGGATCGGAGCCGGCTTCGGCCTGGAACGGCT
GCTGAAAGTCAAGCACGACTTTAAGAACATCAAGCGGGCTGCCAGAAGCGAGAGCTACTACAACGGCATCAGCACCAACCT
GTGATGATAAGGATCCACTAGTCCAGTGTGGTGGAATTGACATTGATTATTGACTAGTTATTAATAGTAATCAATTACGGGGTCAT
TAGTTCATAGCCCATATATGGAGTTCCGCGTTACATAACTTACGGTAAATGGCCCGCCTGGCTGACCGCCCAACGACCCCCGCC
CATTGACGTCAATAATGACGTATGTTCCCATAGTAACGCCAATAGGGACTTTCCATTGACGTCAATGGGTGGAGTATTTACGGTA
AACTGCCCACTTGGCAGTACATCAAGTGTATCATATGCCAAGTACGCCCCCTATTGACGTCAATGACGGTAAATGGCCCGCCTG
GCATTATGCCCAGTACATGACCTTATGGGACTTTCCTACTTGGCAGTACATCTACGTATTAGTCATCGCTATTACCATGGTCGAGG
TGAGCCCCACGTTCTGCTTCACTCTCCCCATCTCCCCCCCCTCCCCACCCCCAATTTTGTATTTATTTATTTTTTAATTATTTTGTG
CAGCGATGGGGGCGGGGGGGGGGGGGGCGCGCGCCAGGCGGGGCGGGGCGGGGCGAGGGGCGGGGCGGGGCGAGGCG
GAGAGGTGCGGCGGCAGCCAATCAGAGCGGCGCGCTCCGAAAGTTTCCTTTTATGGCGAGGCGGCGGCGGCGGCGGCCCTAT
AAAAAGCGAAGCGCGCGGCGGGCGGGAGTCGCTGCGTTGCCTTCGCCCCGTGCCCCGCTCCGCGCCGCCTCGCGCCGCCCG
CCCCGGCTCTGACTGACCGCGTTACTCCCACAGGTGAGCGGGCGGGACGGCCCTTCTCCTCCGGGCTGTAATTAGCGCTTGGT
TTAATGACGGCTCGTTTCTTTTCTGTGGCTGCGTGAAAGCCTTAAAGGGCTCCGGGAGGGCCCTTTGTGCGGGGGGGAGCGGC
TCGGGGGGTGCGTGCGTGTGTGTGTGCGTGGGGAGCGCCGCGTGCGGCCCGCGCTGCCCGGCGGCTGTGAGCGCTGCGGG
CGCGGCGCGGGGCTTTGTGCGCTCCGCGTGTGCGCGAGGGGAGCGCGGCCGGGGGCGGTGCCCCGCGGTGCGGGGGGGCT
GCGAGGGGAACAAAGGCTGCGTGCGGGGTGTGTGCGTGGGGGGGTGAGCAGGGGGTGTGGGCGCGGCGGTCGGGCTGTAA
CCCCCCCCTGCACCCCCCTCCCCGAGTTGCTGAGCACGGCCCGGCTTCGGGTGCGGGGCTCCGTACGGGGCGTGGCGCGGG
GCTCGCCGTGCCGGGCGGGGGGTGGCGGCAGGTGGGGGTGCCGGGCGGGGCGGGGCCGCCTCGGGCCGGGGAGGGCTCG
GGGGAGGGGCGCGGCGGCCCCCGGAGCGCCGGCGGCTGTCGAGGCGCGGCGAGCCGCAGCCATTGCCTTTTATGGTAATCG
TGCGAGAGGGCGCAGGGACTTCCTTTGTCCCAAATCTGTGCGGAGCCGAAATCTGGGAGGCGCCGCCGCACCCCCTCTAGCG
GGCGCGGGGCGAAGCGGTGCGGCGCCGGCAGGAAGGAAATGGGCGGGGAGGGCCTTCGTGCGTCGCCGCGCCGCCGTCCC
CTTCTCCCTCTCCAGCCTCGGGGCTGTCCGCGGGGGGACGGCTGCCTTCGGGGGGGACGGGGCAGGGCGGGGTTCGGCTTC
TGGCGTGTGACCGGCGGCTCTAGAGCCTCTGCTAACCATGTTCATGCCTTCTTCTTTTTCCTACAGCTCCTGGGCAACGTGCTG
GTTATTGTGCTGTCTCATCATTTTGGCAAAGAATTCGCCACC**ATGGTGAGCAAGGGCGAGGAGGATAACATGGCCATCATCAA**
GGAGTTCATGCGCTTCAAGGTGCACATGGAGGGCTCCGTGAACGGCCACGAGTTCGAGATCGAGGGCGAGGGCGAGGGCC
GCCCCTACGAGGGCACCCAGACCGCCAAGCTGAAGGTGACCAAGGGTGGCCCCCTGCCCTTCGCCTGGGACATCCTGTCCC
CTCAGTTCATGTACGGCTCCAAGGCCTACGTGAAGCACCCCGCCGACATCCCCGACTACTTGAAGCTGTCCTTCCCCGAGGG
CTTCAAGTGGGAGCGCGTGATGAACTTCGAGGACGGCGGCGTGGTGACCGTGACCCAGGACTCCTCCCTGCAGGACGGCGA
GTTCATCTACAAGGTGAAGCTGCGCGGCACCAACTTCCCCTCCGACGGCCCCGTAATGCAGAAGAAGACCATGGGCTGGGA
GGCCTCCTCCGAGCGGATGTACCCCGAGGACGGCGCCCTGAAGGGCGAGATCAAGCAGAGGCTGAAGCTGAAGGACGGCG
GCCACTACGACGCTGAGGTCAAGACCACCTACAAGGCCAAGAAGCCCGTGCAGCTGCCCGGCGCCTACAACGTCAACATCA
AGTTGGACATCACCTCCCACAACGAGGACTACACCATCGTGGAACAGTACGAACGCGCCGAGGGCCGCCACTCCACCGGCG
GCATGGACGAGCTGTACGCGTCTGGAGGCGCCACCAATTTCAGCCTGCTGAAACAGGCTGGCGACGTGGAAGAGAACCCTG
GACCTGGACAAAAGTCGGTGAGCAAGGGCGAGGAGCTGTTCACCGGGGTGGTGCCCATCCTGGTCGAGCTGGACGGCGACG
TAAACGGCCACAAGTTCAGCGTGTCCGGCGAGGGCGAGGGCGATGCCACCTACGGCAAGCTGACCCTGAAGTTCATCTGCA
CCACCGGCAAGCTGCCCGTGCCCTGGCCCACCCTCGTGACCACCCTGACCTACGGCGTGCAGTGCTTCAGCCGCTACCCCGA
CCACATGAAGCAGCACGACTTCTTCAAGTCCGCCATGCCCGAAGGCTACGTCCAGGAGCGCACCATCTTCTTCAAGGACGAC
GGCAACTACAAGACCCGCGCCGAGGTGAAGTTCGAGGGCGACACCCTGGTGAACCGCATCGAGCTGAAGGGCATCGACTTC
AAGGAGGACGGCAACATCCTGGGGCACAAGCTGGAGTACAACTACAACAGCCACTAGGGTCTATATCATGGCCGACAAGCA
GAAGAACGGCATCAAGGTGAACTTCAAGATCCGCCACAACATCGAGGACGGCAGCGTGCAGCTCGCCGACCACTACCAGCA
GAACACCCCCATCGGCGACGGCCCCGTGCTGCTGCCCGACAACCACTACCTGAGCACCCAGTCCGCCCTGAGCAAAGACCC
CAACGAGAAGCGCGATCACATGGTCCTGCTGGAGTTCGTGACCGCCGCCGGGATCACTCTCGGCATGGACGAGCTGTACAA
GTAAGAATTCACTCCTCAGGTGCAGGCTGCCTATCAGAAGGTGGTGGCTGGTGTGGCCAATGCCCTGGCTCACAAATACCACTG
AGATCTTTTTCCCTCTGCCAAAAAATTATGGGGACATCATGAAGCCCCTTGAGCATCTGACTTCTGGCTAATAAAGGAAATTTATTT
TCATTGCAATAGTGTGTTGGAATTTTTTGTGTCTCTCACTCGGAAGGACATATGGGAGGGGTCGACAATCAACCTCTGGATTACA
AAATTTGTGAAAGATTGACTGGTATTCTTAACTATGTTGCTCCTTTTACGCTATGTGGATACGCTGCTTTAATGCCTTTGTATCATG
CGTTAACTAAACTTGTTTATTGCAGCTTATAATGGTTACAAATAAAGCAATAGCATCACAAATTTCACAAATAAAGCATTTTTTTCAC
TGCATTCTAGTTGTGGTTTGTCCAAACTCATCAATGTATCTTATCATGTCTGGAATTGACTCAAATGATGTCAATTAGTCTATCAGA
AGCTATCTGGTCTCCCTTCCGGGGGACAAGACATCCCTGTTTAATATTTAAACAGCAGTGTTCCCAAACTGGGTTCTTATATCCCT
TGCTCTGGTCAACCAGGTTGCAGGGTTTCCTGTCCTCACAGGAACGAAGTCCCTAAAGAAACAGTGGCAGCCAGGTTTAGCCCC
GGAATTGACTGGATTCCTTTTTTAGGGCCCATTGGTATGGCTTTTTCCCCGTATCCCCCCAGGTGTCTGCAGGCTCAAAGAGCAG
CGAGAAGCGTTCAGAGGAAAGCGATCCCGTGCCACCTTCCCCGTGCCCGGGCTGTCCCCGCACGCTGCCGGCTCGGGGATGC
GGGGGGAGCGCCGGACCGGAGCGGAGCCCCGGGCGGCTCGCTGCTGCCCCCTAGCGGGGGAGGGACGTAATTACATCCCTG
GGGGCTTTGGGGGGGGGCTGTCCCTGATATCTATAACAAGAAAATATATATATAATAAGTTATCACGTAAGTAGAACATGAAATAA

CAATATAATTATCGTATGAGTTAAATCTTAAAAGTCACGTAAAAGATAATCATGCGTCATTTTGACTCACGCGGTCGTTATAGTTCA
AAATCAGTGACACTTACCGCATTGACAAGCACGCCTCACGGGAGCTCCAAGCGGCGACTGAGATGTCCTAAATGCACAGCGACG
GATTCGCGCTATTTAGAAAGAGAGAGCAATATTTCAAGAATGCATGCGTCAATTTTACGCAGACTATCTTTCTAGGGTTAATCTAG
CTGCATCAGGATCATATCGTCGGGTCTTTTTTCCGGCTCAGTCATCGCCCAAGCTGGCGCTATCTGGGCATCGGGGAGGAAGAA
GCCCGTGCCTTTTCCCGCGAGGTTGAAGCGGCATGGAAAGAGTTTGCCGAGGATGACTGCTGCTGCATTGACGTTGAGCGAAA
ACGCACGTTTACCATGATGATTCGGGAAGGTGTGGCCATGCACGCCTTTAACGGTGAACTGTTCGTTCAGGCCACCTGGGATAC
CAGTTCGTCGCGGCTTTTCCGGACACAGTTCCGGATGGTCAGCCCGAAGCGCATCAGCAACCCGAACAATACCGGCGACAGCC
GGAACTGCCGTGCCGGTGTGCAGATTAATGACAGCGGTGCGGCGCTGGGATATTACGTCAGCGAGGACGGGTATCCTGGCTG
GATGCCGCAGAAATGGACATGGATACCCCGTGAGTTACCCGGCGGGCGCGCTTGGCGTAATCATGGTCATAGCTGTTTCCTGT
GTGAAATTGTTATCCGCTCACAATTCCACACAACATACGAGCCGGAAGCATAAAGTGTAAAGCCTGGGGTGCCTAATGAGTGAG
CTAACTCACATTAATTGCGTTGCGCTCACTGCCCGCTTTCCAGTCGGGAAACCTGTCGTGCCAGCTGCATTAATGAATCGGCCAA
CGCGCGGGGAGAGGCGGTTTGCGTATTGGGCGCTCTTCCGCTTCCTCGCTCACTGACTCGCTGCGCTCGGTCGTTCGGCTGCG
GCGAGCGGTATCAGCTCACTCAAAGGCGGTAATACGGTTATCCACAGAATCAGGGGATAACGCAGGAAAGAACATGTGAGCAAA
AGGCCAGCAAAAGGCCAGGAACCGTAAAAAGGCCGCGTTGCTGGCGTTTTTCCATAGGCTCCGCCCCCCTGACGAGCATCACA
AAAATCGACGCTCAAGTCAGAGGTGGCGAAACCCGACAGGACTATAAAGATACCAGGCGTTTCCCCCTGGAAGCTCCCTCGTGC
GCTCTCCTGTTCCGACCCTGCCGCTTACCGGATACCTGTCCGCCTTTCTCCCTTCGGGAAGCGTGGCGCTTTCTCATAGCTCAC
GCTGTAGGTATCTCAGTTCGGTGTAGGTCGTTCGCTCCAAGCTGGGCTGTGTGCACGAACCCCCCGTTCAGCCCGACCGCTGC
GCCTTATCCGGTAACTATCGTCTTGAGTCCAACCCGGTAAGACACGACTTATCGCCACTGGCAGCAGCCACTGGTAACAGGATT
AGCAGAGCGAGGTATGTAGGCGGTGCTACAGAGTTCTTGAAGTGGTGGCCTAACTACGGCTACACTAGAAGGACAGTATTTGGT
ATCTGCGCTCTGCTGAAGCCAGTTACCTTCGGAAAAAGAGTTGGTAGCTCTTGATCCGGCAAACAAACCACCGCTGGTAGCGGT
GGTTTTTTTGTTTGCAAGCAGCAGATTACGCGCAGAAAAAAAGGATCTCAAGAAGATCCTTTGATCTTTTCTACGGGGTCTGACG
CTCAGTGGAACGAAAACTCACGTTAAGGGATTTTGGTCATGAGATTATCAAAAAGGATCTTCACCTAGATCCTTTTAAATTAAAAAT
GAAGTTTTAAATCAATCTAAAGTATATATGAGTAAACTTGGTCTGACAGTTACCAATGCTTAATCAGTGAGGCACCTATCTCAGCG
ATCTGTCTATTTCGTTCATCCATAGTTGCCTGACTCCCCGTCGTGTAGATAACTACGATACGGGAGGGCTTACCATCTGGCCCCA
GTGCTGCAATGATACCGCGAGACCCACGCTCACCGGCTCCAGATTTATCAGCAATAAACCAGCCAGCCGGAAGGGCCGAGCGC
AGAAGTGGTCCTGCAACTTTATCCGCCTCCATCCAGTCTATTAATTGTTGCCGGGAAGCTAGAGTAAGTAGTTCGCCAGTTAATA
GTTTGCGCAACGTTGTTGCCATTGCTACAGGCATCGTGGTGTCACGCTCGTCGTTTGGTATGGCTTCATTCAGCTCCGGTTCCCA
ACGATCAAGGCGAGTTACATGATCCCCCATGTTGTGCAAAAAAGCGGTTAGCTCCTTCGGTCCTCCGATCGTTGTCAGAAGTAAG
TTGGCCGCAGTGTTATCACTCATGGTTATGGCAGCACTGCATAATTCTCTTACTGTCATGCCATCCGTAAGATGCTTTTCTGTGAC
TGGTGAGTACTCAACCAAGTCATTCTGAGAATAGTGTATGCGGCGACCGAGTTGCTCTTGCCCGGCGTCAATACGGGATAATAC
CGCGCCACATAGCAGAACTTTAAAAGTGCTCATCATTGGAAAACGTTCTTCGGGGCGAAAACTCTCAAGGATCTTACCGCTGTTG
AGATCCAGTTCGATGTAACCCACTCGTGCACCCAACTGATCTTCAGCATCTTTTACTTTCACCAGCGTTTCTGGGTGAGCAAAAA
CAGGAAGGCAAAATGCCGCAAAAAAGGGAATAAGGGCGACACGGAAATGTTGAATACTCAT

2.2.16 Vector 69: Pyl tRNA$_{GCUA}$ PylRS mCherry-P2A-eGFP(150TAGC)

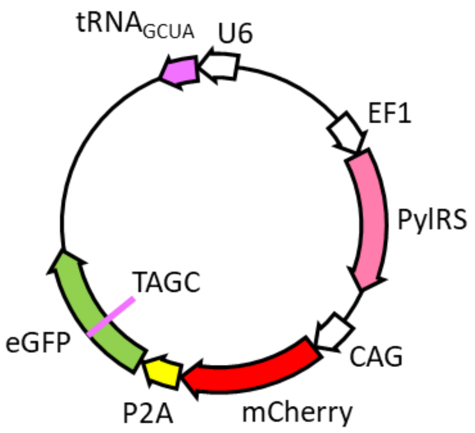

ACTCTTCCTTTTTCAATATTATTGAAGCATTTATCAGGGTTATTGTCTCATGAGCGGATACATATTTGAATGTATTTAGAAAAATAAA
CAAATAGGGGTTCCGCGCACATTTCCCCGAAAAGTGCCACCTAAATTGTAAGCGTTAATATTTTGTTAAAATTCGCGTTAAATTTTT
GTTAAATCAGCTCATTTTTTAACCAATAGGCCGAAATCGGCAAAATCCCTTATAAATCAAAAGAATAGACCGAGATAGGGTTGAGT
GTTGTTCCAGTTTGGAACAAGAGTCCACTATTAAAGAACGTGGACTCCAACGTCAAAGGGCGAAAAACCGTCTATCAGGGCGAT
GGCCCACTACGTGAACCATCACCCTAATCAAGTTTTTTGGGGTCGAGGTGCCGTAAAGCACTAAATCGGAACCCTAAAGGGAGC
CCCCGATTTAGAGCTTGACGGGGAAAGCCGGCGAACGTGGCGAGAAAGGAAGGGAAGAAAGCGAAAGGAGCGGGCGCTAGGG
CGCTGGCAAGTGTAGCGGTCACGCTGCGCGTAACCACCACACCCGCCGCGCTTAATGCGCCGCTACAGGGCGCGTCCCATTC
GCCATTCAGGCTGCGCAACTGTTGGGAAGGGCGATCGGTGCGGGCCTCTTCGCTATTACGCCAGCTGGCGAAAGGGGGATGT
GCTGCAAGGCGATTAAGTTGGGTAACGCCAGGGTTTTCCCAGTCACGACGTTGTAAAACGACGGCCAGTGAGCGCGCCTCGTT
CATTCACGTTTTTGAACCCGTGGAGGACGGGCAGACTCGCGGTGCAAATGTGTTTTACAGCGTGATGGAGCAGATGAAGATGCT
CGACACGCTGCAGAACACGCAGCTAGATTAACCCTAGAAAGATAATCATATTGTGACGTACGTTAAAGATAATCATGCGTAAAATT
GACGCATGTGTTTTATCGGTCTGTATATCGAGGTTTATTTATTAATTTGAATAGATATTAAGTTTTATTATATTTACACTTACATACTA
ATAATAAATTCAACAAACAATTTATTTATGTTTATTTATTTATTAAAAAAAAACAAAAACTCAAAATTTCTTCTATAAAGTAACAAAACTT
TTATGAGGGACAGCCCCCCCCCAAAGCCCCCAGGGATGTAATTACGTCCCTCCCCCGCTAGGGGGCAGCAGCGAGCCGCCCG
GGGCTCCGCTCCGGTCCGGCGCTCCCCCCGCATCCCCGAGCCGGCAGCGTGCGGGGACAGCCCGGGCACGGGGAAGGTGG
CACGGGATCGCTTTCCTCTGAACGCTTCTCGCTGCTCTTTGAGCCTGCAGACACCTGGGGGGATACGGGGAAAAGGCCTAAAAA
CCGCACTTGTC**CGGAAACCCCGGGAATCTAACCCGGCTGAACGGATTTAGCAGTCCATTCGATCTACATGATCAGGTTTCC**GG
TGTTTCGTCCTTTCCACAAGATATATAAAGCCAAGAAATCGAAATACTTTCAAGTTACGGTAAGCATATGATAGTCCATTTTAAAAC
ATAATTTTAAAACTGCAAACTACCCAAGAAATTATTACTTTCTACGTCACGTATTTTGTACTAATATCTTTGTGTTTACAGTCAAATT
AATTCTAATTATCTCTCTAACAGCCTTGTATCGTATATGCAAATATGAAGGAATCATGGGAAATAGGCCCTCTTCCTGCCCAACCG
GTTGCGATCGCTCCGGTGCCCGTCAGTGGGCAGAGCGCACATCGCCCACAGTCCCCGAGAAGTTGGGGGGAGGGGTCGGCAA

TTGAACGGGTGCCTAGAGAAGGTGGCGCGGGGTAAACTGGGAAAGTGATGTCGTGTACTGGCTCCGCCTTTTTCCCGAGGGTG
GGGGAGAACCGTATATAAGTGCAGTAGTCGCCGTGAACGTTCTTTTTCGCAACGGGTTTGCCGCCAGAACACAGCTGAAGCTTC
GAGGGGCTCGCATCTCTCCTTCACGCGCCCGCCGCCCTACCTGAGGCCGCCATCCACGCCGGTTGAGTCGCGTTCTGCCGCCT
CCCGCCTGTGGTGCCTCCTGAACTGCGTCCGCCGTCTAGGTAAGTTTAAAGCTCAGGTCGAGACCGGGCCTTTGTCCGGCGCT
CCCTTGGAGCCTACCTAGACTCAGCCGGCTCTCCACGCTTTGCCTGACCCTGCTTGCTCAACTCTACGTCTTTGTTTCGTTTTCT
GTTCTGCGCCGTTACAGATCCAAGCTGTGACCGGCGCCTACTCTACAGATAGCGTTTAAACTTACGCTTGCCACCATGGCTAGC
GACTACAAGGACGACGACGACAAGGACAAGAAGCCCCTGAACACCCTGATCAGCGCCACAGGACTGTGGATGTCCAGAACC
GGCACCATCCACAAGATCAAGCACCACGAGGTGTCCCGGTCCAAAATCTACATCGAGATGGCCTGCGGCGATCACCTGGTCG
TCAACAACAGCAGAAGCAGCCGGACAGCCAGAGCCCTGCGGCACCACAAGTACAGAAAGACCTGCAAGCGGTGCAGAGTGT
CCGACGAGGACCTGAACAAGTTCCTGACCAAGGCCAACGAGGACCAGACCAGCGTGAAAGTGAAGGTGGTGTCCGCCCCCA
CCCGGACCAAGAAAGCCATGCCCAAGAGCGTGGCCAGAGCCCCCAAGCCCCTGGAAAACACCGAAGCCGCTCAGGCCCAG
CCCAGCGGCAGCAAGTTCAGCCCCGCCATCCCCGTGTCTACCCAGGAAAGCGTCAGCGTCCCCGCCAGCGTGTCCACCAGC
ATCTCTAGCATCTCAACCGGCGCCACAGCTTCTGCCCTGGTCAAGGGCAACACCAACCCCATCACCAGCATGTCTGCCCCTGT
GCAAGCCTCTGCCCCAGCCCTGACCAAGTCCCAGACCGACCGGCTGGAAGTGCTCCTGAACCCCAAGGACGAGATCAGCCT
GAACAGCGGCAAGCCCTTCCGGGAGCTGGAAAGCGAGCTGCTGAGCCGGCGGAAGAAGGACCTCCAGCAAATCTACGCCG
AGGAACGGGAGAACTACCTGGGCAAGCTGGAAAGAGAGATCACCCGGTTCTTCGTGGACCGGGGCTTCCTGGAAATCAAGA
GCCCCATCCTGATCCCCCTGGAGTACATCGAGCGGATGGGCATCGACAACGACACCGAGCTGAGCAAGCAGATTTTCCGGGT
GGACAAGAACTTCTGCCTGCGGCCCATGCTGGCCCCCAACCTGTACAACTACCTGCGGAAACTGGATCGCGCTCTGCCCGAC
CCCATCAAGATTTTCGAGATCGGCCCCTGCTACCGGAAAGAGAGCGACGGCAAAGAGCACCTGGAAGAGTTTACAATGCTGA
ACTTTTGCCAGATGGGCAGCGGCTGCACCAGAGAGAACCTGGAATCCATCATCACCGACTTTCTGAACCACCTGGGGATCGA
CTTCAAGATCGTGGGCGACAGCTGCATGGTGTACGGCGACACCCTGGACGTGATGCACGGCGACCTGGAACTGTCTAGCGC
CGTCGTGGGACCCATCCCTCTGGACCGGGAGTGGGGCATCGATAAGCCCTGGATCGGAGCCGGCTTCGGCCTGGAACGGCT
GCTGAAAGTCAAGCACGACTTTAAGAACATCAAGCGGGCTGCCAGAAGCGAGAGCTACTACAACGGCATCAGCACCAACCT
GTGATGATAAGGATCCACTAGTCCAGTGTGGTGGAATTGACATTGATTATTGACTAGTTATTAATAGTAATCAATTACGGGGTCAT
TAGTTCATAGCCCATATATGGAGTTCCGCGTTACATAACTTACGGTAAATGGCCCGCCTGGCTGACCGCCCAACGACCCCCGCC
CATTGACGTCAATAATGACGTATGTTCCCATAGTAACGCCAATAGGGACTTTCCATTGACGTCAATGGGTGGAGTATTTACGGTA
AACTGCCCACTTGGCAGTACATCAAGTGTATCATATGCCAAGTACGCCCCCTATTGACGTCAATGACGGTAAATGGCCCGCCTG
GCATTATGCCCAGTACATGACCTTATGGGACTTTCCTACTTGGCAGTACATCTACGTATTAGTCATCGCTATTACCATGGTCGAGG
TGAGCCCCACGTTCTGCTTCACTCTCCCCATCTCCCCCCCCTCCCCACCCCCAATTTTGTATTTATTTATTTTTTAATTATTTTGTG
CAGCGATGGGGGCGGGGGGGGGGGGGGGGCGCGCGCCAGGCGGGGCGGGGCGGGGCGAGGGGCGGGGCGGGGCGAGGCG
GAGAGGTGCGGCGGCAGCCAATCAGAGCGGCGCGCTCCGAAAGTTTCCTTTTATGGCGAGGCGGCGGCGGCGGCGGCCCTAT
AAAAAGCGAAGCGCGCGGCGGGCGGGAGTCGCTGCGTTGCCTTCGCCCCGTGCCCCGCTCCGCGCCGCCTCGCGCCGCCCG
CCCCGGCTCTGACTGACCGCGTTACTCCCACAGGTGAGCGGGCGGGACGGCCCTTCTCCTCCGGGCTGTAATTAGCGCTTGGT
TTAATGACGGCTCGTTTCTTTTCTGTGGCTGCGTGAAAGCCTTAAAGGGCTCCGGGAGGGCCCTTTGTGCGGGGGGGAGCGGC
TCGGGGGGTGCGTGCGTGTGTGTGTGCGTGGGGAGCGCCGCGTGCGGCCCGCGCTGCCCGGCGGCTGTGAGCGCTGCGGG
CGCGGCGCGGGGCTTTGTGCGCTCCGCGTGTGCGCGAGGGGAGCGCGGCCGGGGGCGGTGCCCCGCGGTGCGGGGGGGCT
GCGAGGGGAACAAAGGCTGCGTGCGGGGTGTGTGCGTGGGGGGGTGAGCAGGGGGTGTGGGCGCGGCGGTCGGGCTGTAA
CCCCCCCCTGCACCCCCCTCCCCGAGTTGCTGAGCACGGCCCGGCTTCGGGTGCGGGGCTCCGTACGGGGCGTGGCGCGGG
GCTCGCCGTGCCGGGCGGGGGGTGGCGGCAGGTGGGGGTGCCGGGCGGGGCGGGGCCGCCTCGGGCCGGGGAGGGCTCG
GGGGAGGGGCGCGGCGGCCCCCGGAGCGCCGGCGGCTGTCGAGGCGCGGCGAGCCGCAGCCATTGCCTTTTATGGTAATCG
TGCGAGAGGGCGCAGGGACTTCCTTTGTCCCAAATCTGTGCGGAGCCGAAATCTGGGAGGCGCCGCCGCACCCCCTCTAGCG
GGCGCGGGGCGAAGCGGTGCGGCGCCGGCAGGAAGGAAATGGGCGGGGAGGGCCTTCGTGCGTCGCCGCGCCGCCGTCCC
CTTCTCCCTCTCCAGCCTCGGGGCTGTCCGCGGGGGGACGGCTGCCTTCGGGGGGGACGGGGCAGGGCGGGGTTCGGCTTC
TGGCGTGTGACCGGCGGCTCTAGAGCCTCTGCTAACCATGTTCATGCCTTCTTCTTTTTCCTACAGCTCCTGGGCAACGTGCTG
GTTATTGTGCTGTCTCATCATTTTGGCAAAGAATTCGCCACCATGGTGAGCAAGGGCGAGGAGGATAACATGGCCATCATCAA
GGAGTTCATGCGCTTCAAGGTGCACATGGAGGGCTCCGTGAACGGCCACGAGTTCGAGATCGAGGGCGAGGGCGAGGGCC
GCCCCTACGAGGGCACCCAGACCGCCAAGCTGAAGGTGACCAAGGGTGGCCCCCTGCCCTTCGCCTGGGACATCCTGTCCC
CTCAGTTCATGTACGGCTCCAAGGCCTACGTGAAGCACCCCGCCGACATCCCCGACTACTTGAAGCTGTCCTTCCCCGAGGG
CTTCAAGTGGGAGCGCGTGATGAACTTCGAGGACGGCGGCGTGGTGACCGTGACCCAGGACTCCTCCCTGCAGGACGGCGA
GTTCATCTACAAGGTGAAGCTGCGCGGCACCAACTTCCCCTCCGACGGCCCCGTAATGCAGAAGAAGACCATGGGCTGGGA
GGCCTCCTCCGAGCGGATGTACCCCGAGGACGGCGCCCTGAAGGGCGAGATCAAGCAGAGGCTGAAGCTGAAGGACGGCG
GCCACTACGACGCTGAGGTCAAGACCACCTACAAGGCCAAGAAGCCCGTGCAGCTGCCCGGCGCCTACAACGTCAACATCA
AGTTGGACATCACCTCCCACAACGAGGACTACACCATCGTGGAACAGTACGAACGCGCCGAGGGCCGCCACTCCACCGGCG
GCATGGACGAGCTGTACGCGTCTGGAGGCGCCACCAATTTCAGCCTGCTGAAACAGGCTGGCGACGTGGAAGAGAACCCTG
GACCTGGACAAAAGTCGGTGAGCAAGGGCGAGGAGCTGTTCACCGGGGTGGTGCCCATCCTGGTCGAGCTGGACGGCGACG
TAAACGGCCACAAGTTCAGCGTGTCCGGCGAGGGCGAGGGCGATGCCACCTACGGCAAGCTGACCCTGAAGTTCATCTGCA
CCACCGGCAAGCTGCCCGTGCCCTGGCCCACCCTCGTGACCACCCTGACCTACGGCGTGCAGTGCTTCAGCCGCTACCCCGA
CCACATGAAGCAGCACGACTTCTTCAAGTCCGCCATGCCCGAAGGCTACGTCCAGGAGCGCACCATCTTCTTCAAGGACGAC
GGCAACTACAAGACCCGCGCCGAGGTGAAGTTCGAGGGCGACACCCTGGTGAACCGCATCGAGCTGAAGGGCATCGACTTC
AAGGAGGACGGCAACATCCTGGGGCACAAGCTGGAGTACAACTACAACAGCCACTAGCGTCTATATCATGGCCGACAAGCA
GAAGAACGGCATCAAGGTGAACTTCAAGATCCGCCACAACATCGAGGACGGCAGCGTGCAGCTCGCCGACCACTACCAGCA
GAACACCCCCATCGGCGACGGCCCCGTGCTGCTGCCCGACAACCACTACCTGAGCACCCAGTCCGCCCTGAGCAAAGACCC
CAACGAGAAGCGCGATCACATGGTCCTGCTGGAGTTCGTGACCGCCGCCGGGATCACTCTCGGCATGGACGAGCTGTACAA
GTAAGAATTCACTCCTCAGGTGCAGGCTGCCTATCAGAAGGTGGTGGCTGGTGTGGCCAATGCCCTGGCTCACAAATACCACTG
AGATCTTTTTCCCTCTGCCAAAAATTATGGGGACATCATGAAGCCCCTTGAGCATCTGACTTCTGGCTAATAAAGGAAATTTATTT
TCATTGCAATAGTGTGTTGGAATTTTTTGTGTCTCTCACTCGGAAGGACATATGGGAGGGGTCGACAATCAACCTCTGGATTACA
AAATTTGTGAAAGATTGACTGGTATTCTTAACTATGTTGCTCCTTTTACGCTATGTGGATACGCTGCTTTAATGCCTTTGTATCATG
CGTTAACTAAACTTGTTTATTGCAGCTTATAATGGTTACAAATAAAGCAATAGCATCACAAATTTCACAAATAAAGCATTTTTTTCAC
TGCATTCTAGTTGTGGTTTGTCCAAACTCATCAATGTATCTTATCATGTCTGGAATTGACTCAAATGATGTCAATTAGTCTATCAGA
AGCTATCTGGTCTCCCTTCCGGGGGACAAGACATCCCTGTTTAATATTTAAACAGCAGTGTTCCCAAACTGGGTTCTTATATCCCT
TGCTCTGGTCAACCAGGTTGCAGGGTTTCCTGTCCTCACAGGAACGAAGTCCCTAAAGAAACAGTGGCAGCCAGGTTTAGCCCC
GGAATTGACTGGATTCCTTTTTTAGGGCCCATTGGTATGGCTTTTTCCCCGTATCCCCCCAGGTGTCTGCAGGCTCAAAGAGCAG
CGAGAAGCGTTCAGAGGAAAGCGATCCCGTGCCACCTTCCCCGTGCCCGGGCTGTCCCCGCACGCTGCCGGCTCGGGGATGC
GGGGGGAGCGCCGGACCGGAGCGGAGCCCCGGGCGGCTCGCTGCTGCCCCCTAGCGGGGGAGGGACGTAATTACATCCCTG

GGGGCTTTGGGGGGGGGCTGTCCCTGATATCTATAACAAGAAAATATATATATAATAAGTTATCACGTAAGTAGAACATGAAATAA
CAATATAATTATCGTATGAGTTAAATCTTAAAAGTCACGTAAAAGATAATCATGCGTCATTTTGACTCACGCGGTCGTTATAGTTCA
AAATCAGTGACACTTACCGCATTGACAAGCACGCCTCACGGGAGCTCCAAGCGGCGACTGAGATGTCCTAAATGCACAGCGACG
GATTCGCGCTATTTAGAAAGAGAGAGCAATATTTCAAGAATGCATGCGTCAATTTTACGCAGACTATCTTTCTAGGGTTAATCTAG
CTGCATCAGGATCATATCGTCGGGTCTTTTTTCCGGCTCAGTCATCGCCCAAGCTGGCGCTATCTGGGCATCGGGGAGGAAGAA
GCCCGTGCCTTTTCCCGCGAGGTTGAAGCGGCATGGAAAGAGTTTGCCGAGGATGACTGCTGCTGCATTGACGTTGAGCGAAA
ACGCACGTTTACCATGATGATTCGGGAAGGTGTGGCCATGCACGCCTTTAACGGTGAACTGTTCGTTCAGGCCACCTGGGATAC
CAGTTCGTCGCGGCTTTTCCGGACACAGTTCCGGATGGTCAGCCCGAAGCGCATCAGCAACCCGAACAATACCGGCGACAGCC
GGAACTGCCGTGCCGGTGTGCAGATTAATGACAGCGGTGCGGCGCTGGGATATTACGTCAGCGAGGACGGGTATCCTGGCTG
GATGCCGCAGAAATGGACATGGATACCCCGTGAGTTACCCGGCGGGCGCGCTTGGCGTAATCATGGTCATAGCTGTTTCCTGT
GTGAAATTGTTATCCGCTCACAATTCCACACAACATACGAGCCGGAAGCATAAAGTGTAAAGCCTGGGGTGCCTAATGAGTGAG
CTAACTCACATTAATTGCGTTGCGCTCACTGCCCGCTTTCCAGTCGGGAAACCTGTCGTGCCAGCTGCATTAATGAATCGGCCAA
CGCGCGGGGAGAGGCGGTTTGCGTATTGGGCGCTCTTCCGCTTCCTCGCTCACTGACTCGCTGCGCTCGGTCGTTCGGCTGCG
GCGAGCGGTATCAGCTCACTCAAAGGCGGTAATACGGTTATCCACAGAATCAGGGGATAACGCAGGAAAGAACATGTGAGCAAA
AGGCCAGCAAAAGGCCAGGAACCGTAAAAAGGCCGCGTTGCTGGCGTTTTTCCATAGGCTCCGCCCCCCTGACGAGCATCACA
AAAATCGACGCTCAAGTCAGAGGTGGCGAAACCCGACAGGACTATAAAGATACCAGGCGTTTCCCCCTGGAAGCTCCCTCGTGC
GCTCTCCTGTTCCGACCCTGCCGCTTACCGGATACCTGTCCGCCTTTCTCCCTTCGGGAAGCGTGGCGCTTTCTCATAGCTCAC
GCTGTAGGTATCTCAGTTCGGTGTAGGTCGTTCGCTCCAAGCTGGGCTGTGTGCACGAACCCCCCGTTCAGCCCGACCGCTGC
GCCTTATCCGGTAACTATCGTCTTGAGTCCAACCCGGTAAGACACGACTTATCGCCACTGGCAGCAGCCACTGGTAACAGGATT
AGCAGAGCGAGGTATGTAGGCGGTGCTACAGAGTTCTTGAAGTGGTGGCCTAACTACGGCTACACTAGAAGGACAGTATTTGGT
ATCTGCGCTCTGCTGAAGCCAGTTACCTTCGGAAAAAGAGTTGGTAGCTCTTGATCCGGCAAACAAACCACCGCTGGTAGCGGT
GGTTTTTTTGTTTGCAAGCAGCAGATTACGCGCAGAAAAAAAGGATCTCAAGAAGATCCTTTGATCTTTTCTACGGGGTCTGACG
CTCAGTGGAACGAAAACTCACGTTAAGGGATTTTGGTCATGAGATTATCAAAAAGGATCTTCACCTAGATCCTTTTAAATTAAAAAT
GAAGTTTTAAATCAATCTAAAGTATATATGAGTAAACTTGGTCTGACAGTTACCAATGCTTAATCAGTGAGGCACCTATCTCAGCG
ATCTGTCTATTTCGTTCATCCATAGTTGCCTGACTCCCCGTCGTGTAGATAACTACGATACGGGAGGGCTTACCATCTGGCCCCA
GTGCTGCAATGATACCGCGAGACCCACGCTCACCGGCTCCAGATTTATCAGCAATAAACCAGCCAGCCGGAAGGGCCGAGCGC
AGAAGTGGTCCTGCAACTTTATCCGCCTCCATCCAGTCTATTAATTGTTGCCGGGAAGCTAGAGTAAGTAGTTCGCCAGTTAATA
GTTTGCGCAACGTTGTTGCCATTGCTACAGGCATCGTGGTGTCACGCTCGTCGTTTGGTATGGCTTCATTCAGCTCCGGTTCCCA
ACGATCAAGGCGAGTTACATGATCCCCCATGTTGTGCAAAAAAGCGGTTAGCTCCTTCGGTCCTCCGATCGTTGTCAGAAGTAAG
TTGGCCGCAGTGTTATCACTCATGGTTATGGCAGCACTGCATAATTCTCTTACTGTCATGCCATCCGTAAGATGCTTTTCTGTGAC
TGGTGAGTACTCAACCAAGTCATTCTGAGAATAGTGTATGCGGCGACCGAGTTGCTCTTGCCCGGCGTCAATACGGGATAATAC
CGCGCCACATAGCAGAACTTTAAAAGTGCTCATCATTGGAAAACGTTCTTCGGGGCGAAAACTCTCAAGGATCTTACCGCTGTTG
AGATCCAGTTCGATGTAACCCACTCGTGCACCCAACTGATCTTCAGCATCTTTTACTTTCACCAGCGTTTCTGGGTGAGCAAAAA
CAGGAAGGCAAAATGCCGCAAAAAAGGGAATAAGGGCGACACGGAAATGTTGAATACTCAT

2.2.17 Vector 71: Tyr $tRNA_{CUA}$ TyrRS* mCherry-P2A-eGFP(150TAG)

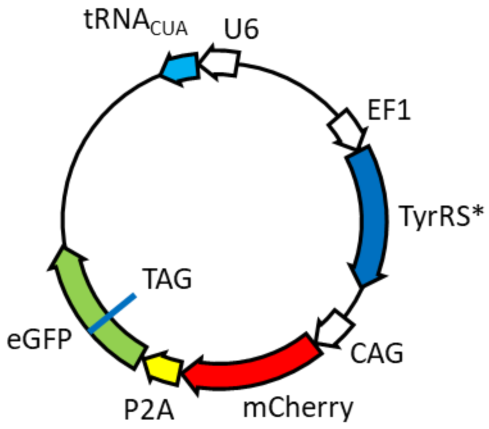

ACTCTTCCTTTTTCAATATTATTGAAGCATTTATCAGGGTTATTGTCTCATGAGCGGATACATATTTGAATGTATTTAGAAAAATAAA
CAAATAGGGGTTCCGCGCACATTTCCCCGAAAAGTGCCACCTAAATTGTAAGCGTTAATATTTTGTTAAAATTCGCGTTAAATTTTT
GTTAAATCAGCTCATTTTTTAACCAATAGGCCGAAATCGGCAAAATCCCTTATAAATCAAAAGAATAGACCGAGATAGGGTTGAGT
GTTGTTCCAGTTTGGAACAAGAGTCCACTATTAAAGAACGTGGACTCCAACGTCAAAGGGCGAAAAACCGTCTATCAGGGCGAT
GGCCCACTACGTGAACCATCACCCTAATCAAGTTTTTTGGGGTCGAGGTGCCGTAAAGCACTAAATCGGAACCCTAAAGGGAGC
CCCCGATTTAGAGCTTGACGGGGAAAGCCGGCGAACGTGGCGAGAAAGGAAGGGAAGAAAGCGAAAGGAGCGGGCGCTAGGG
CGCTGGCAAGTGTAGCGGTCACGCTGCGCGTAACCACCACACCCGCCGCGCTTAATGCGCCGCTACAGGGCGCGTCCCATTC
GCCATTCAGGCTGCGCAACTGTTGGGAAGGGCGATCGGTGCGGGCCTCTTCGCTATTACGCCAGCTGGCGAAAGGGGGATGT
GCTGCAAGGCGATTAAGTTGGGTAACGCCAGGGTTTTCCCAGTCACGACGTTGTAAAACGACGGCCAGTGAGCGCGCCTCGTT
CATTCACGTTTTTGAACCCGTGGAGGACGGGCAGACTCGCGGTGCAAATGTGTTTTACAGCGTGATGGAGCAGATGAAGATGCT
CGACACGCTGCAGAACACGCAGCTAGATTAACCCTAGAAAGATAATCATATTGTGACGTACGTTAAAGATAATCATGCGTAAAATT
GACGCATGTGTTTTATCGGTCTGTATATCGAGGTTTATTTATTAATTTGAATAGATATTAAGTTTTATTATATTTACACTTACATACTA
ATAATAAATTCAACAAACAATTTATTTATGTTTATTTATTTATTAAAAAAAAACAAAAACTCAAAATTTCTTCTATAAAGTAACAAAACTT
TTATGAGGGACAGCCCCCCCCCAAAGCCCCCAGGGATGTAATTACGTCCCTCCCCCGCTAGGGGGCAGCAGCGAGCCGCCCG
GGGCTCCGCTCCGGTCCGGCGCTCCCCCCGCATCCCCGAGCCGGCAGCGTGCGGGGACAGCCCGGGCACGGGGAAGGTGG
CACGGGATCGCTTTCCTCTGAACGCTTCTCGCTGCTCTTTGAGCCTGCAGACACCTGGGGGGATACGGGGAAAAGGCCTAAAAA
CCGCACTTGTC**TGGTGGGGGAAGGATTCGAACCTTCGAAGTCTGTGACGGCAGATTTAGAGTCTGCTCCCTTTGGCCGCTCGG**
GAACCCCACCGGTGTTTCGTCCTTTCCACAAGATATATAAAGCCAAGAAATCGAAATACTTTCAAGTTACGGTAAGCATATGATAG
TCCATTTTAAAACATAATTTTAAAACTGCAAACTACCCAAGAAATTATTACTTTCTACGTCACGTATTTTGTACTAATATCTTTGTGTT
TACAGTCAAATTAATTCTAATTATCTCTCTAACAGCCTTGTATCGTATATGCAAATATGAAGGAATCATGGGAAATAGGCCCTCTTC

CTGCCCAACCGGTTGCGATCGCTCCGGTGCCCGTCAGTGGGCAGAGCGCACATCGCCCACAGTCCCCGAGAAGTTGGGGGGA
GGGGTCGGCAATTGAACGGGTGCCTAGAGAAGGTGGCGCGGGGTAAACTGGGAAAGTGATGTCGTGTACTGGCTCCGCCTTTT
TCCCGAGGGTGGGGGAGAACCGTATATAAGTGCAGTAGTCGCCGTGAACGTTCTTTTTCGCAACGGGTTTGCCGCCAGAACACA
GCTGAAGCTTCGAGGGGCTCGCATCTCTCCTTCACGCGCCCGCCGCCCTACCTGAGGCCGCCATCCACGCCGGTTGAGTCGC
GTTCTGCCGCCTCCCGCCTGTGGTGCCTCCTGAACTGCGTCCGCCGTCTAGGTAAGTTTAAAGCTCAGGTCGAGACCGGGCCT
TTGTCCGGCGCTCCCTTGGAGCCTACCTAGACTCAGCCGGCTCTCCACGCTTTGCCTGACCCTGCTTGCTCAACTCTACGTCTTT
GTTTCGTTTTCTGTTCTGCGCCGTTACAGATCCAAGCTGTGACCGGCGCCTACTCTACAGATAGCGTTTAAACTTACGCTTGCCA
CC**ATGGCTAGCGACTACAAGGACGACGACGACAAGGCAAGCAGTAACTTGATTAAACAATTGCAAGAGCGGGGGCTGGTAG**
CCCAGGTGACGGACGAGGAAGCGTTAGCAGAGCGACTGGCGCAAGGCCCGATCGCACTCGTGTGTGGCTTCGATCCTACCG
CTGACAGCTTGCATTTGGGGCATCTTGTTCCATTGTTATGCCTGAAACGCTTCCAGCAGGCGGGCCACAAGCCGGTTGCGCTG
GTAGGCGGCGCGACGGGTCTGATTGGCGACCCGAGCTTCAAAGCTGCCGAGCGTAAGCTGAACACCGAAGAAACTGTTCAG
GAGTGGGTGGACAAAATCCGTAAGCAGGTTGCCCCGTTCCTCGATTTCGACTGTGGAGAAAACTCTGCTATCGCGGCCAATA
ATTATGACTGGTTCGGCAATATGAATGTGCTGACCTTCCTGCGCGATATTGGCAAACACTTCTCCGTTAACCAGATGATCAACA
AAGAAGCGGTTAAGCAGCGTCTCAACCGTGAAGATCAGGGGATTTCGTTCACTGAGTTTTCCTACAACCTGCTGCAGGGTTAT
AGTATGGCCTGTTTGAACAAACAGTACGGTGTGGTGCTGCAAATTGGTGGTTCTGACCAGTGGGGTAACATCACTTCTGGTAT
CGACCTGACCCGTCGTCTGCATCAGAATCAGGTGTTTGGCCTGACCGTTCCGCTGATCACTAAAGCAGATGGCACCAAATTTG
GTAAAACTGAAGGCGGCGCAGTCTGGTTGGATCCGAAGAAAACCAGCCCGTACAAATTCTACCAGTTCTGGATCAACACTGC
GCGTGCCGACGTTTACCGCTTCCTGAAGTTCTTCACCTTTATGAGCATTGAAGAGATCAACGCCCTGGAAGAAGAAGATAAAA
ACAGCGGTAAAGCACCGCGCGCCCAGTATGTACTGGCGGAGCAGGTGACTCGTCTGGTTCACGGTGAAGAAGGTTTACAGG
CGGCAAAACGTATTACCGAATGCCTGTTCAGCGGTTCTTTGAGTGCGCTGAGTGAAGCGGACTTCGAACAGCTGGCGCAGGA
CGGCGTACCGATGGTTGAGATGGAAAAGGGCGCAGACCTGATGCAGGCACTGGTCGATTCTGAACTGCAACCTTCCCGTGGT
CAGGCACGTAAAACTATCGCCTCCAATGCCATCACCATTAACGGTGAAAAACAGTCCGATCCTGAATACTTCTTTAAAGAAGA
AGATCGTCTGTTTGGTCGTTTTACCTTACTGCGTCGCGGTAAAAAGAATTACTGTCTGATTTGCTGGAAAGGGCCCGTTTAATG
ATAAGGATCCACTAGTCCAGTGTGGTGGAATTGACATTGATTATTGACTAGTTATTAATAGTAATCAATTACGGGGTCATTAGTTC
ATAGCCCATATATGGAGTTCCGCGTTACATAACTTACGGTAAATGGCCCGCCTGGCTGACCGCCCAACGACCCCCGCCCATTGA
CGTCAATAATGACGTATGTTCCCATAGTAACGCCAATAGGGACTTTCCATTGACGTCAATGGGTGGAGTATTTACGGTAAACTGC
CCACTTGGCAGTACATCAAGTGTATCATATGCCAAGTACGCCCCCTATTGACGTCAATGACGGTAAATGGCCCGCCTGGCATTAT
GCCCAGTACATGACCTTATGGGACTTTCCTACTTGGCAGTACATCTACGTATTAGTCATCGCTATTACCATGGTCGAGGTGAGCC
CCACGTTCTGCTTCACTCTCCCCATCTCCCCCCCCTCCCCACCCCCAATTTTGTATTTATTTATTTTTTAATTATTTTGTGCAGCGA
TGGGGGCGGGGGGGGGGGGGGCGCGCGCCAGGCGGGGCGGGGCGGGGCGAGGGGCGGGGCGGGGCGAGGCGGAGAGGT
GCGGCGGCAGCCAATCAGAGCGGCGCGCTCCGAAAGTTTCCTTTTATGGCGAGGCGGCGGCGGCGGCGGCCCTATAAAAAGC
GAAGCGCGCGGCGGGCGGGAGTCGCTGCGTTGCCTTCGCCCCGTGCCCCGCTCCGCGCCGCCTCGCGCCGCCCGCCCCGGC
TCTGACTGACCGCGTTACTCCCACAGGTGAGCGGGCGGGACGGCCCTTCTCCTCCGGGCTGTAATTAGCGCTTGGTTTAATGAC
GGCTCGTTTCTTTTCTGTGGCTGCGTGAAAGCCTTAAAGGGCTCCGGGAGGGCCCTTTGTGCGGGGGGGAGCGGCTCGGGGG
GTGCGTGCGTGTGTGTGTGCGTGGGGAGCGCCGCGTGCGGCCCGCGCTGCCCGGCGGCTGTGAGCGCTGCGGGCGCGGCG
CGGGGCTTTGTGCGCTCCGCGTGTGCGCGAGGGGAGCGCGGCCGGGGGCGGTGCCCCGCGGTGCGGGGGGGCTGCGAGGG
GAACAAAGGCTGCGTGCGGGGTGTGTGCGTGGGGGGGTGAGCAGGGGGTGTGGGCGCGGCGGTCGGGCTGTAACCCCCCC
TGCACCCCCCTCCCCGAGTTGCTGAGCACGGCCCGGCTTCGGGTGCGGGGCTCCGTACGGGGCGTGGCGCGGGGCTCGCCG
TGCCGGGCGGGGGGTGGCGGCAGGTGGGGGTGCCGGGCGGGGCGGGGCCGCCTCGGGCCGGGGAGGGCTCGGGGGAGG
GGCGCGGCGGCCCCCGGAGCGCCGGCGGCTGTCGAGGCGCGGCGAGCCGCAGCCATTGCCTTTTATGGTAATCGTGCGAGA
GGGCGCAGGGACTTCCTTTGTCCCAAATCTGTGCGGAGCCGAAATCTGGGAGGCGCCGCCGCACCCCCTCTAGCGGGCGCGG
GGCGAAGCGGTGCGGCGCCGGCAGGAAGGAAATGGGCGGGGAGGGCCTTCGTGCGTCGCCGCGCCGCCGTCCCCTTCTCCC
TCTCCAGCCTCGGGGCTGTCCGCGGGGGGACGGCTGCCTTCGGGGGGGACGGGGCAGGGCGGGGTTCGGCTTCTGGCGTGT
GACCGGCGGCTCTAGAGCCTCTGCTAACCATGTTCATGCCTTCTTCTTTTTCCTACAGCTCCTGGGCAACGTGCTGGTTATTGTG
CTGTCTCATCATTTTGGCAAAGAATTCGCCACC**ATGGTGAGCAAGGGCGAGGAGGATAACATGGCCATCATCAAGGAGTTCAT**
GCGCTTCAAGGTGCACATGGAGGGCTCCGTGAACGGCCACGAGTTCGAGATCGAGGGCGAGGGCGAGGGCCGCCCCTACG
AGGGCACCCAGACCGCCAAGCTGAAGGTGACCAAGGGTGGCCCCCTGCCCTTCGCCTGGGACATCCTGTCCCCTCAGTTCAT
GTACGGCTCCAAGGCCTACGTGAAGCACCCCGCCGACATCCCCGACTACTTGAAGCTGTCCTTCCCCGAGGGCTTCAAGTGG
GAGCGCGTGATGAACTTCGAGGACGGCGGCGTGGTGACCGTGACCCAGGACTCCTCCCTGCAGGACGGCGAGTTCATCTAC
AAGGTGAAGCTGCGCGGCACCAACTTCCCCTCCGACGGCCCCGTAATGCAGAAGAAGACCATGGGCTGGGAGGCCTCCTCC
GAGCGGATGTACCCCGAGGACGGCGCCCTGAAGGGCGAGATCAAGCAGAGGCTGAAGCTGAAGGACGGCGGCCACTACGA
CGCTGAGGTCAAGACCACCTACAAGGCCAAGAAGCCCGTGCAGCTGCCCGGCGCCTACAACGTCAACATCAAGTTGGACAT
CACCTCCCACAACGAGGACTACACCATCGTGGAACAGTACGAACGCGCCGAGGGCCGCCACTCCACCGGCGGCATGGACGA
GCTGTACGCGTCTGGAGGCGCCACCAATTTCAGCCTGCTGAAACAGGCTGGCGACGTGGAAGAGAACCCTGGACCTGGACA
AAAGTCGGTGAGCAAGGGCGAGGAGCTGTTCACCGGGGTGGTGCCCATCCTGGTCGAGCTGGACGGCGACGTAAACGGCCA
CAAGTTCAGCGTGTCCGGCGAGGGCGAGGGCGATGCCACCTACGGCAAGCTGACCCTGAAGTTCATCTGCACCACCGGCAA
GCTGCCCGTGCCCTGGCCCACCCTCGTGACCACCCTGACCTACGGCGTGCAGTGCTTCAGCCGCTACCCCGACCACATGAAG
CAGCACGACTTCTTCAAGTCCGCCATGCCCGAAGGCTACGTCCAGGAGCGCACCATCTTCTTCAAGGACGACGGCAACTACA
AGACCCGCGCCGAGGTGAAGTTCGAGGGCGACACCCTGGTGAACCGCATCGAGCTGAAGGGCATCGACTTCAAGGAGGACG
GCAACATCCTGGGGCACAAGCTGGAGTACAACTACAACAGCCACTAGGTCTATATCATGGCCGACAAGCAGAAGAACGGCAT
CAAGGTGAACTTCAAGATCCGCCACAACATCGAGGACGGCAGCGTGCAGCTCGCCGACCACTACCAGCAGAACACCCCCAT
CGGCGACGGCCCCGTGCTGCTGCCCGACAACCACTACCTGAGCACCCAGTCCGCCCTGAGCAAAGACCCCAACGAGAAGCG
CGATCACATGGTCCTGCTGGAGTTCGTGACCGCCGCCGGGATCACTCTCGGCATGGACGAGCTGTACAAGTAAGAATTCACT
CCTCAGGTGCAGGCTGCCTATCAGAAGGTGGTGGCTGGTGTGGCCAATGCCCTGGCTCACAAATACCACTGAGATCTTTTTCCC
TCTGCCAAAAATTATGGGGACATCATGAAGCCCCTTGAGCATCTGACTTCTGGCTAATAAAGGAAATTTATTTTCATTGCAATAGT
GTGTTGGAATTTTTTGTGTCTCTCACTCGGAAGGACATATGGGAGGGGTCGACAATCAACCTCTGGATTACAAAATTTGTGAAAG
ATTGACTGGTATTCTTAACTATGTTGCTCCTTTTACGCTATGTGGATACGCTGCTTTAATGCCTTTGTATCATGCGTTAACTAAACT
TGTTTATTGCAGCTTATAATGGTTACAAATAAAGCAATAGCATCACAAATTTCACAAATAAAGCATTTTTTTCACTGCATTCTAGTTG
TGGTTTGTCCAAACTCATCAATGTATCTTATCATGTCTGGAATTGACTCAAATGATGTCAATTAGTCTATCAGAAGCTATCTGGTCT
CCCTTCCGGGGGACAAGACATCCCTGTTTAATATTTAAACAGCAGTGTTCCCAAACTGGGTTCTTATATCCCTTGCTCTGGTCAAC
CAGGTTGCAGGGTTTCCTGTCCTCACAGGAACGAAGTCCCTAAAGAAACAGTGGCAGCCAGGTTTAGCCCCGGAATTGACTGGA
TTCCTTTTTTAGGGCCCATTGGTATGGCTTTTTCCCCGTATCCCCCCAGGTGTCTGCAGGCTCAAAGAGCAGCGAGAAGCGTTCA
GAGGAAAGCGATCCCGTGCCACCTTCCCCGTGCCCGGGCTGTCCCCGCACGCTGCCGGCTCGGGGATGCGGGGGGAGCGCC
GGACCGGAGCGGAGCCCCGGGCGGCTCGCTGCTGCCCCCTAGCGGGGGAGGGACGTAATTACATCCCTGGGGGCTTTGGGG

GGGGGCTGTCCCTGATATCTATAACAAGAAAATATATATATAATAAGTTATCACGTAAGTAGAACATGAAATAACAATATAATTATC
GTATGAGTTAAATCTTAAAAGTCACGTAAAAGATAATCATGCGTCATTTTGACTCACGCGGTCGTTATAGTTCAAAATCAGTGACA
CTTACCGCATTGACAAGCACGCCTCACGGGAGCTCCAAGCGGCGACTGAGATGTCCTAAATGCACAGCGACGGATTCGCGCTA
TTTAGAAAGAGAGAGCAATATTTCAAGAATGCATGCGTCAATTTTACGCAGACTATCTTTCTAGGGTTAATCTAGCTGCATCAGGA
TCATATCGTCGGGTCTTTTTTCCGGCTCAGTCATCGCCCAAGCTGGCGCTATCTGGGCATCGGGGAGGAAGAAGCCCGTGCCTT
TTCCCGCGAGGTTGAAGCGGCATGGAAAGAGTTTGCCGAGGATGACTGCTGCTGCATTGACGTTGAGCGAAAACGCACGTTTAC
CATGATGATTCGGGAAGGTGTGGCCATGCACGCCTTTAACGGTGAACTGTTCGTTCAGGCCACCTGGGATACCAGTTCGTCGCG
GCTTTTCCGGACACAGTTCCGGATGGTCAGCCCGAAGCGCATCAGCAACCCGAACAATACCGGCGACAGCCGGAACTGCCGTG
CCGGTGTGCAGATTAATGACAGCGGTGCGGCGCTGGGATATTACGTCAGCGAGGACGGGTATCCTGGCTGGATGCCGCAGAAA
TGGACATGGATACCCCGTGAGTTACCCGGCGGGCGCGCTTGGCGTAATCATGGTCATAGCTGTTTCCTGTGTGAAATTGTTATC
CGCTCACAATTCCACACAACATACGAGCCGGAAGCATAAAGTGTAAAGCCTGGGGTGCCTAATGAGTGAGCTAACTCACATTAAT
TGCGTTGCGCTCACTGCCCGCTTTCCAGTCGGGAAACCTGTCGTGCCAGCTGCATTAATGAATCGGCCAACGCGCGGGGAGAG
GCGGTTTGCGTATTGGGCGCTCTTCCGCTTCCTCGCTCACTGACTCGCTGCGCTCGGTCGTTCGGCTGCGGCGAGCGGTATCA
GCTCACTCAAAGGCGGTAATACGGTTATCCACAGAATCAGGGGATAACGCAGGAAAGAACATGTGAGCAAAAGGCCAGCAAAAG
GCCAGGAACCGTAAAAAGGCCGCGTTGCTGGCGTTTTTCCATAGGCTCCGCCCCCCTGACGAGCATCACAAAAATCGACGCTCA
AGTCAGAGGTGGCGAAACCCGACAGGACTATAAAGATACCAGGCGTTTCCCCCTGGAAGCTCCCTCGTGCGCTCTCCTGTTCCG
ACCCTGCCGCTTACCGGATACCTGTCCGCCTTTCTCCCTTCGGGAAGCGTGGCGCTTTCTCATAGCTCACGCTGTAGGTATCTC
AGTTCGGTGTAGGTCGTTCGCTCCAAGCTGGGCTGTGTGCACGAACCCCCCGTTCAGCCCGACCGCTGCGCCTTATCCGGTAA
CTATCGTCTTGAGTCCAACCCGGTAAGACACGACTTATCGCCACTGGCAGCAGCCACTGGTAACAGGATTAGCAGAGCGAGGTA
TGTAGGCGGTGCTACAGAGTTCTTGAAGTGGTGGCCTAACTACGGCTACACTAGAAGGACAGTATTTGGTATCTGCGCTCTGCT
GAAGCCAGTTACCTTCGGAAAAAGAGTTGGTAGCTCTTGATCCGGCAAACAAACCACCGCTGGTAGCGGTGGTTTTTTTGTTTGC
AAGCAGCAGATTACGCGCAGAAAAAAAGGATCTCAAGAAGATCCTTTGATCTTTTCTACGGGGTCTGACGCTCAGTGGAACGAAA
ACTCACGTTAAGGGATTTTGGTCATGAGATTATCAAAAAGGATCTTCACCTAGATCCTTTTAAATTAAAAATGAAGTTTTAAATCAA
TCTAAAGTATATATGAGTAAACTTGGTCTGACAGTTACCAATGCTTAATCAGTGAGGCACCTATCTCAGCGATCTGTCTATTTCGT
TCATCCATAGTTGCCTGACTCCCCGTCGTGTAGATAACTACGATACGGGAGGGCTTACCATCTGGCCCCAGTGCTGCAATGATA
CCGCGAGACCCACGCTCACCGGCTCCAGATTTATCAGCAATAAACCAGCCAGCCGGAAGGGCCGAGCGCAGAAGTGGTCCTGC
AACTTTATCCGCCTCCATCCAGTCTATTAATTGTTGCCGGGAAGCTAGAGTAAGTAGTTCGCCAGTTAATAGTTTGCGCAACGTTG
TTGCCATTGCTACAGGCATCGTGGTGTCACGCTCGTCGTTTGGTATGGCTTCATTCAGCTCCGGTTCCCAACGATCAAGGCGAG
TTACATGATCCCCCATGTTGTGCAAAAAAGCGGTTAGCTCCTTCGGTCCTCCGATCGTTGTCAGAAGTAAGTTGGCCGCAGTGTT
ATCACTCATGGTTATGGCAGCACTGCATAATTCTCTTACTGTCATGCCATCCGTAAGATGCTTTTCTGTGACTGGTGAGTACTCAA
CCAAGTCATTCTGAGAATAGTGTATGCGGCGACCGAGTTGCTCTTGCCCGGCGTCAATACGGGATAATACCGCGCCACATAGCA
GAACTTTAAAAGTGCTCATCATTGGAAAACGTTCTTCGGGGCGAAAACTCTCAAGGATCTTACCGCTGTTGAGATCCAGTTCGAT
GTAACCCACTCGTGCACCCAACTGATCTTCAGCATCTTTTACTTTCACCAGCGTTTCTGGGTGAGCAAAAACAGGAAGGCAAAAT
GCCGCAAAAAAGGGAATAAGGGCGACACGGAAATGTTGAATACTCAT

2.2.18 Vector 73: 4x(Pyl tRNA$_{UCCU(Ev2)}$) PylRS mCherry-P2A-eGFP(150AGGA)

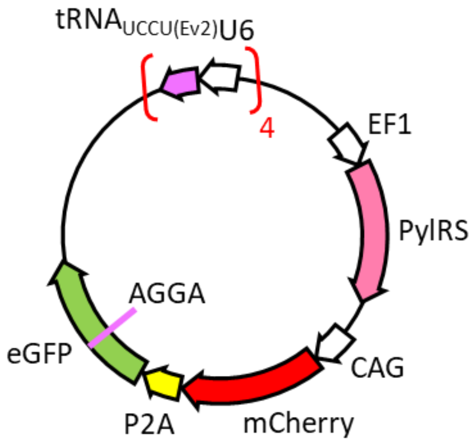

ACTCTTCCTTTTTCAATATTATTGAAGCATTTATCAGGGTTATTGTCTCATGAGCGGATACATATTTGAATGTATTTAGAAAAATAAA
CAAATAGGGGTTCCGCGCACATTTCCCCGAAAAGTGCCACCTAAATTGTAAGCGTTAATATTTTGTTAAAATTCGCGTTAAATTTTT
GTTAAATCAGCTCATTTTTTAACCAATAGGCCGAAATCGGCAAAATCCCTTATAAATCAAAAGAATAGACCGAGATAGGGTTGAGT
GTTGTTCCAGTTTGGAACAAGAGTCCACTATTAAAGAACGTGGACTCCAACGTCAAAGGGCGAAAAACCGTCTATCAGGGCGAT
GGCCCACTACGTGAACCATCACCCTAATCAAGTTTTTTGGGGTCGAGGTGCCGTAAAGCACTAAATCGGAACCCTAAAGGGAGC
CCCCGATTTAGAGCTTGACGGGGAAAGCCGGCGAACGTGGCGAGAAAGGAAGGGAAGAAAGCGAAAGGAGCGGGCGCTAGGG
CGCTGGCAAGTGTAGCGGTCACGCTGCGCGTAACCACCACACCCGCCGCGCTTAATGCGCCGCTACAGGGCGCGTCCCATTC
GCCATTCAGGCTGCGCAACTGTTGGGAAGGGCGATCGGTGCGGGCCTCTTCGCTATTACGCCAGCTGGCGAAAGGGGGATGT
GCTGCAAGGCGATTAAGTTGGGTAACGCCAGGGTTTTCCCAGTCACGACGTTGTAAAACGACGGCCAGTGAGCGCGCCTCGTT
CATTCACGTTTTTGAACCCGTGGAGGACGGGCAGACTCGCGGTGCAAATGTGTTTTACAGCGTGATGGAGCAGATGAAGATGCT
CGACACGCTGCAGAACACGCAGCTAGATTAACCCTAGAAAGATAATCATATTGTGACGTACGTTAAAGATAATCATGCGTAAAATT
GACGCATGTGTTTTATCGGTCTGTATATCGAGGTTTATTTATTAATTTGAATAGATATTAAGTTTTATTATATTTACACTTACATACTA
ATAATAAATTCAACAAACAATTTATTTATGTTTATTTATTTATTAAAAAAAAACAAAAACTCAAAATTTCTTCTATAAAGTAACAAAACTT
TTATGAGGGACAGCCCCCCCCCAAAGCCCCCAGGGATGTAATTACGTCCCTCCCCCGCTAGGGGGCAGCAGCGAGCCGCCCG
GGGCTCCGCTCCGGTCCGGCGCTCCCCCCGCATCCCCGAGCCGGCAGCGTGCGGGGACAGCCCGGGCACGGGGAAGGTGG
CACGGGATCGCTTTCCTCTGAACGCTTCTCGCTGCTCTTTGAGCCTGCAGACACCTGGGGGGATACGGGGAAAAGGCCTCTTAA
GAAAAACCGCACTTGTC**CGGAAACCCCGGGAATCTAACCCGGCTGAAGCGGATAGGAAGCCCCTTCGATCTACATGATCAGG
TTTCC**GGTGTTTCGTCCTTTCCACAAGATATATAAAGCCAAGAAATCGAAATACTTTCAAGTTACGGTAAGCATATGATAGTCCATT
TTAAAACATAATTTTAAAACTGCAAACTACCCAAGAAATTATTACTTTCTACGTCACGTATTTTGTACTAATATCTTTGTGTTTACAG

TCAAATTAATTCTAATTATCTCTCTAACAGCCTTGTATCGTATATGCAAATATGAAGGAATCATGGGAAATAGGCCCTCTTCCTGCC
CAATCGACGAACTTGTGGCCGTTTACCCTCTTAAGAAAAACCGCACTTGTCCGGAAACCCCGGGAATCTAACCCGGCTGAAGC
GGATAGGAAGCCCCTTCGATCTACATGATCAGGTTTCCGGTGTTTCGTCCTTTCCACAAGATATATAAAGCCAAGAAATCGAAAT
ACTTTCAAGTTACGGTAAGCATATGATAGTCCATTTTAAAACATAATTTTAAAACTGCAAACTACCCAAGAAATTATTACTTTCTACG
TCACGTATTTTGTACTAATATCTTTGTGTTTACAGTCAAATTAATTCTAATTATCTCTCTAACAGCCTTGTATCGTATATGCAAATAT
GAAGGAATCATGGGAAATAGGCCCTCTTCCTGCCCAATTCGGCAAGTTCTCCACCAGCCTCTTAAGAAAAACCGCACTTGTCCG
GAAACCCCGGGAATCTAACCCGGCTGAAGCGGATAGGAAGCCCCTTCGATCTACATGATCAGGTTTCCGGTGTTTCGTCCTTT
CCACAAGATATATAAAGCCAAGAAATCGAAATACTTTCAAGTTACGGTAAGCATATGATAGTCCATTTTAAAACATAATTTTAAAAC
TGCAAACTACCCAAGAAATTATTACTTTCTACGTCACGTATTTTGTACTAATATCTTTGTGTTTACAGTCAAATTAATTCTAATTATC
TCTCTAACAGCCTTGTATCGTATATGCAAATATGAAGGAATCATGGGAAATAGGCCCTCTTCCTGCCCAACCTCTTAAGAAAAACC
GCACTTGTCCCTAAAAACCGCACTTGTCCGGAAACCCCGGGAATCTAACCCGGCTGAAGCGGATAGGAAGCCCCTTCGATCTA
CATGATCAGGTTTCCGGTGTTTCGTCCTTTCCACAAGATATATAAAGCCAAGAAATCGAAATACTTTCAAGTTACGGTAAGCATAT
GATAGTCCATTTTAAAACATAATTTTAAAACTGCAAACTACCCAAGAAATTATTACTTTCTACGTCACGTATTTTGTACTAATATCTT
TGTGTTTACAGTCAAATTAATTCTAATTATCTCTCTAACAGCCTTGTATCGTATATGCAAATATGAAGGAATCATGGGAAATAGGCC
CTCTTCCTGCCCAACCGGTTGCGATCGCTCCGGTGCCCGTCAGTGGGCAGAGCGCACATCGCCCACAGTCCCCGAGAAGTTGG
GGGGAGGGGTCGGCAATTGAACGGGTGCCTAGAGAAGGTGGCGCGGGGTAAACTGGGAAAGTGATGTCGTGTACTGGCTCCG
CCTTTTTCCCGAGGGTGGGGGAGAACCGTATATAAGTGCAGTAGTCGCCGTGAACGTTCTTTTTCGCAACGGGTTTGCCGCCAG
AACACAGCTGAAGCTTCGAGGGGCTCGCATCTCTCCTTCACGCGCCCGCCGCCCTACCTGAGGCCGCCATCCACGCCGGTTGA
GTCGCGTTCTGCCGCCTCCCGCCTGTGGTGCCTCCTGAACTGCGTCCGCCGTCTAGGTAAGTTTAAAGCTCAGGTCGAGACCG
GGCCTTTGTCCGGCGCTCCCTTGGAGCCTACCTAGACTCAGCCGGCTCTCCACGCTTTGCCTGACCCTGCTTGCTCAACTCTAC
GTCTTTGTTTCGTTTTCTGTTCTGCGCCGTTACAGATCCAAGCTGTGACCGGCGCCTACTCTACAGATAGCGTTTAAACTTACGCT
TGCCACCATGGCTAGCGACTACAAGGACGACGACGACAAGGACAAGAAGCCCCTGAACACCCTGATCAGCGCCACAGGACT
GTGGATGTCCAGAACCGGCACCATCCACAAGATCAAGCACCACGAGGTGTCCCGGTCCAAAATCTACATCGAGATGGCCTGC
GGCGATCACCTGGTCGTCAACAACAGCAGAAGCAGCCGGACAGCCAGAGCCCTGCGGCACCACAAGTACAGAAAGACCTGC
AAGCGGTGCAGAGTGTCCGACGAGGACCTGAACAAGTTCCTGACCAAGGCCAACGAGGACCAGACCAGCGTGAAAGTGAAG
GTGGTGTCCGCCCCCACCCGGACCAAGAAAGCCATGCCCAAGAGCGTGGCCAGAGCCCCCAAGCCCCTGGAAAACACCGAA
GCCGCTCAGGCCCAGCCCAGCGGCAGCAAGTTCAGCCCCGCCATCCCCGTGTCTACCCAGGAAAGCGTCAGCGTCCCCGCC
AGCGTGTCCACCAGCATCTCTAGCATCTCAACCGGCGCCACAGCTTCTGCCCTGGTCAAGGGCAACACCAACCCCATCACCA
GCATGTCTGCCCCTGTGCAAGCCTCTGCCCCAGCCCTGACCAAGTCCCAGACCGACCGGCTGGAAGTGCTCCTGAACCCCAA
GGACGAGATCAGCCTGAACAGCGGCAAGCCCTTCCGGGAGCTGGAAAGCGAGCTGCTGAGCCGGCGGAAGAAGGACCTCC
AGCAAATCTACGCCGAGGAACGGGAGAACTACCTGGGCAAGCTGGAAAGAGAGATCACCCGGTTCTTCGTGGACCGGGGCT
TCCTGGAAATCAAGAGCCCCATCCTGATCCCCCTGGAGTACATCGAGCGGATGGGCATCGACAACGACACCGAGCTGAGCA
AGCAGATTTTCCGGGTGGACAAGAACTTCTGCCTGCGGCCCATGCTGGCCCCCAACCTGTACAACTACCTGCGGAAACTGGA
TCGCGCTCTGCCCGACCCCATCAAGATTTTCGAGATCGGCCCCTGCTACCGGAAAGAGAGCGACGGCAAAGAGCACCTGGA
AGAGTTTACAATGCTGAACTTTTGCCAGATGGGCAGCGGCTGCACCAGAGAGAACCTGGAATCCATCATCACCGACTTTCTGA
ACCACCTGGGGATCGACTTCAAGATCGTGGGCGACAGCTGCATGGTGTACGGCGACACCCTGGACGTGATGCACGGCGACC
TGGAACTGTCTAGCGCCGTCGTGGGACCCATCCCTCTGGACCGGGAGTGGGGCATCGATAAGCCCTGGATCGGAGCCGGCT
TCGGCCTGGAACGGCTGCTGAAAGTCAAGCACGACTTTAAGAACATCAAGCGGGCTGCCAGAAGCGAGAGCTACTACAACG
GCATCAGCACCAACCTGTGATGATAAGGATCCACTAGTCCAGTGTGGTGGAATTGACATTGATTATTGACTAGTTATTAATAGTA
ATCAATTACGGGGTCATTAGTTCATAGCCCATATATGGAGTTCCGCGTTACATAACTTACGGTAAATGGCCCGCCTGGCTGACCG
CCCAACGACCCCCGCCCATTGACGTCAATAATGACGTATGTTCCCATAGTAACGCCAATAGGGACTTTCCATTGACGTCAATGGG
TGGAGTATTTACGGTAAACTGCCCACTTGGCAGTACATCAAGTGTATCATATGCCAAGTACGCCCCCTATTGACGTCAATGACGG
TAAATGGCCCGCCTGGCATTATGCCCAGTACATGACCTTATGGGACTTTCCTACTTGGCAGTACATCTACGTATTAGTCATCGCT
ATTACCATGGTCGAGGTGAGCCCCACGTTCTGCTTCACTCTCCCCATCTCCCCCCCCTCCCCACCCCCAATTTTGTATTTATTTAT
TTTTTAATTATTTTGTGCAGCGATGGGGGCGGGGGGGGGGGGGGCGCGCGCCAGGCGGGGCGGGGCGGGGCGAGGGGCGG
GGCGGGGCGAGGCGGAGAGGTGCGGCGGCAGCCAATCAGAGCGGCGCGCTCCGAAAGTTTCCTTTTATGGCGAGGCGGCGG
CGGCGGCGGCCCTATAAAAAGCGAAGCGCGCGGCGGGCGGGAGTCGCTGCGTTGCCTTCGCCCCGTGCCCCGCTCCGCGCC
GCCTCGCGCCGCCCGCCCCGGCTCTGACTGACCGCGTTACTCCCACAGGTGAGCGGGCGGGACGGCCCTTCTCCTCCGGGCT
GTAATTAGCGCTTGGTTTAATGACGGCTCGTTTCTTTTCTGTGGCTGCGTGAAAGCCTTAAAGGGCTCCGGGAGGGCCCTTTGT
GCGGGGGGGAGCGGCTCGGGGGGTGCGTGCGTGTGTGTGTGCGTGGGGAGCGCCGCGTGCGGCCCGCGCTGCCCGGCGGC
TGTGAGCGCTGCGGGCGCGGCGCGGGGCTTTGTGCGCTCCGCGTGTGCGCGAGGGGAGCGCGGCCGGGGGCGGTGCCCCG
CGGTGCGGGGGGGCTGCGAGGGGAACAAAGGCTGCGTGCGGGGTGTGTGCGTGGGGGGGTGAGCAGGGGGTGTGGGCGCG
GCGGTCGGGCTGTAACCCCCCCCTGCACCCCCCTCCCCGAGTTGCTGAGCACGGCCCGGCTTCGGGTGCGGGGCTCCGTACG
GGGCGTGGCGCGGGGCTCGCCGTGCCGGGCGGGGGGTGGCGGCAGGTGGGGGTGCCGGGCGGGGCGGGGCCGCCTCGGG
CCGGGGAGGGCTCGGGGGAGGGGCGCGGCGGCCCCCGGAGCGCCGGCGGCTGTCGAGGCGCGGCGAGCCGCAGCCATTG
CCTTTTATGGTAATCGTGCGAGAGGGCGCAGGGACTTCCTTTGTCCCAAATCTGTGCGGAGCCGAAATCTGGGAGGCGCCGCC
GCACCCCCTCTAGCGGGCGCGGGGCGAAGCGGTGCGGCGCCGGCAGGAAGGAAATGGGCGGGGAGGGCCTTCGTGCGTCG
CCGCGCCGCCGTCCCCTTCTCCCTCTCCAGCCTCGGGGCTGTCCGCGGGGGGACGGCTGCCTTCGGGGGGGACGGGGCAGG
GCGGGGTTCGGCTTCTGGCGTGTGACCGGCGGCTCTAGAGCCTCTGCTAACCATGTTCATGCCTTCTTCTTTTTCCTACAGCTC
CTGGGCAACGTGCTGGTTATTGTGCTGTCTCATCATTTTGGCAAAGAATTCGCCACCATGGTGAGCAAGGGCGAGGAGGATAA
CATGGCCATCATCAAGGAGTTCATGCGCTTCAAGGTGCACATGGAGGGCTCCGTGAACGGCCACGAGTTCGAGATCGAGGG
CGAGGGCGAGGGCCGCCCCTACGAGGGCACCCAGACCGCCAAGCTGAAGGTGACCAAGGGTGGCCCCCTGCCCTTCGCCT
GGGACATCCTGTCCCCTCAGTTCATGTACGGCTCCAAGGCCTACGTGAAGCACCCCGCCGACATCCCCGACTACTTGAAGCT
GTCCTTCCCCGAGGGCTTCAAGTGGGAGCGCGTGATGAACTTCGAGGACGGCGGCGTGGTGACCGTGACCCAGGACTCCTC
CCTGCAGGACGGCGAGTTCATCTACAAGGTGAAGCTGCGCGGCACCAACTTCCCCTCCGACGGCCCCGTAATGCAGAAGAA
GACCATGGGCTGGGAGGCCTCCTCCGAGCGGATGTACCCCGAGGACGGCGCCCTGAAGGGCGAGATCAAGCAGAGGCTGA
AGCTGAAGGACGGCGGCCACTACGACGCTGAGGTCAAGACCACCTACAAGGCCAAGAAGCCCGTGCAGCTGCCCGGCGCCT
ACAACGTCAACATCAAGTTGGACATCACCTCCCACAACGAGGACTACACCATCGTGGAACAGTACGAACGCGCCGAGGGCC
GCCACTCCACCGGCGGCATGGACGAGCTGTACGCGTCTGGAGGCGCCACCAATTTCAGCCTGCTGAAACAGGCTGGCGACG
TGGAAGAGAACCCTGGACCTGGACAAAAGTCGGTGAGCAAGGGCGAGGAGCTGTTCACCGGGGTGGTGCCCATCCTGGTCG
AGCTGGACGGCGACGTAAACGGCCACAAGTTCAGCGTGTCCGGCGAGGGCGAGGGCGATGCCACCTACGGCAAGCTGACC
CTGAAGTTCATCTGCACCACCGGCAAGCTGCCCGTGCCCTGGCCCACCCTCGTGACCACCCTGACCTACGGCGTGCAGTGCT
TCAGCCGCTACCCCGACCACATGAAGCAGCACGACTTCTTCAAGTCCGCCATGCCCGAAGGCTACGTCCAGGAGCGCACCAT
CTTCTTCAAGGACGACGGCAACTACAAGACCCGCGCCGAGGTGAAGTTCGAGGGCGACACCCTGGTGAACCGCATCGAGCT

GAAGGGCATCGACTTCAAGGAGGACGGCAACATCCTGGGGCACAAGCTGGAGTACAACTACAACAGCCACAGGAGTCTATA
TCATGGCCGACAAGCAGAAGAACGGCATCAAGGTGAACTTCAAGATCCGCCACAACATCGAGGACGGCAGCGTGCAGCTCG
CCGACCACTACCAGCAGAACACCCCCATCGGCGACGGCCCCGTGCTGCTGCCCGACAACCACTACCTGAGCACCCAGTCCG
CCCTGAGCAAAGACCCCAACGAGAAGCGCGATCACATGGTCCTGCTGGAGTTCGTGACCGCCGCCGGGATCACTCTCGGCA
TGGACGAGCTGTACAAGTAAGAATTCACTCCTCAGGTGCAGGCTGCCTATCAGAAGGTGGTGGCTGGTGTGGCCAATGCCCTG
GCTCACAAATACCACTGAGATCTTTTTCCCTCTGCCAAAAATTATGGGGACATCATGAAGCCCCTTGAGCATCTGACTTCTGGCTA
ATAAAGGAAATTTATTTTCATTGCAATAGTGTGTTGGAATTTTTTGTGTCTCTCACTCGGAAGGACATATGGGAGGGTCGACAAT
CAACCTCTGGATTACAAAATTTGTGAAAGATTGACTGGTATTCTTAACTATGTTGCTCCTTTTACGCTATGTGGATACGCTGCTTTA
ATGCCTTTGTATCATGCGTTAACTAAACTTGTTTATTGCAGCTTATAATGGTTACAAATAAAGCAATAGCATCACAAATTTCACAAA
TAAAGCATTTTTTTCACTGCATTCTAGTTGTGGTTTGTCCAAACTCATCAATGTATCTTATCATGTCTGGAATTGACTCAAATGATG
TCAATTAGTCTATCAGAAGCTATCTGGTCTCCCTTCCGGGGGACAAGACATCCCTGTTTAATATTTAAACAGCAGTGTTCCCAAAC
TGGGTTCTTATATCCCTTGCTCTGGTCAACCAGGTTGCAGGGTTTCCTGTCCTCACAGGAACGAAGTCCCTAAAGAAACAGTGGC
AGCCAGGTTTAGCCCCGGAATTGACTGGATTCCTTTTTTAGGGCCCATTGGTATGGCTTTTTCCCCGTATCCCCCCAGGTGTCTG
CAGGCTCAAAGAGCAGCGAGAAGCGTTCAGAGGAAAGCGATCCCGTGCCACCTTCCCCGTGCCCGGGCTGTCCCCGCACGCT
GCCGGCTCGGGGATGCGGGGGGAGCGCCGGACCGGAGCGGAGCCCCGGGCGGCTCGCTGCTGCCCCCTAGCGGGGGAGGG
ACGTAATTACATCCCTGGGGGCTTTGGGGGGGGGCTGTCCCTGATATCTATAACAAGAAAATATATATATAATAAGTTATCACGTA
AGTAGAACATGAAATAACAATATAATTATCGTATGAGTTAAATCTTAAAAGTCACGTAAAAGATAATCATGCGTCATTTTGACTCAC
GCGGTCGTTATAGTTCAAAATCAGTGACACTTACCGCATTGACAAGCACGCCTCACGGGAGCTCCAAGCGGCGACTGAGATGTC
CTAAATGCACAGCGACGGATTCGCGCTATTTAGAAAGAGAGAGCAATATTTCAAGAATGCATGCGTCAATTTTACGCAGACTATC
TTTCTAGGGTTAATCTAGCTGCATCAGGATCATATCGTCGGGTCTTTTTTCCGGCTCAGTCATCGCCCAAGCTGGCGCTATCTGG
GCATCGGGGAGGAAGAAGCCCGTGCCTTTTCCCGCGAGGTTGAAGCGGCATGGAAAGAGTTTGCCGAGGATGACTGCTGCTGC
ATTGACGTTGAGCGAAAACGCACGTTTACCATGATGATTCGGGAAGGTGTGGCCATGCACGCCTTTAACGGTGAACTGTTCGTT
CAGGCCACCTGGGATACCAGTTCGTCGCGGCTTTTCCGGACACAGTTCCGGATGGTCAGCCCGAAGCGCATCAGCAACCCGAA
CAATACCGGCGACAGCCGGAACTGCCGTGCCGGTGTGCAGATTAATGACAGCGGTGCGGCGCTGGGATATTACGTCAGCGAG
GACGGGTATCCTGGCTGGATGCCGCAGAAATGGACATGGATACCCCGTGAGTTACCCGGCGGGCGCGCTTGGCGTAATCATGG
TCATAGCTGTTTCCTGTGTGAAATTGTTATCCGCTCACAATTCCACACAACATACGAGCCGGAAGCATAAAGTGTAAAGCCTGGG
GTGCCTAATGAGTGAGCTAACTCACATTAATTGCGTTGCGCTCACTGCCCGCTTTCCAGTCGGGAAACCTGTCGTGCCAGCTGC
ATTAATGAATCGGCCAACGCGCGGGGAGAGGCGGTTTGCGTATTGGGCGCTCTTCCGCTTCCTCGCTCACTGACTCGCTGCGC
TCGGTCGTTCGGCTGCGGCGAGCGGTATCAGCTCACTCAAAGGCGGTAATACGGTTATCCACAGAATCAGGGGATAACGCAGG
AAAGAACATGTGAGCAAAAGGCCAGCAAAAGGCCAGGAACCGTAAAAAGGCCGCGTTGCTGGCGTTTTTCCATAGGCTCCGCC
CCCCTGACGAGCATCACAAAAATCGACGCTCAAGTCAGAGGTGGCGAAACCCGACAGGACTATAAAGATACCAGGCGTTTCCCC
CTGGAAGCTCCCTCGTGCGCTCTCCTGTTCCGACCCTGCCGCTTACCGGATACCTGTCCGCCTTTCTCCCTTCGGGAAGCGTGG
CGCTTTCTCATAGCTCACGCTGTAGGTATCTCAGTTCGGTGTAGGTCGTTCGCTCCAAGCTGGGCTGTGTGCACGAACCCCCCG
TTCAGCCCGACCGCTGCGCCTTATCCGGTAACTATCGTCTTGAGTCCAACCCGGTAAGACACGACTTATCGCCACTGGCAGCAG
CCACTGGTAACAGGATTAGCAGAGCGAGGTATGTAGGCGGTGCTACAGAGTTCTTGAAGTGGTGGCCTAACTACGGCTACACTA
GAAGGACAGTATTTGGTATCTGCGCTCTGCTGAAGCCAGTTACCTTCGGAAAAAGAGTTGGTAGCTCTTGATCCGGCAAACAAAC
CACCGCTGGTAGCGGTGGTTTTTTTGTTTGCAAGCAGCAGATTACGCGCAGAAAAAAAGGATCTCAAGAAGATCCTTTGATCTTT
TCTACGGGGTCTGACGCTCAGTGGAACGAAAACTCACGTTAAGGGATTTTGGTCATGAGATTATCAAAAAGGATCTTCACCTAGA
TCCTTTTAAATTAAAAATGAAGTTTTAAATCAATCTAAAGTATATATGAGTAAACTTGGTCTGACAGTTACCAATGCTTAATCAGTGA
GGCACCTATCTCAGCGATCTGTCTATTTCGTTCATCCATAGTTGCCTGACTCCCCGTCGTGTAGATAACTACGATACGGGAGGGC
TTACCATCTGGCCCCAGTGCTGCAATGATACCGCGAGACCCACGCTCACCGGCTCCAGATTTATCAGCAATAAACCAGCCAGCC
GGAAGGGCCGAGCGCAGAAGTGGTCCTGCAACTTTATCCGCCTCCATCCAGTCTATTAATTGTTGCCGGGAAGCTAGAGTAAGT
AGTTCGCCAGTTAATAGTTTGCGCAACGTTGTTGCCATTGCTACAGGCATCGTGGTGTCACGCTCGTCGTTTGGTATGGCTTCAT
TCAGCTCCGGTTCCCAACGATCAAGGCGAGTTACATGATCCCCCATGTTGTGCAAAAAAGCGGTTAGCTCCTTCGGTCCTCCGA
TCGTTGTCAGAAGTAAGTTGGCCGCAGTGTTATCACTCATGGTTATGGCAGCACTGCATAATTCTCTTACTGTCATGCCATCCGTA
AGATGCTTTTCTGTGACTGGTGAGTACTCAACCAAGTCATTCTGAGAATAGTGTATGCGGCGACCGAGTTGCTCTTGCCCGGCGT
CAATACGGGATAATACCGCGCCACATAGCAGAACTTTAAAAGTGCTCATCATTGGAAAACGTTCTTCGGGGCGAAAACTCTCAAG
GATCTTACCGCTGTTGAGATCCAGTTCGATGTAACCCACTCGTGCACCCAACTGATCTTCAGCATCTTTTACTTTCACCAGCGTTT
CTGGGTGAGCAAAAACAGGAAGGCAAAATGCCGCAAAAAAGGGAATAAGGGCGACACGGAAATGTTGAATACTCAT

2.2.19 Vector 74: 4x(Tyr tRNA$_{CUA}$) TyrRS* mCherry-P2A-eGFP(150TAG)

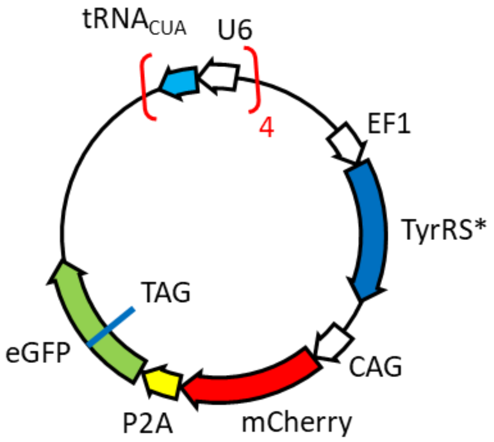

ACTCTTCCTTTTTCAATATTATTGAAGCATTTATCAGGGTTATTGTCTCATGAGCGGATACATATTTGAATGTATTTAGAAAAATAAA
CAAATAGGGGTTCCGCGCACATTTCCCCGAAAAGTGCCACCTAAATTGTAAGCGTTAATATTTTGTTAAAATTCGCGTTAAATTTTT
GTTAAATCAGCTCATTTTTTAACCAATAGGCCGAAATCGGCAAAATCCCTTATAAATCAAAAGAATAGACCGAGATAGGGTTGAGT
GTTGTTCCAGTTTGGAACAAGAGTCCACTATTAAAGAACGTGGACTCCAACGTCAAAGGGCGAAAAACCGTCTATCAGGGCGAT

GGCCCACTACGTGAACCATCACCCTAATCAAGTTTTTTGGGGTCGAGGTGCCGTAAAGCACTAAATCGGAACCCTAAAGGGAGC
CCCCGATTTAGAGCTTGACGGGGAAAGCCGGCGAACGTGGCGAGAAAGGAAGGGAAGAAAGCGAAAGGAGCGGGCGCTAGGG
CGCTGGCAAGTGTAGCGGTCACGCTGCGCGTAACCACCACACCCGCCGCGCTTAATGCGCCGCTACAGGGCGCGTCCCATTC
GCCATTCAGGCTGCGCAACTGTTGGGAAGGGCGATCGGTGCGGGCCTCTTCGCTATTACGCCAGCTGGCGAAAGGGGGATGT
GCTGCAAGGCGATTAAGTTGGGTAACGCCAGGGTTTTCCCAGTCACGACGTTGTAAAACGACGGCCAGTGAGCGCGCCTCGTT
CATTCACGTTTTTGAACCCGTGGAGGACGGGCAGACTCGCGGTGCAAATGTGTTTTACAGCGTGATGGAGCAGATGAAGATGCT
CGACACGCTGCAGAACACGCAGCTAGATTAACCCTAGAAAGATAATCATATTGTGACGTACGTTAAAGATAATCATGCGTAAAATT
GACGCATGTGTTTTATCGGTCTGTATATCGAGGTTTATTTATTAATTTGAATAGATATTAAGTTTTATTATATTTACACTTACATACTA
ATAATAAATTCAACAAACAATTTATTTATGTTTATTTATTTATTAAAAAAAAACAAAAACTCAAAATTTCTTCTATAAAGTAACAAAACTT
TTATGAGGGACAGCCCCCCCCCAAAGCCCCCAGGGATGTAATTACGTCCCTCCCCCGCTAGGGGGCAGCAGCGAGCCGCCCG
GGGCTCCGCTCCGGTCCGGCGCTCCCCCCGCATCCCCGAGCCGGCAGCGTGCGGGGACAGCCCGGGCACGGGGAAGGTGG
CACGGGATCGCTTTCCTCTGAACGCTTCTCGCTGCTCTTTGAGCCTGCAGACACCTGGGGGGATACGGGGAAAAGGCCTCTTAA
GAAAAACCGCACTTGTC**TGGTGGGGGAAGGATTCGAACCTTCGAAGTCTGTGACGGCAGATTTAGAGTCTGCTCCCTTTGGCC**
GCTCGGGAACCCCACCGGTGTTTCGTCCTTTCCACAAGATATATAAAGCCAAGAAATCGAAATACTTTCAAGTTACGGTAAGCAT
ATGATAGTCCATTTTAAAACATAATTTTAAAACTGCAAACTACCCAAGAAATTATTACTTTCTACGTCACGTATTTTGTACTAATATC
TTTGTGTTTACAGTCAAATTAATTCTAATTATCTCTCTAACAGCCTTGTATCGTATATGCAAATATGAAGGAATCATGGGAAATAGG
CCCTCTTCCTGCCCAATCGACGAACTTGTGGCCGTTTACCCTCTTAAGAAAAACCGCACTTGTC**TGGTGGGGGAAGGATTCGAA**
CCTTCGAAGTCTGTGACGGCAGATTTAGAGTCTGCTCCCTTTGGCCGCTCGGGAACCCCACCGGTGTTTCGTCCTTTCCACAA
GATATATAAAGCCAAGAAATCGAAATACTTTCAAGTTACGGTAAGCATATGATAGTCCATTTTAAAACATAATTTTAAAACTGCAAA
CTACCCAAGAAATTATTACTTTCTACGTCACGTATTTTGTACTAATATCTTTGTGTTTACAGTCAAATTAATTCTAATTATCTCTCTAA
CAGCCTTGTATCGTATATGCAAATATGAAGGAATCATGGGAAATAGGCCCTCTTCCTGCCCAATTCGGCAAGTTCTCCACCAGCC
TCTTAAGAAAAACCGCACTTGTC**TGGTGGGGGAAGGATTCGAACCTTCGAAGTCTGTGACGGCAGATTTAGAGTCTGCTCCCTT**
TGGCCGCTCGGGAACCCCACCGGTGTTTCGTCCTTTCCACAAGATATATAAAGCCAAGAAATCGAAATACTTTCAAGTTACGGTA
AGCATATGATAGTCCATTTTAAAACATAATTTTAAAACTGCAAACTACCCAAGAAATTATTACTTTCTACGTCACGTATTTTGTACTA
ATATCTTTGTGTTTACAGTCAAATTAATTCTAATTATCTCTCTAACAGCCTTGTATCGTATATGCAAATATGAAGGAATCATGGGAA
ATAGGCCCTCTTCCTGCCCAACCTCTTAAGAAAAACCGCACTTGTCCCTAAAAACCGCACTTGTC**TGGTGGGGGAAGGATTCGA**
ACCTTCGAAGTCTGTGACGGCAGATTTAGAGTCTGCTCCCTTTGGCCGCTCGGGAACCCCACCGGTGTTTCGTCCTTTCCACA
AGATATATAAAGCCAAGAAATCGAAATACTTTCAAGTTACGGTAAGCATATGATAGTCCATTTTAAAACATAATTTTAAAACTGCAA
ACTACCCAAGAAATTATTACTTTCTACGTCACGTATTTTGTACTAATATCTTTGTGTTTACAGTCAAATTAATTCTAATTATCTCTCTA
ACAGCCTTGTATCGTATATGCAAATATGAAGGAATCATGGGAAATAGGCCCTCTTCCTGCCCAACCGGTTGCGATCGCTCCGGT
GCCCGTCAGTGGGCAGAGCGCACATCGCCCACAGTCCCCGAGAAGTTGGGGGGAGGGGTCGGCAATTGAACGGGTGCCTAGA
GAAGGTGGCGCGGGGTAAACTGGGAAAGTGATGTCGTGTACTGGCTCCGCCTTTTTCCCGAGGGTGGGGGAGAACCGTATATA
AGTGCAGTAGTCGCCGTGAACGTTCTTTTTCGCAACGGGTTTGCCGCCAGAACACAGCTGAAGCTTCGAGGGGCTCGCATCTCT
CCTTCACGCGCCCGCCGCCCTACCTGAGGCCGCCATCCACGCCGGTTGAGTCGCGTTCTGCCGCCTCCCGCCTGTGGTGCCT
CCTGAACTGCGTCCGCCGTCTAGGTAAGTTTAAAGCTCAGGTCGAGACCGGGCCTTTGTCCGGCGCTCCCTTGGAGCCTACCTA
GACTCAGCCGGCTCTCCACGCTTTGCCTGACCCTGCTTGCTCAACTCTACGTCTTTGTTTCGTTTTCTGTTCTGCGCCGTTACAG
ATCCAAGCTGTGACCGGCGCCTACTCTACAGATAGCGTTTAAACTTACGCTTGCCACC**ATGGCTAGCGACTACAAGGACGACG**
ACGACAAGGCAAGCAGTAACTTGATTAAACAATTGCAAGAGCGGGGGCTGGTAGCCCAGGTGACGGACGAGGAAGCGTTAG
CAGAGCGACTGGCGCAAGGCCCGATCGCACTCGTGTGTGGCTTCGATCCTACCGCTGACAGCTTGCATTTGGGGCATCTTGTT
CCATTGTTATGCCTGAAACGCTTCCAGCAGGCGGGCCACAAGCCGGTTGCGCTGGTAGGCGGCGCGACGGGTCTGATTGGC
GACCCGAGCTTCAAAGCTGCCGAGCGTAAGCTGAACACCGAAGAAACTGTTCAGGAGTGGGTGGACAAAATCCGTAAGCAG
GTTGCCCCGTTCCTCGATTTCGACTGTGGAGAAAACTCTGCTATCGCGGCCAATAATTATGACTGGTTCGGCAATATGAATGT
GCTGACCTTCCTGCGCGATATTGGCAAACACTTCTCCGTTAACCAGATGATCAACAAAGAAGCGGTTAAGCAGCGTCTCAACC
GTGAAGATCAGGGGATTTCGTTCACTGAGTTTTCCTACAACCTGCTGCAGGGTTATAGTATGGCCTGTTTGAACAAACAGTAC
GGTGTGGTGCTGCAAATTGGTGGTTCTGACCAGTGGGGTAACATCACTTCTGGTATCGACCTGACCCGTCGTCTGCATCAGAA
TCAGGTGTTTGGCCTGACCGTTCCGCTGATCACTAAAGCAGATGGCACCAAATTTGGTAAAACTGAAGGCGGCGCAGTCTGG
TTGGATCCGAAGAAAACCAGCCCGTACAAATTCTACCAGTTCTGGATCAACACTGCGCGTGCCGACGTTTACCGCTTCCTGAA
GTTCTTCACCTTTATGAGCATTGAAGAGATCAACGCCCTGGAAGAAGAAGATAAAAACAGCGGTAAAGCACCGCGCGCCCAG
TATGTACTGGCGGAGCAGGTGACTCGTCTGGTTCACGGTGAAGAAGGTTTACAGGCGGCAAAACGTATTACCGAATGCCTGT
TCAGCGGTTCTTTGAGTGCGCTGAGTGAAGCGGACTTCGAACAGCTGGCGCAGGACGGCGTACCGATGGTTGAGATGGAAAA
GGGCGCAGACCTGATGCAGGCACTGGTCGATTCTGAACTGCAACCTTCCCGTGGTCAGGCACGTAAAACTATCGCCTCCAAT
GCCATCACCATTAACGGTGAAAAACAGTCCGATCCTGAATACTTCTTTAAAGAAGAAGATCGTCTGTTTGGTCGTTTTACCTTA
CTGCGTCGCGGTAAAAAGAATTACTGTCTGATTTGCTGGAAAGGGCCCGTTTAATGATAAGGATCCACTAGTCCAGTGTGGTG
GAATTGACATTGATTATTGACTAGTTATTAATAGTAATCAATTACGGGGTCATTAGTTCATAGCCCATATATGGAGTTCCGCGTTAC
ATAACTTACGGTAAATGGCCCGCCTGGCTGACCGCCCAACGACCCCCGCCCATTGACGTCAATAATGACGTATGTTCCCATAGT
AACGCCAATAGGGACTTTCCATTGACGTCAATGGGTGGAGTATTTACGGTAAACTGCCCACTTGGCAGTACATCAAGTGTATCAT
ATGCCAAGTACGCCCCCTATTGACGTCAATGACGGTAAATGGCCCGCCTGGCATTATGCCCAGTACATGACCTTATGGGACTTTC
CTACTTGGCAGTACATCTACGTATTAGTCATCGCTATTACCATGGTCGAGGTGAGCCCCACGTTCTGCTTCACTCTCCCCATCTC
CCCCCCCTCCCCACCCCCAATTTTGTATTTATTTATTTTTTAATTATTTTGTGCAGCGATGGGGGCGGGGGGGGGGGGGGCGCG
CGCCAGGCGGGGCGGGGCGGGGCGAGGGGCGGGGCGGGGCGAGGCGGAGAGGTGCGGCGGCAGCCAATCAGAGCGGCGC
GCTCCGAAAGTTTCCTTTTATGGCGAGGCGGCGGCGGCGGCGGCCCTATAAAAAGCGAAGCGCGCGGCGGGCGGGAGTCGCT
GCGTTGCCTTCGCCCCGTGCCCCGCTCCGCGCCGCCTCGCGCCGCCCGCCCCGGCTCTGACTGACCGCGTTACTCCCACAGG
TGAGCGGGCGGGACGGCCCTTCTCCTCCGGGCTGTAATTAGCGCTTGGTTTAATGACGGCTCGTTTCTTTTCTGTGGCTGCGTG
AAAGCCTTAAAGGGCTCCGGGAGGGCCCTTTGTGCGGGGGGGAGCGGCTCGGGGGGTGCGTGCGTGTGTGTGTGCGTGGGG
AGCGCCGCGTGCGGCCCGCGCTGCCCGGCGGCTGTGAGCGCTGCGGGCGCGGCGCGGGGCTTTGTGCGCTCCGCGTGTGC
GCGAGGGGAGCGCGGCCGGGGGCGGTGCCCCGCGGTGCGGGGGGGCTGCGAGGGGAACAAAGGCTGCGTGCGGGGTGTGT
GCGTGGGGGGGTGAGCAGGGGGTGTGGGCGCGGCGGTCGGGCTGTAACCCCCCCCTGCACCCCCCTCCCCGAGTTGCTGAG
CACGGCCCGGCTTCGGGTGCGGGGCTCCGTACGGGGCGTGGCGCGGGGCTCGCCGTGCCGGGCGGGGGGTGGCGGCAGGT
GGGGGTGCCGGGCGGGGCGGGGCCGCCTCGGGCCGGGGAGGGCTCGGGGGAGGGGCGCGGCGGCCCCCGGAGCGCCGG
CGGCTGTCGAGGCGCGGCGAGCCGCAGCCATTGCCTTTTATGGTAATCGTGCGAGAGGGCGCAGGGACTTCCTTTGTCCCAAA
TCTGTGCGGAGCCGAAATCTGGGAGGCGCCGCCGCACCCCCTCTAGCGGGCGCGGGGCGAAGCGGTGCGGCGCCGGCAGGA
AGGAAATGGGCGGGGAGGGCCTTCGTGCGTCGCCGCGCCGCCGTCCCCTTCTCCCTCTCCAGCCTCGGGGCTGTCCGCGGG
GGGACGGCTGCCTTCGGGGGGGACGGGGCAGGGCGGGGTTCGGCTTCTGGCGTGTGACCGGCGGCTCTAGAGCCTCTGCTA
ACCATGTTCATGCCTTCTTCTTTTTCCTACAGCTCCTGGGCAACGTGCTGGTTATTGTGCTGTCTCATCATTTTGGCAAAGAATTC

GCCACCATGGTGAGCAAGGGCGAGGAGGATAACATGGCCATCATCAAGGAGTTCATGCGCTTCAAGGTGCACATGGAGGGC
TCCGTGAACGGCCACGAGTTCGAGATCGAGGGCGAGGGCGAGGGCCGCCCCTACGAGGGCACCCAGACCGCCAAGCTGAA
GGTGACCAAGGGTGGCCCCCTGCCCTTCGCCTGGGACATCCTGTCCCCTCAGTTCATGTACGGCTCCAAGGCCTACGTGAAG
CACCCCGCCGACATCCCCGACTACTTGAAGCTGTCCTTCCCCGAGGGCTTCAAGTGGGAGCGCGTGATGAACTTCGAGGACG
GCGGCGTGGTGACCGTGACCCAGGACTCCTCCCTGCAGGACGGCGAGTTCATCTACAAGGTGAAGCTGCGCGGCACCAACT
TCCCCTCCGACGGCCCCGTAATGCAGAAGAAGACCATGGGCTGGGAGGCCTCCTCCGAGCGGATGTACCCCGAGGACGGCG
CCCTGAAGGGCGAGATCAAGCAGAGGCTGAAGCTGAAGGACGGCGGCCACTACGACGCTGAGGTCAAGACCACCTACAAG
GCCAAGAAGCCCGTGCAGCTGCCCGGCGCCTACAACGTCAACATCAAGTTGGACATCACCTCCCACAACGAGGACTACACC
ATCGTGGAACAGTACGAACGCGCCGAGGGCCGCCACTCCACCGGCGGCATGGACGAGCTGTACGCGTCTGGAGGCGCCAC
CAATTTCAGCCTGCTGAAACAGGCTGGCGACGTGGAAGAGAACCCTGGACCTGGACAAAAGTCGGTGAGCAAGGGCGAGGA
GCTGTTCACCGGGGTGGTGCCCATCCTGGTCGAGCTGGACGGCGACGTAAACGGCCACAAGTTCAGCGTGTCCGGCGAGGG
CGAGGGCGATGCCACCTACGGCAAGCTGACCCTGAAGTTCATCTGCACCACCGGCAAGCTGCCCGTGCCCTGGCCCACCCT
CGTGACCACCCTGACCTACGGCGTGCAGTGCTTCAGCCGCTACCCCGACCACATGAAGCAGCACGACTTCTTCAAGTCCGCC
ATGCCCGAAGGCTACGTCCAGGAGCGCACCATCTTCTTCAAGGACGACGGCAACTACAAGACCCGCGCCGAGGTGAAGTTC
GAGGGCGACACCCTGGTGAACCGCATCGAGCTGAAGGGCATCGACTTCAAGGAGGACGGCAACATCCTGGGGCACAAGCTG
GAGTACAACTACAACAGCCACTAGGTCTATATCATGGCCGACAAGCAGAAGAACGGCATCAAGGTGAACTTCAAGATCCGCC
ACAACATCGAGGACGGCAGCGTGCAGCTCGCCGACCACTACCAGCAGAACACCCCCATCGGCGACGGCCCCGTGCTGCTGC
CCGACAACCACTACCTGAGCACCCAGTCCGCCCTGAGCAAAGACCCCAACGAGAAGCGCGATCACATGGTCCTGCTGGAGT
TCGTGACCGCCGCCGGGATCACTCTCGGCATGGACGAGCTGTACAAGTAAGAATTCACTCCTCAGGTGCAGGCTGCCTATCAG
AAGGTGGTGGCTGGTGTGGCCAATGCCCTGGCTCACAAATACCACTGAGATCTTTTTCCCTCTGCCAAAAATTATGGGGACATCA
TGAAGCCCCTTGAGCATCTGACTTCTGGCTAATAAAGGAAATTTATTTTCATTGCAATAGTGTGTTGGAATTTTTTGTGTCTCTCAC
TCGGAAGGACATATGGGAGGGGTCGACAATCAACCTCTGGATTACAAAATTTGTGAAAGATTGACTGGTATTCTTAACTATGTTG
CTCCTTTTACGCTATGTGGATACGCTGCTTTAATGCCTTTGTATCATGCGTTAACTAAACTTGTTTATTGCAGCTTATAATGGTTAC
AAATAAAGCAATAGCATCACAAATTTCACAAATAAAGCATTTTTTTCACTGCATTCTAGTTGTGGTTTGTCCAAACTCATCAATGTAT
CTTATCATGTCTGGAATTGACTCAAATGATGTCAATTAGTCTATCAGAAGCTATCTGGTCTCCCTTCCGGGGGACAAGACATCCCT
GTTTAATATTTAAACAGCAGTGTTCCCAAACTGGGTTCTTATATCCCTTGCTCTGGTCAACCAGGTTGCAGGGTTTCCTGTCCTCA
CAGGAACGAAGTCCCTAAAGAAACAGTGGCAGCCAGGTTTAGCCCCGGAATTGACTGGATTCCTTTTTTAGGGCCCATTGGTAT
GGCTTTTTCCCCGTATCCCCCCAGGTGTCTGCAGGCTCAAAGAGCAGCGAGAAGCGTTCAGAGGAAAGCGATCCCGTGCCACC
TTCCCCGTGCCCGGGCTGTCCCCGCACGCTGCCGGCTCGGGGATGCGGGGGGAGCGCCGGACCGGAGCGGAGCCCCGGGC
GGCTCGCTGCTGCCCCCTAGCGGGGGAGGGACGTAATTACATCCCTGGGGGCTTTGGGGGGGGGCTGTCCCTGATATCTATAA
CAAGAAAATATATATATAATAAGTTATCACGTAAGTAGAACATGAAATAACAATATAATTATCGTATGAGTTAAATCTTAAAAGTCAC
GTAAAAGATAATCATGCGTCATTTTGACTCACGCGGTCGTTATAGTTCAAAATCAGTGACACTTACCGCATTGACAAGCACGCCTC
ACGGGAGCTCCAAGCGGCGACTGAGATGTCCTAAATGCACAGCGACGGATTCGCGCTATTTAGAAAGAGAGAGCAATATTTCAA
GAATGCATGCGTCAATTTTACGCAGACTATCTTTCTAGGGTTAATCTAGCTGCATCAGGATCATATCGTCGGGTCTTTTTTCCGGC
TCAGTCATCGCCCAAGCTGGCGCTATCTGGGCATCGGGGAGGAAGAAGCCCGTGCCTTTTCCCGCGAGGTTGAAGCGGCATGG
AAAGAGTTTGCCGAGGATGACTGCTGCTGCATTGACGTTGAGCGAAAACGCACGTTTACCATGATGATTCGGGAAGGTGTGGCC
ATGCACGCCTTTAACGGTGAACTGTTCGTTCAGGCCACCTGGGATACCAGTTCGTCGCGGCTTTTCCGGACACAGTTCCGGATG
GTCAGCCCGAAGCGCATCAGCAACCCGAACAATACCGGCGACAGCCGGAACTGCCGTGCCGGTGTGCAGATTAATGACAGCGG
TGCGGCGCTGGGATATTACGTCAGCGAGGACGGGTATCCTGGCTGGATGCCGCAGAAATGGACATGGATACCCCGTGAGTTAC
CCGGCGGGCGCGCTTGGCGTAATCATGGTCATAGCTGTTTCCTGTGTGAAATTGTTATCCGCTCACAATTCCACACAACATACGA
GCCGGAAGCATAAAGTGTAAAGCCTGGGGTGCCTAATGAGTGAGCTAACTCACATTAATTGCGTTGCGCTCACTGCCCGCTTTC
CAGTCGGGAAACCTGTCGTGCCAGCTGCATTAATGAATCGGCCAACGCGCGGGGAGAGGCGGTTTGCGTATTGGGCGCTCTTC
CGCTTCCTCGCTCACTGACTCGCTGCGCTCGGTCGTTCGGCTGCGGCGAGCGGTATCAGCTCACTCAAAGGCGGTAATACGGT
TATCCACAGAATCAGGGGATAACGCAGGAAAGAACATGTGAGCAAAAGGCCAGCAAAAGGCCAGGAACCGTAAAAAGGCCGCG
TTGCTGGCGTTTTTCCATAGGCTCCGCCCCCCTGACGAGCATCACAAAAATCGACGCTCAAGTCAGAGGTGGCGAAACCCGACA
GGACTATAAAGATACCAGGCGTTTCCCCCTGGAAGCTCCCTCGTGCGCTCTCCTGTTCCGACCCTGCCGCTTACCGGATACCTG
TCCGCCTTTCTCCCTTCGGGAAGCGTGGCGCTTTCTCATAGCTCACGCTGTAGGTATCTCAGTTCGGTGTAGGTCGTTCGCTCC
AAGCTGGGCTGTGTGCACGAACCCCCCGTTCAGCCCGACCGCTGCGCCTTATCCGGTAACTATCGTCTTGAGTCCAACCCGGTA
AGACACGACTTATCGCCACTGGCAGCAGCCACTGGTAACAGGATTAGCAGAGCGAGGTATGTAGGCGGTGCTACAGAGTTCTTG
AAGTGGTGGCCTAACTACGGCTACACTAGAAGGACAGTATTTGGTATCTGCGCTCTGCTGAAGCCAGTTACCTTCGGAAAAAGA
GTTGGTAGCTCTTGATCCGGCAAACAAACCACCGCTGGTAGCGGTGGTTTTTTTGTTTGCAAGCAGCAGATTACGCGCAGAAAAA
AAGGATCTCAAGAAGATCCTTTGATCTTTTCTACGGGGTCTGACGCTCAGTGGAACGAAAACTCACGTTAAGGGATTTTGGTCAT
GAGATTATCAAAAAGGATCTTCACCTAGATCCTTTTAAATTAAAAATGAAGTTTTAAATCAATCTAAAGTATATATGAGTAAACTTGG
TCTGACAGTTACCAATGCTTAATCAGTGAGGCACCTATCTCAGCGATCTGTCTATTTCGTTCATCCATAGTTGCCTGACTCCCCGT
CGTGTAGATAACTACGATACGGGAGGGCTTACCATCTGGCCCCAGTGCTGCAATGATACCGCGAGACCCACGCTCACCGGCTC
CAGATTTATCAGCAATAAACCAGCCAGCCGGAAGGGCCGAGCGCAGAAGTGGTCCTGCAACTTTATCCGCCTCCATCCAGTCTA
TTAATTGTTGCCGGGAAGCTAGAGTAAGTAGTTCGCCAGTTAATAGTTTGCGCAACGTTGTTGCCATTGCTACAGGCATCGTGGT
GTCACGCTCGTCGTTTGGTATGGCTTCATTCAGCTCCGGTTCCCAACGATCAAGGCGAGTTACATGATCCCCCATGTTGTGCAAA
AAAGCGGTTAGCTCCTTCGGTCCTCCGATCGTTGTCAGAAGTAAGTTGGCCGCAGTGTTATCACTCATGGTTATGGCAGCACTG
CATAATTCTCTTACTGTCATGCCATCCGTAAGATGCTTTTCTGTGACTGGTGAGTACTCAACCAAGTCATTCTGAGAATAGTGTAT
GCGGCGACCGAGTTGCTCTTGCCCGGCGTCAATACGGGATAATACCGCGCCACATAGCAGAACTTTAAAAGTGCTCATCATTGG
AAAACGTTCTTCGGGGCGAAAACTCTCAAGGATCTTACCGCTGTTGAGATCCAGTTCGATGTAACCCACTCGTGCACCCAACTGA
TCTTCAGCATCTTTTACTTTCACCAGCGTTTCTGGGTGAGCAAAAACAGGAAGGCAAAATGCCGCAAAAAAGGGAATAAGGGCGA
CACGGAAATGTTGAATACTCAT
